# Patient blood management interventions do not lead to important clinical benefits or cost-effectiveness for major surgery: a network meta-analysis

**DOI:** 10.1016/j.bja.2020.04.087

**Published:** 2020-06-30

**Authors:** Marius A. Roman, Riccardo G. Abbasciano, Suraj Pathak, Shwe Oo, Syabira Yusoff, Marcin Wozniak, Saqib Qureshi, Florence Y. Lai, Tracy Kumar, Toby Richards, Guiqing Yao, Lise Estcourt, Gavin J. Murphy

**Affiliations:** 1 Department of Cardiovascular Sciences and National Institute for Health Research Leicester Biomedical Research Unit in Cardiovascular Medicine, College of Life Sciences, University of Leicester, Leicester, UK; 2 Department of Cardiac Surgery, Liverpool Heart and Chest Hospital NHS Foundation Trust, Liverpool, UK; 3 Department of Cardiothoracic Surgery, University Hospitals of Nottingham, Nottingham, UK; 4 Faculty of Health and Medical Sciences, University of Western Australia, Perth, Australia; 5 Biostatistics Research Group, Department of Health Sciences, College of Life Sciences, University of Leicester, Leicester, UK; 6 Haematology/Transfusion Medicine, NHS Blood, and Transplant, John Radcliffe Hospital, Headington, Oxford, UK

**Keywords:** bleeding, cost, effectiveness, haematology, network, patient blood management, surgery, transfusion

## Abstract

**Background:**

Patient blood management (PBM) interventions aim to improve clinical outcomes by reducing bleeding and transfusion. We assessed whether existing evidence supports the routine use of combinations of these interventions during and after major surgery.

**Methods:**

Five systematic reviews and a National Institute of Health and Care Excellence health economic review of trials of common PBM interventions enrolling participants of any age undergoing surgery were updated. The last search was on June 1, 2019. Studies in trauma, burns, gastrointestinal haemorrhage, gynaecology, dentistry, or critical care were excluded. The co-primary outcomes were: risk of receiving red cell transfusion and 30-day or hospital all-cause mortality. Treatment effects were estimated using random-effects models and risk ratios (RR) with 95% confidence intervals (CIs). Heterogeneity assessments used *I*^2^. Network meta-analyses used a frequentist approach. The protocol was registered prospectively (PROSPERO CRD42018085730).

**Results:**

Searches identified 393 eligible randomised controlled trials enrolling 54 917 participants. PBM interventions resulted in a reduction in exposure to red cell transfusion (RR=0.60; 95% CI 0.57, 0.63; *I*^2^=77%), but had no statistically significant treatment effect on 30-day or hospital mortality (RR=0.93; 95% CI 0.81, 1.07; *I*^2^=0%). Treatment effects were consistent across multiple secondary outcomes, sub-groups and sensitivity analyses that considered clinical setting, type of intervention, and trial quality. Network meta-analysis did not demonstrate additive benefits from the use of multiple interventions. No trial demonstrated that PBM was cost-effective.

**Conclusions:**

In randomised trials, PBM interventions do not have important clinical benefits beyond reducing bleeding and transfusion in people undergoing major surgery.

**Editor's key points**

• The authors assessed the evidence base for routine blood management interventions in patients undergoing major surgery.
• This network meta-analysis included 393 eligible trials (54 917 participants). Interventions alone or in combination reduced transfusion and bleeding but did not affect mortality, other clinical outcomes, or cost-effectiveness.
• Patient blood management guidelines should report uncertainty with respect to improving clinical outcomes after major surgery beyond reducing transfusion rates and bleeding.

Anaemia, coagulopathy, and severe bleeding are common during and after major surgery where they are associated with transfusion of allogeneic blood components and adverse clinical outcomes.^1^ Patient blood management (PBM) describes the application of personalised, evidence-based, care bundles of interventions that reduce bleeding and transfusion intending to improve clinical outcomes.^2^^,^^3^ Three common interventions to reduce anaemia include pre-surgery parenteral or oral iron therapy, cell salvage devices that collect blood lost from the surgical field and wash it for autotransfusion, or the adoption of restrictive (lower) haemoglobin thresholds for red cell transfusion. Two common interventions used to prevent or manage bleeding are the antifibrinolytic drug tranexamic acid or point-of-care diagnostic test based algorithms for the personalised treatment of coagulopathy. Randomised trials evaluating these PBM interventions have not demonstrated important clinical benefits.^3^, ^4^, ^5^, ^6^, ^7^, ^8^, ^9^ However, treatment guidelines recommend the routine use of these interventions in patients undergoing surgery, albeit acknowledging Low to Moderate certainty as to the evidence.^3^^,^^4^ To assess whether existing evidence supports the routine administration of PBM interventions, we performed a systematic review and network meta-analysis of randomised trials that have evaluated these five PBM interventions administered alone or in combination to patients undergoing surgery. Outcomes of interest included treatment effects on transfusion and bleeding, measures of clinical effectiveness, and cost-effectiveness.

## Methods

Five systematic reviews, plus a National Institute of Health and Care Excellence (NICE, UK) health economic review^4^ of trials of common PBM interventions (pre-surgery iron administration,^9^ cell salvage and autotransfusion,^8^ restrictive transfusion thresholds,^5^ tranexamic acid,^7^ and point of care testing for coagulopathy^6^) were updated using the methods described in the *Cochrane Handbook for Systematic Reviews of Interventions*.^10^ The review protocol was registered prospectively (PROSPERO, CRD42018085730) and is available at https://www.crd.york.ac.uk/prospero/display_record.php?RecordID=85730. Detailed methods are included in the Supplementary Appendix.

Peer-review published RCTs were included, irrespective of blinding, language, date of publication, and sample size. We excluded abstracts, unpublished trials, and cluster randomised trials.

The population consisted of patients of any age undergoing surgery in the following fields: cardiovascular, neoplastic, orthopaedic, gastrointestinal, urology, organ transplantation, plastic, or maxillofacial surgery. The following interventions were considered: Interventions targeting anaemia; pre-surgery iron administration, perioperative cell salvage, the use of restrictive red cell transfusion thresholds, and interventions targeting bleeding; and tranexamic acid, point-of-care testing for coagulopathy. The control group consisted of patients not receiving the intervention or alternative goal-directed therapy.

The following additional, prespecified, exclusion criteria were applied: trials with patients undergoing trauma, burns or gastrointestinal haemorrhage, gynaecological procedures, dental procedures, or critical care patients; trials that used unwashed autologous red cells in trials of cell salvage; trials comparing different drug formulations or doses without a control group; trials without placebo or no treatment controls; and trials that did not report the pre-specified co-primary outcomes or trials that were not peer-reviewed. In trials comparing multiple drug formulations *vs* controls, the intravenous group was included if present; otherwise, oral or topical formulations were included.

The outcomes included:

• Measures of transfusion and bleeding; risk of receiving red cell transfusion (co-primary outcome), perioperative blood loss, re-operation for bleeding, numbers of red cells transfused, risk of receiving non-red cell components.
• Measures of clinical effectiveness; 30-day or hospital all-cause mortality (co-primary outcome), acute brain injury (stroke, transient ischaemic attack [TIA]), myocardial infarction, low cardiac output, acute kidney injury stage 3 (or requiring hemofiltration), sepsis and infection, as reported by study authors.
• Measures of resource use and cost-effectiveness; ICU and hospital length of stay, treatment costs, cost-utility analyses.

The electronic searches were updated for the following reviews from the final search date recorded in their respective publications until June 1, 2019: Cochrane review of iron therapy in patients without chronic kidney disease,^11^ Cochrane review of restrictive red cell transfusion thresholds,^5^ Cochrane review of cell salvage,^12^ systematic reviews of tranexamic acid in surgical patients,^7^ Cochrane review of blood management algorithms based on point-of-care tests for coagulopathy,^6^ and the 2015 NICE transfusion guideline review of studies evaluating the cost-effectiveness of PBM interventions.^4^ A full description of the searches, extraction, bias assessment, and synthesis, are listed in the Supplementary Appendix.

Treatment effects were estimated for the following type of variables: dichotomous variables – the number of events in the treatment and control groups were collected, and the risk ratio (RR) with (95% confidence intervals [CIs]) was calculated; continuous variables – the standardised mean differences (SMDs) with 95% CI were calculated. The pooled treatment effects were estimated with random-effects models using Review Manager (RevMan; version 5.3; The Nordic Cochrane Centre, The Cochrane Collaboration, 2014, Copenhagen).

Inconsistency within each meta-analysis was explored with Cochrane's *Q*^2^ test (with significance set at *P*=0.05) and *I*^2^ statistics.^13^ Expected sources of heterogeneity explored in sub-group and sensitivity analyses included type of intervention, clinical setting, participant characteristics, and risk of bias.

The network meta-analysis adopted a frequentist approach using the R package *netmeta* (R Foundation for Statistical Computing, Vienna, Austria) developed on the basis of graph-theoretical methods.^14^ This analysis considered the concomitant interventions specified in the included studies. A network diagram was produced for each outcome. Interventions that were not connected to the network were excluded from the analysis. To rank the interventions, we reported the *P* score, which measures the extent of certainty that an intervention is better than the competing interventions.^15^ Heterogeneity was evaluated using tau^2^, *I*^2^ statistics, and Cochran's *Q* test. To check for consistency, network estimates were split into the contribution of direct and indirect evidence, and their agreement was tested.

The pre-specified intention to model the cost-effectiveness of PBM interventions was abandoned because of the heterogeneity of the trial cohorts, interventions, follow-up, and paucity of available data for analysis. A narrative review of studies identified in these searches was performed and reported according to the Consolidated Health Economic Evaluation Reporting Standards (CHEERS) checklist.^16^

GRADEPRO GDT software (gdt.gradepro.org) was used to prepare the ‘Summary of findings’ table according to the Grades of Recommendation, Assessment, Development, and Evaluation (GRADE) approach.^17^

## Results

### Study selection

Preferred Reporting Items for Systematic Reviews and Meta-Analyses (PRISMA) diagrams describing the updated searches of the six reviews of PBM interventions are presented in Supplementary Figure 1. A detailed description of the searches is reported in the Supplementary Appendix. In summary, the following trials fulfilled the inclusion and exclusion criteria and provided quantitative data for the review: pre-surgery iron therapy, eight RCTs (*n*=1031 participants); perioperative cell salvage, 43 RCTs (*n*=6083 participants); restrictive transfusion thresholds, 23 RCTs (*n*=13 324 participants); tranexamic acid, 307 RCTs (*n*=33 572 participants); point-of-care test based algorithms, 13 RCTs (*n*=907 participants); health economic outcomes, 41 trials (*n*=15 003 participants) plus four published economic modelling studies.

### Characteristics of included studies

Details of included studies are listed in Supplementary Table 1. Of 393 RCTs enrolling a total of 54 917 participants included in the review, there were 120 cardiac surgery RCTs, 209 orthopaedic, six hepatobiliary, 10 urology, and 48 RCTs in other types of surgery. Patients were grouped as having cardiovascular disease in 148 trials, cancer in 14 trials, renal disease in 16 trials, anaemia in 40 trials, or elevated bleeding risk in 156 trials.

### Risk of bias within studies

The results of the risk of bias assessments for individual trials are shown in Supplementary Figure 2. The proportions of trials with low risk, unclear risk, and high risk of bias in each of the domains are shown in Supplementary Figure 3. From the 393 included trials, the following methodological limitations were identified:

*Sequence generation:* random sequence generation was adequate in 212 (53.9%) trials and unclear in 153 (38.9%) trials. There was a high risk of bias for random sequence generation in 28 (7.1%) trials.
*Allocation:* allocation concealment was adequate in 161 (40.9%) trials and unclear in 188 (47.8%) trials. There was a high risk of bias for random sequence generation in 44 (11.2%) trials.
*Blinding:* there was evidence of blinding of patients and clinical staff caring for patients after operation in 202 (51.4%) trials, and unclear evidence in 98 (24.9%) trials. There was a high risk of bias in 93 (23.6%) trials. There was evidence of blinding of outcome assessors in 214 (54.4%) trials and unclear evidence of blinding of outcome assessors in 122 (31%) trials. There was a high risk of bias in 57 (14.5%) trials.
*Incomplete outcome data:* a total of 110 (27.9%) trials that failed to report completeness of follow-up or adequate follow-up were considered to be at high risk of attrition bias.

Excluding trials considered at high risk of bias in any domain resulted in 254 trials enrolling 40 756 participants.

### Data synthesis

**Fig 1:**
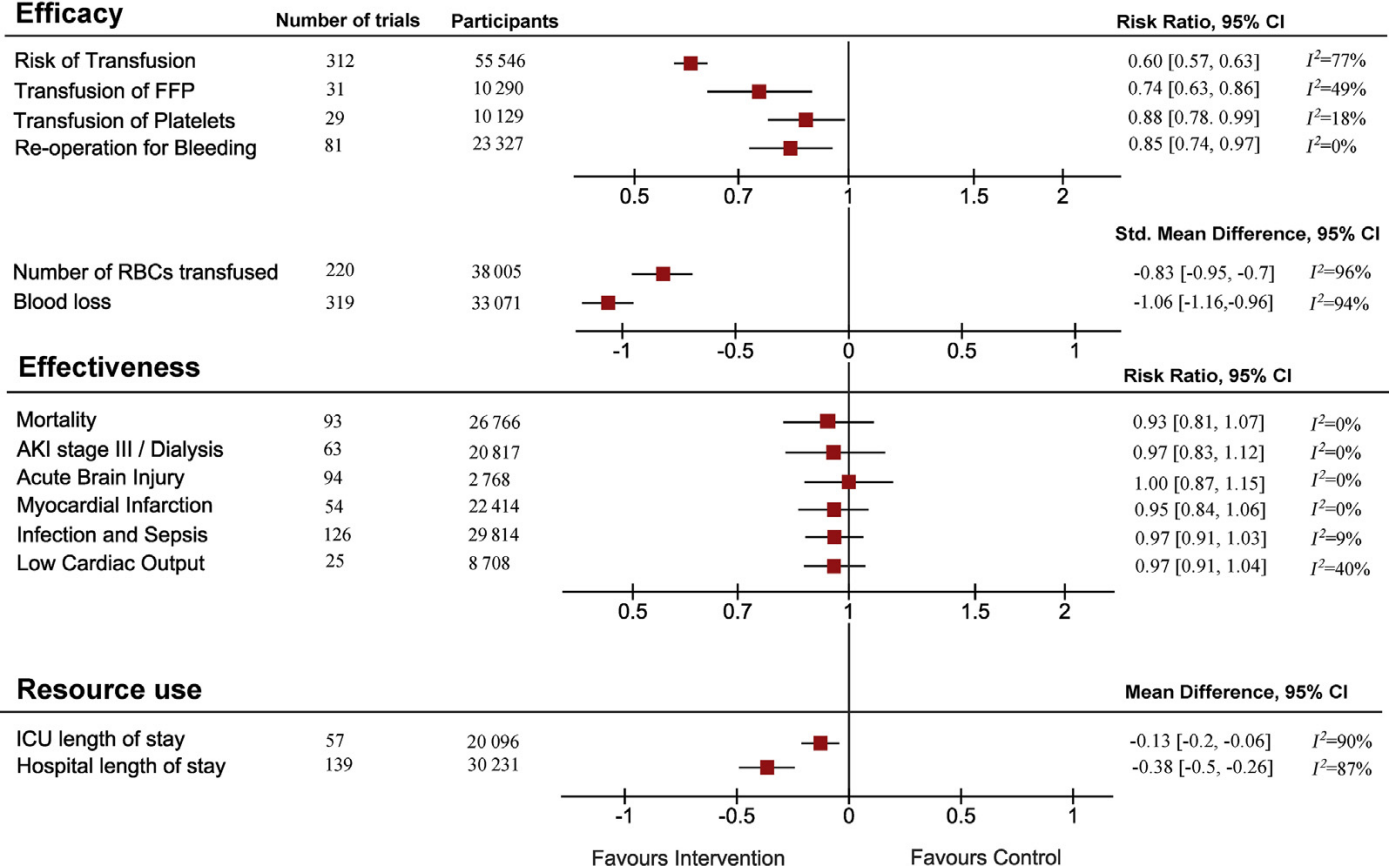
Forest plots of transfusion and bleeding, effectiveness and resource use outcomes. Interventions were compared with controls, showing a significant reduction of the effect on transfusion and bleeding outcomes, but no significant difference in the effectiveness outcomes. There was moderate heterogeneity for the risk of red blood cell transfusion (*I*^2^=77%) and no heterogeneity for mortality (*I*^2^=0%). The results are expressed as risk ratio (RR), mean difference (MD), or standard mean difference (SMD), along with 95% confidence intervals (CIs). The heterogeneity for each outcome is expressed as *I*^2^. AKI, acute kidney injury.

**Fig 2:**
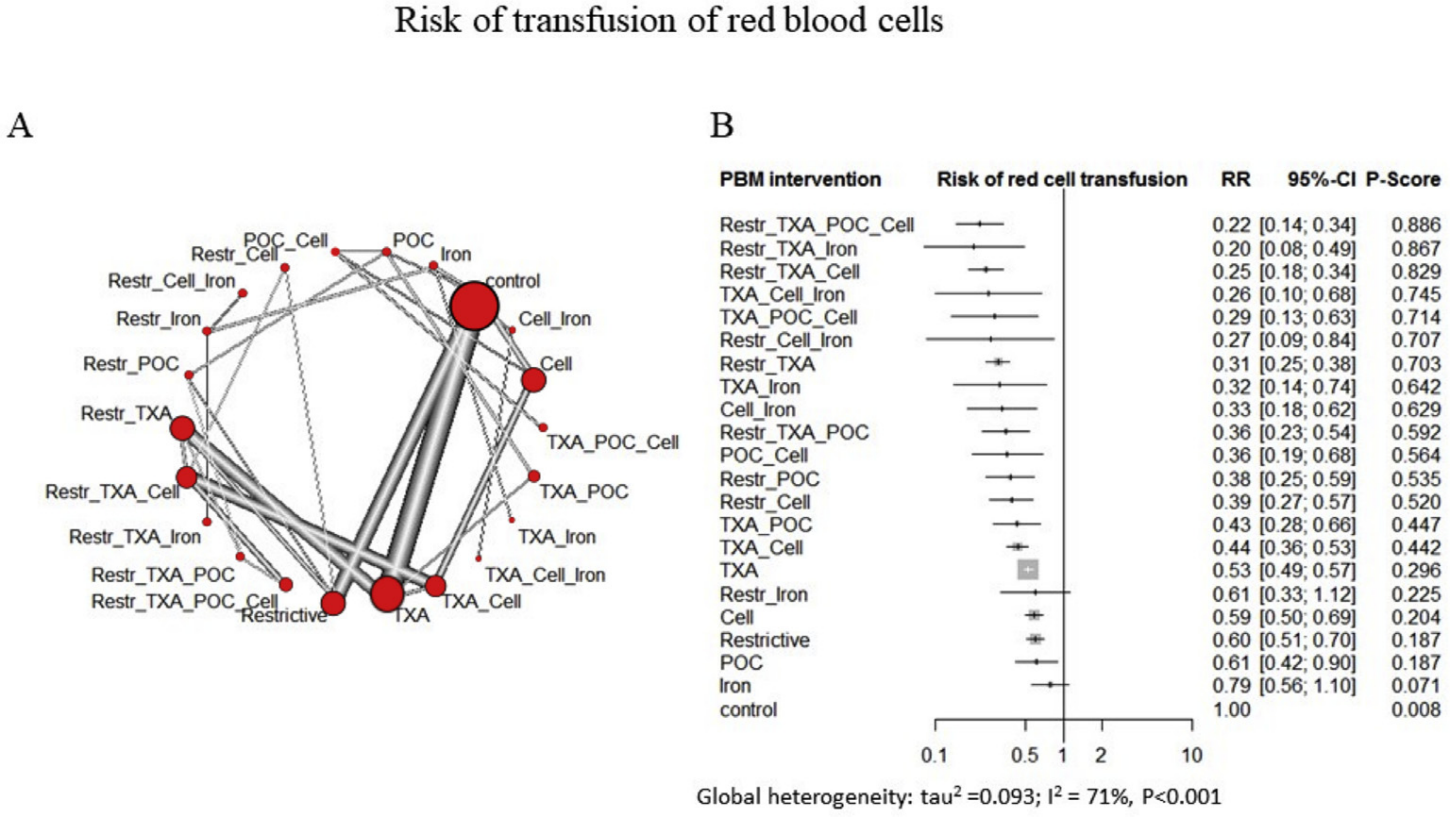
A network meta-analysis of eligible comparisons for the rate of red blood cells transfusions. (a) Width of the lines is proportional to the number of trials comparing every pair of treatments. (b) The network model comparing different combinations of interventions show that a combination of restrictive threshold (Restr), tranexamic acid (TXA), point of care (POC) testing and cell salvage ranked highest in reducing the rate of red blood cells transfusions (risk ratio [RR]=0.22; 95% confidence interval [CI] 0.14, 0.34; *P* score=0.88), followed by a combination of restrictive threshold, tranexamic acid, and iron treatment (RR=0.2; 95% CI 0.08, 0.49) when compared with the control group. There were no inconsistencies between direct and indirect comparisons. There is a moderate global heterogeneity (*I*^2^=71%, *P*<0.001). The effects are reported as *P* score, which is the probability of an intervention having a higher effect when compared with other treatments, with *P*=1 showing the maximum probability. The results are expressed as RR along with 95% CIs and *P* scores. The global heterogeneity is expressed as *I*^2^, with *P*<0.05 considered statistically significant.

**Fig 3:**
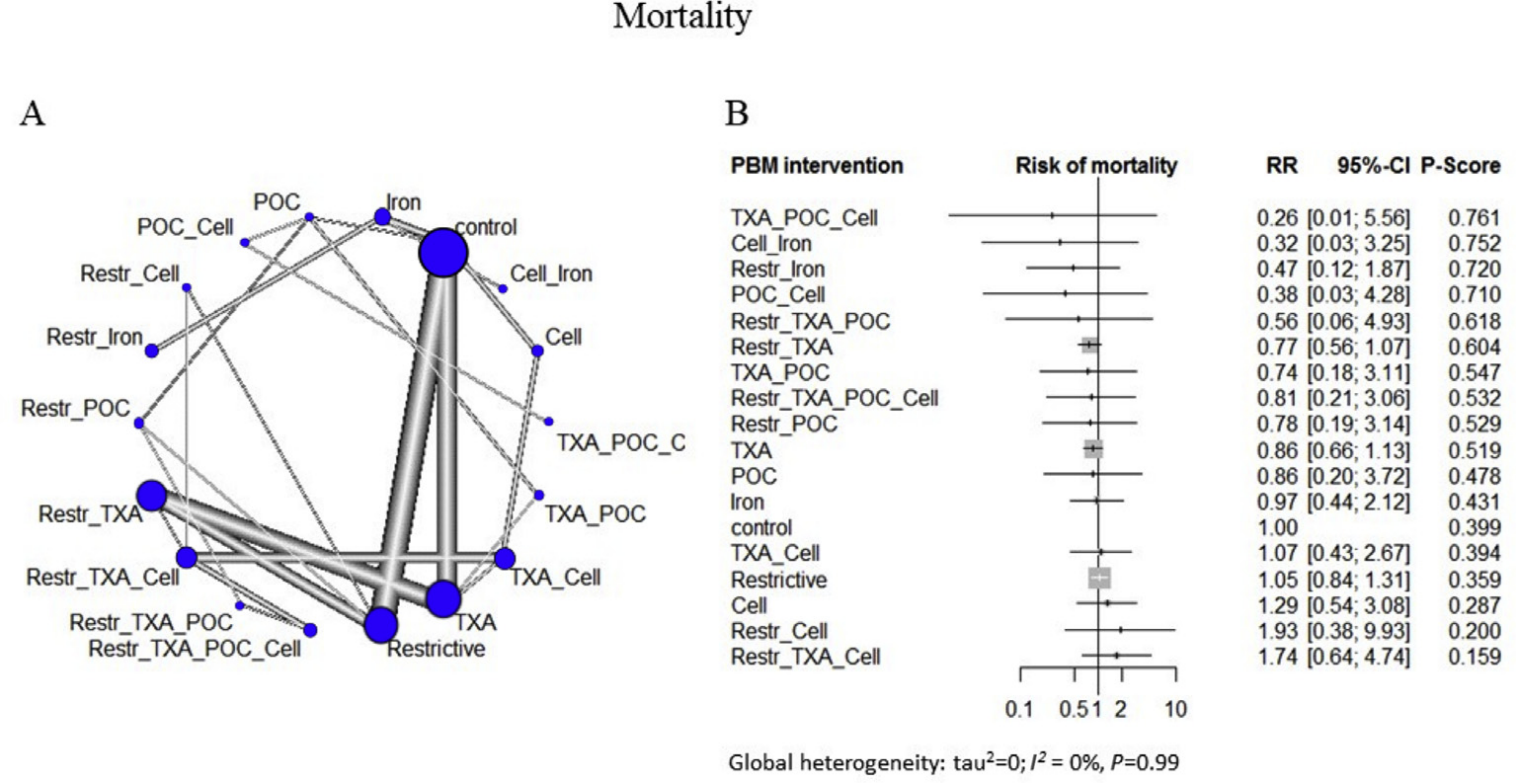
A network meta-analysis of eligible comparisons for mortality. (a) Width of the lines is proportional to the number of trials comparing every pair of treatments. (b) The network model comparing different combinations of interventions show that a combination of tranexamic acid (TXA), point of care (POC), and cell salvage ranked highest in reducing mortality (risk ratio [RR]=0.26; 95% confidence interval [CI] 0.01, 5.56; *P*=0.76), followed by a combination of cell salvage and iron treatment (RR=0.32; 95% CI 0.03, 3.25) when compared with the control group. There were no inconsistencies between direct and indirect comparisons. There is no significant global heterogeneity (*I*^2^=0%, *P*=0.99). The effects reported as *p*-score, which is the probability of an intervention having a higher effect when compared with other treatments, with *P*=1 showing the maximum probability. The results are expressed as RR along with 95% CIs and *P* scores. The global heterogeneity is expressed as *I*^2^, with *P*<0.05 considered statistically significant. Restr, restrictive threshold.

The main results of the data synthesis are reported in Fig 1, Fig 2, Fig 3.

### Effect on transfusion and bleeding

A meta-analysis of 312 trials with 55 546 participants (iron therapy: eight trials, *n*=1181; cell salvage: 42 trials, *n*=5895; restrictive thresholds: 22 trials, *n*=13 156; tranexamic acid: 231 trials, *n*=27 159; point-of-care: 10 trials, *n*=8155) demonstrated a significant reduction in exposure to red cell transfusion in participants allocated to PBM interventions *vs* controls (RR=0.60; 95% CI 0.57, 0.63; *I*^2^=77%).

There was a significant subgroup interaction for treatment effect with the type of intervention (χ^2^=53.49, *P*<0.001; Supplementary Fig. 4). The greatest reduction was observed with tranexamic acid (RR=0.54; 95% CI 0.50, 0.58; *I*^2^=75%), and the least with point-of-care tests (RR=0.84; 95% CI 0.76, 0.93; *I*^2^=43%; Supplementary Fig. 4).

The results of the meta-analyses of secondary transfusion and bleeding outcomes are reported in Figure 1 and Supplementary Figure 4. There was high heterogeneity for the effect of PBM on the volume of red cells transfused or risk of receiving non-red cell components that were not resolved by sub-group analyses. Tranexamic acid significantly reduced reoperation rates for bleeding (RR=0.63; 95% CI 0.5, 0.78; *I*^2^=0%; Supplementary Fig. 4).

Subgroup analyses indicated that the reduction in transfusion and bleeding outcomes was consistent across all pre-specified sources of heterogeneity (Supplementary Table 3). Funnel plots for transfusion and bleeding related outcomes demonstrated significant publication bias in favour of PBM interventions (Supplementary Fig. 5). Sensitivity analyses that excluded trials at high risk of bias (Supplementary Table 4), or included only trials where there was evidence of allocation concealment (Supplementary Table 5), demonstrated effect estimates similar to those of the primary analyses.

The network model indicated that no combination of PBM interventions had a significantly greater effect on bleeding and transfusion outcomes than any individual intervention (Fig. 2 and Supplementary Fig. 6).

### Clinical effectiveness

Meta-analyses of 93 trials with 26 766 participants (iron therapy: four trials, *n*=906; cell salvage: 16 trials, *n*=1710; restrictive thresholds: 19 trials, *n*=12 866; tranexamic acid: 47 trials, *n*=10 621; point-of-care: seven trials, *n*=663) demonstrated no significant difference in 30-day or hospital mortality between participants randomised to PBM interventions *vs* controls without heterogeneity (RR=0.93; 95% CI 0.81, 1.07; *I*^2^=0%; Supplementary Fig. 7).

There was no significant reduction in mortality for any individual PBM intervention, in any subgroup (Supplementary Tables 6–8). There was no reduction in secondary clinical outcomes including acute kidney injury stage 3, acute brain injury, myocardial infarction, sepsis and infection, and low cardiac output, with no or mild heterogeneity (Fig. 1). Funnel plots indicated no publication bias for effectiveness outcomes (Supplementary Fig. 8). The results of sensitivity analyses were similar to those of the primary analyses (Supplementary Tables 7 and 8).

For the network meta-analyses, no combination of PBM interventions showed significant reductions in mortality *vs* controls (Supplementary Fig. 9).

### Economic evaluation

#### Resource use

Pooled treatment effect estimates for PBM interventions showing a reduction in ICU length of stay (reported in 57 trials, *n*=20 096) and hospital length of stay (reported in 139 trials, *n*=30 231) were limited by high heterogeneity (Fig. 1 and Supplementary Fig. 10). Tranexamic acid was the primary driver for the direction of treatment effect favouring the intervention; however, there was residual high heterogeneity for these estimates (both *I*^2^=89%) and in the subgroup and sensitivity analyses (Supplementary Tables 10 and 11). Funnel plots indicated likely publication bias for hospital length of stay but not for intensive care length of stay (Supplementary Fig. 11). The network model indicated that no combinations of interventions were more effective at reducing resource use *vs* any single intervention (Supplementary Fig. 12).

#### Cost reporting

Forty-one studies reported costs from the secondary care perspective. Cost savings were reported in all intervention types except for studies on cell salvage. The cost savings ranged from £36 to £5264, whereas for cell salvage costs varied from savings of £5264 to excess costs of £22 126. Six of the cell salvage studies showed that the control groups were dominant (Supplementary Table 12).

#### Cost-effectiveness

Only one trial compared the cost-effectiveness of a PBM intervention to controls (Supplementary Table 13).^18^ This trial, in adult cardiac surgery, demonstrated that a restrictive red cell transfusion threshold reduced costs, mean restrictive minus liberal difference of −£182, (from −£1108 to £744), but had no effect on quality adjusted life years, 0.0004 (from −0.0037 to 0.0045). Four model-based studies that were informed in part by RCT evidence had conflicting results, largely attributable to the different assumptions used across the studies (Supplementary Table 14).

### GRADE summary of evidence

PBM interventions reduce transfusion and hospital length of stay but not mortality, acute kidney injury, acute brain injury, acute myocardial infarction, or sepsis (Supplementary Table 15). Levels of certainty were downgraded to low or very low for all clinical effectiveness outcomes because of the small numbers of patients in many of the trials and a large number of studies at increased risk of bias.

## Discussion

A systematic review and meta-analysis of existing RCTs demonstrated that five common PBM interventions, either alone or in combination, reduced bleeding and transfusion in people undergoing major surgery. Treatment estimates for these outcomes were not influenced by the trial quality, although there was a significant publication bias in favour of PBM. Heterogeneity of treatment effect on bleeding and transfusion outcomes were attributable to pre-specified factors including the risk of bleeding, type of surgery, type of intervention, and co-morbid conditions. PBM interventions did not demonstrate clinical effectiveness, without heterogeneity of treatment effects across treatments and settings. These results were not attributable to bias and were consistent for all secondary outcomes and sensitivity analyses. Treatment effect estimates for resource use, despite showing a reduction in ICU and hospital length of stay, demonstrated high heterogeneity across primary, sub-group, and sensitivity analyses, limiting interpretation of these data. Where costs were reported, 32 of 38 trials concluded that PBM interventions reduced costs, and six of 38 suggested that PBM interventions increased costs. No trial demonstrated that a PBM intervention was cost-effective. Network meta-analyses did not demonstrate significant additional increases in the effect on bleeding and transfusion, effectiveness, or reductions in resource use when more than one PBM intervention was administered.

The most important finding of this review is that the PBM interventions included in this analysis, which reduced red cell transfusion by one-third, had no effect on important clinical outcomes, even in trial cohorts at medium or high risk of bleeding. This is strong evidence that there is no causal relationship between red cell transfusion and adverse clinical outcomes. Another key finding is that interventions that reduce the severity of anaemia in surgery patients have no important clinical benefits. This argues against a causal relationship between anaemia and adverse outcomes in surgery. Anaemia may simply be a biomarker of the severity of chronic disease.^19^ Thirdly, the use of multiple interventions did not increase further the effect on transfusion and bleeding or translate into effectiveness. This is at odds with the multifaceted PBM approach. A qualification of this observation is that network meta-analyses may not reproduce the personalised application of PBM interventions in practice.^20^

From the blood services perspective, the benefits of PBM are clear.^3^^,^^21^ Donated blood components are a precious commodity.^22^ Reducing transfusion requirements reduces pressures on transfusion services and lowers direct transfusion costs to users, which may benefit policymakers and hospital expenditures. This perspective is reflected in recent international guidelines, in which, for example, pre-surgery iron therapy is recommended based on evidence of a reduced effect on transfusion and bleeding and in the absence of evidence of effectiveness.^3^^,^^23^^,^^24^ The routine use of PBM may also be indicated in countries where there are acute blood shortages^25^ or inadequate proactive pathogen reduction strategies in donated blood, or in selected populations that mandate transfusion avoidance strategies, e.g. on religious grounds).^22^ However, in other clinical settings, the certainty of the evidence is insufficient to recommend routine use. This manuscript is a direct rebuttal of recent international treatment recommendations published recently.^3^ We feel that these guidelines produced an unbalanced interpretation of the benefits to society from PBM, primarily in terms of reduction in blood use, *vs* the limited benefits to patients. Crucially these findings are at odds with the original WHO definition of PBM.^26^

PBM interventions may reduce secondary care costs, but this has not been shown to equate to cost-effectiveness. This is explained by two observations. First, the reductions in transfusion costs with PBM interventions are small; the mean reduction in red cell transfusion in this review was 0.83 units per patient. In the UK, a single unit of red cells costs £190. In contrast, administration of intravenous iron costs approximately £79.7 per patient, consumables for point-of-care tests range from £43 to £79 per patient, and cell salvage consumables cost approximately £300.^27^^,^^28^ Second, no analysis demonstrated clinical effectiveness.

The results of this study suggest that the adoption of restrictive transfusion thresholds in surgery patients is reasonable as this has a direct and significant cost reduction in the absence of harm.^29^ Tranexamic acid also undoubtedly has a role in clinical care. The reductions in mortality in this analysis were not statistically significant. The point treatment effect estimate (RR=0.83) was also less than that observed in a previous review^7^ (RR=0.61), but included an additional 220 trials of tranexamic acid in 26 399 participants. However, it is inexpensive, costing £7–15 per patient, and reduces reoperation for bleeding, and perhaps resource use. In settings where coagulative haemorrhage is common such as liver, cardiac, and transplant surgery, these are undoubted benefits.^21^ A research question identified by this study is whether there is any added benefit from the use of PBM interventions beyond restrictive transfusion thresholds and tranexamic acid use.

A potential limitation of the analyses is the pooling of studies undertaken in different cohorts and using different interventions. However, the contrast in the heterogeneity of effects across treatments and settings for bleeding and transfusion outcomes but not for effectiveness outcomes supports this approach and provides a unique perspective on the evidence for PBM. It also enables comprehensive network meta-analyses, the results of which support the findings of the primary analyses. Inconsistency of outcome definitions and reporting is also likely given the multiple clinical settings; however, our two primary outcomes, red cell transfusion and mortality, are resilient to detection bias. Many of the trials included in the analyses recruited small sample sizes and low-risk cohorts. This may have diminished the ability of the review to detect treatment effects on clinical outcomes. However, the consistency of the findings of the clinical effectiveness analyses across subgroups, including those stratified by anaemia and bleeding risk, sensitivity analyses and secondary outcomes in large sample size, and without heterogeneity, argues against this as an important confounder. Finally, other interventions that reduce anaemia such as the use of erythropoietin were not included in our study. Given the absence of evidence of clinical effectiveness for erythropoietin in multiple previous systematic reviews, we consider this unlikely to have influenced the analyses.^3^^,^^4^

In conclusion, a systematic review of the existing evidence suggests that PBM interventions do not have important clinical benefits beyond reducing bleeding and transfusion in people undergoing major surgery. Treatment effects were consistent across multiple types of surgery, strata of bleeding or transfusion risk, and comorbidity. These conclusions are qualified by the Low or Very Low confidence in the precision of the treatment estimates in the GRADE summaries. Further high-quality trials are required to address remaining uncertainty, and to define the treatment indications for PBM interventions before these are recommended for routine use.

## Authors' contributions

Study concept and design: MR, RA, SQ, FYL, TK, TR, GY, GJM

Data acquisition/analysis/interpretation: MR, RA, SP, SO, SY, FYL, TR, GY, LE, GJM

Writing of first draft: MR, RA, SP, SO, MW, SQ, FYL, TK, TR, GY, LE, GJM

Revision of manuscript for important intellectual content: MR, RA, SP, SQ, MW, FYL, TK, TR, GY, LE, GJM

All authors agree to be accountable for all aspects of the work thereby ensuring that questions related to the accuracy or integrity of any part of the work are appropriately investigated and resolved. All authors approved the final version of the manuscript.

## Declarations of interest

MR is a NIHR Clinical Lecturer. GJM reports support for educational activities from Terumo, outside the submitted work. TR reports grants from the UK, NIHR HTA; Australian, NHMRC; NIAA/BJA/ACTA/VASGBI; and NIHR EME; grants, personal fees, and non-financial support from Pharmocosmos and Vifor Pharma; and grants and personal fees from Acelity, Amgen, Medtronic, and Tiash Ltd, outside the submitted work. TR is a director of The Iron Clinic Ltd and Veincare London Ltd, and is the Vascular lead for 18 week wait. The other authors declare that they have no conflicts of interest.

## Funding

10.13039/501100000274British Heart Foundation (RG/13/6/29947, CH/12/1/29419 to GJM, MW, TK, MR). 10.13039/100015250Leicester NIHR Biomedical Research Centre (to MR, RGA and SP). NIHR Health Technology Assessment (to TR and 13/34/64 to GY). 10.13039/100009033NHS Blood and Transplant Research & Development Funding (to LJE). UK-China AMR Partnership Hub under Newton Fund (MR/S013717/1 to GY). 10.13039/100012630Zimmer Biomet (to GJM, TK, and MW).

## References

[1] Shander A., Van Aken H., Colomina M.J. (2012). Patient blood management in Europe. Br J Anaesth.

[2] Meybohm P., Richards T., Isbister J. (2017). Patient blood management bundles to facilitate implementation. Transfus Med Rev.

[3] Mueller M.M., Van Remoortel H., Meybohm P. (2019). Patient blood management: recommendations from the 2018 Frankfurt consensus conference. JAMA.

[4] Padhi S., Kemmis-Betty S., Rajesh S., Hill J., Murphy M.F. (2015). Blood transfusion: summary of NICE guidance. BMJ.

[5] Carson J.L., Stanworth S.J., Roubinian N. (2016). Transfusion thresholds and other strategies for guiding allogeneic red blood cell transfusion. Cochrane Database Syst Rev.

[6] Wikkelso A., Wetterslev J., Moller A.M., Afshari A. (2016). Thromboelastography (TEG) or thromboelastometry (ROTEM) to monitor haemostatic treatment versus usual care in adults or children with bleeding. Cochrane Database Syst Rev.

[7] Ker K., Edwards P., Perel P., Shakur H., Roberts I. (2012). Effect of tranexamic acid on surgical bleeding: systematic review and cumulative meta-analysis. BMJ (Online).

[8] Meybohm P., Choorapoikayil S., Wessels A., Herrmann E., Zacharowski K., Spahn D.R. (2016). Washed cell salvage in surgical patients: a review and meta-analysis of prospective randomized trials under PRISMA. Medicine (Baltimore).

[9] Clevenger B., Gurusamy K., Klein A.A., Murphy G.J., Anker S.D., Richards T. (2016). Systematic review and meta-analysis of iron therapy in anaemic adults without chronic kidney disease: updated and abridged Cochrane review. Eur J Heart Fail.

[10] Higgins J., Green S.E. (2011). Cochrane Handbook for systematic reviews of interventions. http://www.handbook.cochrane.org.

[11] Gurusamy K.S., Nagendran M., Broadhurst J.F., Anker S.D., Richards T. (2014). Iron therapy in anaemic adults without chronic kidney disease. Cochrane Database Syst Rev.

[12] Carless P.A., Henry D.A., Moxey A.J., O'Connell D., Brown T., Fergusson D.A. (2010). Cell salvage for minimising perioperative allogeneic blood transfusion. Cochrane Database Syst Rev.

[13] Higgins J.P., Thompson S.G. (2002). Quantifying heterogeneity in a meta-analysis. Stat Med.

[14] Rucker G. (2012). Network meta-analysis, electrical networks and graph theory. Res Synth Methods.

[15] Rücker G., Schwarzer G. (2015). Ranking treatments in frequentist network meta-analysis works without resampling methods. BMC Med Res Methodol.

[16] Schünemann H., Oxman A., Vist G., Higgins J.P.T., Green S. (2011). Interpreting results and drawing conclusions. Cochrane Handbook for systematic reviews of interventions version 5.1.0 [updated March 2011].

[17] Guyatt G.H., Oxman A.D., Vist G.E. (2008). GRADE: an emerging consensus on rating quality of evidence and strength of recommendations. BMJ.

[18] Reeves B.C., Pike K., Rogers C.A. (2016). A multicentre randomised controlled trial of Transfusion Indication Threshold Reduction on transfusion rates, morbidity and health-care resource use following cardiac surgery (TITRe2). Health Technol Assess.

[19] Jankowska E.A., Malyszko J., Ardehali H. (2013). Iron status in patients with chronic heart failure. Eur Heart J.

[20] Nelson M., Green J., Spiess B. (2018). Measurement of blood loss in cardiac surgery: still too much. Ann Thorac Surg.

[21] Alshryda S., Mason J., Hungin A.P.S. (2013). Topical (intra-articular) tranexamic acid reduces blood loss and transfusion rates following total hip replacement: a randomized controlled trial (TRANX-H). J Bone Jt Surg Am.

[22] Dean C.L., Wade J., Roback J.D. (2018). Transfusion-transmitted infections: an update on product screening, diagnostic techniques, and the path ahead. J Clin Microbiol.

[23] (2012). Patient blood management guidelines: module 2 — perioperative.

[24] (2014). Patient blood management recommendations.

[25] Vaglio S., Gentili S., Marano G. (2017). The Italian regulatory guidelines for the implementation of patient blood management. Blood Transfus.

[26] World Health Organisation (2011). Global forum for blood safety: patient blood management. https://www.who.int/bloodsafety/events/gfbs_01_pbm/en/.

[27] Whiting P., Al M., Westwood M. (2015). Viscoelastic point-of-care testing to assist with the diagnosis, management and monitoring of haemostasis: a systematic review and cost-effectiveness analysis. Health Technol Assess.

[28] National Institute for Care Excellence (2015). Costing statement: blood transfusion implementing the NICE guideline on blood transfusion (NG24). https://www.nice.org.uk/guidance/ng24/resources/costing-statement-pdf-2177158141.

[29] Stokes E.A., Wordsworth S., Bargo D. (2016). Are lower levels of red blood cell transfusion more cost-effective than liberal levels after cardiac surgery? Findings from the TITRe2 randomised controlled trial. BMJ Open.

