# Supplementary material for: Patient blood management interventions do not lead to important clinical benefits or cost-effectiveness for major surgery: a network meta-analysis
Source: Br J Anaesth. 2020 Jun 30;126(1):149–56. doi: 10.1016/j.bja.2020.04.087 (PMC7844348; doi:10.1016/j.bja.2020.04.087)
Supplement: Multimedia component 1 [file mmc1.docx]

**Patient Blood Management interventions do not demonstrate important clinical benefits or cost effectiveness in people undergoing surgery: a network meta-analysis**

Marius Roman, Riccardo Abbasciano, Suraj Pathak, Shwee Oo, Syabira Yussoff, Marcin Wozniak, Saqib Qureshi, Florence Lai, Tracy Kumar, Guiqing Yao, Lise Estcourt, and Gavin James Murphy.

**Supplementary Appendix**

**Contents**

[1 PRISMA NMA checklist 8](#_Toc26829711)

[2 Search strategy 10](#_Toc26829712)

[2.1 Search Strategy Restrictive vs. Liberal Transfusion 10](#_Toc26829713)

[2.2 Search Strategy Tranexamic Acid 11](#_Toc26829714)

[2.3 Search Strategy Iron Therapy 11](#_Toc26829715)

[2.4 Search Strategy Point of Care testing 12](#_Toc26829716)

[2.5 Search Strategy Cell Salvage 12](#_Toc26829717)

[2.6 Search Strategy for Cost Effectiveness 13](#_Toc26829718)

[3 Detailed methods and criteria for considering studies for this review 13](#_Toc26829719)

[3.1 Searching other resources 13](#_Toc26829720)

[3.2 Selection of studies 13](#_Toc26829721)

[3.3 Data extraction and management 13](#_Toc26829722)

[3.4 Assessment of risk of bias in included studies 14](#_Toc26829723)

[3.5 Measures of treatment effect 14](#_Toc26829724)

[3.6 Likely sources of heterogeneity 14](#_Toc26829725)

[3.7 Assessment of heterogeneity 14](#_Toc26829726)

[3.8 Investigation of heterogeneity 14](#_Toc26829727)

[3.9 Assessment of reporting biases 14](#_Toc26829728)

[3.10 Unit of analysis issues 14](#_Toc26829729)

[3.11 Dealing with missing data 15](#_Toc26829730)

[3.12 Summary of findings 15](#_Toc26829731)

[3.13 Network meta-analysis (NMA) methodology 15](#_Toc26829732)

[4 PRISMA flow diagrams (eFigure 1. A-G) 16](#_Toc26829733)

[A 16](#_Toc26829734)

[5 Table of reasons for excluded studies following assessment of full texts (eTable 1.) 23](#_Toc26829735)

[6 Characteristics of included studies (eTable 2.) 26](#_Toc26829736)

[6.1 Controls: 27](#_Toc26829737)

[6.2 Co-interventions: 27](#_Toc26829738)

[7 Summary of Risk of bias table (eFigure 2) 135](#_Toc26829739)

[7.1 Iron treatment 136](#_Toc26829740)

[7.2 Cell Salvage 137](#_Toc26829741)

[7.3 Restrictive vs. Liberal 138](#_Toc26829742)

[7.4 Tranexamic Acid 139](#_Toc26829743)

[7.5 Point of Care testing 140](#_Toc26829744)

[8 Risk of bias table of included studies (eFigure 3) 141](#_Toc26829745)

[8.1 Iron treatment 141](#_Toc26829746)

[8.2 Cell Salvage 142](#_Toc26829747)

[8.3 Restrictive vs. Liberal 145](#_Toc26829748)

[8.4 Tranexamic Acid 147](#_Toc26829749)

[8.5 Point of Care 163](#_Toc26829750)

[9 Forest plots for transfusion and bleeding outcomes 164](#_Toc26829751)

[9.1 Risk of Transfusion (eFigure4a) 164](#_Toc26829752)

[9.2 Risk of receiving red cell transfusion GRADE assessment (eTable 15a) 165](#_Toc26829753)

[9.3 Number of red cells transfused (eFigure4b) 168](#_Toc26829754)

[9.4 Number of red cells transfused GRADE assessment (eTable 15b) 169](#_Toc26829755)

[9.5 Risk of FFP transfusion (eFigure4c) 172](#_Toc26829756)

[9.6 Risk of FFP transfusion GRADE assessment (eTable 15c) 173](#_Toc26829757)

[9.7 Risk of Platelets transfusion (eFigure4d) 176](#_Toc26829758)

[9.8 Risk of Platelets transfusion GRADE assessment (eTable 15d) 177](#_Toc26829759)

[9.9 Perioperative blood loss (eFigure4e) 181](#_Toc26829760)

[9.10 Perioperative blood loss GRADE assessment (eTable 15e) 182](#_Toc26829761)

[9.11 Re-operation for bleeding (eFigure4f) 185](#_Toc26829762)

[9.12 Re-operation for bleeding GRADE assessment (eTable 15f) 186](#_Toc26829763)

[10 Funnel plots for transfusion and bleeding outcomes (eFigure 5) 190](#_Toc26829764)

[11 Subgroup analyses for transfusion and bleeding outcomes (eTable 3) 195](#_Toc26829765)

[12 Sensitivity analysis for transfusion and bleeding outcomes based on exclusion of studies at high risk of bias (eTable 4) 201](#_Toc26829766)

[13 Sensitivity analysis for transfusion and bleeding outcomes stratified by risk of concealment bias (eTable 5) 204](#_Toc26829767)

[14 Network meta-analyses of transfusion and bleeding outcomes 206](#_Toc26829768)

[14.1 Risk of receiving red cell transfusion (eFigure 6a) 206](#_Toc26829769)

[14.2 Summary of study limitations of the included studies 207](#_Toc26829770)

[14.3 Evaluation of Inconsistency (Split direct and indirect evidence to test for local consistency) 208](#_Toc26829772)

[14.4 Number of red cells transfused (eFigure 6b) 209](#_Toc26829773)

[14.5 Summary of study limitations of the included studies 210](#_Toc26829774)

[14.6 Evaluation of Inconsistency (Split direct and indirect evidence to test for local consistency) 211](#_Toc26829777)

[14.7 Risk of receiving fresh frozen plasma (FFP) (eFigure 6c) 212](#_Toc26829778)

[14.8 Summary of study limitations of the included studies 213](#_Toc26829779)

[14.9 Evaluation of Inconsistency (Split direct and indirect evidence to test for local consistency) 214](#_Toc26829781)

[14.10 Risk of receiving Platelets (eFigure 6d) 215](#_Toc26829782)

[14.11 Summary of study limitations of the included studies 216](#_Toc26829783)

[14.12 Evaluation of Inconsistency (Split direct and indirect evidence to test for local consistency) 217](#_Toc26829785)

[14.13 Perioperative blood loss (eFigure 6e) 218](#_Toc26829786)

[14.14 Summary of study limitations of the included studies 219](#_Toc26829787)

[14.15 Evaluation of Inconsistency (Split direct and indirect evidence to test for local consistency) 220](#_Toc26829789)

[14.16 Re-operation for bleeding (eFigure 6f) 221](#_Toc26829790)

[14.17 Summary of study limitations of the included studies 222](#_Toc26829791)

[14.18 Evaluation of Inconsistency (Split direct and indirect evidence to test for local consistency) 223](#_Toc26829793)

[15 Forest plots for Effectiveness outcomes 224](#_Toc26829794)

[15.1 30-day mortality (eFigure 7a) 224](#_Toc26829795)

[15.2 30-day mortality GRADE assessment (eTable 16a) 225](#_Toc26829796)

[15.3 AKI Stage 3 or requiring dialysis (eFigure 7b) 228](#_Toc26829797)

[15.4 AKI Stage 3 or requiring dialysis GRADE assessment (eTable 16b) 229](#_Toc26829798)

[15.5 Acute brain injury (eFigure 7c) 233](#_Toc26829799)

[15.6 Acute brain injury GRADE assessment (eTable 16c) 234](#_Toc26829800)

[15.7 Myocardial Infarction (eFigure 7d) 237](#_Toc26829801)

[15.8 Myocardial Infarction GRADE assessment (eTable 16d) 238](#_Toc26829802)

[15.9 Sepsis and Infection (eFigure 7e) 241](#_Toc26829803)

[15.10 Sepsis and Infection GRADE assessment (eTable 16e) 242](#_Toc26829804)

[15.11 Low Cardiac Output (eFigure 7f) 245](#_Toc26829805)

[15.12 Low Cardiac Output GRADE assessment (eTable 16f) 246](#_Toc26829806)

[16 Funnel plots for Effectiveness outcomes (eFigure 8) 249](#_Toc26829807)

[17 Subgroup analysis for Effectiveness outcomes (eTable 6) 255](#_Toc26829808)

[18 Sensitivity analysis for Effectiveness outcomes excluding all trials considered at high risk of bias (eTable 7) 261](#_Toc26829809)

[19 Sensitivity analysis for Effectiveness outcomes stratified by risk of concealment bias (eTable 8) 264](#_Toc26829810)

[20 Network meta-analyses of Effectiveness outcomes (eFigure 9a) 266](#_Toc26829811)

[20.1 30-day mortality 266](#_Toc26829812)

[20.2 Summary of study limitations of the included studies 267](#_Toc26829813)

[20.3 Evaluation of Inconsistency (Split direct and indirect evidence to test for local consistency) 268](#_Toc26829815)

[20.4 AKI stage III / Dialysis (eFigure 9b) 269](#_Toc26829816)

[20.5 Summary of study limitations of the included studies 270](#_Toc26829817)

[20.6 Evaluation of Inconsistency (Split direct and indirect evidence to test for local consistency) 271](#_Toc26829820)

[20.7 Acute brain injury / stroke / TIA (eFigure 9c) 272](#_Toc26829821)

[20.8 Summary of study limitations of the included studies 273](#_Toc26829822)

[20.9 Evaluation of Inconsistency (Split direct and indirect evidence to test for local consistency) 274](#_Toc26829824)

[20.10 Myocardial infarction (eFigure 9d) 275](#_Toc26829825)

[20.11 Summary of study limitations of the included studies 276](#_Toc26829826)

[20.12 Evaluation of Inconsistency (Split direct and indirect evidence to test for local consistency) 277](#_Toc26829828)

[20.13 Sepsis and Infection (eFigure 9e) 278](#_Toc26829829)

[20.14 Summary of study limitations of the included studies 279](#_Toc26829830)

[20.15 Evaluation of Inconsistency (Split direct and indirect evidence to test for local consistency) 280](#_Toc26829832)

[20.16 Low cardiac output (eFigure 9f) 281](#_Toc26829833)

[20.17 Summary of study limitations of the included studies 282](#_Toc26829834)

[20.18 Evaluation of Inconsistency (Split direct and indirect evidence to test for local consistency) 283](#_Toc26829836)

[21 Forest plots for Resource use outcomes 284](#_Toc26829837)

[21.1 Intensive Care Unit (ICU) Length of Stay (eFigure 10a) 284](#_Toc26829838)

[21.2 Intensive Care Unit (ICU) Length of Stay GRADE assessment (eTable 17a) 285](#_Toc26829839)

[21.3 Hospital LOS (eFigure 10b) 288](#_Toc26829840)

[21.4 Hospital LOS GRADE assessment (eTable 17b) 289](#_Toc26829841)

[22 Funnel plots for Resource use outcomes (eFigure 11) 292](#_Toc26829842)

[23 Subgroup Analysis for Resource use outcomes (eTable 9) 294](#_Toc26829843)

[24 Sensitivity analysis for Resource use outcomes based on overall low risk of bias (eTable 10) 296](#_Toc26829844)

[25 Sensitivity analysis for Resource use outcomes stratified by risk of concealment bias (eTable 11) 297](#_Toc26829845)

[26 Network meta-analyses of Resource use outcomes (eFigure 12a) 298](#_Toc26829846)

[26.1 ICU length of stay 298](#_Toc26829847)

[26.2 Summary of study limitations of the included studies 299](#_Toc26829848)

[26.3 Evaluation of Inconsistency (Split direct and indirect evidence to test for local consistency) 300](#_Toc26829850)

[26.4 Hospital length of stay (eFigure 12b) 301](#_Toc26829851)

[26.5 Summary of study limitations of the included studies 302](#_Toc26829852)

[26.6 Evaluation of Inconsistency (Split direct and indirect evidence to test for local consistency) 303](#_Toc26829854)

[27 Cost descriptive table reported according to the CHEERS criteria (eTable 12) 304](#_Toc26829855)

[28 The CHEERS checklist for 36 studies reporting economic and cost evaluations (eTable 13) 309](#_Toc26829856)

[29 Cost modelling (eTable 14) 315](#_Toc26829857)

[30 Acknowledgements 317](#_Toc26829858)

[31 References 317](#_Toc26829859)

# PRISMA NMA checklist

**eTable 1**. PRISMA NMA Checklist of Items to Include When Reporting A Systematic Review Involving a Network Meta-analysis

| **Section/Topic** | **Item #** | **Checklist Item** | **Reported on Page #** |
| --- | --- | --- | --- |
| **TITLE** |  |  |  |
| Title | 1 | Identify the report as a systematic review *incorporating a network meta-analysis (or related form of meta-analysis).* | **1** |
| **ABSTRACT** |  |  |  |
| Structured summary | 2 | Provide a structured summary including, as applicable:  **Background:** main objectives  **Methods:** data sources; study eligibility criteria, participants, and interventions; study appraisal; and *synthesis methods, such as network meta-analysis.*  **Results:** number of studies and participants identified; summary estimates with corresponding confidence/credible intervals; *treatment rankings may also be discussed. Authors may choose to summarize pairwise comparisons against a chosen treatment included in their analyses for brevity.*  **Discussion/Conclusions:** limitations; conclusions and implications of findings.  **Other:** primary source of funding; systematic review registration number with registry name. | **N/A** |
| **INTRODUCTION** |  |  |  |
| Rationale | 3 | Describe the rationale for the review in the context of what is already known*, including mention of why a network meta-analysis has been conducted.* | **3** |
| Objectives | 4 | Provide an explicit statement of questions being addressed, with reference to participants, interventions, comparisons, outcomes, and study design (PICOS). | **3-4** |
| **METHODS** |  |  |  |
| Protocol and registration | 5 | Indicate whether a review protocol exists and if and where it can be accessed (e.g., Web address); and, if available, provide registration information, including registration number. | **3** |
| Eligibility criteria | 6 | Specify study characteristics (e.g., PICOS, length of follow-up) and report characteristics (e.g., years considered, language, publication status) used as criteria for eligibility, giving rationale. *Clearly describe eligible treatments included in the treatment network, and note whether any have been clustered or merged into the same node (with justification).* | **3-4** |
| Information sources | 7 | Describe all information sources (e.g., databases with dates of coverage, contact with study authors to identify additional studies) in the search and date last searched. | **4** |
| Search | 8 | Present full electronic search strategy for at least one database, including any limits used, such that it could be repeated. | **Supplement** |
| Study selection | 9 | State the process for selecting studies (i.e., screening, eligibility, included in systematic review, and, if applicable, included in the meta-analysis). | **Supplement** |
| Data collection process | 10 | Describe method of data extraction from reports (e.g., piloted forms, independently, in duplicate) and any processes for obtaining and confirming data from investigators. | **4** |
| Data items | 11 | List and define all variables for which data were sought (e.g., PICOS, funding sources) and any assumptions and simplifications made. | **4** |
| **Geometry of the network** | **S1** | Describe methods used to explore the geometry of the treatment network under study and potential biases related to it. This should include how the evidence base has been graphically summarized for presentation, and what characteristics were compiled and used to describe the evidence base to readers. | **Supplement** |
| Risk of bias within individual studies | 12 | Describe methods used for assessing risk of bias of individual studies (including specification of whether this was done at the study or outcome level), and how this information is to be used in any data synthesis. | **Supplement** |
| Summary measures | 13 | State the principal summary measures (e.g., risk ratio, difference in means). *Also describe the use of additional summary measures assessed, such as treatment rankings and surface under the cumulative ranking curve (SUCRA) values, as well as modified approaches used to present summary findings from meta-analyses.* | **4-5** |
| Planned methods of analysis | 14 | Describe the methods of handling data and combining results of studies for each network meta-analysis. This should include, but not be limited to:   - *Handling of multi-arm trials;* - *Selection of variance structure;* - *Selection of prior distributions in Bayesian analyses; and* - *Assessment of model fit.* | **4** |
| **Assessment of Inconsistency** | **S2** | Describe the statistical methods used to evaluate the agreement of direct and indirect evidence in the treatment network(s) studied. Describe efforts taken to address its presence when found. | **5** |
| Risk of bias across studies | 15 | Specify any assessment of risk of bias that may affect the cumulative evidence (e.g., publication bias, selective reporting within studies). | **Supplement** |
| Additional analyses | 16 | Describe methods of additional analyses if done, indicating which were pre-specified. This may include, but not be limited to, the following:   - Sensitivity or subgroup analyses; - Meta-regression analyses; - *Alternative formulations of the treatment network; and* - *Use of alternative prior distributions for Bayesian analyses (if applicable).* |  |
| **RESULTS** |  |  |  |
| Study selection | 17 | Give numbers of studies screened, assessed for eligibility, and included in the review, with reasons for exclusions at each stage, ideally with a flow diagram. | **Supplement** |
| **Presentation of network structure** | **S3** | Provide a network graph of the included studies to enable visualization of the geometry of the treatment network. | **Supplement** |
| **Summary of network geometry** | **S4** | Provide a brief overview of characteristics of the treatment network. This may include commentary on the abundance of trials and randomized patients for the different interventions and pairwise comparisons in the network, gaps of evidence in the treatment network, and potential biases reflected by the network structure. | **Supplement** |
| Study characteristics | 18 | For each study, present characteristics for which data were extracted (e.g., study size, PICOS, follow-up period) and provide the citations. | **Supplement** |
| Risk of bias within studies | 19 | Present data on risk of bias of each study and, if available, any outcome level assessment. | **Supplement** |
| Results of individual studies | 20 | For all outcomes considered (benefits or harms), present, for each study: 1) simple summary data for each intervention group, and 2) effect estimates and confidence intervals. *Modified approaches may be needed to deal with information from larger networks.* | **Supplement** |
| Synthesis of results | 21 | Present results of each meta-analysis done, including confidence/credible intervals. *In larger networks, authors may focus on comparisons versus a particular comparator (e.g. placebo or standard care), with full findings presented in an appendix. League tables and forest plots may be considered to summarize pairwise comparisons.* If additional summary measures were explored (such as treatment rankings), these should also be presented. | **Supplement** |
| **Exploration for inconsistency** | **S5** | Describe results from investigations of inconsistency. This may include such information as measures of model fit to compare consistency and inconsistency models, *P* values from statistical tests, or summary of inconsistency estimates from different parts of the treatment network. | **6-7**  **Supplement** |
| Risk of bias across studies | 22 | Present results of any assessment of risk of bias across studies for the evidence base being studied. | **6** |
| Results of additional analyses | 23 | Give results of additional analyses, if done (e.g., sensitivity or subgroup analyses, meta-regression analyses*, alternative network geometries studied, alternative choice of prior distributions for Bayesian analyses,* and so forth). | **Supplement** |
| **DISCUSSION** |  |  |  |
| Summary of evidence | 24 | Summarize the main findings, including the strength of evidence for each main outcome; consider their relevance to key groups (e.g., healthcare providers, users, and policy-makers). | **8-10** |
| Limitations | 25 | Discuss limitations at study and outcome level (e.g., risk of bias), and at review level (e.g., incomplete retrieval of identified research, reporting bias). *Comment on the validity of the assumptions, such as transitivity and consistency. Comment on any concerns regarding network geometry (e.g., avoidance of certain comparisons).* | **10** |
| Conclusions | 26 | Provide a general interpretation of the results in the context of other evidence, and implications for future research. | **10** |
| **FUNDING** |  |  | **10** |
| Funding | 27 | Describe sources of funding for the systematic review and other support (e.g., supply of data); role of funders for the systematic review. This should also include information regarding whether funding has been received from manufacturers of treatments in the network and/or whether some of the authors are content experts with professional conflicts of interest that could affect use of treatments in the network. |  |

PICOS = population, intervention, comparators, outcomes, study design.

* Text in italics indicate wording specific to reporting of network meta-analyses that has been added to guidance from the PRISMA statement.

**From: Hutton B, Salanti G, Caldwell DM, et al. The PRISMA Extension Statement for Reporting of Systematic Reviews Incorporating Network Meta-analyses of Health Care Interventions: Checklist and Explanations. Ann Intern Med. 2015;162(11):777-784.^1^

# Search strategy

## Search Strategy Restrictive vs. Liberal Transfusion

MEDLINE (OvidSP)

1. *Blood Transfusion/ad, mt, st, td or *Erythrocyte Transfusion/mt, st, td

2. ((transfus* or red cell* or red blood cell* or RBC* or PRBC*) adj5 (trigger* or thresh?old* or target* or restrict* or liberal* or aggressive* or conservative* or prophylactic* or limit* or protocol* or policy or policies or practic* or indicat* or strateg* or regimen* or criteri* or standard* or management or program*)).tw.

3. ((h?emoglobin or h?ematocrit orHB orHCT) adj5 (polic* or practic* or protocol* or trigger* or threshold* ormaintain* or indicator* or strateg* or criteri* or standard*)).tw.

4. (blood adj3 (management or program*)).mp.

5. ((transfus* or red cell* or red blood cell* or RBC* or PRBC*) and (critical* or intensive* or h?emorrhag* or bleed*)).ti.

6. or/1-5

7. randomized controlled trial.pt.

8. controlled clinical trial.pt.

9. randomi*.tw.

10. placebo.ab.

11. clinical trials as topic.sh.

12. randomly.ab.

13. groups.ab.

14. trial.tw.

15. 7 or 8 or 9 or 10 or 11 or 12 or 13 or 14

16. exp animals/ not humans/

17. 15 not 16

18. 6 and 17

## Search Strategy Tranexamic Acid

1. exp Antifibrinolytic Agents/

2. (anti-fibrinolytic* or antifibrinolytic* or antifibrinolysin* or anti-fibrinolysin* or antiplasmin* or antiplasmin* or ((plasmin or fibrinolysis) adj3 inhibitor*)).ab,ti.

3. exp Aprotinin/

4. (Aprotinin* or kallikrein-trypsin inactivator* or bovine kunitz pancreatic trypsin inhibitor* or bovine pancreatic trypsin inhibitor* or basic pancreatic trypsin inhibitor* or BPTI or contrykal or kontrykal or kontrikal or contrical or dilmintal or iniprol or zymofren or traskolan or antilysin or pulmin or amicar or caprocid or epsamon or epsikapron or antilysin or iniprol or kontrikal or kontrykal or pulmin* or Trasylol or Antilysin Spofa or rp?9921 or antagosan or antilysin or antilysine or apronitin* or apronitrine or bayer a?128 or bovine pancreatic secretory trypsin inhibitor* or contrycal or frey inhibitor* or gordox or kallikrein trypsin inhibitor* or kazal type trypsin inhibitor* or (Kunitz adj3 inhibitor*) or midran or (pancrea* adj2 antitrypsin) or (pancrea* adj2 trypsin inhibitor*) or riker?52g or rp?9921or tracylol or trascolan or trasilol or traskolan or trazylol or zymofren or zymophren).ab,ti.

5. exp Tranexamic Acid/

6. (tranexamic or Cyclohexanecarboxylic Acid* or Methylamine* or amcha or trans-4 aminomethylcyclohexanecarboxylic acid* or t-amcha or amca or kabi 2161 or transamin* or exacyl or amchafibrin or anvitoff or spotof or cyklokapron or ugurol oramino methylcyclohexane carboxylate or aminomethylcyclohexanecarbonic acid or aminomethylcyclohexanecarboxylic acid or AMCHA or amchafibrin or amikapron or aminomethyl cyclohexane carboxylic acid or aminomethyl cyclohexanecarboxylic acid or aminomethylcyclohexane carbonic acid or aminomethylcyclohexane carboxylic acid or aminomethylcyclohexanecarbonic acid or aminomethylcyclohexanecarboxylic acid or aminomethylcyclohexanocarboxylic acid or aminomethylcyclohexanoic acid or amstat or anvitoff or cl?65336 or cl65336 or cyclocapron or cyclokapron or cyklocapron or exacyl or frenolyse or hexacapron or hexakapron or tranex or TXA).ab,ti.

7. exp Aminocaproic Acids/ or exp 6-Aminocaproic Acid/

8. (((aminocaproic or amino?caproic or aminohexanoic or amino?hexanoic or epsilon-aminocaproic or E-aminocaproic) adj2 acid*) or epsikapron or cy-116 or cy116 or epsamon or amicar or caprocid or lederle or Aminocaproic or aminohexanoic or amino caproic or amino n hexanoic or acikaprin or afibrin or capracid or capramol or caprogel or caprolest or caprolisine or caprolysin or capromol or cl 10304 or EACA or eaca roche or ecapron or ekaprol or epsamon or epsicapron or epsilcapramin or epsilon amino caproate or epsilon aminocaproate or epsilonaminocaproic or etha?aminocaproic or ethaaminocaproich or emocaprol or hepin or ipsilon or jd?177or neocaprol or nsc?26154 or tachostyptan).ab,ti.

9. 1 or 2 or 3 or 4 or 5 or 6 or 7 or 8

10. randomi?ed.ab,ti.

11. randomized controlled trial.pt.

12. controlled clinical trial.pt.

13. placebo.ab.

14. clinical trials as topic.sh.

15. randomly.ab.

16. trial.ti.

17. 10 or 11 or 12 or 13 or 14 or 15 or 16

18. (animals not (humans and animals)).sh.

19. 17 not 18

20. 9 and 19

## Search Strategy Iron Therapy

(MedLine search strategy not published) Embase Search Strategy

1 exp iron therapy/

2 (iron or ferrous or ferric).af.

3 1 or 2

4 exp anemia/

5 (anemi* OR anaemi*).af.

6 4 or 5

7 exp crossover-procedure/ or exp double-blind procedure/ or exp randomized controlled trial/ or single-blind procedure/

8 (random* or factorial* or crossover* or placebo*).af.

9 7 or 8

10 3 and 6 and 9

## Search Strategy Point of Care testing

1. exp Thrombelastography/ or Thromb?elastograph*.mp.or (ROTEM or TEG or ROTEG).

mp. or Thromboelastometry.mp.

2. ((randomized controlled trial or controlled clinical trial).pt. or randomized.ab. or placebo.

ab. or drug therapy.fs. or randomly.ab. or trial.ab. or groups.ab.) not (animals not (humans and

animals)).sh. (2177961)

3. 1 and 2

## Search Strategy Cell Salvage

1. cell$ sav$.mp.

2. cell$ salvage.mp.

3. blood transfusion, autologous/

4. autotransfusion$.mp.

5. auto-transfusion$.mp.

6. blood salvage.mp.

7. autovac.mp.

8. solcotrans system.mp.

9. constavac.mp.

10. solcotrans.mp.

11. hemovac.mp.

12. BRAT.mp.

13. fresenius.mp.

14. consta vac.mp.

15. cell saver.mp.

16. dideco.mp.

17. electromedic.mp.

18. electromedics.mp.

19. gish biomedical.mp.

20. haemonetics.mp.

21. orth-evac.mp.

22. pleur-evac.mp.

23. sorenson.mp.

24. reinfusion system.mp.

25. sorin biomedical.mp.

26. or/1-25

27. exp blood transfusion/

28. exp hemorrhage/

29. exp anesthesia/

30. transfusion$.mp.

31. bleed$.mp.

32. blood loss$.mp.

33. hemorrhag$.mp.

34. haemorrhag$.mp.

35. or/27-34

36. 26 and 35

37. randomized controlled trial.pt.

38. controlled clinical trial.pt.

39. randomized controlled trials.sh.

40. random allocation.sh.

41. double blind method.sh.

42. single blind method.sh.

43. or/37-42

44. clinical trial.pt.

45. exp Clinical trials/

46. (clin$ adj25 trial$).ti,ab.

47. ((singl$ or doubl$ or trebl$ or tripl$) adj25 (blind$ or mask$)).ti,ab.

48. placebos.sh.

49. placebo$.ti,ab.

50. random$.ti,ab.

51. research design.sh.

52. or/44-51

53. comparative study.sh.

54. exp Evaluation studies/

55. follow up studies.sh.

56. prospective studies.sh.

57. (control$ or prospectiv$ or volunteer$).ti,ab.

58. or/53-57

59. 43 or 52 or 58

60. 36 and 59

61. animal/ not human/

62. 60 not 61

## Search Strategy for Cost Effectiveness

Medline search terms

1 exp blood transfusion/

2 ((blood or red cell or rbc or platelet* or plasma or ffp or cryoprecipitate or prothrombin) adj3 (transfus* or retransfus* or therap*)).ti,ab.

3 (hemotransfus* or haemotransfus*).ti,ab.

4 ((blood adj2 (management or administ*5 or component*1)) or blood support).ti,ab.

5 or/1-4

Embase search terms

1 exp *blood transfusion/

2 ((blood or red cell or rbc or platelet* or plasma or ffp or cryoprecipitate or prothrombin) adj3 (transfus* or retransfus* or therap*)).ti,ab.

3 (hemotransfus* or haemotransfus*).ti,ab.

4 ((blood adj2 (management or administ*5 or component*1)) or blood support).ti,ab.

5 or/1-4

CRD search terms

#1 mesh descriptor blood transfusion explode all trees in NHSEED,HTA

#2 (((blood or red cell or RBC or platelet* or plasma or ffp or cryoprecipitate or prothrombin) adj3 (transfus* or retransfus* or therap*))) in NHSEED, HTA

#3 ((hemotransfus* or haemotransfus*)) in NHSEED, HTA

#4 (blood adj2 (management or administ* or component*)) OR (blood support) in NHSEED, HTA

#5 #1 or #2 or #3 or #4

# Detailed methods and criteria for considering studies for this review

## Searching other resources

The reference lists of eligible trials and reviews were examined. Searches were not restricted by language or publication status. Trial registrations and abstracts within the included studies were cross-checked.

## Selection of studies

Three reviewers (MR, RA and SP) screened the search results and identified trials for inclusion. The full texts of these studies were retrieved and assessed for inclusion. Cross validation of 10% of the selected studies was performed by the lead author (GJM) to assess inter observer reproducibility. Excluded studies and the reason for exclusion were recorded.^2^ Disagreements were resolved by discussion and consensus. In instances where this was not possible the Lead Author (GJM) determined whether or not the study was included.

## Data extraction and management

Three independent reviewers (MR, RA, and SP) extracted data independently onto electronic databases from the included studies for assessment of study quality and evidence synthesis. The extracted information included: year and language of publication; country of participants recruitment; year of the trial completion; study population; inclusion and exclusion criteria; sample size; participant demographics; baseline characteristics; cardiovascular disease, cancer, anaemia; type of surgery; details of interventions: Iron Therapy, Tranexamic Acid, Restrictive Transfusion, POC testing, Cell salvage; details of comparator: Untreated groups, or alternative goal directed therapy groups (e.g. standard care); and pre-defined primary and secondary outcomes and times of measurement.

Where there was more than one intervention group, the data was extracted for the group with the highest effect on outcomes in the opinion of the investigators (e.g. IV iron when compared to oral iron treatment). Cross validation of 10% of each search was performed by MR and RA to assess inter observer reproducibility. The discrepancies identified was resolved through discussion. Where no consensus was reached, this was discussed with a third reviewer (GJM). Missing data was requested from study authors where possible. If there was doubt as to whether trials shared participants completely or partially (with common authors and centres) we contacted the study authors to ascertain whether the study report was duplicated.

## Assessment of risk of bias in included studies

The following bias risk domains were assessed as Low, Unclear, or High, based on the guidelines in the Cochrane Handbook for Systematic Reviews of Interventions^3^:sequence Generation; allocation Concealment; blinding of participants; blinding of investigators; incomplete outcome data;and selective outcome reporting.

Trials were classified as having a low risk of bias if they are graded as being at low risk of bias in all of these domains. Three review authors (MR, RA, and SP) independently assessed the risk of bias at study level in all of the studies. Discrepancies were resolved by discussion.^4^

## Measures of treatment effect

For dichotomous variables, the number of events in the treatment and control groups were collected, and the risk ratio (RR) with 95% confidence interval (CI) was calculated. For continuous variables, the standardised mean difference (SMD) with 95% CI were calculated. The treatment effects were analysed using random effects models.

## Likely sources of heterogeneity

The results of each outcome were combined and the effects assessed as a combined or treatment specific measure.

Use of unwashed red cells, comparison of multiple formulations without controls, comparisons of intervention with another therapy (e.g. Erythropoietin), gynaecological/obstetric and neonatal populations were considered significant sources of heterogeneity and excluded from the analysis.

Clinical setting: cardiac surgery, orthopaedic surgery, hepatobiliary surgery, urogenital surgery, other.

Interventions: pre-surgery iron therapy, cell salvage, washing, and auto-transfusion, restrictive red cell transfusion thresholds, tranexamic acid, point-of-care testing algorithms for the management of coagulopathy.

Interventions that target anaemia versus those that primarily target bleeding.

Comorbidity: cardiovascular disease, cancer, renal disease, anaemia at baseline, sepsis, bleeding risk.

Concomitant administration of other blood saving techniques: Pre-surgery iron therapy, cell salvage, washing, and auto-transfusion, restrictive red cell transfusion thresholds, tranexamic acid, point-of-care testing algorithms for the management of coagulopathy.

## Assessment of heterogeneity

The inconsistency within each meta-analysis was explored by Cochrane’s Q^2^ test (with significance set at a P value of 0.10), and I^2^ statistics.^5^

We considered:

I^2^ 0-40%: no or mild heterogeneity

I^2^ 40-80%: moderate heterogeneity

I^2^ > 80%: severe heterogeneity

## Investigation of heterogeneity

Subgroup analyses considered likely sources of inconsistency as pre-specified in the study protocol including clinical setting, type of intervention, interventions that targeted anaemia versus those that primarily targeted bleeding, comorbidity, and concomitant administration of other blood saving techniques. Test for sub-group differences^6^ was used with a P value of <0.05 considered statistically significant.

## Assessment of reporting biases

Where 10 or more studies are identified for each outcome, publication bias was quantified through the visual assessment of funnel plots and Eggers test. Where more than 10 included studies are of varying study size, the funnel plots were also used to assess small study effects. A sensitivity analysis evaluated the potential impact of the small studies by assessing random-effects analyses.

## Unit of analysis issues

The review authors extracted the data on a per participant basis. Discrepancies in randomization units were analysed for each study based on:groups of individuals that were randomized together to the same intervention; individuals undergoing more than one intervention;and if there were multiple observations for the same outcome (repeated measurements or recurring events).

These discrepancies were mitigated by contacting the study authors to obtain clarification in the randomisation units. For the individuals undergoing more than one intervention, this was recorded and included in the network meta-analysis as a concomitant intervention if it was one of the 5 interventions included in the statistical plan. Studies that had concomitant interventions aimed to reduce blood transfusions (e.g. Aprotinin, Epinephrine) were excluded from this analysis. If repeated measures were encountered, the first reported time measure was used in this analysis. Hazard ratios and time effect analyses were not performed.

## Dealing with missing data

An intention-to-treat analysis was performed where possible. For dichotomous data presented only as percentages the frequencies were estimated by using reported sample sizes for this outcome. For continuous outcomes if the mean and the standard deviation were not available from the trial report, this information was sought from the trial authors. If this information was still not available, the mean and standard deviation were calculated from median (interquartile ranges) using the Review Manager Version 5.3 software.

## Summary of findings

The main results of the review were presented in a ‘Summary of findings’ table. The following outcomes were recorded:30 day or hospital all-cause mortality; risk of stroke, myocardial infarction, or severe acute kidney injury; low cardiac output; sepsis and infection; perioperative blood loss; re-operation for bleeding; risk of receiving blood transfusions; risk of receiving haemostatic agents; and resource Use: ICU and Hospital length of stay.

The GRADEPRO software was used to prepare the ’Summary of findings’ table. The overall quality of the evidence for each outcome was interpreted as: ‘high,’ ‘moderate,’ ‘low’ or ‘very low’ according to the GRADE (Grades of Recommendation, Assessment, Development and Evaluation) approach.^7^ The impact of the following factors on the outcomes was assessed^6^:impact of risk of bias of individual trials; precision of pooled estimate. Inconsistency or heterogeneity (clinical, methodological and statistical); indirectness of evidence;and impact of selective reporting and publication bias on effect estimate.

## Network meta-analysis (NMA) methodology

We conducted network meta-analysis to compare multiple patient blood management interventions simultaneously. In this analysis, we considered the concomitant interventions specified in the included studies. We adopted a frequentist approach using the R package netmeta^8^ based on the graph-theoretical methods that were originally developed for the electrical network theory.^9^ Separate network meta-analysis was conducted for each outcome. In each analysis, a network diagram was produced with the nodes representing the interventions and the edges displaying the observed direct comparisons of interventions. The thickness of the edges is in proportion to the number of studies examining such comparisons. Interventions that were not connected to the network were excluded from the analysis. We used the consistency network meta-analysis model and reported the results of random-effects (rather than fixed-effect) meta-analysis. Statistical heterogeneity was evaluated using tau2, I2 statistics and Cochran’s Q. Patient blood management interventions were ranked using P-score which measures the extent to which an intervention is better than another intervention, averaged over all competing interventions.^10^ Network estimates were split into the contribution of direct and indirect evidence using Back-calculation method and the agreement between the estimates from direct and indirect evidence was tested for local consistency.^11^

# PRISMA flow diagrams (eFigure 1. A-G)

# A


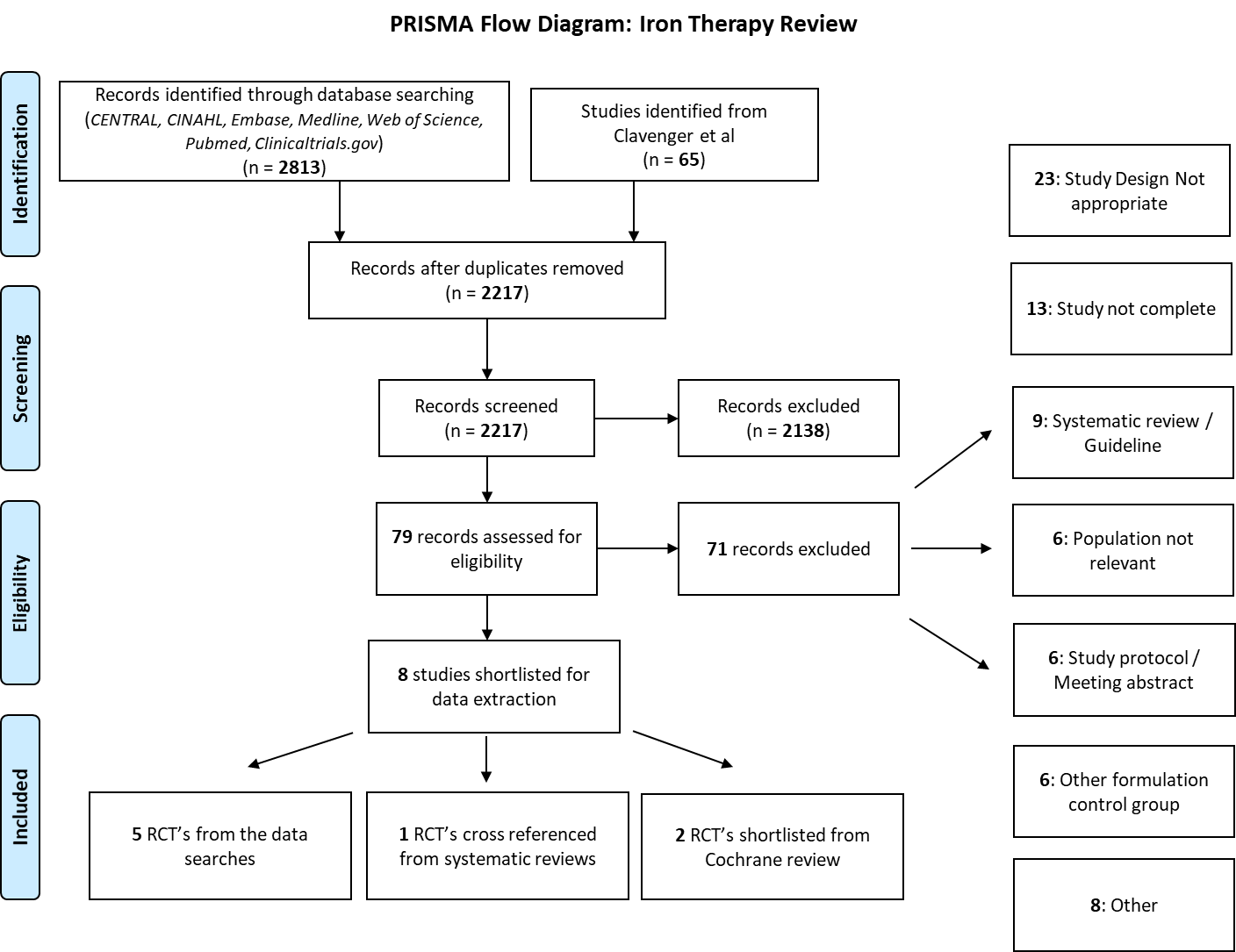


**B**

**
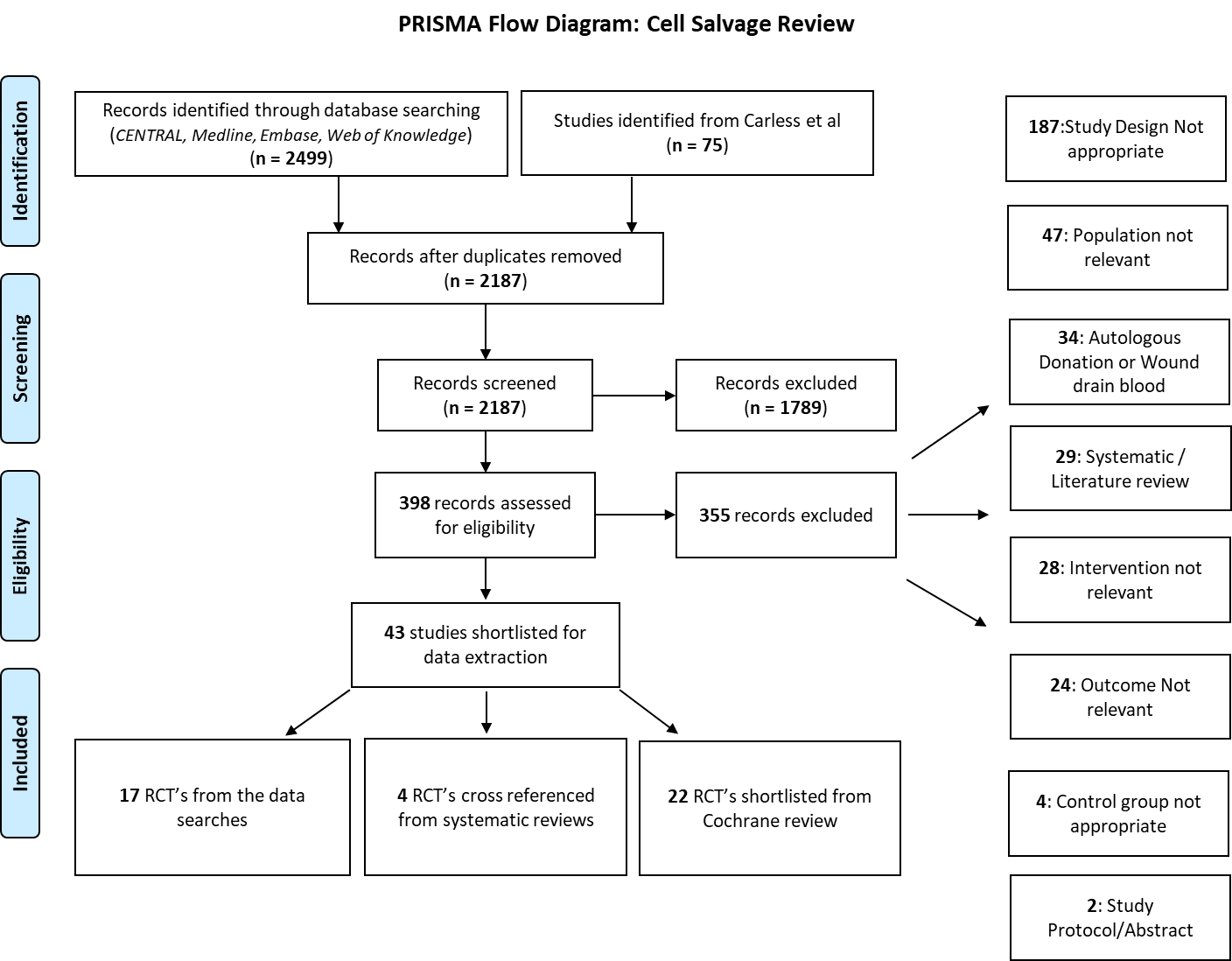
**

**C**


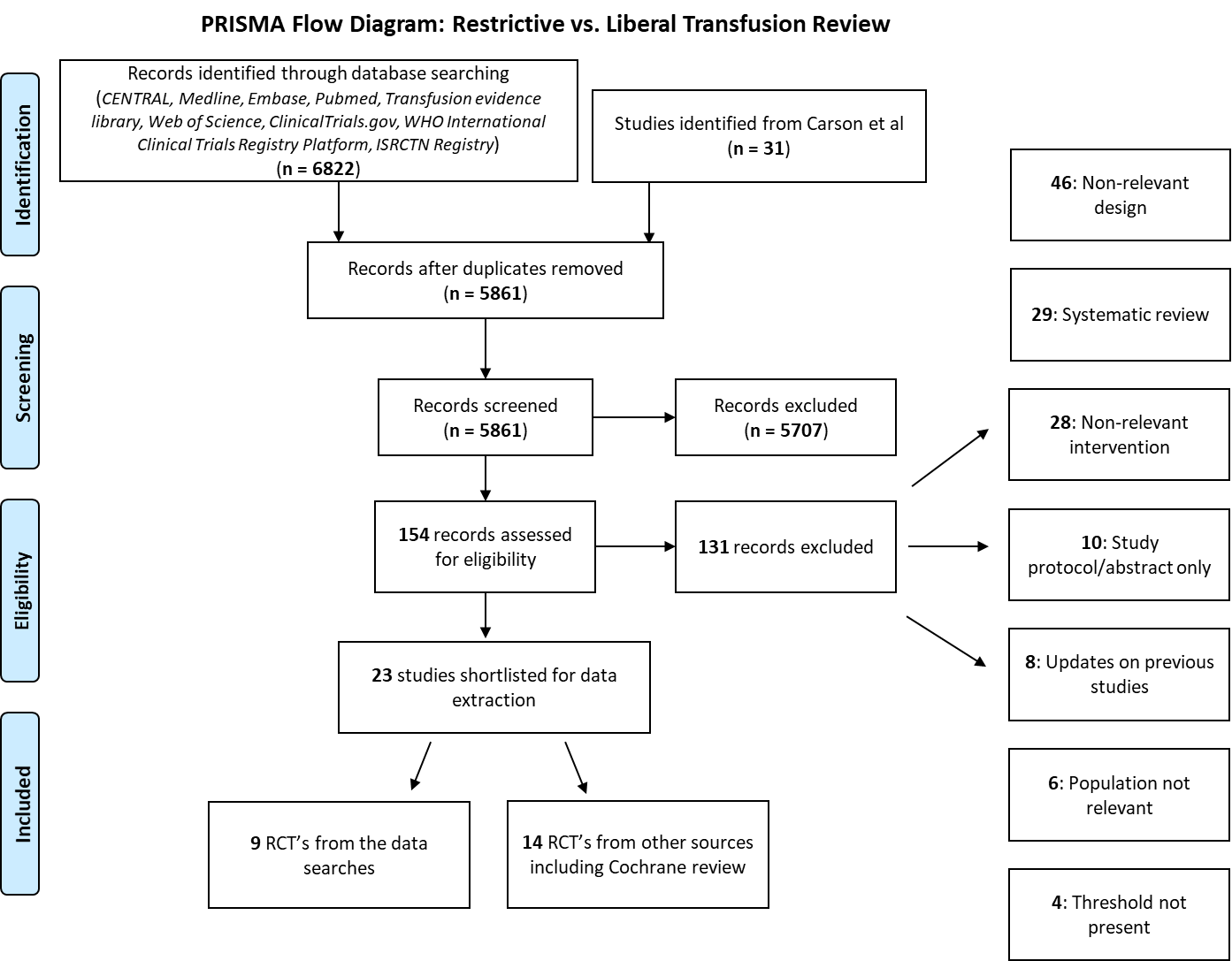


**D**


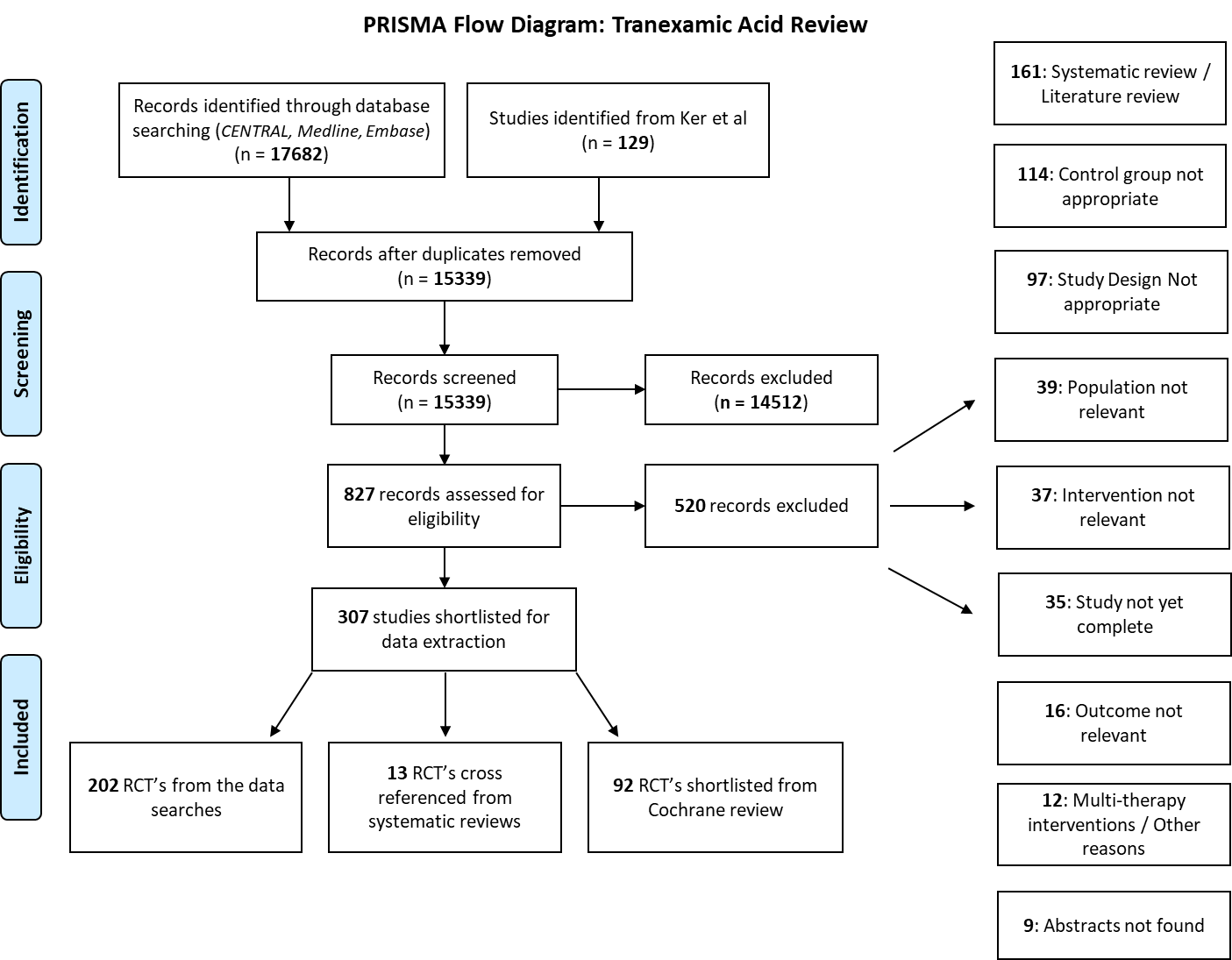


**E**


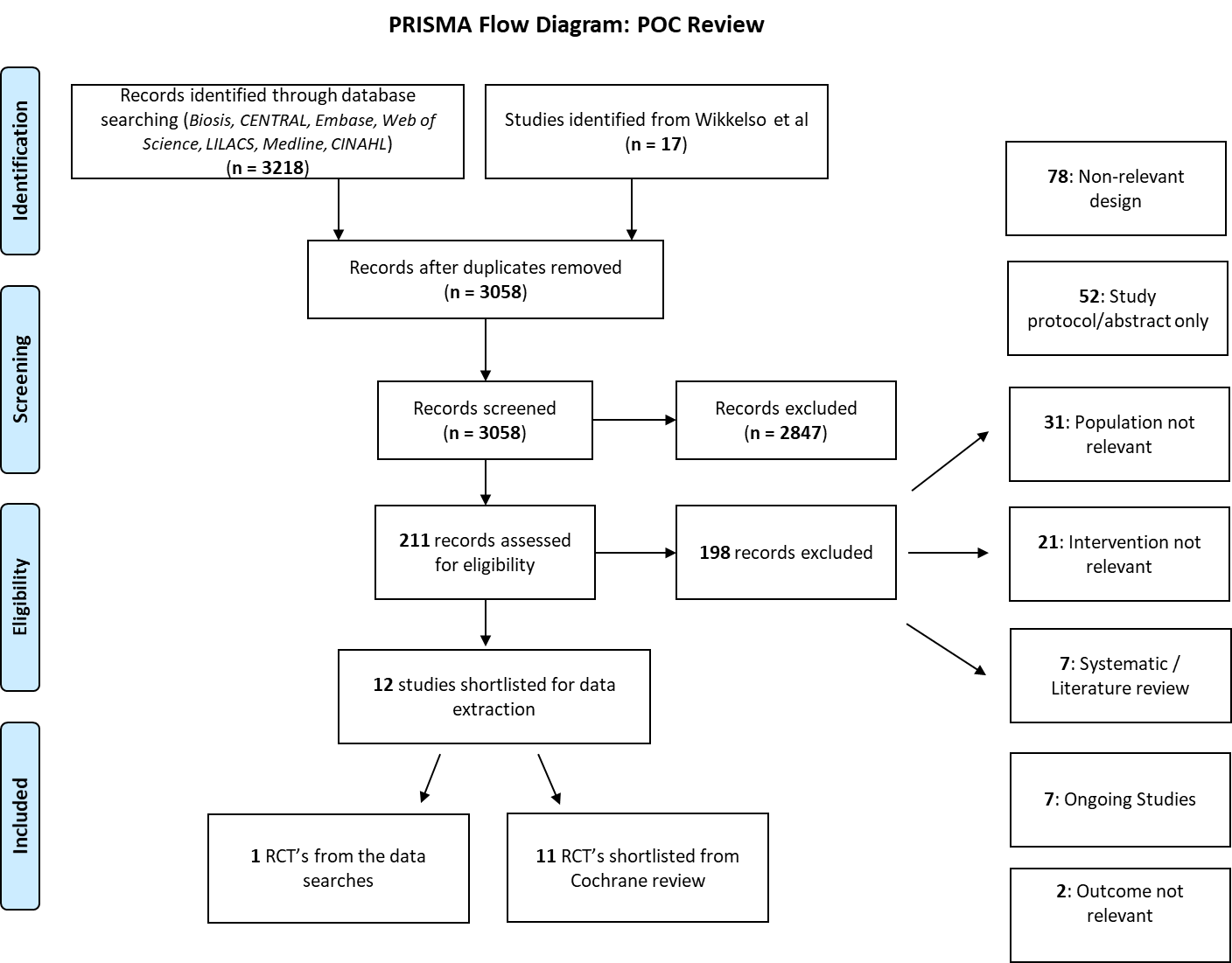


**F**


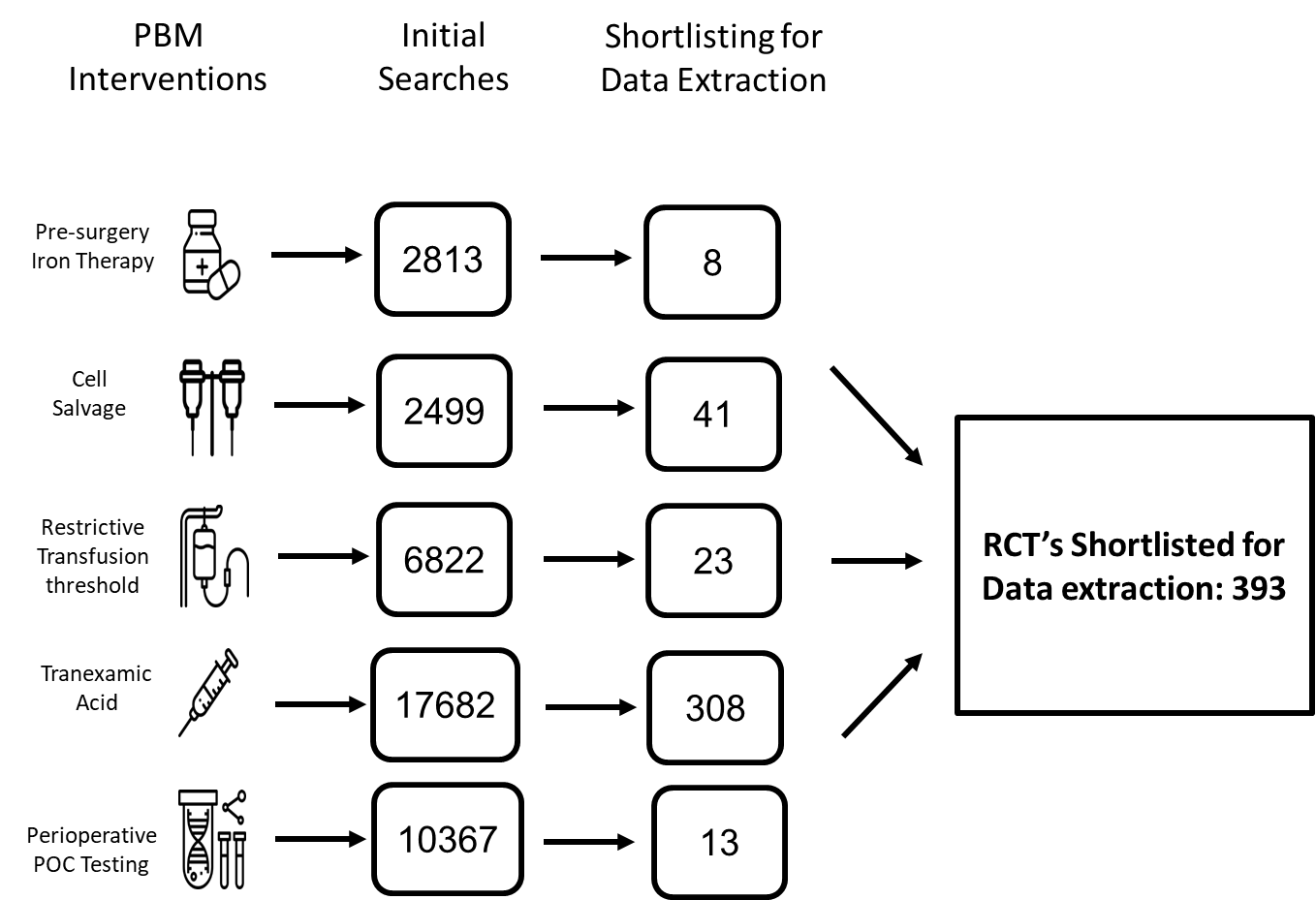


**G**


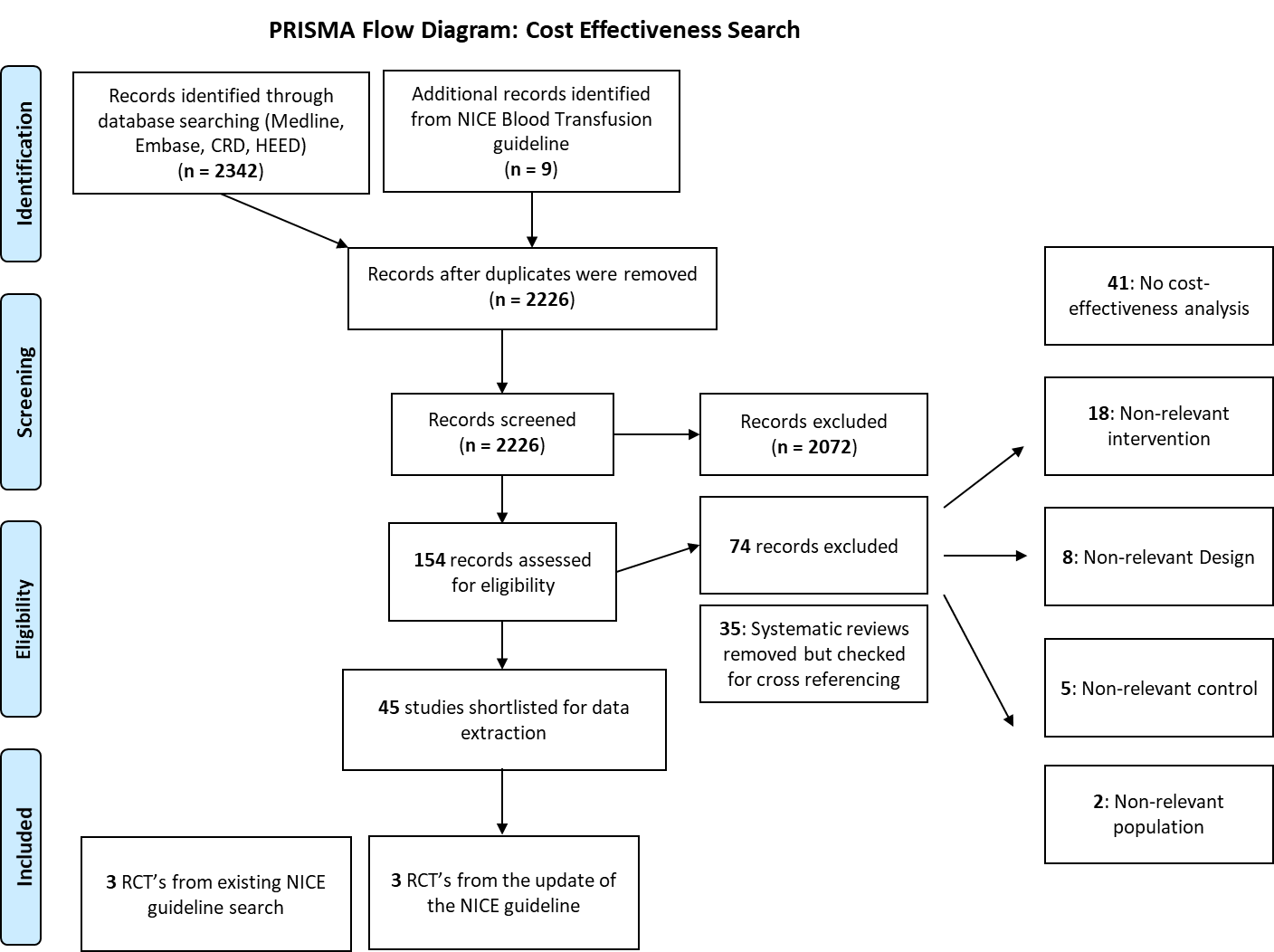


# Table of reasons for excluded studies following assessment of full texts (eTable 1.)

- ***Iron therapy (studies included in Clevenger et al review^12^)***

| **Study Reference** | **Exclusion criteria** |
| --- | --- |
| **13-63** | Population not relevant (e.g. non-surgical population) |
| **64-68** | Post-surgery delayed intervention (e.g. 4 weeks post-surgery) |
| **69,70** | Poster / Conference Abstract |
| **71** | Outcomes not relevant |

- ***Iron therapy (studies obtained from database searches)***

| **Study References** | **Exclusion criteria** |
| --- | --- |
| **72-94** | Study design not appropriate (e.g. retrospective / observational studies) |
| **95-107** | Study incomplete, ongoing or published protocol |
| **108-116** | Systematic review, literature review or guideline |
| **117-122** | Population not relevant (e.g. obstetric, neonatal) |
| **123-126** | Intervention not relevant (e.g. treatment with Erythropoietin) |
| **127,128** | Other (Update / Correspondence on previous studies / Outcomes not relevant) |

- ***Cell Salvage (studies included in Carless et al review^129^)***

| **Study Reference** | **Exclusion criteria** |
| --- | --- |
| **130-164** | Non-washed red cells (e.g. Auto-transfusion directly from drain systems) |
| **165-171** | Design not relevant (e.g. non-randomised) |
| **172-177** | Outcome not relevant (doesn’t include the specified primary outcomes) |
| **178** | Control group not appropriate (e.g. other cell saving devices) |

- ***Cell Salvage (studies obtained from database searches)***

| **Study References** | **Exclusion criteria** |
| --- | --- |
| **179-365** | Study design not appropriate (e.g. retrospective / observational studies) |
| **366-412** | Population not relevant (e.g. obstetric, neonatal) |
| **338,413-444** | Autologous Donation or Wound drain blood |
| **445-473** | Systematic review, literature review or guideline |
| **412,474-500** | Intervention not relevant (e.g. no cell salvage, red cells not washed) |
| **501-524** | Outcome not relevant |
| **525-528** | Control group not appropriate (e.g. comparison with another cell salvage method) |
| **529,530** | Study incomplete, ongoing or published protocol |

- ***Restrictive vs. Liberal Transfusion threshold (studies included in Carson et al review^531^)***

| **Study References** | **Exclusion criteria** |
| --- | --- |
| **532-549** | Population not relevant (e.g. non-surgical patients, obstetric, neonatal) |
| **550-554** | Study incomplete, ongoing or published protocol |

- ***Restrictive vs. Liberal Transfusion threshold (studies obtained from database searches)***

| **Study Reference** | **Exclusion criteria** |
| --- | --- |
| **555-600** | Design not relevant (e.g. non-randomised) |
| **601-629** | Systematic / Literature review / Meta-analysis |
| **630-657** | Intervention not relevant (e.g. no clear restrictive vs liberal strategy) |
| **658-666** | Study protocol / report / meeting abstract |
| **667-674** | Update on previous studies |
| **675-680** | Population not relevant (e.g. Neonatal populations) |
| **681-684** | Threshold not present |

- ***Tranexamic acid (studies included in Ker et al review^685^)***

| **Study Reference** | **Exclusion criteria** |
| --- | --- |
| **686-690** | Study incomplete, ongoing or published protocol |
| **691-702** | Population not relevant (e.g. Neonatal) |
| **703,704** | Outcome not relevant (doesn’t include the specified primary outcomes) |
| **705** | Multi-therapy interventions (including concomitant medications to reduce bleeding) / Other reasons |
| **706** | Control group not appropriate (e.g. comparison with another formulation only or not standard care treatment) |

- ***Tranexamic acid (studies obtained from database searches)***

| **Study References** | **Exclusion criteria** |
| --- | --- |
| **685,707-838** | Systematic review, literature review or guideline |
| **839-975** | Control group not appropriate (e.g. comparison with another formulation only or not standard care treatment) |
| **976-1072** | Study design not appropriate (e.g. retrospective / observational studies) |
| **1073-1111** | Population not relevant (e.g. obstetric, neonatal) |
| **1112-1148** | Intervention not relevant (e.g. no tranexamic acid treatment) |
| **1149-1183** | Study incomplete, ongoing or published protocol |
| **1184-1199** | Outcome not relevant |
| **1200-1209** | Multi-therapy interventions (including concomitant medications to reduce bleeding) / Other reasons |
| **1210-1218** | Abstracts not found |

- ***Point of care (studies included in Wikkelsø et al review^1219^)***

| **Study Reference** | **Exclusion criteria** |
| --- | --- |
| **1220-1223** | Population not relevant (e.g. Neonatal populations) |
| **1224-1226** | Study protocol / Meeting abstract / Letter to Editor only |
| **1227-1230** | Ongoing studies, results unpublished or trial terminated |

- ***Point of care (studies obtained from database searches)***

| **Study References** | **Exclusion criteria** |
| --- | --- |
| **1231-1307** | Study design not appropriate (e.g. retrospective / observational studies) |
| **666,1308-1358** | Study protocol / Meeting abstract / Letter to Editor |
| **1281,1359-1388** | Population not relevant (e.g. obstetric, neonatal) |
| **1189,1389-1408** | Intervention not relevant (e.g. no cell salvage, red cells not washed) |
| **1409-1415** | Systematic review, literature review or guideline |
| **1416-1422** | Study incomplete, ongoing or published protocol |
| **1423,1424** | Outcome not relevant |

# Characteristics of included studies (eTable 2.)

Eight trials evaluated preoperative iron therapy, enrolling 1031 participants. The average age of participants in these trials ranged from 55.1 to 82.5 years (95% CI 60.7, 73.5) in the intervention group and 53.2 to 83.4 years (95% CI 60.9, 75) in the control group in the trials that provided this information. The numbers of patients enrolled ranged from 49 to 484 across trials. 1, 4 and 3 trials were in orthopaedic, cardiac and general surgery respectively. 1 trial did not state the setting. 5, 3, 2 and 5 were undertaken in patients with cardiovascular disease, cancer, anaemia, or elevated bleeding risk respectively.

Forty three trials evaluated perioperative cell salvage enrolling 6083 participants. The average age of participants in these trials ranged from 0.25 to 72 years (95% CI 54.9, 64.6) in the intervention group and 0.4 to 74 years (95% CI 55.1, 64.9) in the control group in the trials that provided this information. The numbers of patients enrolled ranged from 20 to 1759 across trials. 16, 23, 3 and 1 trials were in orthopaedic, cardiac, vascular and congenital cardiac surgery respectively. 27, 0, 3 and 31 were undertaken in patients with cardiovascular disease, cancer, anaemia, or elevated bleeding risk respectively.

Twenty three trials evaluated restrictive transfusion thresholds enrolling 13324 participants. The average age of participants in these trials ranged from 41 to 86 years (95% CI 64.4, 73.3) in the intervention group and 41 to 88 years (95% CI 65.1, 74.1) in the control group in the trials that provided this information. The numbers of patients enrolled ranged from 38 to 4860 across trials. 10, 8, 2, 1, 1 and 1 trials were in orthopaedic surgery, cardiac, vascular, general, plastic and oncological surgery respectively. 28, 0, 4 and 9 were undertaken in patients with cardiovascular disease, cancer, anaemia, or elevated bleeding risk respectively.

Three hundred and seven trials evaluated tranexamic acid enrolling 33770 participants. The average age of participants in these trials ranged from 1 to 84.3 years (95% CI 57, 60.6) in the intervention group and 1.1 to 84.5 years (95% CI 56.7, 60.3) in the control group in the trials that provided this information. The numbers of patients enrolled ranged from 10 to 4631 across trials. 182 orthopaedic, 74 cardiac, 10 urology, 10 ENT, 8 spinal, 6 maxillo-facial, 5 hepatobiliary, 5 plastic, 2 neurosurgery, 1 general, 1 oncological, 1 congenital, 2 thoracic surgery trials were included. 77, 11, 29 and 99 were undertaken in patients with cardiovascular disease, cancer, anaemia, or elevated bleeding risk respectively.

Thirteen trials evaluated point of care testing algorithms for the management of coagulopathy bleeding enrolling 907 participants. The average age of participants in these trials ranged from 2.5 to 72 years (95% CI 45.7, 72) in the intervention group and 3.7 to 70.5 years (95% CI 43.1, 71.5) in the control group.

## **Controls:**

The type of controls included for the different interventions were:

- Iron treatment
- 6 placebo
- 2 standard of care
- Cell salvage
- 22 had no intervention
- 16 had normal drainage
- 5 were non cell salvage transfusion
- Restrictive vs. liberal threshold
- 23 liberal thresholds
- Tranexamic acid
- 225 had placebo treatment
- 82 had no intervention;
- Point of care
- 12 received standard of care.

## Co-interventions:

For the network meta-analysis all the possible combinations of co-interventions were considered based on the concomitant interventions reported in the individual trials. All possible combinations between Iron treatment, Cell salvage, Restrictive vs. Liberal threshold, Tranexamic acid, and Point of Care were considered. These are listed in the network meta-analyses in in eFigure 6, 9 and 12.

| **Study**  ***(Author, Year)*** | | - **Country** - **Language** - **Year of the trial completion** - **Single- or Multi-Centre** - **Study population size (n)** - **Inclusion criteria (descriptive)** | | **Exclusion criteria**  **(descriptive)** | | **Demographics (Age between groups)** | **Comorbidities (CV disease, Cancer, Renal Disease, Anaemia)** | | **Type of Surgery** | - **Type of Intervention (subtype if available)** - **Type of Control** - **Concomitant PBMs (list)** | **Primary Outcomes**  **(list)** | **Secondary Actual Outcomes**  **(list)** |
| --- | --- | --- | --- | --- | --- | --- | --- | --- | --- | --- | --- | --- |
| - ***Iron Treatment*** | | | | | | | | | | | | |
| Bernabeu Wittel 2016^123^ | | - Spain - English - 2016 - Multi-Centre - 303 - Patients >65years admitted with hip fracture and Hb level 90-120 g/L | | Marrow diseases that could interfere in the erythropoietic process, blood coagulation diseases or current treatment with anticoagulants, documented allergy or intolerance and/or contraindication to EPO use and/or IV iron, rheumatoid arthritis and/or another demonstrated origin of inflammatory anaemia and/or uncontrolled arterial hypertension, current or previous treatment with EPO or IV iron for at least 3 months, and chronic renal failure receiving haemodialysis or peritoneal dialysis. | | 83.4(6.4)  Vs  84.6(6.3)  Vs  82.3(6.9) | CV disease  Renal disease  Anaemia | Orthopaedic Surgery | | - S/C EPO + IV Fe - IV Fe - Placebo | Percentage of patients receiving RBC transfusion | - Survival  - Number of RBC transfused/patient  - Haemoglobinaemia - Health-related quality of life |
| Edwards 2009^1425^ | | - UK - English - 2009 - Single-Centre - 60 - All patients scheduled to undergo bowel resection for suspected colorectal cancer at the centre during the study period. | | Patients were excluded if age <18 years, those receiving oral iron/blood transfusion supplementation within 6 weeks of being approached, if the date of their scheduled surgery fell within 15 days of the date of recruitment | | 67 Vs 70 | Cancer | General Surgery | | - IV Fe - Placebo | Median number of units transfused at peri-operative period. | Transfusion rate   - Changes in serum iron markers over the same time period - Length of hospital stay - Adverse perioperative events. |
| Froessler 2016^1426^ | | - Australia - English - 2014 - 72 - Patients undergoing abdominal surgery with iron deficiency anaemia between August 2011 and November 2014. (>18 yrs with IDA, ferritin <300 mcg/L, transferrin saturation <25%, Hb <12.0 g/dL for women, Hb <13.0 g/dL for men | | Not stated | | 64.5(15)  Vs  68(15) | Cancer  Anaemia | General Surgery | | - IV Fe - Standard Care | Incidence of Autologus Blood Transfusion | - Haemoglobin (Hb) on admission  - Hb difference from randomization to admission  - ICU admission  - Perioperative morbidity (defined as new onset infection, respiratory failure, renal impairment, deep venous thrombosis)  - Discharge Hb  - Length of stay  - Hb at follow-up  - Hb difference from discharge to follow-up  - Iron status  - 30-day mortality  - Quality of life (QoL) |
| Garrido-Martin 2012^1427^ | | - Spain - English - 2012 - Single-Centre - 210 - Patients older than 18 years of age, elective cardiac surgery under extracorporeal circulation, without previous anaemia, susceptible to treatment, without preoperative blood transfusion, able to complete all study visits per protocol and providing written informed consent | | Elective cardiac surgery patients without extracorporeal circulation, treatment with fibrinolytic therapy 48 h before CPB surgery, history of impaired renal function (creatinine clearance <50 ml/min), previous surgery for active endocarditis, redo-surgery patients, pregnant or lactating, signs of active gastrointestinal bleeding, vitamin B12 deficit, ferropenic anaemia, clinical history of asthma or allergy, active infection, included in another clinical study, hepatic disease, history of allergy to iron, unlikely to adhere to protocol follow-up, unable to comply with the study protocol. | | 65(11)  Vs  65(10)  Vs.  65(12) | CV disease | Cardiac Surgery | | - IV Fe - Oral Fe - Placebo | Number of patients transfused at end of follow up | - Protocol outcomes not reported by the study Quality of life at end of follow-up  - Length of hospital stay at end of follow-up  - Mortality (all causes) at 30 days  - Mortality  (transfusion related) at 30 days  - Infections (includes pneumonia, surgical site infection, UTI and  septicaemia/bacteraemia) at within 30 days of surgery  - Bleeding at end of follow-up  - Serious adverse events (as  described in studies) at end of follow-up  - Mortality (all causes) at 1 year  - Thrombosis at end of follow-up  - Number of  units transfused at end of follow-up |
| Johansson 2015^1428^ | | - Denmark - English - 2013 - 60 - Non-anaemic patients undergoing cardiac surgery | | Iron overload or disturbances in utilization of iron (e.g. haemochromatosis and haemosiderosis), s-ferritin >800 ng/ml, known hypersensitivity to any excipients in the investigational drug products, history of multiple allergies, decompensated liver cirrhosis and hepatitis, alanine aminotransferase >3 times normal upper value, acute infections, rheumatoid arthritis with symptoms or signs of active joint inflammation, pregnant or nursing women, participation in any other clinical trial where the trial drug had not passed five half-lives prior to screening, untreated vitamin B12 or folate deficiency, other IV or oral iron treatment within 4 weeks prior to screening visit, erythropoietin treatment within 4 weeks prior to screening visit, and impaired renal function defined by creatinine >150 mol/L. Patients who received blood transfusion <30 days before screening and/or during the elective or subacute CABG, valve replacement or a combination | | 65(9.2)  Vs  65(10) | CV disease | Cardiac Surgery | | - IV Fe - Placebo | Change in Hb concentrations from baseline to 4 weeks postoperatively | - Proportion of patients who were anaemic (women Hb <12 g/dl and men Hb <13 g/dl) at day 5 and week 4,  - Proportion of patients who were able to maintain a Hb between 9·5 and 12·5 g/dl (both values included) at day 5 and week 4  - Number of patients in each treatment group who needed blood transfusion and number of transfusions administered  - Change from baseline in concentrations of s-ferritin, s-iron, transferrin saturation (TSAT) and reticulocytes at day 5 and week 4  - Safety (adverse events, vital signs, electrocardiogram (ECG), s-phosphate, and haematology and biochemistry parameters). |
| Lidder 2007^1429^ | | - UK - English - 2007 - Single-Centre - 49 - Patients diagnosed with colorectal cancer who are fit for surgery | | Not stated | | 69(12.1)  Vs  72(6.6) | Cancer | General Surgery | | - Oral Fe - Standard Care - - | - | Functional Recovery  Hospital LOS  Risk & number of RBC transfusion  Perioperative blood loss |
| Serrano-Trenas 2011^1430^ | | - Spain - English - 2008 - Single-Centre - 200 - Patients aged over 65 undergoing hip fracture surgery at the Orthopaedic and Trauma Surgery Unit of the Hospital Reina Sofia in Córdoba (Spain) between October 2006 and October 2008 | | Patients with diseases diagnosed before the admission of patient (iron overload disorders, hypersensitivity to oral or parenteral iron preparations, asthma or other severe atopic, active infection or neoplasm),  treatment with Clopidogrel or with acetylsalicylic acid at dose rates greater than 150 mg/24 hr, no surgical indication for the current fracture, disorders impaired coagulation (partial thromboplastin time > 2.5%, international normalized ratio > 1.5), liver disorders with elevated transaminases (aspartase aminotransferase [AST] > 70 U/L, alanine aminotransferase [ALT] > 55 U/L), and chronic kidney failure (creatinine > 2 mg/dL) or patients including in dialysis. | | 82.53(6.37)  Vs  83.46(7.11) | CV disease | Orthopaedic Surgery | | - IV Fe - No treatment | 30-day mortality | Functional Recovery  Sepsis  Hospital LOS  Risk & number of RBC transfusion  Risk of receiving non red cell component |
| Spahn 2019^1431^ | | - Switzerland - English - 2019 - Single-Centre - 484 - Adult patients with anaemia scheduled for elective isolated coronary artery bypass grafting (CABG), valve surgery, and combined CABG and valve procedures were eligible | | - Patients in need of urgent surgery the day of hospital admission  - Participation in another clinical trial during the last 4 weeks prior to patient screening  - Impairments, diseases or language problems which do not allow the patient to fully understand the consequences of study participation  - Age < 18 years- Pregnant and/or breastfeeding women  - Jehovah's Witnesses  - Patients suffering from endocarditis- Known allergy against iron-carboxymaltose or mannitol- Need for intraoperative extra-corporeal membrane oxygenation  - Untreatable surgical bleeding with massive transfusion (≥ 10 red blood cell (RBC) transfusions per 24h | | 73(12) Vs 75(10) | CV disease | Cardiac Surgery | | - IV Fe - Placebo - Restrictive threshold | number of RBC  transfusions administered during the first 7 days (starting  with the day of operation), until death or hospital  discharge, whichever came first | **7 day (short)**: acute kidney injury (increase of creatinine >50% vs preoperative value), infections requiring antibiotic treatment and perioperative course of Hb, reticulocyte count, reticulocyte Hb content,  platelet and leucocyte counts, international normalised ratio, high-sensitivity troponin, creatinine, C-reactiveprotein, calculated RBC loss (preoperative RBC mass minus RBC mass at postoperative day 5 plus transfused RBC mass10) as well as tolerance of study drugs and placebo administration.  **90 days secondary** outcomes: percentage of patients without any RBC transfusion, number of allogeneic blood products (RBC, plasma, platelets) administered, length of stay in intensive care and in hospital, duration of mechanical ventilation, major adverse cardiac and  cerebrovascular events, new onset of atrial fibrillation, thrombotic and thromboembolic complications, mortality,  product acquisition costs, and the occurrence of  serious adverse events |
| Xu 2019^1432^ | | - China - English - 2018 - Single-Centre - 150 - Patients aged 20 to 70 years and elective cardiac valvular surgery under extracorporeal circulation, without preoperative anaemia and blood transfusion. | | (1) history of iron allergy;  (2) determined iron overload or hereditary iron utilization disorder;  (3) Severe hepatic insufficiency (alanine aminotransferase >3 times normal upper value). | | 55.1(11) Vs 53.2(11.4) | CV disease | Cardiac Surgery | | - IV Fe - Placebo - Restrictive threshold | changes in Hb concentration  on POD 7 and POD 14 between the 2 groups | changes in HCT, RBC count, serum ferritin and transferrin saturation, the length of ventilation,  ICU stay and postoperative hospital stay, and occurrence of  adverse events during admission between the 2 groups |
| - **Cell Salvage** | | | | | | | | | | | | |
| Aghdaii 2012^1433^ | | - Iran - English - 2012 - Single-Centre - 50 - The inclusion criteria were as follows: primary, elective, on -pump CABG surgery; age between 30 and 70 years; left ventricular ejection fraction ≥45%, pump time | | The exclusion criteria were: patients with known coagulation disorders; redo or emergency surgery; patients on Warfarin, heparin, or other systemic anticoagulant drugs and antiplatelet drugs such as Aspirin (the patients either did not take Aspirin or took a maximum dose of 80 mg/day) preoperatively; and co -existing diseases (renal and hepatic disease diabetes mellitus, hypertension, and endocrine and haematology disorders) .B | | 55(14)  Vs  58(5.4) | CV disease | | Cardiac surgery | - Cell Salvage - Non Cell Salvage Transfusion - - | - | Volumes of the intraoperative autologous and homologous transfusion, activated clotting time (ACT) of the transfused bloods, and ACT and amount of blood loss in the patients were measured intra and postoperatively. |
| Blatsoukas 2010^1434^ | | - Greece - English - 2010 - Single-Centre - 248 - Patients undergoing unilateral TKR for knee osteoarthritis | | Exclusion criteria were patients on anticoagulation therapy, with rheumatoid or seronegative arthritis, blood dyscrasia, malignancy or immunocompromised disease | | 69.4(6.5)  Vs  68.5(7.3)  Vs  69.1(7.2) | - | | Orthopaedic surgery | - Intra+Post Cell Salvage - Non Cell Salvage Transfusion - Post-operative Auto-transfusion - - | - | Patients demographic and clinical data including age, gender, body mass index (BMI), preoperative Hb value, operation time, side of operation, the need of ABT, reinfusion blood volume (IAT and PAT), blood loss, side effects, complications, and postoperative Hb levels on post-operative days 1, 2, 3, and 7 were documented. |
| Campbell 2012^1435^ | | - UK - English - 2012 - Single-Centre - 20 - Patients undergoing CABG | | Patients older than 70 years of age, those with a known clotting deficiency, those taking warfarin or antiplatelet drugs within 5 days of surgery, or those who had a pre-operative platelet count | | 62(10)  Vs  60(10) | CV disease | | Cardiac surgery | - Intra+Post Cell Salvage - Control - - | thrombelastometric parameters, platelet count after surgery and the amount of blood present in chest drains in the first 4 hours. | INTEM (ellagic acid activated intrinsic pathway) clotting time, clot formation time and maximum clot firmness and FIBTEM (tissue factor-triggered extrinsic pathway with platelet inhibitor) maximum clot firmness were measured by Rotem® (Pentapharm, Munich, Germany) thrombelastometry |
| Cholette 2013^1436^ | | - USA - English - 2013 - Single-Centre - 106 - Children ≤ 20 kg presenting to the University of Rochester Medical Centre (URMC) for cardiac surgical repair/palliation with CPB | | Weight > 21 kg, if their parent/guardian did not speak English, or if consent could not be obtained. | | 0.25(1.75)  Vs  0.4(1.5) | CV disease | | Paediatric Cardiac Surgery/ICU | - Cell Salvage - Control - Restrictive threshold | - | Number of RBC and component blood product transfusions, donor exposures, and volume of crystalloid/colloid administered were recorded. Length of mechanical ventilation, vasoactive agents, PCICU and hospital length of stay was followed. Infections (based on clinical and culture data), bleeding complications and thrombosis (based on clinical and radiographic data) were recorded. Mediastinal tube drainage, Hb, platelet and coagulant protein levels were also followed. |
| Cip 2013^1437^ | | - Austria - English - 2013 - Single-Centre - 140 - Patients treated with primary elective TKA for osteoarthritis from December 2007 to January 2009 | | Patients not willing to take part in the study or receiving revision arthroplasty | | 70(8)  Vs  69(8) | - | | Orthopaedic surgery | - Cell Salvage - Control - - | - | demographic data, medical history (coronary artery disease, use of anticoagulants, and American Society of Anaesthesiologists [ASA] classification [13]), preoperative and postoperative haemoglobin levels, duration of surgery, need for ABT, amount of re-transfused WSB, and early complications (including allergic reactions, wound infections, minor and major bleeding, deep venous thrombosis, nerve injuries, pulmonary embolism) at the preoperative examination and during the hospital stay. |
| Clagett 1999^1438^ | | - USA - English - 1999 - Single-Centre - 100 - Patients undergoing elective AAA repair or AFB for occlusive disease | | Patients undergoing Thoraco-abdominal or suprarenal aneurysm repair, concomitant renal or visceral artery reconstruction, and re-operative aortic operations; those with congenital or acquired bleeding disorders, creatinine levels higher than 3 mg/dL, significant pre-existing anaemia (haemoglobin level [Hgb] less than 10 g/dL), cirrhosis, and liver failure; those undergoing an emergency operation; and those who refused to join the study. | | 63(11)  Vs  65(9.0) | CV disease  Renal disease | | Vascular Surgery | - Intra Cell Salvage - Normal Drainage - - | total amount of allogeneic blood transfusion per patient during the period of hospitalization and the proportion of patients in whom allogeneic blood was not transfused. | Hematologic parameters, fluid and colloid requirements, morbidity, and mortality. |
| Dalrymple-Hay 1999^1439^ | | - UK - English - 1999 - Single-Centre - 112 - patients undergoing either coronary artery - bypass grafting, valve replacement/repair operations or a combination of the two | | Patients with previous cardiac surgery, emergency operations, patients anticoagulated with warfarin and Jehovah Witness patients. | | 67.4(9.0)  Vs  65.3(10.5) | CV disease | | Cardiac Surgery | - Post Cell Salvage - Normal Drainage - - | - | Amount of allogeneic blood transfused. Number of patients transfused allogeneic blood. Mortality. Reoperation for bleeding. Blood loss. Coagulopathy. |
| Damgaard 2010^1440^ | | - Denmark - English - 2010 - Single-Centre - 29 - Patient undergoing CABG | | Off-pump, redo or valve operations, current infection or antibiotic treatment, s-creatinine concentration exceeding 200 mol/L, liver disease, immune disease, and anti-inflammatory or immune-modulating treatment, except for nonsteroidal anti-inflammatory drugs and aspirin | | 66(14)  Vs  68(14) | CV disease | | Cardiac Surgery | - Intra+Post Cell Salvage - Normal Drainage - Tranexamic acid | Patient plasma concentrations of IL-6 at 6, 24, and 72 hours after end of CPB. | plasma concentrations of IL-1b, IL-8, IL-10, IL-12, TNF-, sTNF-RI, sTNF-RII, and Procalcitonin at the same intervals; bleeding, allogenic transfusions, cell saver effectiveness regarding inflammatory marker reduction, and complications. |
| Dietrich 1989^1441^ | | - Germany - English - 1989 - Single-Centre - 100 - Patients undergoing aorto-coronary bypass | | Not-stated | | 54.1(6.8)  Vs  56(6) | CV disease | | Cardiac Surgery | - Cell Salvage - Re-transfusion of oxygenator blood - Predonation - Pre-donation +Cell separator - - | - | Amount of blood re-transfused from the cell saver. Amount of allogeneic blood transfused. Number of patients transfused allogeneic blood. Complications. Mortality. ICU length of stay. Blood loss. Re-exploration for bleeding. Operation time. Haematological variables. Hct levels. |
| Elawad 1991^1442^ | | - Sweden - English - 1991 - Single-Centre - 40 - Patients undergoing primary hip arthroplasty | | Not stated | | 68(7.5)  Vs  74(10.2) | - | | Orthopaedic Surgery | - Post Cell Salvage - Control Group - - | - | Amount of allogeneic units transfused. Number of patients receiving allogeneic blood. Complications. Blood loss. Haematological variables. |
| Hogan 2015^505^ | | - United Kingdom - English - 2015 - Single-Centre - 53 - Patient undergoing elective or urgent CABG or valve surgery or both utilizing CPB | | Emergency surgery, a contra-indication to either heparin, protamine or tranexamic acid, or inability to understand the study protocol. | | 67.7(10.2)  Vs  70.5(10.2) | CV disease | | Cardiac surgery | - Post Cell Salvage - Non Cell Salvage Transfusion - Tranexamic acid | Haemoglobin concentration after auto-transfusion | Red cell or blood product transfusions, total fluid administration or blood loss in the first 12 h, and ICU length of stay. |
| Horstmann 2013^1443^ | | - Netherlands - English - 2013 - Single-Centre - 204 - Total hip arthroplasty patients | | Coagulation disorders including deep venous thrombosis and pulmonary embolism, malignancy, ongoing infections, untreated hypertension, unstable angina pectoris, myocardial infarction within the past 12 months, coronary bypass operation within the past 12 months, intake of anticoagulants or participation in other clinical trials dealing with any drugs that affect blood loss. | | 67.3(9.3)  Vs  67.6(9.4) | - | | Orthopaedic | - Intra+Post Cell Salvage - Control - - | Hb level on the first postoperative day | Hb levels on the day of surgery, the second and third days, the lowest post-operative level, any HBT requirement, adverse events, and total blood loss. |
| Horstmann 2014^1444^ | | - Netherlands - English - 2014 - Single-Centre - 118 - Patients undergoing primary total hip arthroplasty | | coagulation disorders, including deep venous thrombosis and pulmonary embolism; malignancy; ongoing infections; untreated hypertension; unstable angina pectoris; myocardial infarction within the past 12months; coronary bypass surgery within the past 12 months; renal dysfunction; anticoagulant intake or participation in other clinical trials dealing with any drugs that affect blood loss. | | 67.6(9.1)  Vs  69.3(9.5) | - | | Orthopaedic surgery | - Post Cell Salvage - Normal Drainage - - | Hb level on the first postoperative day | Hb levels on the second and third postoperative  days, the lowest postoperative Hb level, blood loss during surgery, volume of intraoperatively suctioned and re-transfused blood, volume of re-transfused drained wound blood, allogeneic blood transfusions, postoperative pain, hospital stay, adverse events and total blood loss. |
| Klein 2008^1445^ | | - UK - English - 2008 - Single-Centre - 213 - Nonemergency first time CABG, valve surgery or combined CABG, and valve procedures requiring cardiopulmonary bypass (CPB) | | Patient refusal to receive blood or blood products; previous cardiac or thoracic surgery; known coagulation disorders; contraindication to antifibrinolytic; participation in another trial of an investigational drug or device; or specific request for cell salvage by the operating surgeon. Operations associated with a high risk of transfusion, such as transplantation and operations on the thoracic aorta were excluded | | 68.6(9.6)  Vs  67.4(10.2) | CV disease | | Cardiac Surgery | - Cell Salvage - Control Group - Tranexamic acid | Any allogeneic blood transfusion. | The number of units of RBCs, FFP, or platelets transfused. Serious adverse events, hematology, and biochemistry variables (sampled preoperatively and at 1 h, 24 h, and 5 days after operation) were recorded to monitor safety. |
| Koopman 1993a^1446^ | | - Netherlands - English - 1993 - Single-Centre - 40 - - | | - | | 64(7)  Vs  62(10) | CV disease | | Cardiac Surgery | - Cell Salvage - Control Group | - | Amount of blood collected by the cell saver. Amount of blood re-transfused from the cell save. Amount of allogeneic blood transfused. Number of patients transfused allogeneic blood. Complications. Blood loss. Hb & Hct levels. |
| Koopman 1993b^1446^ | | - Netherlands - English - 1993 - Single-Centre - 60 | | - | | 51(18)  Vs  53(18) | - | | Orthopaedic Surgery | - Cell Salvage - Control Group | - | Amount of blood collected by the cell saver. Amount of blood re-transfused from the cell save. Amount of allogeneic blood transfused. Number of patients transfused allogeneic blood. Complications. Blood loss. Hb & Hct levels. |
| Laub 1993^1447^ | | - USA - English - 1993 - Single-Centre - 38 - Patients undergoing primary coronary revascularization between July and December 1989 | | Not stated | | 65(10.5)  Vs  64.4(9.2) | CV disease | Cardiac Surgery | | - Cell Salvage - Control Group - - | - | Amount of blood re-transfused from the cell saver. Number of patients transfused allogeneic blood. Amount of allogeneic blood transfused. Amount of any blood product transfused. |
| Liang 2014^1448^ | | - China - English - 2014 - Single-Centre - 110 - scoliosis patients undergoing posterior instrumented spinal fusion between January 2012 and June 2013 at a single hospital | | Scoliosis patients who underwent osteotomy, growing rod extending or revision surgery, with a history of a bleeding disorder, a low platelet count (<150,000), abnormal partial thromboplastin time or international ratio test, previous thromboembolic event, or a family history of thromboembolism | | 15.5(5.6)  Vs  16.8±(6.9) | - | Orthopaedic surgery | | - Intra Cell Salvage - Normal Drainage - Iron Therapy - Restrictive Threshold | - | Perioperative haemoglobin levels, surgical time, levels fused, perioperative estimated blood loss, perioperative transfusions and incidence of transfusion-related complications. |
| Marberg 2010^1449^ | | - Sweden - English - 2010 - Single-Centre - 77 - Elective CABG patients | | Known liver, kidney or bleeding disorder, perioperative use of Aprotinin or Clopidogrel treatment within 5 days before surgery. | | 66(10)  Vs  68(8) | CV disease | Cardiac Surgery | | - Post Cell Salvage - Normal Drainage - Tranexamic acid | Bleeding during the first 12 postoperative hours. | Postoperative transfusion requirements, haemoglobin levels, thrombo-elastometric variables and plasma concentrations of interleukin-6, thrombin—anti-thrombin complex and D-dimer. R |
| McGill 2002^1450^ | | - USA - English - 2002 - Single-Centre - 168 - Age 18­80 years Ejection fraction > 30%, Serum creatinine concentration < 150 umol/l, International normalised ratio and activated partial, thromboplastin time < 1.5, Platelet count > 150 × 10^9/l, Haemoglobin concentration > 120 g/l, Haematocrit > 0.36, Weight > 60 kg | | Emergency operation  Redo procedures and multiple procedures  Known carotid stenosis > 50%  Myocardial infarction in past three weeks  Heparin or warfarin taken in previous five days  Antiplatelet treatment other than aspirin  Cerebrovascular disease  History of liver disease  Jehovah's Witnesses | | 63.8(7.8)  Vs  63.1(8.2)  Vs  63.4(9.1) | CV disease | Cardiac Surgery | | - Cell salvage - Cell salvage+normovolaemic haemodilution - Control Group - Tranexamic acid | - | Number of patients transfused allogeneic blood. Number of patients receiving any blood product. Amount of allogeneic blood transfused. Blood loss. Re-operation for bleeding. Hospital length of stay. Infection. Stroke. Renal failure. Myocardial infarction. |
| Menges 1992^1451^ | | - German - German - 1992 - Single-Centre - 26 - Requires Translation | | Requires Translation | | 55.9(18.2)  Vs  66.7(12.7) | - | Orthopaedic Surgery | | - Cell salvage - Control Group - Tranexamic acid | - | Amount of blood re-transfused from the cell saver. Number of patients transfused allogeneic blood. Blood loss. Hb & Hct levels. Clotting status (PT/TT/PTT/ATIII). Immunological methods. |
| Mercer 2004^1452^ | | - UK - English - 2004 - Single-Centre - 81 - Patients undergoing elective repair of infra-renal AAA | | Not stated | | 72(5.1)  Vs  73(8.1) | CV disease  Renal disease | Vascular Surgery | | - Intra Cell Salvage - Control Group - - | incidence of systemic inflammatory response syndrome (SIRS) | requirement for homologous blood transfusion and postoperative infection |
| Murphy 2004^1453^ | | - UK - English - 2004 - Single-Centre - 196 - Patients aged 18 or over who were undergoing nonemergency first time coronary artery bypass grafting | | Patients who are prevented from utilizing blood and blood products according to a system of beliefs (e.g., Jehovah’s Witnesses), patients o warfarin, heparin, or  other systemic anticoagulant drugs preoperatively, patients  with congenital or acquired platelet, red cell, or clotting disorders, patients with ongoing or recurrent systemic sepsis and patients who were unable to give full  informed consent for the study | | 64.3(9.2)  Vs  62.3(18.7) | CV disease | Cardiac Surgery | | - Cell salvage - Control Group - POC testing | - | intraoperative homologous blood transfusion, Hb concentration and haematocrit measurements, platelet count, prothrombin time, activated partial thromboplastin time, fibrinogen concentration, D-dimer concentration, and thromboelastography |
| Murphy 2005^1454^ | | - UK - English - 2005 - Single-Centre - 61 - Patients aged 18 years or more and who were undergoing nonemergency first-time CABG | | Patients who are prevented from receiving blood and blood products according to a system of beliefs (eg, Jehovah  Witnesses); patients receiving preoperative warfarin, heparin, or  other systemic anticoagulant drugs; patients with congenital or acquired platelet, red blood cell, or clotting disorders; patients with  ongoing or recurrent systemic sepsis; and patients who were unable to give full informed consent for the study | | 62.3(9.3)  Vs  66.4(7.6) | CV disease | Cardiac Surgery | | - Cell salvage - Control Group - POC testing | - | 24-hour postoperative haemoglobin concentration, frequency of homologous blood product use, platelet count, prothrombin time, activated partial thromboplastin time, fibrinogen concentration, D-dimer concentration, and thromboelastography |
| Naumenko 2003^1455^ | | - Russia - Russian - 2003 - Single-Centre - 66 - coronary bypass for IHD under conditions of extracorporeal circulation | | - | | - | CV disease | Cardiac surgery | | - Post Cell Salvage - Normal Drainage - - | - | Number of patients transfused allogeneic blood. |
| Parrot 1991^1456^ | | - France - English - 1991 - Single-Centre - 44 - Patients undergoing aortocoronary bypass surgery | | Emergency patients, patients with an intra-aortic balloon pump or preoperative haematocrit less than 3S%, and re-operative patients were not included in this study. | | 60(7)  Vs  61(7) | CV disease | Cardiac surgery | | - Intra Cell Salvage - Control - - | - | Amount of blood re-transfused from the cell saver. Amount of allogeneic blood transfused. Number of patients transfused allogeneic blood. Complications. Mortality. Blood loss. Hct levels. |
| Reyes 2010^1457^ | | - Spain - English - 2010 - Single-Centre - 63 - Patients undergoing coronary or valve procedure | | Combined procedure, aorta procedure, redo surgery, emergency procedures, creatinine levels of 2mg/ml, anaemic patients and patients with body surface area (BSA) 1.6m2 | | 65.5(12.1)  Vs  63.7(12.7) | CV disease | Cardiac surgery | | - Cell Salvage - Normal Drainage - Tranexamic acid - Restrictive Threshold | - | Need of blood products and clinical outcomes |
| Rollo 1995^1458^ | | - US - English - 1995 - Single-Centre Quasi-randomised by age - 73 - Patients undergoing primary uncemented THAs | | Patients were excluded from the study if they had a history of a bleeding disorder, infection, carcinoma, or previous surgery involving the operative hip. | | 68(9)  Vs  68(14)  Vs  64(11)  Vs  61(12) | - | Orthopaedic surgery | | - Cell Salvage - Re-infusion - Auto-transfusion - Normal Drainage - - | - | Amount of allogeneic and/or autologous blood transfused. Number of patients transfused allogeneic blood. Complications. Hb & Hct levels. Thigh circumference measures. Wound drainage. |
| Sarkanovic 2013^1459^ | | - Serbia - English - 2013 - Single-Centre - 112 - Patients undergoing TKR surgery in a 3-months period during 2010. | | patients with  septic complications, multiple fractures, malignancy, ASA physical status classification IV or more, hemi-arthroplasty and all patients  with incomplete data | | 65.2(7.7)  Vs  67.7(7.2) | - | Orthopaedic surgery | | - Cell Salvage - Normal Drainage - - | - | transfusion of allogeneic blood, length of hospital stay |
| Savvidou 2009^1460^ | | - Greece - English - 2009 - Single-Centre - 50 - Patients for posterolateral fusion with internal fixation | | Not stated | | 67.7(7.2)  Vs  61(13.5) | - | Orthopaedic surgery | | - Post Cell Salvage - Non Cell Salvage Transfusion - Restrictive Threshold | - | Surgical time, intraoperative blood loss, haemoglobin and haematocrit levels preoperatively and at discharge were recorded. Intraoperative blood loss was measured by the drain output of the surgical field. |
| Scrascia 2012^1461^ | | - Italy - English - 2012 - Single-Centre - 34 - Patients undergoing first-time, elective, isolated CABG | | Patients aged >80 years old, preoperative haemoglobin (Hb) <12 g/dL, body surface area  (BSA) <1.7 m2, redo or emergency surgery, valvular, thoracic aorta or combined procedures, liver insufficiency (Child Pugh B or C class), platelet count below  50,000 or antiplatelet treatment taken within 5 days before surgery, pre-existing haemolytic or haemostatic disorders, anticoagulant treatment, inflammatory disorders or steroids treatment. | | 71(8)  Vs  66(10) | CV disease | Cardiac surgery | | - Cell Salvage - Normal Drainage - Tranexamic acid | The influence of CPB circuit residual blood salvage infusion after cell saving treatment on inflammatory, coagulative and fibrinolytic system activation, measuring specific parameters. | The influence of pump blood salvage on postoperative haemoglobin levels and transfusion rate. |
| Shen 2016^1462^ | | - China - English - 2016 - Single-Centre - 103 - High bleeding risk undergoing cardiac surgery with CPB | | Emergency cardiac surgery with CPB  The first time single valve replacement | | 50.4(15.4)  Vs  52.5(15.6) | CV disease  Anaemia | Cardiac surgery | | - Intra+Post Cell Salvage - Normal Drainage - Tranexamic acid - POC testing - Restrictive threshold | The incidence of  impairment of blood coagulation during perioperative  period (peri-op) | The incidence of adverse events during postoperative  period (post-op) |
| Shenolikar 1997^1463^ | | - UK - English - 1997 - Single-Centre - 100 - patients with a preoperative haemoglobin>11 g /dL, scheduled for knee replacement surgery | | Not stated | | 69.6(7.4)  Vs  69.3(9.1) | - | Orthopaedic surgery | | - Post Cell Salvage - Control - - | - | Amount of blood collected by the cell saver. Amount of allogeneic blood transfused. Number of patients transfused allogeneic blood. Complications. Hospital length of stay. |
| Slagis 1991^1464^ | | - USA - English - 1991 - Single-Centre - 102 - Patients undergoing hip or knee arthroplasty at the University of Arizona Medical Centre between August 1, 1988 and June 1, 1989. | | Patients who needed transfusion pre-operatively and those who had refused to participate. | | 70  Vs  70 | - | Orthopaedic surgery | | - Intra+Post Cell Salvage - Normal Drainage - - | - | Amount of blood collected by the cell saver. Amount of blood re-transfused from the cell saver. Amount of allogeneic blood transfused. Number of patients transfused allogeneic blood. Complications. Coagulopathy. Blood loss. Transfusion reactions. |
| So-Osman 2014^1465^ | | - Germany - English - 2014 - Single-Centre - 1759 - Adult elective hip-and knee surgery patients | | Hb (haemoglobin)  less than 13 g/dl, untreated hypertension (diastolic blood  pressure >95 mmHg); a serious disorder of the coronary,  peripheral, and/or carotid arteries; a recent myocardial infarction or stroke (within 6 months); sickle cell anaemia; a malignancy in the surgical area; a contraindication for anticoagulation prophylaxis; an infected wound bed; a revision of an infected prosthesis, which was being treated with local antibiotics difficulty understanding the Dutch language (unable to give informed consent); or were pregnant or refused homologous blood transfusions. | | 69(10)  Vs  68(10) | - | Orthopaedic surgery | | - Intra+Post Cell Salvage - Normal Drainage - Restrictive threshold | RBC use | Cost effectiveness, in which length of hospital stay was included. |
| Spark 1997^1466^ | | - UK - English - 1997 - Single-Centre - 50 - Patients undergoing elective infra-renal abdominal aortic aneurysm repair. | | - | | 71(6)  Vs  68(7) | - | Vascular surgery | | - Intra Cell Salvage - Control - - | - | Amount of allogeneic blood transfused. Number of patients transfused allogeneic blood. Complications. Hospital length of stay. Blood loss. Mortality. |
| Tempe 1996^1467^ | | - India - English - 1996 - Single-Centre - 100 - Patients undergoing elective valve surgery, using cardiopulmonary bypass (CPB) | | Patients having a re-operation or preoperative coagulation abnormalities were excluded | | 29.1(11.8)  Vs  26.1(9.3) | CV disease | Cardiac surgery | | - Intra+Post Cell Salvage - Control - Iron therapy | - | Amount of allogeneic blood transfused. Number of patients transfused allogeneic blood. Complications. Re-exploration for bleeding. Chest drainage. Hct levels. |
| Tempe 2001^1468^ | | - India - English - 2001 - Single-Centre - 40 - Patients scheduled for elective primary valve surgery | | - | | 27.7(10.1)  Vs  26.6(7.35) | CV disease  Anaemia | Cardiac surgery | | - Cell Salvage - Control - Iron therapy | - | Amount of allogeneic blood transfused. Re-exploration for bleeding. |
| Thomas 2001^1469^ | | - UK - English - 2001 - Single-Centre - 231 - Patients undergoing TKR | | Not stated | | - | - | Orthopaedic surgery | | - Post Cell Salvage - Control - - | - | Number of patients transfused allogeneic blood. Amount of allogeneic blood transfused. Complications. |
| Thomassen 2012^1470^ | | - Netherlands - English - 2012 - Multi-Centre - 216 - Patients receiving primary or revision total hip arthroplasty with ASA I, II, or II | | -Exclusion due to ethical concern included previous randomization  in this study, involvement in the planning and/or conduct of this study, and participation in an interfering study.  – Exclusion due to safety concerns included current symptoms of haemophilia and contraindications for autologous blood use, i.e. hyperkalaemia, current systemic infection or local infection in the operation field or impaired renal function, known malignancy in the last five years and expected use of cytotoxic drugs.  – Exclusion due to expected impact on outcome included  untreated anaemia (haemoglobin (Hb) level <11 g/dL), revision total hip arthroplasties with expected serious bone grafting, and use of other alternatives for blood conservation such as recombinant erythropoietin, fibrin sealant, Aprotinin and other autologous blood transfusion. | | 67(11)  Vs  65(12) | - | Orthopaedic surgery | | - Post Cell Salvage - Control - Tranexamic acid | allogeneic blood transfusion frequency | blood loss, postoperative haemoglobin/haematocrit, safety and quality of life Perioperative blood loss |
| Vermeijden 2015^1471^ | | - Netherlands - English - 2015 - Multi-Centre - 366 - Patients undergoing elective coronary, valve, or combined surgical procedures | | Patients scheduled for off-pump surgery and patients with known coagulation disorders except after the use of aspirin, Clopidogrel, or low molecular-weight heparin | | 66(9.5)  Vs  66(9.7) | CV disease | Cardiac surgery | | - Cell Salvage - Normal Drainage - Tranexamic acid - Restrictive threshold | The number of allogeneic blood products transfused in each group during hospital admission. | Percentage of patients who received any allogeneic blood products, number of re-explorations, myocardial infarction, stroke, postoperative ventilation time, length of stay in the intensive care unit and in the hospital, and 1-year mortality. |
| Wiefferink 2007^1472^ | | - Netherlands - English - 2007 - Single-Centre - 30 - Adult patients, undergoing isolated primary elective myocardial re-vascularization | | Not stated | | 62(11)  Vs  66(8) | CV disease | Cardiac surgery | | - Post Cell Salvage - Control - - | - | The volume of the chest tube drainage was noted 2 hours after arrival at the ICU, and the transfusion requirements were noted during the entire ICU period. |
| Xie 2015^1473^ | | - China - English - 2015 - Single-Centre - 141 - 3 inclusion criteria that should be satisfied at the same time: firstly, patients were scheduled for cardiac surgery with CPB; secondly, surgery was combined aortic valve replacement and mitral valve replacement, or Bentall, or reoperation; thirdly, at least two of the following conditions are satisfied: age >70 years; body surface area (BSA)<1.6 m2; renal dysfunction (creatinine >15mg/L ); liver insufficiency (Child -Pugh B or C); coagulation disorders (thromboelastography, TEG, R value before surgery >10 min); haemoglobin(HB) levels < 130 g L-1 in males or <120 g L-1 in females; Platelets (PLT) count <50 ×10^9 L-1; intake of aspirin 3 days before surgery or Clopidogrel 7 days before surgery | | Not stated | | 51.7(15.6)  Vs  53.1(15.1) | CV disease  Anaemia | Cardiac surgery | | - Intra+Post Cell Salvage - Normal Drainage - Tranexamic acid - POC testing - Restrictive Threshold | - | Perioperative allogeneic red blood cell (RBC) transfusion, perioperative impairment of blood coagulative function, postoperative adverse events and costs of transfusion-related. |
| Zhang 2008^1474^ | | - China - Chinese - 2008 - Single-Centre - 40 - Patients undergoing selective orthopaedic surgery | | - | | - | - | Orthopaedic surgery | | - Intra Cell Salvage - Control - - | - | Risk of RBC transfusion |
| Zhao 2017^1475^ | | - China - English - 2017 - Single-Centre - 120 - Patients undergoing off-pump coronary artery bypass operations. | | - | | 60.4(9.2)  Vs  59.2(7.4) | CV disease | Cardiac surgery | | - Cell Salvage - Non Cell Salvage Transfusion - - | - | all adverse reactions, such as haemoglobin urine, allergic reactions, and coagulation abnormalities, autologous blood transfusion volume and allogeneic blood transfusion volume were also recorded. One day after the operation, routine blood tests and biochemistry were performed; ICU retention time and complications were recorded. |
| - ***Restrictive vs. Liberal*** | | | | | | | | | | | | |
| Bracey 1999^1476^ | | - USA - English - 1999 - Single-Centre - 428 - Patients who underwent first time, elective CABG surgery - Restrictive threshold 8g/dl | | Patient exclusion criteria included a preoperative Hb level 2500 mL within 24 hours of operation, and the patient’s refusal of blood transfusion for religious reasons. | | 61(11)  Vs  62(11) | CV disease | Cardiac Surgery | | - Restrictive 80g/L - Liberal - - | - | Mortality, length of hospital stay, blood usage (units), blood loss, complications, infection  rates, cardiac events |
| Bush 1997^1477^ | | - USA - English - 1997 - Single-Centre - 99 - Patients undergoing elective aortic or infra inguinal arterial reconstructions - Restrictive threshold 9g/dl | | Patients were excluded from participation if they refused blood transfusions for religious or other reasons, did not speak English, or had had a myocardial infarction within 3 months preceding the scheduled operation. | | 64(11)  Vs  66(10) | CV disease | Vascular surgery | | - Restrictive 90g/L - Liberal - - | myocardial ischaemia, myocardial infarction, and death | Length of intensive care unit stay, hospital stay, and graft  patency |
| Carson 1998^1478^ | | - USA - English - 1998 - Single-Centre - 84 - Patients were eligible for the trial if their Hb levels were less than 10 g per dL in the immediate postoperative period, defined as the time from the end of anaesthesia in the operating room to 11:59 PM 3 days after surgery (counted from 12:OO midnight on the first day after surgery) - Restrictive threshold 8g/dl | | Patients who refused transfusion because of religious beliefs, suffered multiple trauma (defined as any in- jury that required surgical repair in addition to the hip fracture), or had symptoms of anaemia were excluded from the trial. | | 81.3(8.1)  Vs  83.3(10.8) | CV disease  Anaemia | Orthopaedic Surgery | | - Restrictive 80g/L - Liberal - - | - | Mortality, length of hospital stay, blood usage (units), complications, pneumonia, stroke,  thromboembolism |
| Carson 2011^1479^ | | - USA - English - 2011 - Multi-Centre - 2016 - Patients 50 years of age or older who were undergoing primary surgical repair of a hip fracture and who had clinical evidence of or risk factors for cardiovascular disease were eligible if they had a haemoglobin level of less than 10 g per decilitre within 3 days after surgery. According to the original protocol, only patients with cardiovascular disease (a history of ischemic heart disease, electrocardiographic evidence of previous myocardial infarction, a history or presence of congestive heart failure or peripheral vascular disease, or a history of stroke or transient ischemic attack) were eligible. - Restrictive threshold 8g/dl | | Patients were excluded if they were unable to walk without human assistance before hip fracture, declined blood transfusions, had multiple trauma (defined as having had or planning to undergo surgery for non–hip-related traumatic injury), had a pathologic hip fracture associated with cancer, had a history of clinically recognized acute myocardial infarction within 30 days before randomization, had previously participated in the trial with a contralateral hip fracture, had symptoms associated with anaemia (e.g., ischemic chest pain), or were actively bleeding at the time of potential randomization. | | 81.8(8.8)  Vs  81.5(9) | CV disease  Renal disease  Anaemia | Orthopaedic Surgery | | - Restrictive 80g/L - Liberal - - | inability to walk 10 feet (or across a room) without human  assistance or death prior to closure of the window for 60-day mortality | Hb concentration, acute coronary syndrome (ACS), in-hospital myocardial infarction,  unstable angina or death, disposition on discharge, survival, functional measures, fatigue/energy, readmission to hospital, pneumonia, wound infection, thromboembolism,  stroke or transient ischaemic attack, cognition (Gruber-Baldini), mortality at 30 days, and long-term mortality |
| De Almeida 2015^1480^ | | - Brazil - English - 2015 - Single-Centre - 198 - All adult patients who had a major surgical procedure for abdominal cancer and required postoperative care in the ICU because of physiological instability and had an expected ICU stay of more than 24 h were included. - Restrictive threshold 7g/dl | | Patients with the following characteristics: age less than 18 yr, haematological malignancy, a Karnofsky score less than 50, pre-existing anaemia (defined as a preoperative haemoglobin concentration <9 g/dl), pre-existing thrombocytopenia (defined as a platelet count <50,000/mm3), pre-existing coagulopathy (defined as a prothrombin time >14.8 s) or anticoagulation therapy, active or uncontrolled bleeding, expected death within 24 h of ICU admission, end-stage renal failure requiring renal replacement therapy, pregnancy, a do-not-resuscitate order, inability to receive transfusion of blood components, or refusal to participate in the study. | | 64(14)  Vs  64(12) | Cancer  Renal disease | Oncology surgery | | - Restrictive 70g/L - Liberal - - | Composite of all-cause mortality or severe clinical complications within 30 days. | Major cardiovascular  complications, septic shock, acute kidney injury requiring renal replacement therapy,  ARDS, and reoperation |
| Fan 2014^1481^ | | - China - English - 2014 - Single-Centre - 186 - Consecutively admitted patients, with the age of more than 65 years, undergoing elective unilateral total hip replacement from October, 2011 to May 2013 were enrolled in the present study. - Restrictive threshold 8g/dl | | The exclusion criteria were as follows: ASA physical status ≧ IV; preoperative delirium; unwilling to comply with the procedures; inability to understand the language (Mandarin Chinese); hearing loss, or a failure in spinal anaesthesia. | | 73(7)  Vs  75(6) | Renal disease | Orthopaedic Surgery | | - Restrictive 80g/L - Liberal - - | - | Delirium, cerebrovascular accident, cardiac failure, myocardial infarction, pulmonary embolism, pneumonia, superficial wound infection, urinary tract infection, acute renal  failure |
| Foss 2009^1482^ | | - Denmark - English - 2009 - Single-Centre - 120 - Inclusion criteria were primary hip fracture occurring in the community in patients older than 65 years of age with an independent pre-fracture walking function, community dwelling, and intact cognitive status. - Threshold 8g/dl | | Patients with multiple fractures, pre-fracture terminal condition, alcoholism, chronic transfusion needs, acute cardiac or other acute severe medical conditions, or contraindication to epidural analgesia were excluded. | | 81(6.8)  Vs  81(7.3) | CV disease  Renal disease | Orthopaedic Surgery | | - Restrictive 80g/L - Liberal - - | - | Ambulatory capacity, mortality, length of stay, cardiac complications, infectious complications |
| Gregersen 2015^1483^ | | - Denmark - English - 2015 - Single-Centre - 284 - Patients (aged ≥ 65 years) admitted from nursing homes or sheltered housing facilities for unilateral hip fracture surgery and with postoperative Hb levels between 9.7 g/dL (6 mmol/L) and 11.3 g/dL (7 mmol/L) during the first 6 postoperative days. - Restrictive threshold 9.7g/dl | | Exclusion criteria were: active cancer, pathological fractures, and inability to understand or speak Danish without an interpreter, refusal of RBC transfusion (e.g. Jehovah’s Witness), fluid overload, irregular erythrocyte antibodies, or previous participation in the trial. | | 86(6.8)  Vs  88(6.9) | CV disease  Anaemia | Orthopaedic Surgery | | - Restrictive 97g/L - Liberal - - | recovery from physical disabilities | total number of infections (pneumonia, urinary tract infection, other), cognition, depression, quality of life, modified Barthels index, and comprehensive frailty index |
| Grover 2006^1484^ | | - UK - English - 2006 - Multi-Centre - 260 - Patients undergoing elective hip and knee replacement surgery - Restrictive threshold 8g/dl | | Exclusion criteria were age < 55 years, digoxin therapy, ECG evidence of conduction defects, ST segment depression, left ventricular hypertrophy or left bundle branch block. Any patient with anaemia was also excluded. | | 71.5(7.6)  Vs  70.7(7.1) | - | Orthopaedic Surgery | | - Restrictive 80g/L - Liberal - - | - | Ischaemic load, blood load, Hb concentration, number of units transfused, length of  hospital stay, adverse events, new infections requiring antibiotic therapy |
| Hajjar 2010^1485^ | | - Belgium - English - 2010 - Single-Centre - 502 - Patients who were undergoing CABG surgery or cardiac valve replacement or repair, alone or in combination. - Restrictive threshold Haematocrit>24% | | Patients were excluded for any of the following reasons: younger than 18 years; surgery without cardiopulmonary bypass; emergency procedure; ascending and descending thoracic aortic procedures; left ventricular aneurysm resection; inability to receive blood products; enrolment in another study; chronic anaemia (preoperative haemoglobin concentration less than 10 g/dL); low platelet count (preoperative platelet count less than 150 ×103/μL); coagulopathy (previous history or prothrombin time longer than 14.8 seconds); pregnancy; neoplasm; endocarditis; congenital heart defect; hepatic dysfunction (total bilirubin value higher than 1.5 mg/dL [to convert to μmol/L, multiply by 17.104]); end-stage renal disease (receiving chronic dialysis therapy); and refusal to consent. | | 60.7(12.5)  Vs  58.6(12.5) | CV disease  Renal disease | Cardiac Surgery | | - Restrictive 80g/L - Liberal - - | 30-day all-cause mortality and severe  morbidity (cardiogenic shock; ARDS or acute renal injury requiring dialysis or  haemofiltration; respiratory, cardiac, neurologic, and infectious complications; inflammatory  complications; bleeding; ICU and hospital lengths of stay, RBC transfusions) | - |
| Johnson 1992^1486^ | | - USA - English - 1992 - Single-Centre - 38 - Autologous blood donors undergoing elective myocardial revascularization. - Restrictive threshold Haematocrit <25% | | - | | 58.2(7.5)  Vs  60.5(6.9) | CV disease | Cardiac Surgery | | - Restrictive 80g/L - Liberal - - | - | Cardiac events, complications, postoperative blood loss, blood use (total units), allogeneic blood use (units), autologous blood use (units), all product blood use (units), number  of participants receiving transfusions, mean cardiac index, mean systemic resistance, exercise capacity, Hct levels, length of ICU stay, length of hospital stay |
| Koch 2017^1487^ | | - USA - English - 2017 - Multi-Centre - 717 - Patients aged 18 years and older scheduled for elective isolated heart valve procedures, coronary artery bypass graft surgery (CABG) with or without valve procedures, and ascending aorta replacement performed on CPB at two centres: Cleveland Clinic (USA) and SAL Hospital (India). - Restrictive threshold Haematocrit <24% | | Not Stated | | 59(15)  Vs  60(13) | CV disease  Renal disease | Cardiac Surgery | | - Restrictive 80g/L - Liberal - - | Composite of postoperative morbidities and mortality. | Lengths of ICU and postoperative hospital stays, number of RBC units transfused, and individual components of the composite. |
| Laine 2017^1488^ | | - Finland - English - 2017 - Single-Centre - 80 - Patients scheduled for elective open-heart surgery - Restrictive threshold 8g/dl | | Any hereditary or acquired haemostatic disorders, any malignancies, and severe chronic kidney disease (glomerular filtration rate o30 mL/min). | | 70.5(1.5)  Vs  64.5(2.2) | CV disease | Cardiac Surgery | | - Restrictive 80g/L - Liberal - Tranexamic acid - POC testing | - | Amount of bleeding during the surgery and postoperatively from the chest tubes, RBC and blood product transfusions, diuresis, and cumulative fluid balance. Patient data during the surgery and intensive care were collected |
| Lotke 1999^1489^ | | - USA - English - 1999 - Single-Centre - 127 - Patients undergoing primary TKA who were able to donate 2 units of blood pre-operatively - Restrictive threshold 9g/dl | | - | | 69.7  Vs  69.7 | - | Orthopaedic Surgery | | - Restrictive 90g/L - Liberal - - | - | Complications, cardiac events,Hb levels, blood usage (units),mental confusion, lethargy,  orthostatic hypotension, number of participants transfused |
| Markatou 2012^1490^ | | - Greece - English - 2012 - Single-Centre - 58 - Patients scheduled for major abdominal surgery - Restrictive threshold 7.7g/dl | | history of bleeding diathesis associated with thrombocytopenia, hereditary haemostatic defects such as haemophilia or chronic anticoagulant administration, refusal of transfusions for religious reasons, ischemic heart disease (unstable angina or myocardial infarction within the last six months), and pre-existing infectious or autoimmune diseases as well use of corticosteroids or immunosuppressive drugs within the last six months | | 58.2(11.7)  Vs  63.4(11.3) | Cancer | General surgery | | - Restrictive 77g/L - Liberal - - | Units  of red blood cells (RBC) per patient and the incidence of transfused patients in each group | Clinical outcome measures, as expressed by time to patient mobilization, time of first liquid and solid food intake and duration of hospital stay. |
| Mazer 2017^1491^ | | - Canada - English - 2017 - Multi-Centre - 4860 - Adults undergoing cardiac surgery who had EUROSCORE I of 6 or more - Restrictive threshold 7.5g/dl | | Patients unable to receive blood products, declined blood products, were involved in a preoperative autologous donation program, were undergoing heart transplantation, were having surgery solely for the insertion of a ventricular assist device, or were pregnant or lactating. | | 72(10)  Vs  72(10) | CV disease  Renal disease | Cardiac Surgery | | - Restrictive 75g/L - Liberal - Tranexamic acid | composite outcome of death from any cause, myocardial infarction, stroke, or new-onset renal failure with dialysis by hospital discharge or by day 28, whichever came first | Red-cell transfusion and other clinical outcomes. |
| Moller 2019^1492^ | | - Denmark - English - 2019 - Single-Centre - 58 - Patients older than 40 years of age, who were referred for elective open infra-renal AAA repair or lower limb bypass (infra-inguinal arterial bypass surgery or femuro-femoral crossover surgery) - Restrictive threshold 8g/dl | | Potential patients were excluded if they refused RBC transfusion, had previous serious adverse reaction with blood products, had previously participated in the TV-trial or if they were unable to understand the benefits and risks of participating. | | 71.3(9.4)  Vs  73.7(7.3) | CV disease | Vascular Surgery | | - Restrictive 80g/L - Liberal - POC | mean postoperative Hb day 0–15 | (1) units of RBCs transfused  (2) randomization rate  (3) proportion of patients with protocol suspensions (4) adherence to haemoglobin concentrations used for transfusion triggers  (5) intraoperative tissue oxygenation as determined by NIRS, and (6) severe adverse events  within 30 days of surgery |
| Murphy 2015^1493^ | | - UK - English - 2015 - Multi-Centre - 2003 - Patients older than 16 years of age who were undergoing non-emergency cardiac surgery. Patients providing written informed consent. Post-operative haemoglobin level below 9.0g/dL or haematocrit below 27 at any stage during patient’s post-operative hospital stay - Restrictive threshold 7.5g/dl | | Patients who are prevented from having blood and blood products according to a system of beliefs. Patients with congenital or acquired platelet, red cell or clotting disorders. Patients with ongoing or recurrent sepsis. Patients with critical limb ischemia. Patients undergoing emergency cardiac surgery. Patients already participating in another interventional research study. Patients unable to give full informed consent for the study. | | 69.9(3.7)  Vs  70.8(3.6) | CV disease | Cardiac Surgery | | - Restrictive 75g/L - Liberal - Tranexamic acid - Cell salvage | Composite of a serious infection (sepsis or wound infection)  or an ischaemic event (permanent stroke, myocardial infarction, infarction of the gut, or  acute kidney injury)within 3months after randomisation. | Units transfused, infection, ischaemic events, acute kidney injury, hospital stay and ICU  stay, and cost |
| Nielsen 2014^1494^ | | - Denmark - English - 2014 - Single-Centre - 66 - Patients were eligible if they were at least 18 years of age and scheduled for elective hip revision surgery. - Restrictive threshold 7.3g/dl | | Exclusion criteria were disseminated cancer or cardiac disease with functional impairment (NYHA class II or above). | | 68(12.4)  Vs  72(10.3) | - | Orthopaedic Surgery | | - Restrictive 73g/L - Liberal - Tranexamic acid | “Time up and go” test (time it takes a patient to stand up, walk three meters, turn around, walk back and sit down again) | Pneumonia, wound infection, gastrointestinal complications, dizziness, hypotension, fatigue, deep  vein thrombosis, and fall |
| Palmieri 2017^1495^ | | - USA - English - 2017 - Multi-Centre - 345 - Admitted to a participating burn centre within 96 hours of injury with a burn injury ≥ 20% TBSA - Restrictive threshold 7-8g/dl | | <18 years of age; pregnant; unable or unwilling to receive blood products; chronically anaemic (haemoglobin <9.0 g/dl one month prior to enrolment); on renal dialysis prior to injury; brain dead, imminent brain death, or a non-survivable burn; experiencing angina or acute myocardial infarction on admission; pre-existing hematologic disease; or closed head injury with Glasgow coma scale <9. | | 41(7.2)  Vs  41(8) | - | Plastic Surgery | | - Restrictive 70-80g/L - Liberal - - | Number of BSIs as defined by the Burn Consensus Conference. | Mortality, number of infectious episodes (urinary tract infections, pneumonia, wound infection), burn ICU LOS, hospital LOS, duration of mechanical ventilation, organ dysfunction (MODS), and time to 90% burn wound healing (defined as 7 days after the last excision and grafting procedure). |
| Parker 2013^1496^ | | - UK - English - 2013 - Single-Centre - 200 - Patients treated at a single centre with a proximal femoral (hip) fracture were considered for inclusion in the study if their haemoglobin measured on the first or second day after surgery was between 8.0 and 9.5 g dl1 and no definite symptoms of anaemia were present. - Restrictive threshold symptoms guided | | Exclusion criteria were age <60 years, patients unwilling or unable to provide written informed consent, multiple trauma (defined as either more than two other fractures), patients treated conservatively, patients treated with percutaneous screw fixation and those with pathological fractures from tumours. | | 84.4(12.7)  Vs  84.2(10.6) | CV disease  Anaemia | Orthopaedic Surgery | | - Restrictive 80g/L - Liberal - - |  | Mobility, mental agility, physical status using the American Society of Anaesthesiologists grade |
| Shehata 2012^1497^ | | - Canada - English - 2012 - Single-Centre - 50 - Eligible participants were adults patients undergoing cardiac surgery with a CARE score (a score for cardiac surgery patients used to predict morbidity and mortality) of 3 or 4 or patients of advanced age defined as greater than or equal to 80 years on the day of screening were included. - Restrictive threshold 7g/dl | | Patients were excluded if they refused participation, were unable to receive or refused blood products, or were involved in the autologous pre-donation program. | | 67.2(11.2)  Vs  68.8(9.2) | CV disease  Renal disease | Cardiac Surgery | | - Restrictive 70g/L - Liberal - Tranexamic acid - Cell Salvage | Enrolment rate and overall adherence to the transfusion strategies. | RBC transfusions, clinical outcomes, and physiologic indicators of hypoxemia (mixed venous oxygen saturation). Clinical outcomes were defined as 1) in-hospital all-cause mortality; SHEHATA ET AL. 92 TRANSFUSION Volume 52, January 2012 2) a composite score of morbidity consisting of a) neurologic events defined as a new focal neurologic deficit lasting more than 24 hours or irreversible encephalopathy, b) dialysis-dependent renal failure or greater than 50% increase in creatinine, c) prolonged low cardiac output state (i.e., need for two or more inotropes for 24 hours or more, intraaortic balloon pump or ventricular assist device for greater than 48 h), and/or myocardial infarction, defined as troponin I level greater than 2.5 mg/L and new Q waves on electrocardiogram or a clinical diagnosis; and 3) hospital lengths of stay |
| So-Osman 2013^1498^ | | - Netherlands - UK - 2013 - 603 - - - Restrictive threshold: most restrictive transfusion policy | | - | | 70.2(10.3)  Vs  70.7(9.6) | CV disease | Orthopaedic Surgery | | - Restrictive (trigger age dependent) - Liberal - - | RBC use | Postoperative complications and quality of life |
| - ***Tranexamic Acid*** | | | | | | | | | | | | |
| Aguilera 2013^1499^ | | - Spain - English - 2013 - Single-Centre - 83 - Adult patients undergoing elective primary total knee arthroplasty from June 2010 to October 2011 | | Patients with an allergy to tranexamic acid or to Aprotinin, a history of coagulopathy or a thromboembolic event, previous vascular or cardiac bypass surgery, treatment with an anticoagulant or contraceptives, presence of a cardiovascular prosthesis, and patients who declined to participate. | | 72.4(6.6)  Vs  74.9(7) | - | Orthopaedic Surgery | | - IV TXA - No TXA - - | Total blood loss collected in drains after surgery | Calculated hidden blood loss, transfusion rate, preoperative and postoperative haemoglobin, number of blood units transfused, adverse events, and mortality. |
| Aguilera 2015^1500^ | | - Spain - English - 2015 - Multi-Centre - 100 - Adult patients undergoing primary total knee arthroplasty | | known allergy to TXA, a history  of coagulopathy or a thromboembolic event, previous bypass surgery, use of anticoagulant or contraceptive treatment, cardiovascular prosthesis, and refusal to participate | | 72.4(7.6)  Vs  73.6(7.3) | Renal disease | Orthopaedic Surgery | | - IV TXA - No TXA - - | Total blood loss | Hidden blood loss, blood collected in drains, transfusion rate, number of blood units transfused, adverse events, and mortality. |
| Ahn 2012^1501^ | | - Korea - English - 2012 - Single-Centre - 76 - Anaemic patients who continued dual antiplatelet therapy until within 5 days of off-pump | | Patients with impaired renal function (serum creatinine [sCr] >20 mg/L), hepatic dysfunction, neurologic dysfunction or hematologic disorders | | 69(7)  Vs  67(7) | CV disease  Anaemia | Cardiac Surgery | | - IV TXA - Placebo - Cell Salvage | Perioperative (combined period of intraoperative and postoperative 24h) transfusion requirement between the groups | Amount of perioperative blood loss between the groups. |
| Albirmawy 2013^1502^ | | - Egypt - English - 2013 - Single-Centre - 400 - Children underwent primary isolated adenoidectomy | | Children who had revision adenoidectomy, combined procedure (adenotonsillectomy), haemoglobin level <9.0 g/dL, bleeding diathesis (e.g. haemophilia or thrombocytopenia), renal or hepatic impairment, known allergy to TA, recent (<7 days before surgery) intake of antiplatelets (e.g. Aspirin, non-steroidal anti-inflammatory drugs) or Heparin administration within 48 h of operation. | | 5.6(2.5)  Vs  4.9(1.8) | - | ENT | | - Top TXA - Placebo - - | Frequency of post-operative bleeding that occurred during the initial admission or during the follow-up period | Perioperative blood loss |
| Ali Shah 2015^1503^ | | - Pakistan - English - 2015 - Single Centre - 100 - Adult patients undergoing elective on pump cardiac surgeries | | Patients for  surgeries for congenital heart diseases and thoracic aorta redo or emergency procedures, patients who were on anti-platelet drugs (Aspirin/ Clopidogrel) within 7 days of surgery, patients with impaired renal functions (creatinine clearance of < 30 ml/minutes), chronic liver disease and bleeding diathesis. | | 51.1(13.3)  Vs  48.8(12.1) | CV disease | Cardiac Surgery | | - Top TXA - Placebo - - | - | Perioperative blood loss |
| Alipour 2013^1504^ | | - Iran - English - 2013 - Single-Centre - 53 - Patients undergoing knee arthroplasty | | Patients with any history of severe ischaemic heart diseases, renal failure, cirrhosis, history of bleeding disorders or thromboembolic events | | 68.6(10)  Vs  63.1(12.4) | - | Orthopaedic Surgery | | - PO TXA - No TXA - - | The bleeding rate in surgery drains at 12 and 24 h after surgery. | Risk & number of RBC transfusion  Perioperative blood loss |
| Alizadeh 2014^1505^ | | - Iran - English - 2014 - Single-Centre - 200 - Patients undergoing elective coronary artery revascularisation | | Patients with a serum creatinine level of >2 mg/dl, previous history of bleeding or coagulation disorders, taking oral anticoagulation medications within 72 hours of the surgery and allergy to the study medications | | 58(9)  Vs  59(10) | CV disease  Renal disease | Cardiac Surgery | | - IV TXA - Placebo - - | The total volume of mediastinal bleeding during the first 24 hours after surgery | MI  Adverse Reaction  AKI  Acute brain injury  Sepsis  Risk & number of RBC transfusion  Perioperative blood loss  Risk of receiving non red cell component |
| Alshryda 2013^1506^ | | - UK - English - 2013 - Single-Centre - 157 - Patients undergoing unilateral primary total hip replacement | | Not stated | | 66(9)  Vs  63(11) | - | Orthopaedic Surgery | | - IV TXA - Placebo - - | Blood transfusion rate | Drain blood loss, haemoglobin concentration drop, generic quality of life (EuroQol), Oxford Hip Score, length of stay, a cost analysis, and complications. |
| Altun 2017^1507^ | | - Turkey - English - 2017 - Single-Centre - 28 - Emergency coronary bypass surgery patients under the influence of dual antiplatelet therapy | | Patients with chronic renal insufficiency, hepatic dysfunction, haematological disorders, drug addiction that might affect the haematological system, requirements for non-coronary cardiac surgery, or use of intra-aortic balloon pumps | | 65.8(6.1)  Vs  57.9(14.6) | CV disease | Cardiac Surgery | | - IV TXA - No TXA - - | - | Hb values  Total drains drainage  Thrombotic complications  Length of ICU and Hospital stay |
| Alvarez 2008^705^ | | - Spain - English - 2008 - Single-Centre - 95 - All patients ASA‐I to ‐III patients diagnosed with osteoarthritis and undergoing unilateral bicondylar cemented total knee arthroplasty. | | Patients with known allergy to tranexamic acid, ASA‐IV physical status or higher, severe ischemia and/or heart valve disease, history of thromboembolic episodes, known coagulopathy, and renal dysfunction (serum creatinine concentration, >1.5 mg/dL). | | 71(9)  Vs  72(7) | - | Orthopaedic surgery | | - IV TXA - Placebo - Iron therapy | Transfusion rate | Postoperative blood loss |
| Andreasen JJ 2004^1508^ | | - Denmark - English - 2004 - Single-Centre - 44 - Primary, elective, on-pump coronary artery bypass grafting (CABG) patients with low baseline risk of postoperative bleeding | | Treatment with acetylsalicylic acid, non-steroidal anti-inflammatory drugs or other platelet inhibitors within 7 days before surgery | | 62.3(9.5)  Vs  63.8(7.6) | CV disease  Anaemia | Cardiac Surgery | | - IV TXA - Placebo - Cell salvage | Postoperative blood loss and the proportion of patients requiring allogeneic transfusion | Development of perioperative myocardial infarction (peak CK-MB . 50 U/l and/or development of new Q waves), acute renal insufficiency (creatinine value twice the baseline or need for dialysis), transient ischemic attacks or stroke, early mortality (<30 days+ hospital mortality) and mediastinal infection within 30 days. |
| Antinolfi 2014^1509^ | | - Italy - English - 2014 - Single-Centre - 40 - Patients receiving primary unilateral total knee arthroplasty due to primary knee osteoarthritis | | Tranexamic acid allergy, the use of pharmacological anticoagulant therapy, previous knee surgery and renal failure | | 71.9(5.1)  Vs  70.7(7.3) | - | Orthopaedic surgery | | - IA TXA - No TXA - - | - | - |
| Apipan 2017^1510^ | | - Thailand - English - 2017 - Single-Centre - 40 - Patients scheduled for elective bi-maxillary osteotomy | | Patients with a known allergy to the study drug, a history or a risk of thromboembolism (including taking oral contraceptive pills), or a body mass index (BMI) more than 30 kg/m2 | | 25.6(4.7)  Vs  25.3(5.2)  Vs  26.4(5.5)  Vs  25.2(4.9) | - | OMFS | | - IV TXA (20mg/kg) - IV TXA (15mg/kg) - IV TXA (10mg/kg) - Placebo - - | Intraoperative blood loss and the number of patients receiving a transfusion of allogeneic blood products. | Difference between preoperative and 24-h postoperative haematocrit, the volume of 24-h postoperative vacuum drainage, and the length of hospital stay. |
| Arantes 2016^1511^ | | - Brazil - English - 2016 - Single-Centre - 70 - Patients who underwent primary palatoplasty with no known or suspected coagulation disorders | | Patients with a platelet count lower than 100,000/mm3, with known or suspected coagulation disorders, family history of coagulopathy, or indication of secondary palatoplasty for the correction of oronasal fistula | | - | - | OMFS | | - IV TXA - Placebo - - | - | The occurrence of significant haemorrhagic events, defined as the  need to use blood products, the need to redo surgery, or the need to  use antifibrinolytic drugs during the postoperative period to control  excessive bleeding, |
| Armellin 2001^1512^ | | - Italy - English - 2001 - Single-Centre - 300 - Adult cardiac surgery patients | | Patients with a  known coagulopathy, thrombocytopenia (platelet count, 100,000/mm3),  anaemia (haemoglobin level, <10 g/dL), hepatic or renal dysfunction  (Creatinine level, >1.5 mg/dL), or endocarditis, autologous blood donors, patients undergoing redo procedures, and patients who refuse blood transfusion for religious reasons. | | 65.7(11.7)  Vs  65.9(12.8) | CV disease | Cardiac surgery | | - IV TXA - Placebo - - | - | - |
| Ausen 2015^1513^ | | - Norway - English - 2015 - Single-Centre - 30 - Consecutive women undergoing bilateral reduction mammoplasty | | A history of any thromboembolic disease, pregnancy or severe co-morbidity (American Society of Anaesthesiologists (ASA) fitness grade III or IV) | | 45(12.2)  Vs  45(12.2) | - | Plastic Surgery | | - IV TXA - Placebo - - | Drain fluid production in the first 24 h after surgery. | Postoperative pain, which was registered for each breast both 3 and 24 h after surgery, using a visual analogue scale from 0 (no pain) to 10 (unbearable). |
| Auvinen 1987^1514^ | | - Finland - English - 1987 - Single-Centre - 76 - Patients who came for scheduled thyroid surgery | | Not stated | | 50(16.4)  Vs  51(13.5) | Cancer | ENT | | - IV TXA - Placebo - - | - | - |
| Bansal 2017^1515^ | | - India - English - 2017 - Single-Centre - 400 - Patients who were planned for percutaneous nephrolithotomy | | Patients having hypersensitivity to tranexamic acid, defective  colour vision, anticoagulant usage, subarachnoid haemorrhage,  abnormal liver function test, unstable cardiovascular  disease, acute or chronic renal failure or any haematological  disease | | 32.7(13.7)  Vs  34.7(15.2) | - | Urology | | - IV TXA - Placebo - - | fall in haemoglobin/haematocrit level and totalblood loss. | Overall complications  rate of PCNL |
| Baradaranfar 2017 | | - Iran - English - 2017 - Single-Centre - 60 - Patients with chronic rhinosinusitis with polyposis | | Patients with previous sinus or nasal surgery, underlying disease with increased risk of thromboses (hypercoagulable states) such as Factor V Leiden, antiphospholipid syndrome, heparin-induced thrombocytopenia, cancer, pregnancy, high blood pressure (systolic >140 mmHg and/or diastolic >90 mmHg), contraindications for the use of tranexamic acid (active clot inside arteries), and patient unwillingness or participation in other similar clinical trials. | | 38.6  Vs  40.7 | - | ENT | | - Top TXA - Placebo - - | - | - |
| Barrachina 2016^1517^ | | - Spain - English - 2016 - Multi-Centre - 78 - ASA physical status I to III patients undergoing unilateral total hip replacement surgery | | pregnancy or breastfeeding, severe vascular ischemia,  history of venous thrombosis, pulmonary embolism or diseases causing embolism, known coagulopathies, long-term treatment with acetylsalicylic acid or nonsteroidal anti- inflammatory drugs not discontinued before surgery, a haemoglobin (Hb) concentration <10 mg/dL, moderate renal impairment, liver cirrhosis, or any contraindications to prophylaxis with enoxaparin. | | 62.5(13.0)  Vs  67.5(12.1) | - | Orthopaedic Surgery | | - IV TXA - Placebo - Cell salvage | total blood loss up to day 2 after surgery | Blood loss up to 1 and 6 hours after the start of surgery. |
| Baruah 2016^1518^ | | - India - English - 2016 - Single-Centre - 60 - Patients who underwent open reduction and internal fixation with a dynamic hip screw plate for stable trochanteric fracture | | Patients who had (1) a fracture unsuitable for dynamic hip screw plate fixation, (2) an allergy to TXA, (3) preoperative renal impairment (serum creatinine >2 mg% or creatinine clearance  <30 ml/min), (4) preoperative hepatic impairment (international normalised ratio [INR] for prothrombin time >1.5 or liver enzymes elevated by >3 times the normal range, (5) known bleeding disorder or preoperative coagulation anomaly determined by prolonged bleeding time and clotting time, an INR >1.5, or a prolonged partial thromboplastin time, (6) a history of any thrombo-embolic events (such ascerebrovascular accident, acute coronary syndrome/ myocardial infarction, pulmonary embolism, deep vein thrombosis, or arterial thrombosis), (7) anticoagulants or aspirin-like drugs, oestroprogestive drugs, or long-acting non-steroidal anti-inflammatory drugs, or (8) were pregnant or breastfeeding. | | 57.6(14.4)  Vs  55.3(15.1) | Anaemia | Orthopaedic Surgery | | - IV TXA - Placebo - - | - | - |
| Basavaraj 2017^1519^ | | - India - English - 2017 - Single-Centre - 60 - Patients undergoing thoracic spine fixation | | Patients with pre-existing  renal or hepatic disorder, bleeding diathesis, history of malignancy or coronary artery disease, thromboembolic event 1 year prior to surgery,  haemoglobin< 8gm/dL, and history of uncontrolled hypertension | | 54.3(5.7)  Vs  54.7(5.7) | - | Orthopaedic Surgery | | - IV TXA - Placebo - - | - | Perioperative blood loss, amount of blood transfusion, postoperative haemoglobin and haematocrit levels. |
| Beikaei 2015^1520^ | | - Iran - English - 2015 - Single-Centre - 100 - Normotensive patients scheduled for elective open rhinoplasty aged 16-42 years with ASA class of either I or II without a history bleeding diathesis | | Presence of a history of allergy or hypersensitivity to Tranexamic acid, brain vascular diseases, coronary artery diseases, cardiac dysrhythmia, liver/kidney or metabolic disorders, ASA class of either III or IV. | | 25.9(6.6)  Vs  26(5) | - | ENT | | - IV TXA - Placebo - - | estimated volume of intraoperative bleed | No secondaryoutcome measures were defined. |
| Benoni 1996^1521^ | | - Sweden - English - 1996 - Single-Centre - 86 - Patients with knee arthroplasty | | - | | 76(7)  Vs  74(7) | - | Orthopaedic Surgery | | - IV TXA - Placebo - - | - | - |
| Benoni G 2000^1522^ | | - Sweden - English - 2000 - Single-Centre - 40 - Primary total hip replacement operations | | Not stated | | 69.5(10)  Vs  68(10) | - | Orthopaedic Surgery | | - IV TXA - Placebo - - | - | - |
| Benoni G 2001^1523^ | | - Sweden - English - 2001 - Single-Centre - 39 - Patients with primary total hip arthroplasties | | Patients who were to  undergo bone grafting or had bleeding disorders or  signs of renal insufficiency | | 66(9.5)  Vs  68(9.4) | - | Orthopaedic Surgery | | - IV TXA - Placebo - - | - | - |
| Bidolegui 2014^1524^ | | - Argentina - English - 2014 - Single-Centre - 50 - Osteoarthritis patient undergoing primary unilateral total knee arthroplasty | | Patients who had allergy to tranexamic acid, a prior history of thromboembolic disease, congenital or acquired coagulopathy, renal or liver dysfunction, myocardial infarction within the last 6 months or retinopathy. | | 71.5(9.4)  Vs  72(6.8) | - | Orthopaedic Surgery | | - IV TXA - Placebo - - | transfusion rate | Drain output, haemoglobin/haematocritlevels. |
| Blauhut 1994^1525^ | | - Switzerland - English - 1994 - Single-Centre - 30 - Patients undergoing cardiopulmonary bypass for coronary disease | | Intake of aspirin, other nonsteroidal anti-rheumatics, or beta-lactam antibiotics; treatment with heparin, fibrinolytic agents, or oral anticoagulants; a condition requiring emergency surgery or reoperation; and liver or kidney disease. | | 62.5(2.2)  Vs  62.7(2.6) | CV disease | Cardiac surgery | | - IV TXA - No TXA - - | - | - |
| Boylan JF 1996^1526^ | | - Canada - English - 1996 - Single-Centre - 45 - Patients undergoing primary isolated orthotopic liver transplantation | | Patients with primary biliary cirrhosis, Primary sclerosing cholangitis, predisposition to a thrombotic tendency, fulminant hepatic failure. | | 49.5(9.1)  Vs  48.8(9.6) | CV disease | Hepatobiliary Surgery | | - IV TXA - Placebo - - | - | - |
| Bradshaw 2012^1527^ | | - Australia - English - 2012 - Single-Centre - 46 - Orthopaedic Patients for primary total knee replacement as a treatment for osteoarthritis | | Patients with a history of thromboembolic events, anticoagulation that  could not be ceased within the recommended timeframe before surgery, peripheral vascular disease, oral contraception, pregnancy, current bleeding at any site, immunocompromised  from a known medical condition or medical  therapy, known hypersensitivity to the study medication, creatinine clearance of less than 30 mLs/min, or significant hepatic disease | | 67.1(9.4)  Vs  68.2(9.8) | - | Orthopaedic Surgery | | - PO TXA - Placebo - Restrictive threshold | - | Haemoglobin and haematocrit taken 24 hours postoperatively and total blood loss in wound drains at 24 hours. |
| Brown RS 1997a^1528^ | | - USA - English - 1997 - Single-Centre - 60 - Adult patients undergoing primary coronary artery bypass grafting surgery | | Patients with a platelet count less than 100,000/mm^3 or a coagulopathy, or those  receiving thrombolytic therapy or warfarin | | 62(10)  Vs  59(7) | CV disease | Cardiac surgery | | - IV TXA - Placebo - Restrictive threshold - Cell salvage | - | Mediastinal chest tube blood loss measured hourly for the first 24 h in the ICU.  New stroke or deaths for any reason within 30 days  Mediastinal or systemic infections within 30 days |
| Brown RS 1997b^1528^ | | - USA - English - 1997 - Single-Centre - 60 - Adult patients undergoing primary coronary artery bypass grafting surgery | | Patients with a platelet count less than 100,000/mm^3 or a coagulopathy, or those  receiving thrombolytic therapy or warfarin | | 61(9)  Vs  59(7) | CV disease | Cardiac surgery | | - IV TXA - Placebo - Restrictive threshold - Cell salvage | - | Mediastinal chest tube blood loss measured hourly for the first 24 h in the ICU.  New stroke or deaths for any reason within 30 days  Mediastinal or systemic infections within 30 days |
| Bulutcu 2005^1529^ | | - Turkey - English - 2005 - Single-Centre - 50 - Children undergoing cardiac surgery | | Patients undergoing reoperations with  sternotomy within 6 months after using Aprotinin or tranexamic acid, patients that required emergency operations, patients taking aspirin, dipyridamole or other anticoagulants, and known coagulation disorders, known metabolic disorders, renal or hepatic insufficiency, or previous exposure to Aprotinin or tranexamic acid | | 3.8(2.4)  Vs  4.1(2) | CV disease | Cardiac surgery | | - IV TXA - No TXA - Cell salvage | - | - |
| Cao 2015^1530^ | | - China - Chinese - 2015 - Single-Centre - 100 - Patients who underwent total knee arthroplasty | | - | | 64.4(5.5)  Vs  65.3(5.2) | - | Orthopaedic Surgery | | - IV TXA - No TXA - Restrictive threshold | - | - |
| Carabini 2017^1531^ | | - USA - English - 2017 - Single-Centre - 61 - Patients undergoing multi-level complex spinal fusion with and without osteotomies (more than 18 years old, had no reported history of arterial or venous thromboembolic disease, and had a more than 80% chance of requiring major transfusion) | | Patients with a history of severe coronary artery disease defined as more than 50% occlusive disease or a history of revascularization, cerebral vascular disease with previous cardiovascular accident or transient ischemic attack, venous thromboembolism, or renal insufficiency with a glomerular filtration rate of less than 40 mL/min/m^2. Patients were also excluded if they were unable or unwilling to provide informed consent or were undergoing surgery for tumour, trauma, or infection. | | 65(5.1)  Vs  68(3.7) | CV disease  Anaemia | Spinal Surgery | | - IV TXA - Placebo - Cell salvage | The total volume of red blood cells transfused intraoperatively. | Estimated blood loss, platelet and cryoprecipitate transfusion, and 24-hour postoperative allogenic  PRBC transfusion. |
| Carvalho 2015^1532^ | | - Brazil - English - 2015 - Single-Centre - 125 - Patients undergoing total knee arthroplasty | | Allergy to TXA or povidone-iodine solution, preoperative anaemia, refusal of blood products, preoperative use of anticoagulants (acetylsalicylic acid, enoxaparin, or any other, oral or intravenous, agent), fibrinolytic disorders, coagulopathy, arterial or venous thromboembolic disease and pregnancy | | 70(8.2)  Vs  70.8(6.5)  Vs  69.3(6) | - | Orthopaedic Surgery | | - Top TXA - Top TXA - Placebo - - | - | Haematimetrics indices (haemoglobin, haematocrit, prothrombin time, activated partial thromboplastin time and international normalised ratio), drain volume (mL), allogenic blood transfusion, thromboembolic events, total calculated blood loss and acute postoperative infection. |
| Casati 2001^1533^ | | - Italy - English - 2001 - Single-Centre - 510 - Patients undergoing elective cardiac surgery with use of cardiopulmonary bypass | | Patients with chronic renal insufficiency (plasmatic creatinine concentration more than 2 mg/kg), history of hematologic disorders, hepatic dysfunction (active hepatitis, cirrhosis), history of pulmonary embolism, deep venous thrombosis, and cerebrovascular injury. | | 64(16.5)  Vs  64(18.5)  Vs  61(16.7) | - | Cardiac surgery | | - IV TXA (2mg/kg/h) - IV TXA (1mg/kg/h) - Placebo - - | Bleeding | Hematologic data, allogeneic transfusions, thrombotic complications, intubation time, and intensive care unit and hospital stay duration also were evaluated. |
| Casati 2002^1534^ | | - Italy - English - 2002 - Single-Centre - 60 - Patients undergoing elective surgery involving thoracic aorta | | Patients with advanced chronic renal insufficiency (creatinine >2 mg/dL), active chronic hepatitis or cirrhosis, and history of hematologic disorders. | | 59(13)  Vs  63(11) | CV disease | Cardiac surgery | | - IV TXA - Placebo - Restrictive threshold | Perioperative bleeding | Perioperative allogeneic transfusions, major thrombotic complications (myocardial infarction, pulmonary embolism, renal insufficiency), and surgical outcomes |
| Casati 2004a ^1535^ | | - Italy - English - 2004 - Single-Centre - 51 - Patients scheduled for on-pump coronary artery bypass grafting | | Patients with a history of hematologic disease, chronic renal insufficiency (creatinine level >2 mg/dL), and liver disease (active chronic hepatitis or cirrhosis). | | 64(9)  Vs  60(9) | CV disease | Cardiac surgery  Cardiac surgery | | - IV TXA - Placebo - Restrictive threshold | Bleeding in the first 24 postoperative hours | Requirement for allogeneic transfusions, thrombotic complications, outcomes, and monitoring of coagulation, fibrinolysis, and inflammation |
| Casati 2004b^1535^ | | - Italy - English - 2004 - Single-Centre - 51 - Patients scheduled for off-pump coronary artery bypass grafting | | Patients with a history of hematologic disease, chronic renal insufficiency (creatinine level >2 mg/dL), and liver disease (active chronic hepatitis or cirrhosis). | | 64(12)  Vs  61(11) | CV disease | Cardiac surgery | | - IV TXA - Placebo - Restrictive threshold | Bleeding in the first 24 postoperative hours | Requirement for allogeneic transfusions, thrombotic complications, outcomes, and monitoring of coagulation, fibrinolysis, and inflammation |
| Castelli 1977^1536^ | | - Germany - German - 1977 - Single-Centre - 80 - Patients who underwent tonsillectomy | | - | | 29.5(10.5)  Vs  29.9(11.7) | - | ENT | | - IV TXA - No TXA - - | - | - |
| Castro-Menendez 2016^1537^ | | - Spain - English - 2016 - Single-Centre - 240 - Patients underwent total hip and knee arthroplasty | | Patients with (1) inflammatory or autoimmune disease; (2) blood coagulation disorders; (3) a history of thromboembolic dis-ease; (4) severe anaemia (preoperative Hb <7 mg/dl); (5)peripheral neuropathy; (6) malign tumour; (7) contraindication or intolerance of the administration of low molecular weight heparin or TXA; (8) a history of epilepsy or severe kidney failure, defined as an estimated glomerular filtration rate of <30 mg albumin per g of creatinine in urine (9),patients with an ASA score of 4 or 5 | | - | - | Orthopaedic Surgery | | - IV TXA (2g) - IV TXA (1g+1g) - No TXA - Restrictive threshold | - | Postoperative blood loss, transfusion rate, and thromboembolic complications |
| Cavusoglu 2015^1538^ | | - Turkey - Turkish - 2015 - Single-Centre - 60 - Patients who were performed primary total knee arthroplasty | | Cardiovascular, cerebrovascular, thromboembolic problems, coagulation disorders and simultaneous bilateral TKA were defined as exclusion criteria. | | 67.8(8.5) | - | Orthopaedic Surgery | | - IV TXA - IA TXA - No TXA - - | - | Pre- and postoperative haemoglobin difference, volume of blood collected in drains, and transfusion rate |
| Chakravarthy 2012a^1539^ | | - India - English - 2012 - Single Centre - 50 - Patients underwent off pump coronary artery bypass surgery | | Emergency OPCAB surgery. Pre-existing coagulation disorders, Recent thrombolysis (in less than 2 days), and patients on antiplatelet medications. Hemodynamic instability - heart rate >130, MAP<50, CVP>15, PAWP>23. Patient likely to need cardiopulmonary bypass (such as patients with narrow coronary arteries likely to require endarterectomy, combined valve and coronary surgery) low ejection fraction, recent MI, requirement of intra-aortic balloon pump and or mechanical ventilation in the preoperative period. Preoperative anaemia Hb less than 9g/dL. Dysfunctions of major organ such as renal and or hepatic failure. Patients with history of convulsion / or receiving anticonvulsant medications | | 58(4)  Vs  60(6) | - | Cardiac Surgery | | - IV TXA+HES - Placebo - POC testing - Cell salvage | - | Intraoperative blood loss by gravimetric method and postoperative blood loss was measured by calculating blood volume lost in the drains until the time of their removal. Duration on ventilator, length of stay (LOS) intensive care unit (ICU) stay were also assessed. Any adverse events such as seizures was noted. |
| Chakravarthy 2012b^1539^ | | - India - English - 2012 - Single-Centre - 50 - Patients underwent off pump coronary artery bypass surgery | | Emergency OPCAB surgery. Pre-existing coagulation disorders, Recent thrombolysis (in less than 2 days), and patients on antiplatelet medications. Hemodynamic instability - heart rate >130, MAP<50, CVP>15, PAWP>23. Patient likely to need cardiopulmonary bypass (such as patients with narrow coronary arteries likely to require endarterectomy, combined valve and coronary surgery) low ejection fraction, recent MI, requirement of intra-aortic balloon pump and or mechanical ventilation in the preoperative period. Preoperative anaemia Hb less than 9g/dL. Dysfunctions of major organ such as renal and or hepatic failure. Patients with history of convulsion / or receiving anticonvulsant medications | | 62.5(4)  Vs  64(4) | - | Cardiac Surgery | | - IV TXA+RL - Placebo - POC testing - Cell salvage | - | Intraoperative blood loss by gravimetric method and postoperative blood loss was measured by calculating blood volume lost in the drains until the time of their removal. Duration on ventilator, length of stay (LOS) intensive care unit (ICU) stay were also assessed. Any adverse events such as seizures was noted. |
| Chareancholvanich 2012a^1540^ | | - Thailand - English - 2012 - Single-Centre - 120 - Patients who diagnosed primary osteoarthritis and scheduled to undergo primary total knee arthroplasty | | Patients who had secondary osteoarthritis (such as rheumatoid arthritis, post-traumatic arthritis, gouty arthritis, post septic arthritis), high risk medical co-morbidity, history of thromboembolic disease, bleeding disorder, known allergy to tranexamic acid, and receiving the anti-coagulant drugs | | 70.1(7.2)  Vs  68.9(7.5) | - | Orthopaedic Surgery | | - IV TXA (post-op) - Placebo - - | - | The amount of drained blood was recorded at 48 hrs. At 48 hours after the operation, the Hb levels of all patients were recorded. Clinical thromboembolic events and wound complications were also examined. |
| Chareancholvanich 2012b^1540^ | | - Thailand - English - 2012 - Single-Centre - 120 - Patients who diagnosed primary osteoarthritis and scheduled to undergo primary total knee arthroplasty | | Patients who had secondary osteoarthritis (such as rheumatoid arthritis, post-traumatic arthritis, gouty arthritis, post septic arthritis), high risk medical co-morbidity, history of thromboembolic disease, bleeding disorder, known allergy to tranexamic acid, and receiving the anti-coagulant drugs | | 69.4(6.3)  Vs  69.8(6.3) | - | Orthopaedic Surgery | | - IV TXA (pre-op) - Placebo - - | - | The amount of drained blood was recorded at 48 hrs. At 48 hours after the operation, the Hb levels of all patients were recorded. Clinical thromboembolic events and wound complications were also examined. |
| Charoencholvanich 2011^1541^ | | - Thailand - English - 2011 - Single-Centre - 100 - Patients with primary osteoarthritis undergoing unilateral cemented total knee arthroplasty | | Patients with secondary osteoarthritis (e.g., rheumatoid arthritis, posttraumatic arthritis, gouty arthritis, post septic arthritis), and patients with a high-risk medical comorbidity, simultaneous bilateral TKAs, history of thromboembolic disease, bleeding disorder, known allergy to tranexamic acid, and receiving anticoagulant drug treatment | | 69.2(6.1)  Vs  68.8(6.1) | - | Orthopaedic Surgery | | - IV TXA - Placebo - - | - | Differences in the mean age, preoperative  haemoglobin, volume of drained blood, decrease in  haemoglobin 12 hours postoperatively, and the mean number  of transfused units |
| Chaudhary 2018^1542^ | | - Pakistan - English - 2018 - Single-Centre - 100 - Patients scheduled for primary isolated elective or urgent open heart surgery | | Patients with abnormal coagulation profile. | | - | CV disease | Cardiac Surgery | | - Top TXA - Placebo - - | - | 48 hours of blood loss, number of pints transfused, perioperative complications, re-exploration for excessive bleeding. |
| Chauhan 2003^1543^ | | - India - English - 2003 - Single-Centre - 120 - Children with cyanotic heart disease | | Patients with renal impairment, previous neurological events or congenital bleeding disorders | | 4.4(3.6)  Vs  4.2(3.3) | CV disease | Congenital surgery | | - IV TXA - No TXA - - | - | Postoperatively, total mediastinal chest tube drainage and blood and blood product usage at 24 h were recorded. Tests of coagulation including activated clotting time, fibrinogen, fibrin degradation products and platelet count were performed at 6 h postoperatively. |
| Chauhan 2004^1544^ | | - India - English - 2004 - Single-Centre - 150 - Children with congenital cyanotic heart disease | | Patients with renal dysfunction, a previous neurological event, or a congenital bleeding disorder | | 3.3(2.9)  Vs  4.2(4)  Vs  3.0(4.3)  Vs  2.9(5)  Vs  4.3(3.3) | CV disease | Cardiac surgery | | - IV TXA (Induction) - IV TXA (Induction+Infusion) - IV TXA (Induction+bypass+end) - IV TXA (Induction+end) - Placebo - - | - | Postoperative cumulative blood loss was recorded at 24 hours. Use of blood and blood products was noted at 24 hours. Blood samples were collected at 6 hours for tests of coagulation including activated clotting time, fibrinogen, fibrin degradation products, and platelet count. |
| Chen 2008^1545^ | | - Taiwan - English - 2008 - Single-Centre - 60 - Patients who underwent head and neck operations | | Patients with an allergy to TXA, a history of hematologic disorders,  advanced chronic renal insufficiency (creatinine >2mg/dL), undergoing anticoagulation therapy, previous radiation  to the head and neck region, or who were reluctant to enrol in this protocol | | 49.8(13)  Vs  46.4(14.8) | - | ENT | | - IV TXA - No TXA - - | - | Basic data, laboratory study, and operation types, which included gender, age, prothrombin time (PT), activated partial thromboplastin time (aPTT), plasma fibrinogen, D-dimers, and perioperative blood loss, were obtained and recorded. |
| Chen 2016a^1546^ | | - Singapore - English - 2016 - Single-Centre - 56 - Patients undergoing unilateral total knee arthroplasty | | Patients with history of renal impairment, cardiovascular diseases (previous myocardial infarction, atrial fibrillation) or cerebrovascular conditions (previous stroke or peripheral vascular surgery) | | - | Anaemia | Orthopaedic Surgery | | - IV TXA - No TXA - - | - | The patient blood routine examination was performed at the 3rd day, and at the same time, the volume of drainage was recorded; and the colour Doppler ultrasound in ipsilateral lower extremity veins was conducted to observe the incidence of thrombosis at 4-5 days. |
| Chen 2016b^1547^ | | - China - English - 2015 - Single-Centre - 120 - Patients undergoing simultaneous bilateral total knee arthroplasty | | Age less than 18, age greater than 80, bleeding or clotting disorders, preoperative anticoagulation therapy, renal disorders or insufficiency, cardiovascular problems, cerebrovascular conditions, thromboembolic disorders, preoperative anaemia, and allergy to TXA | | 66.5(7.1)  Vs  64.2(6.2) | - | Orthopaedic Surgery | | - IV TXA - Placebo - - | Total blood loss. | Blood transfusion rate, transfusion units, intraoperative blood loss, drainage volumes, hidden blood loss, maximum decline of haemoglobin, and postoperative suprapatellar girth increment. |
| Chen 2018^1548^ | | - China - Chinese - 2018 - Single Centre - 60 - Patients undergoing unilateral total knee replacement | | - | | 67.1(8.2)  Vs  67(8)  Vs  67.2(8.4) | - | Orthopaedic Surgery | | - IV TXA - IA TXA - No TXA - - | - | The postoperative dominant and hidden blood loss, blood transfusion rate, pulmonary embolism as well as lower extremity deep venous thrombosis |
| Chen 2013^1549^ | | - China - English - 2013 - Single-Centre - 120 - Patients undergoing heart valve replacement surgery during cardiopulmonary bypass | | Patients with 1) Age greater than 80 years; 2) re-operation; 3) use of hormone and antibiotics 1 week prior to the surgery; 4) preoperative examinations that revealed severe coagulation abnormalities such as significant prolongation of prothrombin time and significant reduction in thrombocytes; 5) severe liver and renal failure; 6) detection of pericardial adhesions during surgery; 7) receipt of treatment with recombinant human coagulation factor VII during and after surgery. | | 50.4(9.7)  Vs  50.4(10) | CV disease | Cardiac Surgery | | - IV TXA - Ulinastatin - TXA+Ulinastatin - No TXA - - | - | Hospital LOS  Perioperative blood loss |
| Choudhuri 2015^1550^ | | - India - English - 2015 - Single-Centre - 52 - Patients scheduled for open heart surgeries under cardiopulmonary bypass | | Patients undergoing redo-cardiac surgery, with renal insufficiency (serum creatinine higher than 2 mg/dl), undergoing ant platelet therapy, having haematological disorders or hepatic dysfunctions | | 33(11)  Vs  35.5(12)  Vs  39(13.1) | CV disease | Cardiac Surgery | | - EACA - IV TXA - No TXA - POC testing | - | Patients were monitored for twenty-four hours postoperatively to assess reopening rate for the management of excessive bleeding. |
| Christabel 2014^1551^ | | - India - English - 2014 - Single-Centre - 49 - Patients undergoing LeFort 1 osteotomy for correction of dentofacial deformity | | Patients with cleft lip, palate, or other facial clefts, systemic disease,  bleeding disorders, pregnant or breast feeding mothers, those with known allergy to the test drug or who were under the influence of  anticoagulants | | 24.1(4.5)  Vs  23.2(4.3) | - | OMFS | | - IV TXA - Placebo - - | Change in Hb% and PCV at 24 hours | Total blood loss by estimation of the total suctioned volume and the amount of soaked gauze minus the volume of saline used. |
| Claeys 2007^1552^ | | - Belgium - English - 2007 - Single-Centre - 40 - Patients scheduled for primary unilateral total hip replacement surgery for degenerative osteoarthrosis | | Patients with an allergy to tranexamic acid preoperative renal or hepatic dysfunction, known bleeding disorders or preoperative coagulation anomalies, anticoagulant or aspirin-like medication and long acting NSAID medication. | | 73(8)  Vs  68(11) | - | Orthopaedic Surgery | | - IV TXA - Placebo - - | - | Perioperative blood loss was measured by carefully weighting the swabs and measuring the volumes in the suction bottles during surgery. The number of units of packed cells and the time of transfusion was recorded. All patients were examined daily for clinical signs of DVT. |
| Clave 2019^1553^ | | - France - English - 2017 - Multi-Centre - 1) over 18 years of age; 2) awaiting primary elective THA; 3) scheduled for antithrombotic prophylaxis with rivaroxaban; 4) provided informed consent; and 5) registered in the national social security system. | | 1) rapidly destructive osteoarthritis of the hip; 2) previous ipsilateral hip surgery; 3) major contraindications for treatment with TXA, such as epilepsy and renal failure (renal clearance < 30 ml/min); 4) patients already receiving antiplatelet agents (aspirin > 160 mg/j) or anticoagulants; 5) ischaemic arterial disease (myocardial infarction, stroke); 6) previous venous thromboembolism (VTE); 7) contraindication to treatment with rivaroxaban and 8) Child B-stage cirrhosis with coagulopathy. | | 67.1(10.5)  Vs  65(11.9)  Vs  64.4(11.6) | - | Orthopaedic Surgery | | - Long IV TXA - Short IV TXA - Placebo | The difference in perioperative RBL between the baseline level and the level on day 3 | The haemostatic effects of TXA on the levels of Hb and Ht and on the need for transfusion. Major bleeding was defined as clinically overt bleeding accompanied by one or more of the following: a decrease in the Hb level of > 2 g/dl over a 24-hour period, transfusion of two or more units of PRBCs, bleeding at a critical site (intracranial, intra-spinal, intraocular, pericardial, intra-articular, intramuscular with compartment syndrome, or retroperitoneal), or fatal bleeding. |
| Coffey 1995^1554^ | | - USA - English - 1995 - Single-Centre - 30 - Patients who were about to undergo cardiac surgery | | Patients undergoing cardiac transplantation or patients with a scram creatinine greater than 3.0 mg/dL | | 63.9  Vs  64.7 | CV disease | Cardiac surgery | | - IV TXA - Placebo - - | - | Shed mediastinal blood and transfused homologous blood were made at 6, 12, and 24 hours postoperatively |
| Colomina 2017^1555^ | | - Spain - English - 2017 - Multi-Centre - 95 - Patients undergoing posterior instrumented spine surgery | | History of allergy or hypersensitivity to  TXA, current treatment with drugs that interfere with coagulation (oral anticoagulant or antiplatelet agents), a clinical history of frequent bleeding, baseline plasma creatinine>1.5mg dL1, platelet count<150 109 Litre1, prothrombin time (PT)<60% and activated partial thromboplastin time (APTT)>38s, history of any thromboembolic episode before surgery, or a family history of thromboembolism. | | 59.2(40.7)  Vs  50.8(42.2) | - | Spinal Surgery | | - IV TXA - Placebo - Iron therapy - Cell salvage | Total number of transfusion units required during the intraoperative and postoperative period up to postoperative day seven. | Intraoperative blood loss and total blood loss. |
| Corbeau 1995^1556^ | | - France - French - 1995 - Single-Centre - 61 - Adults undergoing either coronary artery bypass grafting (CABG) or aortic valve replacement | | Patients who were: minors, cardiac surgery re-operations, antiplatelet therapy within 10 days before the operation, hereditary or acquired coagulopathy, | | 63(19)  Vs  60(22) | CV disease | Cardiac surgery | | - IV TXA - Placebo - - | - | Transfusion requirements within 48 hours |
| Crescenti 2011^1557^ | | - Italy - English - 2011 - Single-Centre - 200 - patients older than 18 years and undergoing radical retro-pubic prostatectomy | | Patients with atrial fibrillation, coronary artery disease treated with drug eluting stent, severe chronic renal failure, congenital or acquired thrombophilia, and known or suspected allergy to tranexamic acid. | | 64(7.4)  Vs  64(7.8) | Cancer | Urology | | - IV TXA - Placebo - - | Number of patients receiving blood transfusions preoperatively | Intraoperative blood loss |
| Cvetanovich 2018^1558^ | | - USA - English - 2016 - Single-Centre - 110 - Patients undergoing primary anastomotic and reverse TSA | | Allergy to TXA, acquired disturbances of colour vision, preoperative use of anticoagulant therapy within 5 days of surgery, history of arterial or venous thromboembolic disease (including deep venous thrombosis, pulmonary embolism, stroke, transient ischemic attack), ongoing pregnancy or breast-feeding, recent myocardial infarction (within 6 months before surgery), cardiac stent placement, renal impairment, haemophilia, refusal of blood products, revision TSA, TSA performed for the indications of acute proximal humeral fracture, or prior open shoulder surgery, including failed open reduction and internal fixation of proximal humeral fractures | | 67.7(10.9)  Vs  65.2(9.2) | - | Orthopaedic Surgery | | - IV TXA - Placebo - - | Calculated postoperative blood loss. | Transfusion rates, weight of haemoglobin loss, hospital length of stay, and thromboembolic events. |
| Dadure 2011^1559^ | | - USA - English - 2011 - Single-Centre - 39 - Children, ASA status 1 or 2, scheduled to undergo surgical correction of craniosynostosis | | Children with bleeding diathesis and abnormal prothrombin time, partial thromboplastin time, or platelets counts; a history of convulsive seizures; or allergy to TXA | | 7(2.7)  Vs  6(1.5) | - | Neurosurgery | | - IV TXA - Placebo - Iron therapy | - | Perioperative blood loss, number and volume of transfusions, percentage of children who underwent transfusion, and side effects were noted after surgery and at the end of the study. |
| Dalmau 2000^1560^ | | - SPAIN - English - 2000 - Single-Centre - 82 - Patients underwent orthotopic liver transplantation | | Patients with 1) Budd-Chiari syndrome, 2) acute liver failure, 3) early re-transplantation, 4) simultaneous kidney and liver transplantation or renal insufficiency with dialysis, and 5) primary familial amyloid neuropathy. | | 58(11.7)  Vs  60(12.2) | - | Hepatobiliary Surgery | | - IV TXA - Placebo - - | - | The number of units of RBCs, FFP, platelets, and cryoprecipitate transfused were recorded throughout the procedure and during the first 24 h in the intensive care unit. |
| Das 2015^1561^ | | - India - English - 2015 - Single-Centre - 80 - Patients, ASA II-III scheduled for unilateral head and neck cancer surgeries | | Patients refusal, patients having previous HNC surgery, anaemia (haemoglobin [Hb] <10 mg/dl for women and Hb <12 mg/dl for men), abnormal coagulation profile, aspirin intake within 7 days, hepatorenal insufficiency, cardiopulmonary abnormality, pregnancy, and history of embolic  manifestations like deep venous thrombosis, transient ischemic attack, and stroke | | 43.9(10.6)  Vs  44(10.5) | Anaemia | ENT | | - IV TXA - Placebo - - | - | - |
| De Napoli 2016^1562^ | | - Argentina - Spanish - 2016 - Single-Centre - 62 - Patients going under primary hip and knee arthroplasty | | - | | 66(9.9)  Vs  67.2(8.5) | - | Orthopaedic Surgery | | - IV TXA - Placebo - Restrictive threshold | - | Preoperative and postoperative haematocrit and haemoglobin, days of stay in hospital and number of red cell unit transfusion. We looked for complications and adverse effects. |
| Dell'Amore 2012^1563^ | | - Italy - English - 2012 - Single-Centre - 89 - Patients, scheduled for pulmonary resection | | Re-do surgery anti-platelets or chronic anticoagulant therapy, liver cirrhosis, renal failure (creatinine >2 mg/dl), primary bleeding diathesis (haemophilia, etc.), known allergy to TA, preoperative documented ischaemic heart disease, presence of coronary or other arterial stents, redo surgery, pleuro/pneumonectomy or pleurectomy/decortication for mesothelioma, pleurectomy/decortication for empyema, thoracoscopic surgery, pneumonectomy, neoadjuvant chemotherapy | | 65.8(7.9)  Vs  67.7(6.4) | - | Thoracic Surgery | | - IV TXA - Placebo - - | - | Postoperative blood loss from the chest tube was recorded at 12 and 24 h from chest closure. |
| Dell'Atti 2016^1564^ | | - Italy - English - 2016 - Single-Centre - 359 - Patients taking chronic low dose aspirin, underwent trans-rectal prostate biopsy | | Patients with a history of biopsy, surgical treatment of prostatic disease, neoadjuvant therapy or incomplete clinical data | | 63.8(6.2)  Vs  64.9(7.5) | Cancer | Urology | | - Oral TXA - No TXA - - | - | Complications, their frequency, severity of bleeding |
| Digas 2015^1565^ | | - Greece - English - 2013 - Single-Centre - 90 - Patients who underwent unilateral total knee arthroplasty | | Patients with secondary and patients with history of thromboembolic disease, bleeding disorder, a history of hepatic or renal dysfunction and severe cardiac respiratory disease. | | 70(6.5)  Vs  71(7)  Vs  68(5.5) | - | Orthopaedic Surgery | | - IV TXA - IA TXA - Placebo - - | - | Thromboembolic complications, such as clinical deep vein thrombosis and pulmonary emboli, and other complications (e.g., wound complications) were noted during the hospital stay |
| Diprose 2005^1566^ | | - UK - English - 2005 - Single-Centre - 123 - Patients undergoing first-time cardiac surgery | | Patients with emergency surgery, combined or re-do surgery, the use of two or more antiplatelet therapies within 72 h of surgery, carotid stenosis of >50%, any chronic  inflammatory process, steroid therapy, liver disease, or any patient not prepared to receive an allogeneic transfusion | | 65(10.2)  Vs  62(14)  Vs  65(10.2) | CV disease | Cardiac surgery | | - IV TXA - Aprotinin - Placebo - Cell salvage | Number of patients in each group exposed to allogeneic red cell transfusion, allogeneic coagulation product transfusion or any allogeneic transfusion (allogeneic red cell and/or allogeneic coagulation product) during their hospital stay. | Mediastinal drain losses and markers of myocardial injury. |
| Drakos 2016^1567^ | | - Greece - English - 2014 - Single-Centre - 200 - Patients over 65years with intertrochanteric fracture treated by intramedullary nail | | Polytrauma patients, patients with pathologic fractures or known history of malignancy, delayed surgery beyond 48 hours, known allergy to tranexamic acid, history of venous or arterial thromboembolic disease, hepatic failure, severe renal insufficiency, hematologic disorder, Coumadin anticoagulant medication, and coagulopathy (INR >1.4). | | 81(22.9)  Vs  80.7(23.7) | - | Orthopaedic Surgery | | - Top TXA - No TXA - - | - | Complications at the surgical site (hematoma formation, infection and wound dehiscence), deep vein thrombosis, pulmonary embolism, myocardial infarction and cerebral stroke |
| Drosos 2016^1568^ | | - Greece - English - 2016 - Single-Centre - 90 - Patients who underwent total knee replacement using enhanced recovery after surgery regime | | Patients with a history of thromboembolic episode, hepatic/cardiorespiratory/renal insufficiency, and congenital or acquired coagulopathy | | 69.2(7.2)  Vs  71.1(6.3)  Vs  71.7(6.5) | - | Orthopaedic Surgery | | - IV TXA - Top TXA - No TXA - - | Calculated blood loss and the need for allogeneic blood transfusion. | Complications such as symptomatic deep vein thrombosis (DVT), pulmonary embolism, or any other thromboembolic event, superficial and deep infections and any deterioration of hepatic or renal function during the first 30 post-operative days. |
| Dryden 1997^1569^ | | - Canada - English - 1997 - Single-Centre - 41 - Patients scheduled for re-do valve replacement | | Patients with a history of thrombosis, pre-existing coagulopathy, creatinine > 250 mg/dl, or a known allergy to TA. A history of thrombosis referred to previous deep vein thrombosis, disseminated intravascular coagulation,  non-embolic stroke within six months, unstable angina, or bleeding into the renal tract | | 63(12.6)  Vs  61(12.5) | CV disease | Cardiac surgery | | - IV TXA - Placebo - - | - | Blood loss, and the transfusion of blood products. |
| Eftekharian 2014^1570^ | | - Iran - English - 2014 - Single-Centre - 56 - Patients who underwent orthognathic surgery | | Patients with coagulopathy, those who used anticoagulants, and those requiring  additional procedures | | 21.6(3.8)  Vs  22.7(6) | - | OMFS | | - IV TXA - No TXA - - | Blood loss | Age, gender, surgical time, the amount of irrigation solution used, baseline hemoglobin and hematocrit, and weight |
| Ekback 2000^1571^ | | - Sweden - English - 2000 - Single-Centre - 40 - Patients undergoing total hip replacement | | Not stated | | 66.4(9)  Vs  65.6(8.8) | - | Orthopaedic Surgery | | - IV TXA - Placebo - Restrictive threshold - Cell salvage | - | - |
| El Shal 2015^1572^ | | - Egypt - English - 2015 - Single-Centre - 90 - Patients ASA I-II aged from 18 to 50 years and undergoing functional endoscopic sinus surgery | | Patients with uncontrolled hypertension, renal or hepatic dysfunction, coronary or cerebral artery disease, autonomic disturbance, deep vein thrombosis or peripheral vascular disease, bleeding diathesis and patients receiving anticoagulants were excluded from the study | | 36.5(6.9)  Vs  35.8(5.8)  Vs  36.3(5.5) | - | ENT | | - IV TXA - EACA - No TXA - - | - | The duration of surgery, volume of blood loss, pre and postoperative haemoglobin, MAP and HR, surgical field quality surgeon satisfaction and side effects |
| Eldaba 2013^1573^ | | - Egypt - English - 2013 - Single-Centre - 100 - Children recruited to undergo functional endoscopic sinus surgery | | Parent refusal, systemic diseases affecting the nose, medical treatment  affecting the study or any congenital anomalies, patients with pre-existing renal and hepatic disorders, bleeding diathesis, abnormal prothrombin time, partial thromboplastin time (PTT) or platelet counts, usage of non-steroidal anti-inflammatory drugs within 7 days of surgery | | 7.5(3.5)  Vs  7.2(3.2) | - | ENT | | - IV TXA - No TXA - - | - | Blood loss, time of operation, Side-effects of TA such as nausea, vomiting, pruritus, hematoma or haemorrhage, thrombotic complications, local infection, fever or convulsive seizure were reported. |
| Elshamaa 2015^1574^ | | - Egypt - English - 2015 - Single-Centre - 50 - Patients undergoing spine surgery | | Patients outside the age range, history of thrombo-embolic event e.g. pulmonary embolism, deep venous thrombosis, traumatic spine injury, morbid obesity (weight > 125 kg), known congenital bleeding disorder, known allergy to the used drugs and known pregnant or lactating patients. Inclusion criteria were the ability to consent, and absence of renal and hepatic diseases. | | 43.4(3.9)  Vs  41.6(3.3) | - | Spinal Surgery | | - IV TXA - No TXA - - | Total volume of blood loss in the perioperative period. | Perioperative transfusion requirement, and the number of patients who needed transfusion, as well as time of operation. |
| Elwatidy 2008^1575^ | | - Saudi Arabia - English - 2008 - Single-Centre - 64 - Patients underwent spinal surgery with expected significant blood loss | | Microdiscectomy, and patients on anticoagulation therapy or with coagulopathy, have previous thrombo-embolic events, renal impairment, hepatic disease, as well as patients known to have  contraindications to anti-fibrinolytic treatment | | 51.5(19)  Vs  49.7(21) | - | Orthopaedic Surgery | | - IV TXA - Placebo - - | - | Preoperative, intraoperative, and postoperative haemoglobin (HB) and haematocrit (HCT) values were documented, as well as the amount of blood and blood products transfused during and after surgery. |
| Emara 2014^1576^ | | - Egypt - English - 2014 - Single-Centre - 40 - Patients who underwent pelvic hemiarthroplasty | | Allergy to TXA; acquired disturbances of colour vision; pre-operative anaemia (haemoglobin <11 gm% in females and haemoglobin <12 gm% in males); pre-operative use of anticoagulant therapy, heparin within 5 days of surgery, fibrinolytic disorders requiring intraoperative anti-fibrinolytic treatment; coagulopathy i.e., pre-operative platelets count <150,000 mm, international normalized ratio (INR) >1.4 and prolonged prothrombin time (PT) >1.4 s; previous history of thromboembolic disease; significant co-morbidities; severe ischemic heart disease, New York Heart Association Class III and IV; previous myocardial infarction; severe pulmonary disease; plasma creatinine greater than 115 mmol/L in males and more than 100 μmol/L in females; hepatic failure; occurrence of intraoperative surgical/medical/anaesthetic complications; patients who need massive blood transfusion; postoperative bleeding of surgical causes. | | 56.5(2.8)  Vs  55(2.6)  Vs  56(3.1) | - | Orthopaedic Surgery | | - IV TXA - Top TXA - Placebo - POC testing | Blood loss | Thromboembolic complications (DVT, PE and cerebrovascular stroke |
| Engel 2001^1577^ | | - Germany - English - 2001 - Single-Centre - 36 - Patients underwent total knee arthroplasty | | Not stated | | 71(9)  Vs  68(11)  Vs  66(11) | - | Orthopaedic Surgery | | - IV TXA - Aprotinin - Placebo - - | - | - |
| Esfandiari 2013^1578^ | | - Iran - English - 2013 - Single-Centre - 150 - Patients who were candidates for coronary artery bypass | | Patients who had emergency surgery, rheumatic fever, bleeding diathesis (haemophilia or platelet count <100x10^9/L), renal failure (creatinine>160mg/dl), known allergy or contraindication  to TA (acquired visual defect, subarachnoid haemorrhage, gall bladder disease, emboli, venous thrombosis), recent (<7 days before surgery) intake of Plavix or heparin, or streptokinase administration within 48 h of operation | | 54.2(9.7)  Vs  54.6(10.4) | CV disease | Cardiac surgery | | - IV TXA - Placebo - - | - | Mortality, MI, Reoperation, Acute tubular necrosis, Cerebrovascular accident |
| Faraoni 2014^1579^ | | - USA - English - 2014 - Single-Centre - 33 - Cardiac surgery patients requiring cardiopulmonary bypass | | Cmergency procedures, previous sternotomy, endocarditis, complex surgeries of the aortic arch, preoperative severe chronic kidney injury (creatinine level >180mmol l1 ), preoperative haemoglobin level less than 10 g dl1 , preoperative coagulopathy, history of stroke or thrombo-embolic disease, allergy or contraindication to tranexamic acid. | | 71(14)  Vs  61(10)  Vs  62(14) | CV disease | Cardiac surgery | | - IV TXA (High) - IV TXA (Low) - Placebo - POC testing | Fibrinolysis was evaluated by thromboelastography | Blood loss, transfusion requirement and side effects. |
| Farrokhi 2011^1580^ | | - Iran - English - 2009 - Single-Centre - 92 - Patients undergoing spinal fixation surgery, aged 40 to 80 years, with physical status I and II | | Platelet count <150,000mm^3, heart disease, severe allergy to TXA, body mass index >30 kg/m2, and history of bleeding disorders. | | 45.5(11.6)  Vs  51.4(11.6) | - | Spinal Surgery | | - IV TXA - Placebo - - | - | Administered liquids (crystalloids, colloids), blood transfusions, and urine output were measured at the end of recovery. Patients were assessed daily for any thromboembolic complications. |
| Felli 2019^1581^ | | - Italy - English - 2016 - Single-Centre - 80 - All patients at our study location who received a diagnosis of ACL rupture | | Patients younger than 18 years or older than 45 years, coagulative disorders, renal impairment, treatment with drugs interfering with coagulation or TXA clearance, and thrombophilia. Also excluded were patients with a history of thrombotic disease, seizures, or ACL revision surgery; patients with a history of knee surgery on the affected knee; patients with multiligament injuries; and patients who received concomitant extra-articular anterolateral procedures. | |  |  | Orthopaedic Surgery | | - IV TXA - Placebo - - | The drained blood volume on PD 1 | Clinical data including the patellar circumference, ROM, quadriceps strength (QS), pain assessed with a visual analog scale (VAS), clinical grade of haemarthrosis, International Knee Documentation Committee (IKDC) score, and Lysholm score. |
| Fernandez-Cortinas 2017^1582^ | | - Spain - English - 2017 - Single-Centre - 134 - Patients who have undergone total hip arthroplasty operation | | Patients allergic to TXA, those with liver failure, haematological diseases, retinopathy, cerebrovascular disease, severe ischaemic cardiopathy, severe kidney failure, severe lung failure, INR > 1.4, coagulopathies, and a background of arterial or venous thromboembolic disease. | | 71.6(8.6)  Vs  71.8(8.1) | - | Orthopaedic surgery | | - IV TXA - Placebo - - | - | - |
| Fraval 2016^1583^ | | - Australia - English - 2015 - Single-Centre - 101 - Patients who underwent total hip arthroplasty | | Patients with contraindications to the use of TXA such as known drug reaction to TXA, active intravascular clotting (deep vein thrombosis [DVT], pulmonary embolism [PE], or cerebral thrombosis), predisposition to thrombosis (previously documented DVT or PE), or a subarachnoid haemorrhage. Patients with rheumatoid arthritis | | 60.2(9.1)  Vs  60.1(10.1) | - | Orthopaedic surgery | | - IV TXA - Placebo - - | thigh swelling | Visual analogue pain score, timed up and go test, a 10 meter walk test, and length of stay. Blood loss and the incidence of blood transfusions were also recorded. |
| Fraval 2018^1584^ | | - Australia - English - 2016 - Single-Centre - 105 - Patients undergoing elective total hip arthroplasty for the treatment of osteoarthritis over the age of 40 years. | | Patients with contraindications to the use of tranexamic acid such as known drug reaction to TXA, active intravascular clotting (DVT, pulmonary embolism [PE] or cerebral thrombosis), predisposition to thrombosis (previously documented DVT or PE) or a subarachnoid haemorrhage. Patients with rheumatoid arthritis were also excluded. | | 58.8(9.7)  Vs  63(9.4) | - | Orthopaedic surgery | | - IV TXA - Placebo - - | thigh swelling | Blood loss and the incidence of blood transfusions was also recorded. Secondary outcome measures including postoperative functional scores and mobility, pain scores and length of stay. |
| Garneti 2004^1585^ | | - UK - English - 2004 - Single-Centre - 50 - Patients who underwent total hip arthroplasty | | Not stated | | 69.6(11.9)  Vs  67.6(11.4) | - | Orthopaedic surgery | | - IV TXA - No TXA - - | - | - |
| Gatling 2018^1586^ | | - USA - English - 2018 - Single-Centre - 82 - Patients scheduled for primary cardiac surgery with anticipated CPB. | | Patients were excluded if they weighed < 30 kg, had pre-existing coagulopathy (INR > 1.5, platelets < 100 ×109/L), had renal failure (defined as BUN / Cr ≥ 20: 1), had severe liver disease (AST&ALT > 3x normal), or were undergoing cardiac surgery known to be associated with greater risk for bleeding and transfusion such as complex aortic surgery, or combination valve replacement with coronary artery bypass graft surgery. | | 64(3.7)  Vs  63(3.7) | CV disease | Cardiac Surgery | | - IV TXA - EACA - Restrictive threshold | difference in transfusion amounts | the amount of transfusion during the operative procedure, calculated  Red blood cell (RBC) volume change, postoperative creatinine, time to extubation, chest tube output and length of ICU stay. |
| Gautam 2013^1587^ | | - India - English - 2013 - Single-Centre - 27 - Patients who underwent total knee arthroplasty | | Patients who were allergic to tranexamic acid or having inherited or acquired hypercoagulable state, abnormal coagulation profile (BT, CT, platelet count, prothrombin time, aPTT), patients who had taken aspirin or other NSAIDS 3 days prior to surgery, patients with renal insufficiency or history of deep vein thrombosis or pulmonary embolism and people who were at risk of these | | 60.5(23.7)  Vs  56.3(14.8) | - | Orthopaedic surgery | | - IV TXA - No TXA - - | - | Blood loss, general condition and vitals were assessed. |
| Geng 2017^1588^ | | - China - English - 2017 - Single-Centre - 100 - Patients who underwent spinal tuberculosis surgery | | 1. People suffering from the second surgery of spine tuberculosis; 2. Tranexamic acid allergy; 3. People who previously used warfarin and other anticoagulant drugs; 4. People with severe renal insufficiency, renal pelvis or ureteral solid lesions, diabetes and other diseases that may affect coagulation function; 5. People who had previous history of deep vein thrombosis. | | 49.1(4.4)  Vs  48.2(3.5) | - | Orthopaedic surgery | | - IV TXA - No TXA - - | - | Blood loss during operation, the postoperative drainage volume within 48 hours after operation, the postoperative haemoglobin (HB) and haematocrit (HCT). |
| Georgiadis 2013^1589^ | | - USA - English - 2013 - Single-Centre - 101 - Patients who underwent primary total knee arthroplasty | | Religious objection to autologous blood  transfusion, preoperative use of anticoagulant medication seven days prior to surgery, history of fibrinolytic disorder or blood dyscrasia, cerebrovascular accident (CVA), myocardial infarction (MI), New York Heart Association Class III or IV heart failure (NYHA III-IV), atrial fibrillation, history of deep vein thrombosis (DVT) or pulmonary embolus (PE), preoperative International Normalized Ratio (INR) N 1.4, activated partial thromboplastin time (aPTT) N 1.4 × normal, platelets b 140,000/mm3, or renal failure defined as creatinine N 1.1 mg/dL or glomerular filtration rate b 60 mL/min/1.73 m2. | | 67(9)  Vs  64.5(8.2) | - | Orthopaedic surgery | | - IV TXA - Placebo - - | - | - |
| Ghaffari 2012^1590^ | | - Iran - English - 2012 - Single-Centre - 100 - Patients undergoing on-pump coronary artery bypass graft surgery (CABG) | | History of haemorrhagic tendency and blood dyscrasia, history of Plavix use, known hepatic, renal, and metabolic diseases, use of other anti-coagulation drugs like Coumadin for valvular disease and arrhythmias and streptokinase, emergency surgery, rheumatic heart disease, known allergy to Aprotinin or Transamine and prohibition for their use on the grounds of acquired visual defects and retinal disease, subarachnoid haemorrhage, disseminated intravascular coagulation, gall bladder disease, leukaemia, embolization, and vein thrombosis | | 54.6(10.4)  Vs  54.2(9.7) | CV disease | Cardiac surgery | | - IV TXA - Placebo - - | - | The amounts of mediastinal and plural blood shed were measured after six, twelve, and twenty-four hours. Postoperative complications like postoperative myocardial  infarction (based on rise in cardiac enzyme, change in  ECG, and change in the ejection fraction estimated by echocardiography), neurological complications (estimated  by clinical examination and CT-scanning), redo-operations for surgical bleeding and pericardial effusion, kidney complications (rise in serum creatinine and low urinary output < 0.5 cc per minute), and other complications were studied. |
| Gill 2009^1591^ | | - USA - English - 2007 - Single-Centre - 10 - Patients who underwent total hip arthroplasty | | Patients in need of primary total hip arthroplasty or those with a known prosthetic infection, a bleeding or coagulation disorder, renal insufficiency (serum creatinine>two standard deviations for age), or history of deep venous thrombosis or pulmonary embolism. | | 66.6(7.5)  Vs  61.4(9.2) | - | Orthopaedic surgery | | - IV TXA - Placebo - Cell salvage | All blood transfusions given | Chest drain output at 48 hours. |
| Gillespie 2015^1592^ | | - USA - English - 2014 - Single-Centre - 111 - Patients who underwent total shoulder arthroplasty | | Revision surgery, history of cardiac disease, liver disease, renal disease, preoperative haemoglobin level <11.5 g/dL or haematocrit <35%, severe joint deformity, history of joint infection, history of bleeding or metabolic disorder, history of peripheral vascular disease, history of prior deep venous thrombosis (DVT) or pulmonary embolism (PE), any patient unwilling to accept a blood transfusion, and any patient with a documented allergy to TXA | | 67.5  Vs  66.4 | - | Orthopaedic surgery | | - IV TXA - Placebo - - | postoperative blood loss | Postoperative haemoglobin level. |
| Goobie 2018^1593^ | | - USA - English - 2018 - Single-Centre - 120 - Patients with adolescent idiopathic scoliosis who were between the ages of 10 and 18 years were included when they were scheduled for elective posterior instrumented spinal fusion at BCH. | | Haematological, coagulation, hepatic, or renal disorders and the administration of nonsteroidal anti-inflammatory drugs or acetylsalicylic acid within the previous 2 or 14 days, respectively, before surgery. | | 14.9(2)  Vs  14.7(1.8) | - | Orthopaedic Surgery | | - IV TXA - Placebo - Cell Salvage | Blood loss | Blood transfusion |
| Good 2003^1594^ | | - Sweden - English - 2003 - Single Centre - 51 - Patients with osteoarthritis and who had unilateral cemented total knee arthroplasty using spinal anaesthesia | | Patients with a history of coagulopathy, an abnormally great prothrombin or activated partial thrombin time, previous history of a thromboembolic event, treatment with aspirin or non-steroidal anti-inflammatory agents (NSAID) in the previous week, plasma creatinine greater than 115 mmol/litre in men and 100 mmol/litre in women, acute infection (e.g. with leucocytosis or fever), and malignant disease, patients with myocardial infarction in the preceding 12 months, those with unstable angina or coronary disease, patients given plasma or other treatment affecting coagulation during the perioperative period. | | 72(9.2)  Vs  72(8.5) | - | Orthopaedic surgery | | - IV TXA - Placebo - - | - | - |
| Greiff 2012^1595^ | | - Norway - English - 2008 - Single-Centre - 63 - Patients, 70 years or older, undergoing combined aortic valve replacement and CABG surgery | | Patients receiving treatment with heparin or low–molecular-weight heparin, oral anticoagulants, nonsteroidal anti-inflammatory drugs, platelet inhibitors other than aspirin, or systemic glucocorticoids. Patients with abnormal kidney function (serum creatinine >140 µmol/L) or liver dysfunction with international normalized ratio (INR) >1.5 | | 77(4)  Vs  77(5) | CV disease | Cardiac surgery | | - IV TXA - Placebo - Cell salvage | - | - |
| Guerreiro 2017^1596^ | | - Brazil - English - 2015 - Single-Centre - 43 - Patients who underwent total knee arthroplasty | | Patients with major deformities that would lead to bone cuts or release of a more extensive area of soft tissue; presence of inflammatory diseases; patients who had undergone previous surgeries of the same knee; use of anticoagulation medication up to seven days before surgery; and patients with history of atrial fibrillation, deep vein thrombosis or prior pulmonary embolism. | | 68.3(22.9)  Vs  69.1(19.2) | - | Orthopaedic surgery | | - IV TXA - Placebo - - | - | 1. Haemoglobin (Hb) levels preoperatively and 24 and 48 hours after surgery.  2. Reports of clinical flexion gain examination using a goniometer for evaluations 24 hours, 48 hours, 7 days, 21 days and 2 months after surgery.  3. Pain evaluation using a visual analogue scale (VAS)  4. Evaluations of knee function, preoperatively and 2 months after surgery, using the“WOMAC” instrument, were translated and validated for the Portuguese language |
| Gupta 2012^1597^ | | - India - English - 2011 - Single-Centre - 60 - Adult consented female patients, ASA class I and II, scheduled for elective radical surgery | | Patients with an allergy to medication (tranexamic acid), anaemia, preoperative hepatic or renal dysfunction, serious cardiac or respiratory disease, congenital or acquired coagulopathy or a history of deep vein thrombosis/thromboembolic disease | | 47.9(13.1)  Vs  46.3(18.2) | - | Plastic surgery | | - IV TXA - Placebo - - | - | Blood Loss  All patients’ preoperative and 12th hour  postoperative blood samples were analysed for haemoglobin, haematocrit, platelet count, prothrombin time (PT), activated partial thromboplastin time (aPTT), serum creatinine, fibrinogen, D-dimer and symptoms of pulmonary embolism such as dyspnea, haemoptysis, pleuritic chest pain, apprehension, tachypnea, tachycardia, rales etc. Doppler ultrasound of lower limbs was done daily in all patients for signs of deep vein thrombosis (DVT). |
| Guzel 2016^1598^ | | - Turkey - English - 2014 - Single-Centre - 100 - Patients who underwent primary unilateral total knee arthroplasty | | Patients with a history of venous thromboembolism, preoperative use of anticoagulants (acetylsalicylic acid, enoxaparin, or any other oral or intravenous agent), obvious anaemia or coagulopathy before surgery | | 66.5(5.1)  Vs  67(4.5) | - | Orthopaedic surgery | | - IV TXA - No TXA - Cell salvage | - | - |
| Haghighi 2017^1599^ | | - Iran - English - 2017 - Single-Centre - 38 - Patient who were undergoing surgery for femoral shaft fractures in trauma setting | | Coronary artery disease, history of arterial fibrillation, thrombophilia, chronic renal failure, haemoglobin<10 g/dl, thromboembolic episodes (DVT or pulmonary embolus), taking anticoagulant medication or oral contraceptive pills (OCP) and allergy to TA, presence of subarachnoid haemorrhage (SAH), pregnancy and breast feeding | | 65.1(4.8)  Vs  66.1(8.5) | Anaemia | Orthopaedic surgery | | - IV TXA - Placebo - - | - | The total amount of blood transfusion during operation and four hours after the surgery was measured |
| Hardy 1998^1600^ | | - Canada - English - 1994 - Single-Centre - 88 - patients older than 18 years scheduled to undergo - elective CABG | | Patients allergic to one of the study medications, patients seen with microscopic or macroscopic haematuria, or patients with an un-correctable defect of haemostasis preoperatively | | - | CV disease | Cardiac surgery | | - IV TXA - Placebo - Restrictive threshold | - | The total volume of mediastinal blood shed after the operation and collected until removal of drains (over 12 to 18 hours) was measured hourly by the ICU nurses. Transfusions of packed red blood cells (PRBCs) and haemostatic blood products (platelets, FFP, or cryoprecipitates) during and after the operation were recorded. |
| Hashemi 2011^1601^ | | - Iran - English - 2009 - Single-Centre - 100 - Patients undergoing on-pump coronary artery bypass grafting surgery (CABG) | | Patients with a history of haemorrhagic tendency and blood dyscrasia, history of Plavix usage, known hepatic, renal and metabolic diseases, use of other anti-coagulation drugs like Comadin for valvular disease and arrhythmias and streptokinase, emergency surgery, rheumatic heart disease, known allergy to Aprotinin or Transamine and prohibition for their use such as acquired visual defects and retinal disease, subarachnoid haemorrhage, disseminated intravascular coagulation, gall bladder disease, leukaemia, embolization, and vein thrombosis. | | 54.6(10.4) | CV disease | Cardiac surgery | | - IV TXA - Placebo - - | - | Post-operative complications like post-operative MI (based  on cardiac enzyme rising, ECG changing and EF changing estimated by echocardiography), Neurological complications (estimated by clinical examination and CT-Scanning), redo operation for surgical bleeding and pericardial effusion, kidney complication(rising of serum creatinine and low urinary out put under 0.5 cc per minute) and other complications were studied. |
| Hiippala 1995^1602^ | | - Finland - English - 1994 - Single-Centre - 28 - Patients underwent total knee arthroplasty | | Not stated | | 70(6.5)  Vs  70(3.7) | - | Orthopaedic surgery | | - IV TXA - Placebo - - | - | Blood loss during surgery, in the recovery room and on the surgical ward was recorded, together with the number of units of blood transfused in hospital |
| Hiippala 1997^1603^ | | - Finland - English - 1996 - Single-Centre - 77 - Patients scheduled for total knee arthroplasty | | Not stated | | 70(7)  Vs  69(5) | - | Orthopaedic surgery | | - IV TXA - Placebo - - | - | Perioperative blood loss gathered in surgical gauzes, suction reservoirs, and postoperative drainage system was measured. The number of transfusions given during hospitalization was registered. |
| Hooda 2017^1604^ | | - India - English - 2017 - Single-Centre - 60 - Adults undergoing elective craniotomy for meningioma excision | | Patients who refused to participate in the study or were allergic to tranexamic acid, had a history suggestive of bleeding diathesis, thromboembolic episode prior to surgery or family history of thromboembolism, patients on medication that could interfere with coagulation, epilepsy, plasma creatinine values more than 1.5 mg/dl and pregnant or lactating mothers | | 39.3(11.4)  Vs  41.6(11.2) | Cancer | Neurosurgery | | - IV TXA - Placebo - Cell salvage | intra-operative blood loss and transfusion requirements | The effect of tranexamic acid on the quality of surgical haemostasis, perioperative complications, length of hospital stay and neurological outcome were also evaluated. |
| Horrow 1990^1605^ | | - USA - English - 1990 - Single-Centre - 38 - Patients undergoing cardiac operation | | Patients with a history of bleeding disorder, those who received aspirin, warfarin, heparin, dipyridamole, streptokinase, NSAID within 7 days of surgery. | | 66(10) | CV disease | Cardiac surgery | | - IV TXA - Placebo - Restrictive threshold - Cell salvage | - | - |
| Horrow 1991^1606^ | | - USA - English - 1991 - Single-Centre - 81 - Patients undergoing cardiac surgery | | Patients who took warfarin or oestrogens within 7 days of surgery; had active haematuria, a serum creatinine concentration of 2 mg-/dl or more, or a personal or family history of abnormal bleeding; or underwent intra-aortic balloon counter-pulsation. | | 65(11)  Vs  64(10) | CV disease | Cardiac surgery | | - IV TXA - Placebo - - | - | Blood loss consisted of mediastinal tube drainage over 12 hours. Follow-up visits sought evidence of myocardial infarction and stroke. |
| Horrow 1995^1607^ | | - USA - English - 1995 - Single-Centre - 148 - Patients undergoing cardiac operation with extracorporeal circulation | | Patients who took warfarin or oestrogens within 7 days of surgery; had active haematuria, a serum creatinine concentration of 2 mg-/dl or more, or a personal or family history of abnormal bleeding; or underwent intra-aortic balloon counter-pulsation before surgery | | 65(10.4)  Vs  63(10.4) | CV disease | Cardiac surgery | | - IV TXA - Placebo - Restrictive threshold | - | The blood loss via mediastinal and pleural drains, transfusion of packed erythrocytes. |
| Hosseini 2014^1608^ | | - Iran - English - 2011 - Single-Centre - 71 - Patients who underwent off pump CABG | | Patients with clotting disorders, kidney failure (Cr> 1.7), allergy to tranexamic acid, consumption of antiplatelet drugs, prescription of heparin 48 h prior to surgery and patients with ejection fraction (EF) <40. | | - | CV disease | Cardiac surgery | | - IV TXA - Placebo - - | - | Patients were examined to find any deep veins thrombosis (DVT), renal failure and cerebrovascular accident (CVA). The amount of blood products including packed red blood cells (RBCs), FFP and platelets were recorded for each group. |
| Hou 2015^1609^ | | - China - Chinese - 2014 - Single-Centre - 40 - Patients who were candidates for unilateral cemented total knee replacement | | - | | 67.3(8.3)Vs  67.2(8.3) | - | Orthopaedic Surgery | | - IA TXA - IV TXA - Placebo - - | - | Blood loss, hidden blood loss, blood transfusion ratio and per capita of each group were compared. Clinical symptoms of pulmonary embolism and lower limb deep vein thrombosis were observed |
| Hsu 2015^1610^ | | - Taiwan - English - 2015 - Single-Centre - 60 - Patients underwent unilateral minimally invasive uncemented total hip arthroplasty | | Patients with a pre-operative level of haemoglobin was < 10 g/dl, or there was a history of ischaemic heart disease, myocardial infarction, cerebrovascular disease, thromboembolic disease or ipsilateral infection of the hip. | | 56.5(15.5)  Vs  59.5(15.2) | - | Orthopaedic Surgery | | - IV TXA - Placebo - - | - | Blood loss |
| Hu 2018^1611^ | | - China - Chinese - 2018 - Single-Centre - 105 - Patients with unilateral knee osteoarthritis undergoing total knee arthroplasty | | - | | 67.1(5.2)  Vs  68.2(5.5) | - | Orthopaedic Surgery | | - IV TXA (high dose) - IV TXA (low dose) - No TXA - - | - | The intraoperative blood loss, haemoglobin level at postoperative 24 and 48 hours, postoperative drainage volume and incidence of deep venous thrombosis were recorded. |
| Huang 2015^1612^ | | - China - Chinese - 2013 - Single-Centre - 60 - Patients who underwent total knee arthroplasty | | - | | - | - | Orthopaedic Surgery | | - IV TXA - No TXA - - | - | The amount of drainage, the total blood loss, the hidden blood loss, the postoperative Hgb, the amount of blood transfusion, the ratio of blood transfusion, and the incidence of vein thrombosis embolism (VTE) were compared between 2 groups. |
| Huang 2016^1613^ | | - China - English - 2014 - Single-Centre - 108 - Patients who underwent total knee arthroplasty | | Patients presenting with any blood disease, or diabetes, or any coagulation disorders or any history of thromboembolism. | | 71.1(5.8)  Vs  60.5(5.2) | - | Orthopaedic Surgery | | - IV TXA - Placebo - - | - | The volumes of blood loss, drainage and transfusion in each group were recorded to calculate the measured/hidden red blood loss (RBL). Haematocrit (Hct) was recorded preoperative­ly and 72 h postoperatively. |
| Huang 2017^1614^ | | - China - English - 2017 - Single-Centre - 150 - Patients who underwent primary total knee arthroplasty | | Patients scheduled for revision procedures, bilateral procedures, previous knee surgery, flexion deformity of >30 deg, varus-valgus deformity of >30 deg anaemia (haemoglobin [Hb] level of <12 g/dL for women and <13 g/dL for men), contraindications for the use of TXA (any history of blood clot events within 6 months), ASA grade IV, and coagulation disorders | | 66.2(8.3)  Vs  65.1(6.8)  Vs  65.8(6.3) | - | Orthopaedic Surgery | | - IV TXA + Tourniquet - IV TXA - No TXA - - | - | Total blood loss, hidden blood loss, maximum decline in Hb, transfusion rate, and CRP and IL-6 concentrations. The groups were also compared for swelling ratio, length of hospital stay, patient satisfaction, perioperative visual analog scale (VAS) pain score, cases of wound secretion, DVT and PE events, and other complications. |
| Husted 2003^1615^ | | - Denmark - English - 2003 - Single-Centre - 40 - Patients scheduled for primary total hip arthroplasty | | Patients with rheumatoid arthritis, malignancy, previous thrombo-embolic episodes, ischemic heart disease, previous subarachnoid bleeding, haematuria and body weight > 100 kg. | | 65  Vs  67 | - | Orthopaedic Surgery | | - IV TXA - Placebo - - | - | Perioperative blood loss and number of transfusions |
| Imai 2012^1616^ | | - Japan - English - 2011 - Single-Centre - 117 - Patients with osteoarthritis of hip, undergoing total hip arthroplasty | | Patients with a history of ischemic heart disease, severe chronic heart failure, hepatic dysfunction, chronic renal failure on haemodialysis, cerebral infarction, or bleeding disorder as well as those who were currently receiving anticoagulant therapy | | 60.2(13.3)  Vs  64.4(6.2)  Vs  62.2(8.7)  Vs  63.3(8.2)  Vs  63.3(8.2) | - | Orthopaedic Surgery | | - No TXA - IV TXA (1 Post-op dose) - IV TXA (2 Post-op doses) - IV TXA (Pre-op) - IV TXA (Pre-+Post-op) - No TXA - - | - | Intra- and Postoperative blood loss; Complications. |
| Ishida 2011^1617^ | | - Japan - English - 2011 - Single-Centre - 100 - Osteoarthritis patients with total knee arthroplasty | | Those with rheumatoid arthritis, revision TKA and simultaneous bilateral TKA | | 73.3(5)  Vs  73.5(6.1) | Anaemia | Orthopaedic Surgery | | - IV TXA - Placebo - - | - | - |
| Jansen 1999^1618^ | | - Belgium - English - 1999 - Single-Centre - 42 - Patients after total knee arthroplasty | | Rheumatoid arthritis, malignancy, previous thrombo-embolic episodes, ischemic heart disease, previous subarachnoid bleeding, haematuria and body weight > 100 kg. | | 70.7(4.5)  Vs  71(5) | - | Orthopaedic Surgery | | - IV TXA - No TXA - - | - | Blood Loss  Use of tranexamic acid for an effective blood conservation strategy after total knee arthroplasty |
| Jares 2003^1619^ | | - Czech Republic - English - 2003 - Single-Centre - 47 - Patients undergoing coronary artery bypass grafting on the beating heart | | Impaired renal function (Cr> 150mmol/l), haematological disease, Pre-op anaemia (Hb <11g/dl, Htc<32) and conversion to CPB | | - | CV disease | Cardiac surgery | | - IV TXA - Placebo - Restrictive threshold | - | Preoperative haematological variables, postoperative blood loss at 4 and 24 hours, transfusion requirements of packed red blood cells, and postoperative thrombotic events such as a myocardial infarction, stroke and pulmonary embolism were recorded. |
| Jaszczyk 2015^1620^ | | - Poland - English - 2015 - Single-Centre - 124 - Patients undergoing total cementless hip arthroplasty | | Patients with contraindications to intravenous TXA administration, i.e. allergy to TXA, deep vein thrombosis, a history of pulmonary embolism, arterial thrombosis, angina, a history of myocardial infarction or stroke, fibrinolysis secondary to consumption coagulopathy, severe kidney and liver failure, and a history of seizures. | | 66.1(28.1)  Vs  65.4(34) | - | Orthopaedic Surgery | | - IV TXA - No TXA - - | - | Intraoperative blood loss (volume of blood in the aspirator), postoperative blood loss (volume of blood drained), total perioperative blood loss, and the number of patients requiring transfusion as well as the number of thromboembolic complications in both groups. |
| Jendoubi 2017a^1621^ | | - Tunisia - French - 2017 - Single-Centre - 60 - Patients, ASA status I or II, undergoing endoscopic transurethral resections (TURP) | | Patients with ASA III or IV, with a known or suspected allergy to tranexamic acid (ATX) or to the excipient, presenting a medical contraindication to the use of ATX: history of convulsion, severe renal insufficiency (creatinine clearance <30 mL / min), coagulopathy, history of venous thromboembolism (deep vein thrombosis, pulmonary embolism) and / or arterial (angina, myocardial infarction, stroke, Acute leg ischemia), atrial fibrillation or acquired or congenital thrombophilia were not included in the study. | | 67.3(7.7)  Vs  71.1(9) | - | Urology | | - IV TXA - Placebo - - | - | Blood loss was evaluated in terms of reduction in the serum haemoglobin level |
| Jendoubi 2017b^1621^ | | - Tunisia - French - 2017 - Single-Centre - 71 - Patients, ASA status I or II, undergoing endoscopic transurethral resections (TURBT) | | Patients with ASA III or IV, with a known or suspected allergy to tranexamic acid (ATX) or to the excipient, presenting a medical contraindication to the use of ATX: history of convulsion, severe renal insufficiency (creatinine clearance <30 mL / min), coagulopathy, history of venous thromboembolism (deep vein thrombosis, pulmonary embolism) and / or arterial (angina, myocardial infarction, stroke, Acute leg ischemia), atrial fibrillation or acquired or congenital thrombophilia were not included in the study | | 64.5(11.3)  Vs  64(11.6) | - | Urology | | - IV TXA - Placebo - - | - | Blood loss was evaluated in terms of reduction in the serum haemoglobin level |
| Jimenez 2007^1622^ | | - Spain - English - 2007 - Single-Centre - 160 - Elective cardiopulmonary bypass patients | | No informed consent, age <  18 years, emergencies, off-pump cardiac surgery, chronic coagulopathy (prothrombin time [PT] <50% or international normalized ratio (INR) >2 and platelets <50,000/ mm3 or aggregation dysfunction), renal failure (creatinine >2 mg/dL), gross haematuria, TA hypersensibility, chronic hepatopathy (Child-B or higher), immunosuppression, endocarditis and post-operative sepsis within 24h | | 66(5.1)  Vs  67(6.6) | CV disease | Cardiac surgery | | - IV TXA - No TXA - - | - | Core body temperature, laboratory data (haematology, inflammation,  coagulation, and fibrinolysis), and hemodynamic parameters were recorded before intervention (baseline), on ICU admission after surgery (0 h), and at 4 h and 24 h post-CPB, once hemodynamic stability was confirmed. We also recorded blood loss (chest-tube drainage and haemoderivatives) at the above time points and on chest tubes removal. |
| Johansson 2005^1623^ | | - Sweden - English - 2005 - Single-Centre - 100 - Patients receiving total hip arthroplasty | | History or laboratory signs of bleeding disorders, malignancy and rheumatic joint disease, consumption of aspirin or NSAIDs within a week before surgery, history of coagulopathy or thrombo-embolic events and plasma creatinine levels above 115 μmol/L in men and 100 μmol/L in women. | | 68(8)  Vs  69(7) | - | Orthopaedic Surgery | | - IV TXA - Placebo - - | - | Total blood loss was calculated from the haemoglobin (Hb) balance. Volume and Hb concentration of the drainage was measured 24 h after the operation. Intraoperative blood loss was estimated volumetrically and visually. |
| Kakar 2009^1624^ | | - India - English - 2009 - Single-Centre - 25 - Total knee replacement patients | | Patients were excluded if they had one of the following criteria: known or suspected allergy to medications used (TAX, local anaesthetics, midazolam, pethidine, Propofol), inherited or acquired haemostatic diseases, abnormal coagulation screening tests (platelet count, prothrombin time, activated partial thromboplastin time), ingestion of aspirin or other nonsteroidal anti-inflammatory drugs within seven days of surgery, renal or hepatic insufficiency, pregnancy, history of deep venous thrombosis (DVT) or pulmonary embolism or history of ocular pathology or ophthalmological procedure other than corrective lenses. | | 63.1(16.8)  Vs  62.4(9.4) | - | Orthopaedic Surgery | | - IV TXA - Placebo - - | - | The postoperative blood loss, transfusion requirement, cost effectiveness and complications were noted. |
| Karaaslan 2015a^1625^ | | - Turkey - English - 2015 - Single-Centre - 81 - Patients who underwent arthroscopic anterior cruciate ligament reconstruction | | Bleeding or clotting disorders, preoperative anticoagulation therapy, abnormal coagulation profile, renal disorders or insufficiency, sickle cell disease, and allergy to local anaesthetics/TXA. | | 65.9(8)  Vs  65.6(7) | - | Orthopaedic Surgery | | - IV TXA - Placebo - - | - | The amount of drained blood. Thromboembolic and other complications were noted during the hospital stay |
| Karaaslan 2015b^1626^ | | - Turkey - English - 2015 - Single-Centre - 105 - Patients who underwent simultaneous bilateral total knee arthroplasty | | Bleeding or clotting disorder, preoperative anticoagulation therapy, abnormal coagulation profile, renal disorder or insufficiency, sickle cell disease, allergy to local anaesthetics/ TXA, significant preoperative pain (VAS score .5), large preoperative swelling (grade 3 or 4 effusion), or a revision case. | | 28.2(6.5)  Vs  28.3(9) | - | Orthopaedic Surgery | | - IV TXA - Placebo - - | grade of haemarthrosis, according to the classification of Coupens and Yates, and pain was measured by a visual analog scale (VAS) | VAS for pain score, haemarthrosis grade, range of motion (ROM), as well as the presence of any complications were documented. Patient satisfaction and knee function were recorded. |
| Karimi 2012^1627^ | | - Iran - English - 2012 - Single-Centre - 32 - Patients scheduled for elective bi-maxillary osteotomy | | Not stated | | 22.8(12.8)  Vs  23.9(12.2) | - | OMFS | | - IV TXA - Placebo - - | - | Intraoperative blood loss, pre and post-operative haemoglobin (Hb) and haematocrit (Hct) concentration, duration of surgery, hospital stay time, and rate of blood transfusion were recorded |
| Karski 2005^1628^ | | - Canada - English - 2005 - Single-Centre - 312 - Patients undergoing cardiac surgery | | Patients with a history of claustrophobia; known contraindications to magnetic resonance imaging (MRI); bleeding disorders; preoperative haemoglobin less than 135 g/L; symptomatic peripheral vascular disease; connective tissue disease; age older than 80 years; impaired renal function (creatinine 2.0 mg/dL); active liver disease; known allergies to TA, aspirin, or contrast dye (Omnipaque; Sterling Winthrop, Inc, Collegeville, Pa); or left ventricular function ejection fraction less than 20% | | 59.9(8.9)  Vs  60(8.3) | CV disease | Cardiac surgery | | - IV TXA - Placebo - - | Graft patency | - |
| Karski1995^1629^ | | - Canada - English - 1995 - Single-Centre - 98 - Patients undergoing cardiopulmonary bypass | | Not stated | | 63(1)  Vs  58(2) | - | Cardiac surgery | | - IV TXA - Placebo - - | - | - |
| Kaspar 1997^1630^ | | - USA - English - 1997 - Single-Centre - 27 - Patients underwent orthotopic liver transplantation | | Not stated | | - | - | Hepatobiliary Surgery | | - IV TXA - Placebo - Cell salvage | - | Intraoperative transfusion requirements were recorded during the procedure and for the first 24 h postoperatively. A record was kept of any intraoperative epsilon-aminocaproic acid administered for uncontrolled fibrinolysis. |
| Katoh 1997^1631^ | | - Japan - English - 1997 - Single-Centre - 62 - Patients undergoing either coronary artery bypass grafting or heart valve operation | | Not stated | | 62.9(1.7)  Vs  64.7(2.1) | CV disease | Cardiac surgery | | - IV TXA - Placebo - - | - | Mediastinal blood loss during the operation, but after discontinuation of CPB and drainage from mediastinal tubes for the first 24 hours after operation were measured. |
| Katsaros 1996^1632^ | | - USA - English - 1993 - Single-Centre - 210 - Patients who had first time CABG, valve replacement and reoperation with cardiopulmonary bypass | | Previous pulmonary embolism, Takayasu's arteritis, and known allergy to TXA | | 65(0.9)  Vs  63(1.2) | CV disease | Cardiac surgery | | - IV TXA - No TXA - Restrictive threshold | - | Shed mediastinal blood was measured for the first 24 hours postoperatively. |
| Kazemi 2010^1633^ | | - Iran - English - 2010 - Single-Centre - 64 - Patients who underwent total hip arthroplasty | | Patients with previous hip surgery, drug sensitivity, anaemia (haemoglobin <11.5 for females and <12.5 for males), congenital or acquired haemostatic disease, disturbed coagulation and platelet count, hepatic or renal failure, pregnancy, history of DVT (deep vein thrombosis) or embolism and atherosclerotic  vascular disease | | 46.6(16.2) | - | Orthopaedic Surgery | | - IV TXA - Placebo - - | - | 6- and 24-hour postoperative haemoglobin levels, intraoperative and postoperative bleeding, and allogenic blood transfusion |
| Keyhani 2016^1634^ | | - Iran - English - 2014 - Single-Centre - 80 - Patients who underwent primary total knee arthroplasty | | Patients with coagulation disorders, history of cardiovascular diseases, history of cerebrovascular disorders, history of thromboembolic problems, renal and hepatic diseases, pregnant women, anaemia, abnormal thrombin and prothrombin time, and abnormal platelet counts | | 68.4(10.4)  Vs  63.9(9) | - | Orthopaedic Surgery | | - IV TXA - No TXA - - | Volume of bleeding based on the amount of drainage, the level of Hb at 24 postoperative hours, the frequency of transfusion, and the number of packed red blood cells transfused. | All complications |
| Kim 2014^1635^ | | - Korea - English - 2014 - Single-Centre - 146 - Patients who underwent total knee arthroplasty | | Patients with a diagnosis other than primary OA, those with an acquired or congenital coagulopathy, those on current anticoagulation therapy, those with preoperative hepatic or renal dysfunction or severe ischaemic heart disease, and those with a history of thromboembolic disease | | 74.3(5.3)  Vs  73.9(5.1) | - | Orthopaedic Surgery | | - IV TXA - No TXA - Iron therapy - Restrictive threshold | Total blood loss and the allogenic transfusion rate. | rate of autologous  transfusion with preoperative autologous blood donation, blood loss via the drain, postoperative Hb drop, proportions of patients with the Hb level below the three cut-off values, namely 7.0, 8.0, and 9.0 g/dL, the incidences of symptomatic DVT and PE, and functional outcomes. |
| Kim 2016^1636^ | | - Korea - English - 2015 - Single-Centre - 48 - Patients who underwent posterior lumbar interbody fusion | | Patients with previous spinal surgery, previous or current bleeding or coagulation issues, established renal or hepatic diseases, or contraindication to antifibrinolytic agents | | 61(9)  Vs  65(7) | - | Orthopaedic Surgery | | - IV TXA - Placebo - - | amount of intraoperative and postoperative blood loss. | - |
| Kim 2018^1637^ | | - Korea - English - 2018 - Single-Centre - 48 - Patients who underwent craniosynostosis. | | Exclusion criteria were as follows: platelet count (PLT), < 50 × 10^3^/μL; prothrombin time (PT) or activated partial thromboplastin time (aPTT) > 1.5 times the reference value; history of convulsive seizure, epilepsy, or brain surgery; treatment with a non-steroidal anti-inflammatory agent within the previous 2 days; treatment with aspirin within 14 days prior to surgery; and known allergy to TXA. | | 1(0.3)  Vs  1.16(0.4) | - | Plastic surgery | | - IV TXA - Placebo - POC testing | blood loss during surgery |  |
| Kimenai 2016^1638^ | | - Netherlands - English - 2016 - Single-Centre - 500 - Adults aged 18 or older, scheduled for elective cardiac surgery on cardiopulmonary bypass | | Emergency cardiac interventions, minimally invasive surgery (port access surgery, thoracoscopic surgery or mini-sternotomy), off-pump procedures and patients with an increased or decreased bleeding tendency (Factor V Leiden thrombophilia, protein C deficiency, protein S deficiency, anti-thrombin deficiency and prothrombin mutation). | | 69(9.6)  Vs  68(9.6) | CV disease | Cardiac Surgery | | - IV TXA - Placebo - POC testing | 12-h postoperative blood loss | Number of transfusion-free patients, the amount of blood component transfusions given, the variables of routine coagulation tests, morbidity and in-hospital mortality. |
| Kojima 2001^1639^ | | - Japan - English - 2001 - Single-Centre - 22 - Patients undergoing cardiopulmonary bypass surgery | | Patients on medication likely to influence coagulation and fibrinolysis, as well as those with renal or hepatic dysfunction. | | 56(4)  Vs  60(2) | CV disease | Cardiac Surgery | | - IV TXA - Placebo - - | - | Intraoperative blood loss was assessed by estimated blood volume on drapes, weighing surgical gauzes, and measuring suction bottle returns. Postoperative blood loss during 24 h after surgery was measured from mediastinal and chest tube drainage following surgery. Blood products were transfused according to a standard protocol. |
| Kuitunen 2005^1640^ | | - Finland - English - 2005 - Single-Centre - 40 - Patients who underwent cardiac surgery | | Patients with pre-operative coagulation disorders; those taking medication with anticoagulants, acetosalicylic acid, platelet inhibitors or non-steroid anti-inflammatory drugs within the previous 5 days; those with renal insufficiency. | | 63(2)  Vs  65(2) | CV disease | Cardiac Surgery | | - IV TXA - Placebo - - | - | Perioperative blood loss |
| Kuitunen 2006^1641^ | | - Finland - English - 2006 - Single-Centre - 30 - Patients who underwent cardiac surgery | | Patients with preoperative coagulation disorders, renal or  hepatic failure or medication with Coumarin anticoagulants,  Heparin or Acetosalicylic acid within the previous 5 days. | | 57(16)  Vs  61(11) | CV disease | Cardiac Surgery | | - IV TXA - Placebo - POC testing | - | Perioperative blood loss |
| Kulkarni 2016^1642^ | | - India - English - 2016 - Single-Centre - 219 - Patients undergoing major head and neck cancer surgeries | | Patients with coagulopathy (partial prothrombin time >50 s, or international normalised ratio >1.5, platelets <50 × 10^9^/L), or those who had recent history of (<5 days) acetylsalicylic acid ingestion, patients on anticoagulant therapy (heparin received within 4 h or warfarin received 3 days pre-operatively) or those with peripheral vascular disease, pre-existing renal dysfunction (serum creatinine >1.2 mg/dL), liver dysfunction or known allergy to TA were excluded. | | 51.2(11.3)  Vs  50.6(11.6) | Cancer | OMFS | | - IV TXA - Placebo - POC testing - Restrictive threshold | reduction in blood loss | The number of patients needing transfusion. |
| Kumar 2013^1643^ | | - India - English - 2012 - Single-Centre - 200 - Patients undergoing percutaneous nephrolithotomy | | Patients with a serum creatinine greater than 1.5 mg/dl and specific contraindications to tranexamic acid, namely hypersensitivity to the drug, active intravascular clotting, acquired defective colour vision and subarachnoid haemorrhage. | | 37.9(10.8)  Vs  39.9(12.3) | Renal disease | Urology | | - IV TXA - No TXA - Restrictive threshold | perioperative total blood loss | Complications associated with PCNL, and to study the factors influencing blood loss and the safety of tranexamic acid in PCNL |
| Kundu 2015^1644^ | | - India - English - 2014 - Single-Centre - 60 - Patients undergoing unilateral total knee replacement | | Patients with history of previous ipsilateral knee surgery, suspected allergy to medication (TA, local anaesthetics, low-molecular weight heparin), anaemia (haemoglobin [Hb] <10 mg/dl for women and Hb <12 mg/dl for men), abnormalities in coagulation screening tests, aspirin intake within 7 days of surgery, renal (serum creatinine >2 standard deviation [SD] for age) or hepatic insufficiency, pregnancy and history of deep vein thrombosis (DVT) or pulmonary embolism, transient ischemic attack and stroke were excluded. | | 60.3(12.5)  Vs  59.6(12) | - | Orthopaedic Surgery | | - IV TXA - Placebo - Restrictive threshold | - | Number of transfusion given to the patients. |
| Lack 2017^1645^ | | - USA - English - 2017 - Single-Centre - 88 - Patients undergoing unilateral total knee replacement | | History of VTE or a baseline hypercoagulable state (ie, factor V Leiden and antiphospholipid antibody). | | 41.7(15.6)  Vs  39.7(16.1) | Anaemia | Orthopaedic Surgery | | - IV TXA - Placebo - Cell salvage | allogeneic blood transfusion | Estimate blood loss (EBL) and venous thromboembolism (VTE). |
| Lacko 2017^1646^ | | - Slovakia - English - 2017 - Single-Centre - 60 - Patients with knee osteoarthritis undergoing unilateral cemented total knee replacement | | Patients with known TA allergy, history of thromboembolism, cerebrovascular accidents, severe liver and kidney disease or blood clotting disorders. | | 67.5(7.7)  Vs  70(6.1) | - | Orthopaedic Surgery | | - IV TXA - No TXA - Restrictive threshold | - | Perioperative blood loss and blood loss to drainage for 24 hours postoperatively, time of operation and the occurrence of postoperative complications in the period of three months. |
| Langille 2013^1647^ | | - Canada - English - 2011 - Single-Centre - 28 - Patients undergoing functional endoscopic sinus surgery | | Patients that had a history of hypertension, renal failure, or vascular disease, or if they were American Society of Anaesthesiologists (ASA) class III or greater | | 43.5(13.6)  Vs  50(16.5) | - | ENT | | - IV TXA - Placebo - - | The Wormald grading scale. | The Peri-Operative Sinus Endoscopy (POSE) score, Lund-Kennedy endoscopic score, and total estimated blood loss. |
| Laoruengthana 2019a^1648^ | | - Thailand/USA - English - 2019 - Single-Centre - 228 - All patients with the diagnosis of primary osteoarthritis of the knee scheduled for primary unilateral TKA | | Patients with preoperative haemoglobin of less than 10 g/dL, previous history of a thromboembolic event, renal insufficiency, cardiovascular disease or cerebrovascular accident were excluded. Patients with a bleeding disorder and patients requiring anticoagulant therapy were also excluded. | | 64.4(7.1)  Vs  65.5(8.3)  Vs  63.3(8.4) | - | Orthopaedic Surgery | | - No TXA - IA TXA - IV TXA - - | - | Blood loss (CBL), drain volume (DV) and an average number of units of blood transfused (ANUBT). |
| Laoruengthana 2019b^1191^ | | - Thailand/USA - English - 2019 - Single-Centre - 226 - patients diagnosed with primary osteoarthritis of the knee and scheduled for primary unilateral TKA | | Patients with previous history of thromboembolic event, cardiovascular disease or cerebrovascular accident were excluded. Patients with preoperative haemoglobin of less than 10 g/dl, bleeding disorder, and patients requiring anticoagulant therapy were also excluded. | | 63.8(7.1)  Vs  64.8(8)  Vs  64(7.6) |  | Orthopaedic Surgery | | - No TXA - IA TXA - IV TXA - - | blood loss reduction | Effect on postoperative 56 pain, morphine consumption and knee flexion after TKA when using the TXA. |
| Later 2009^1649^ | | - Netherlands - English - 2006 - Single-Centre - 202 - Patients scheduled for low or intermediate risk first time heart surgery with use of cardiopulmonary bypass | | Patients with previous sternotomy, known bleeding disorders, an abnormal preoperative coagulation profile for reasons other than anticoagulant therapy, or treatment with antiplatelet agents within 5 days before surgery. | | 64.1(13)  Vs  65(11.2)  Vs  66.5(10.7) | CV disease | Cardiac surgery | | - IV TXA - Placebo - Aprotinin - Restrictive threshold; Cell salvage | postoperative blood loss and transfusion requirements | In-hospital mortality, morbidity, and length of intensive care and hospital stay. |
| Lee 2013a^1650^ | | - Korea - English - 2011 - Single-Centre - 72 - Osteoarthritis patients undergoing unilateral total knee arthroplasty | | Patients who had (1) planned bilateral knee or multiple joint replacements, (2) evidence of chronic or acute preoperative DVT on colour Doppler ultrasonography, (3) rheumatoid arthritis, haemophilia or post-traumatic osteoarthritis, (4) history of thromboembolic disease, (5) renal insufficiency (serum creatinine [1.5 mg/dL), (6) severe cardiovascular or respiratory disease, (7) severe ischaemic or heart disease, (8) acquired disturbances of colour vision, (9) preoperative anaemia (a haemoglobin value \11 g/dL in females and \12 g/dL in males), (10) congenital or acquired coagulopathy, or (11) preoperative use of anticoagulant therapy within 5 days before surgery | | 69.7(7.9)  Vs  69.2(7.7) | - | Orthopaedic Surgery | | - IV TXA - Placebo - Restrictive threshold - Cell salvage | - | Post-operative re-transfusion volume, allogenic transfusion  volume and drain amount were recorded for each patient. Ecchymosis around the operative leg was assessed. The level of haemoglobin, prothrombin time, activated partial thromboplastin time and D-dimer was recorded before and on the first, second and fifth days after operation. The incidence of total venous thromboembolism (DVT total, proximal and distal and symptomatic pulmonary embolism) and mortality was evaluated from all causes up to day 7. |
| Lee 2013b^1651^ | | - Korea - English - 2013 - Single-Centre - 68 - Adults, ASA status 1 and 2, undergoing primary unilateral cementless total hip replacement | | Patients older than 70 years, those with previous hip surgery, drug sensitivity, anaemia (haemoglobin [Hb] b 12 g/ dL for men and b 11 g/dL for women), coagulopathy, thrombocytopenia, hepatic or renal failure, history of deep vein thrombosis (DVT) or embolism, severe aortic or mitral valve stenosis, or neurological or cerebrovascular disease | | 51.4(11.2)  Vs  52.8(10.7) | - | Orthopaedic Surgery | | - IV TXA - Placebo - - | - | Intraoperative blood loss was measured using the difference between the weights of used gauze and the original unused gauze, in addition to the blood volume accumulated in suction bottles. Postoperative blood loss was considered to be the amount of blood accumulated in drainage bags. |
| Lee 2017^1652^ | | - Hong Kong - English - 2015 - Single-Centre - 189 - Patients with primary total knee replacement | | Patients with bilateral arthroplasty, thromboembolic diseases, history of clotting disorder or drug history of antiplatelet, anticoagulant, or deep vein thrombosis (DVT) prophylaxis in the perioperative period, complicated primary total hip arthroplasties with osteotomy, pre-existing implant removal or bone grafting, renal disease, and history of allergy to TXA. | | 70±8  Vs  68±8 | - | Orthopaedic Surgery | | - PO TXA - No TXA - Restrictive threshold | Hb drop | Intraoperative blood loss, drain output, total blood loss (TBL), hidden blood loss, transfusion requirement, thromboembolic complications, cerebrovascular or cardiovascular complications and 30-day mortality. |
| Lei 2017^1653^ | | - China - English - 2017 - Single-Centre - 77 - Patients undergoing hip surgery for intertrochanteric fracture | | Revisions, bilateral procedures, flexion deformity ≥30°, varus/valgus deformity ≥ 30°, patients with anaemia (<120 g/L for female, <130 g/L for male), pre-operative hepatic or renal dysfunction, serious cardiac or cerebrovascular problems, previous history of deep venous thrombosis or pulmonary embolism, congenital or acquired clotting disorders, contraindications for the use of TXA. | | 77.8(9.7)  Vs  79.1(6.5) | Anaemia | Orthopaedic Surgery | | - IV TXA - Placebo - - | - | Haemoglobin and haematocrit levels 1 day before surgery and on postoperative Day 1 and 3; duration of surgery; and visible blood loss collected with a sterile plastic foil, a funnel, and gauzes were measured. Complications associated with surgery—including hematoma, infection, deep vein thrombosis (examined by ultrasonography on day 3 post-operation), pulmonary embolism, myocardial infarction, ischemic cerebral infarction, respiratory infection, and renal failure—were also recorded. |
| Lemay 2004^1654^ | | - Canada - English - 2004 - Single-Centre - 39 - Patients undergoing primary unilateral total hip replacement | | History of previous ipsilateral  hip surgery, known or suspected allergy to medications used (TA, local anaesthetics, Midazolam, Fentanyl, Propofol, or Dalteparin), anaemia [haemoglobin (Hb) < 115 g/L for women, Hb < 130 g/L for men], inherited or acquired haemostatic diseases, abnormal coagulation screening tests (platelet count, prothrombin time, activated partial thromboplastin time),  ingestion of aspirin or other nonsteroidal anti-inflammatory drugs within seven days of surgery, renal (serum creatinine > two standard deviation for age) or hepatic insufficiency, pregnancy, history of deep venous thrombosis (DVT) or pulmonary embolism as well as a history of ocular pathology or ophthalmological procedure other than corrective lenses | | 59.7(10.3)  Vs  53.6(12.8) | - | Orthopaedic Surgery | | - IV TXA - Placebo - - | intraoperative and total blood losses | - |
| Li 2015^1655^ | | - China - Chinese - 2014 - Single-Centre - 224 - Patients who underwent unilateral primary total hip arthroplasty | | - | | 67.5(10.7)  Vs  67.4(8.8) | - | Orthopaedic Surgery | | - IV TXA - Placebo - - | - | Total blood loss, total volume of drainage and transfusion were recorded. Postoperative deep vein thrombosis and other complications was also measured. |
| Liang 2016^1656^ | | - China - English - 2015 - Single-Centre - 60 - Patients undergoing surgery for multilevel posterior lumbar degenerative procedures | | Allergy to TXA, anaemia (male haemoglobin <13 g/dl, female haemoglobin <12 g/dl), coagulopathy, treatment with anticoagulants or antiplatelet agents, history of thromboembolic events (deep vein thrombosis, ischemic heart disease, pulmonary embolism, transient ischemic attack, strokes, subarachnoid haemorrhage), renal impairment (creatinine >2.0 mg/dl), chronic liver disease, and pregnancy. We also excluded patients more than 65 years of age because elderly patients usually limited their activities and are more prone to have deep vein thrombosis. | | 51.1(10.7)  Vs  53.5(10.2) | - | Orthopaedic Surgery | | - Top TXA - Placebo - Restrictive threshold | - | Data were collected on demographics, pre-operative investigations, blood loss, and blood products transfused during surgery. |
| Lin 2011^1657^ | | - Taiwan - English - 2009 - Single-Centre - 100 - Patients who underwent minimally invasive total knee arthroplasty | | Patients with thrombocytopenia or haemophilia, prior surgery of the affected knee, haemoglobin (Hb) less than 10 g/dL on the day of admission, a history of thromboembolic disease or lifelong warfarin therapy for thromboembolism prophylaxis, declined to participate in the study, who did not withhold use of aspirin for 1 week before admission. | | 69.2(6.3)  Vs  68.3(8.4) | - | Orthopaedic Surgery | | - IV TXA - Placebo - - |  | Data were collected on demographics, pre-operative investigations, blood loss, and blood products transfused during surgery. |
| Lin 2012^1658^ | | - Taiwan - English - 2010 - Single-Centre - 151 - Patients undergoing unilateral minimally invasive TKR | | Patients with a history of previous surgery on the same knee, thromboembolic disease, myocardial infarction, cerebrovascular disease or a pre-operative haemoglobin < 10 g/dl were excluded from the trial. | | 69.8(7.5)  Vs  70.6(8)  Vs  69.7(7.8) | - | Orthopaedic Surgery | | - IV TXA (2 dose) - IV TXA (1 dose) - Placebo - Restrictive threshold | - | The volume of blood drained was recorded every two hours during the first eight post-operative hours, and then every eight hours until the drains were removed on the second post-operative day. The haemoglobin and haematocrit were checked on the first, second, and fourth days after operation. |
| Lin 2015^1659^ | | - Taiwan - English - 2013 - Single-Centre - 120 - Patients who underwent total knee arthroplasty | | (1) allergy to TXA; (2) a known history of thromboembolic disease; (3) preoperative renal or hepatic dysfunction; (4) cardiovascular disease (a history of myocardial infarction or angina); (5) cerebral vascular disease (a history of stroke); (6) preoperative anaemia (a haemoglobin (Hb) value less than 11 g/dL in female and less than 12 g/dL in male); and (7) preoperative coagulopathy (a platelet count less than 150,000/mm3 or an international normalized ratio greater than 1.4) | | 71(7.2)  Vs  70.7(8.2)  Vs  69.7(8) | - | Orthopaedic Surgery | | - Top TXA - IV TXA - Placebo - - | - | Postoperative Hb levels, Hb drop, total drain amount, total blood loss, and transfusion rate. |
| Liu 2017^1660^ | | - China - English - 2015 - Single-Centre - 224 - Patients undergoing total knee arthroplasty - 1) Participants: patients undergoing primary THA. 2) Intervention: combined topical with intravenous TXA. 3) Comparison: IV TXA alone. 4) Outcomes: the primary outcomes included total blood loss, hidden blood loss, transfusion rate, and postoperative complications (including DVT/pulmonary embolism (PE)). Secondary outcomes included haemoglobin drop and length of hospital stay. 5) Study: only RCTs were included. | | Articles that without the outcome measures of interest. 2) Quasi-RCT or non-RCT. 3) Retrospective studies, letters, comments, editorials and practice guidelines. | | 63.5(6.5)  Vs  62.9(7)  Vs  61.8(6.4) | - | Orthopaedic Surgery | | - IV TXA (low dose) - IV TXA (high dose) - Placebo - POC testing | - | The intraoperative blood loss, postoperative drainage volume, occult blood loss, blood transfusion rate, and blood transfusion volume in each group were recorded |
| Lopez-Hualda 2018^1661^ | | - Spain - English - 2018 - Single-Centre - 90 - Patients scheduled for unilateral total knee arthroplasty | | The exclusion criteria were having had previous coagulopathies and receiving chronic anticoagulant treatment. | | 73.1(7.3)  Vs  72.9(7.1)  Vs  72.4(6.9) | - | Orthopaedic Surgery | | - IV TXA - Top TXA - No TXA - Restrictive threshold | - | Blood loss and drain outputs |
| Lundin 2014{Lundin, 2014 #33101} | | - Sweden - English - 2014 - Single-Centre - 100 - Women undergoing radical debulking ovarian cancer surgery | | Patients with an allergy to tranexamic acid; treatment with anticoagulants within the past month; a history or present laboratory signs of bleeding disorders, coagulopathy or thromboembolic events; a history of myocardial infarction within the last year; present unstable angina or severe coronary disease; reduced renal function with plasma creatinine levels above  250 µmol/L, and severe psychiatric or mental disorder | | 60.2(14.1)  Vs  66.2(12.7) | Cancer | Oncological surgery | | - IV TXA - Placebo - - | Blood loss and red blood cell transfusions. |  |
| Luo 2019^1663^ | | - China - English - 2017 - Single-Centre - 90 - (1) had intertrochanteric fracture (extracapsular fractures of AO/OTA types 31-A1 to 31-A3) treated with PFNA, (2) closed fracture with low-energy damage, and (3) age ≥60 years. | | (1) preoperative examination revealed DVT; (2) they had any contraindication for anticoagulation therapy; (3) they had a pathological fracture; (4) they had one of the following diseases in the preceding year: myocardial infarction, cerebral infarction, coronary syndrome, DVT, or pulmonary embolism; (5) the duration from injury to operation was >3 weeks; (6) they had allergy to TXA; (7) patients who had adverse drug reactions when using TXA and stopped the medication; (8) they had multiple fractures, with the other fracture also needing surgical treatment; (9) preoperative hemoglobin (Hb) was <8 g/dL; (10) closed reduction failed, and therefore open reduction was performed; and (11) there was any change in the fixation method or if, intraoperatively, the decision was made to perform arthroplasty. | | 75.1(8)  Vs  76.1(9.3) | - | Orthopaedic Surgery | | - IV TXA - Placebo - - | perioperative blood loss | Postoperative transfusion rate, postoperative haemoglobin level, and length of the hospital stay. The safety outcomes were the incidence of thrombotic events and the mortality rate within 6 weeks after surgery. |
| Macgillivray 2011^1664^ | | - UAE - English - 2011 - Single-Centre - 60 - Patients presenting for concurrent total knee arthroplasty | | Patients with known allergy to TXA, a history of hepatic or renal dysfunction, severe cardiac or respiratory disease (myocardial infarction within 6 months, unstable angina, aortic or mitral valvular stenosis), previous stroke, congenital or acquired coagulopathy, or history of thromboembolic disease. | | 66(7.3)  Vs  65(4.3)  Vs  62(4.3) | - | Orthopaedic Surgery | | - IV TXA (low dose) - IV TXA (high dose) - Placebo - Cell salvage | - | Risk of RBC transfusion  Perioperative blood loss |
| Maddali 2007^1665^ | | - Oman - English - 2005 - Single-Centre - 222 - Patients undergoing on-pump primary coronary bypass surgery | | Patients requiring concomitant non-coronary procedures and those with a history of bleeding diathesis or known coagulation factor deficiency | | 57.1(8.9)  Vs  58.2(8.3) | CV disease | Cardiac surgery | | - IV TXA - Placebo - POC testing | - | Postoperative drainage and transfusion requirements were measured in all patients. |
| Malhotra 2011^1666^ | | - India - English - 2011 - Single-Centre - 50 - Patients undergoing total hip arthroplasty | | Patients with a history of severe ischemic heart disease, chronic renal failure, cirrhosis of the liver, and bleeding disorders, as well as those who were currently receiving anticoagulant therapy | | 52.6(24.4)  Vs  54.7(22.9) | - | Orthopaedic Surgery | | - IV TXA - Placebo - - | - | The intraoperative and postoperative blood loss and the number of blood transfusions required were recorded. |
| Maniar 2012^1667^ | | - India - English - 2011 - Single-Centre - 200 - Patients undergoing knee arthroplasty | | Known allergy to tranexamic acid; preoperative hepatic or renal dysfunction; serious cardiac or respiratory disease; congenital or acquired coagulopathy; and a history of thromboembolic disease. | | 67.3(9.1)  Vs  68.3(8)  Vs  67.4(8.4)  Vs  66.8(7)  Vs  66.2(7.2) | - | Orthopaedic Surgery | | - IV TXA (intra-op) - IV TXA (pre-op + intra-op) - IV TXA (intra-op+post-op) - IV TXA (all 3 doses) - IV TXA (local application) - No TXA - - | - | Drain loss and total blood loss. We recorded blood transfusions for quantity and determined the haemoglobin concentration of each transfused unit. |
| Mansouri 2012^1668^ | | - Iran - English - 2012 - Single-Centre - 90 - Patients underwent valvular heart surgery (i) age >18 years; (ii) not pregnant; (iii) elective operation; (iv) absence of known or suspected allergy to Aprotinin or tranexamic acid; (v) absence of previous sternotomy, pre-existing renal dysfunction (serum creatinine >1.36 mg/dl), preoperative coagulation defects [prothrombin time (PT) >18 s or activated partial prothrombin time (aPTT) >50 s or platelet count <100 × 109/l], recent (<5 days) ingestion of acetylsalicylic acid, thrombolytic therapy (streptokinase, Urokinase or tissue plasminogen activator <1 day preoperatively), anticoagulant therapy (heparin <4 h preoperatively or warfarin <3 days preoperatively), autologous pre-donation of blood, history of thrombotic events such as deep vein thrombosis, disseminated intravascular coagulation and cerebral thromboembolic accident in the previous 6 months, or unstable angina | | (i) Pump time >120 min; and (ii) bleeding with a surgical source (identified at postoperative reoperation). | | 48.3(15.5)  Vs  42.5(16.4)  Vs  48.3(13.5) | CV disease | Cardiac surgery | | - IV TXA - Aprotinin - Placebo - Cell salvage | - | The major parameters that we evaluated in this study were as follows: chest-tube drainage, the type and number of units of blood and blood products transfused, coagulation tests and haemoglobin/haematocrit and platelet count preoperatively, 6 and 24 h after ICU admission, neurological deficits (drowsiness, agitation, focal neurological deficit, convulsion and coma), renal failure and plasma FDP concentration at the end of surgery. In addition, we assessed demographic items, the number of exchanged heart valves, the length of stay in the ICU bedridden and the hospital mortality. |
| Martin 2014^1669^ | | - USA - English - 2012 - Single-Centre - 100 - Patients who underwent total hip and total knee arthroplasty | | Revisions, bilateral joint arthroplasty procedures, known hypersensitivity to TXA or its ingredients, active intravascular clotting disorders, and acute subarachnoid haemorrhage. Patients with a history of DVT or PE | | 63.9(15.9)  Vs  62.9(14.6) | - | Orthopaedic Surgery | | - IV TXA - Placebo - Restrictive threshold | the maximum decline in postoperative  haemoglobin (g/dL) | the number of patients who received packed red blood cell transfusions, the average length of hospital stay, number of postoperative wound infections, number of patients diagnosed with deep vein thrombosis (DVT) or pulmonary embolism (PE) within 30 days of surgery. |
| McConnell 2011^1670^ | | - UK - English - 2008 - Single-Centre - 44 - Patients who had cemented total hip arthroplasty | | If there were contraindications to giving the medications in the study: known allergy to the medications used, including allergy to aspirin; previous reaction to blood products; ethical/religious objection to receiving blood products; or previous thromboembolism | | - | - | Orthopaedic Surgery | | - IV TXA - Placebo - Cell salvage | - | total blood volume |
| Mehr-Aein 2007^1671^ | | - Iran - English - 2007 - Single-Centre - 200 - Patients undergoing coronary artery bypass | | Patients undergoing redo operation, emergency CABG, off-pump CABG, haemoglobin < 10 g/dL, platelet count < 100 K·μ/L, a known coagulopathy disorder, and renal insufficiency. | | 54(7)  Vs  52(8) | CV disease | Cardiac surgery | | - IV TXA - No TXA - Cell salvage | - | Blood loss, whole blood transfusions. |
| Melo 2017^1672^ | | - Brazil - English - 2017 - Single-Centre - 42 - Patients who underwent primary total hip arthroplasty | | Patients younger than 18 years Chronic kidney disease (creatinine clearance less than  60 mL/min m^2^)  Bleeding disorders or thrombophilia;  Trauma; Low platelet count (preoperative platelet count less than 150 000) Chronic anaemia (preoperative haemoglobin less than 10 g/dL) Refusal to consent | | - | - | Orthopaedic Surgery | | - IV TXA (low dose - IV TXA (high dose) - No TXA - - | - | The mean blood loss |
| Meng 2014^1673^ | | - China - English - 2013 - Single-Centre - 60 - patients diagnosed with BPH and undergoing TURP | | Preoperative heart and cerebrovascular diseases, renal insufficiency, kidney stones, high risk or a history of thrombosis, long-term anticoagulant therapy, preoperative long-term bed confinement, prostate cancer diagnosis, blood coagulation dysfunction. Patients were also excluded if they had taken 5-a reductase inhibitors, aspirin or warfarin prior to surgery. | | 71.4(5.4)  Vs  70.7(8.5) | - | Urology | | - IV TXA - Placebo | - | Intraoperative and postoperative bladder irrigation volumes and blood loss volumes |
| Menichetti 1996^1674^ | | - Italy - English - 1996 - Single-Centre - 96 - Patients who underwent coronary artery bypass surgery | | 1) emergency operation 2) EF<4% 3) Pre-op Hct <38% 4) Allergy to anti-fibrinolytics 5) thromboembolic disease treated with anticoagulant therapy 6) patients with peripheral vascular disease 7) renal insufficiency (Cr >1.5 mg/dl 8) LFT derangement 9) coagulopathy 10) re-do procedures. 11) Use of acetyl-salicylic acid or dipyridamole within two week of operation date. | | 55.2(8.6)  Vs  61(9.7) | CV disease | Cardiac surgery | | - IV TXA - Aprotinin - Epsilon aminocaproic acid - No TXA - Restrictive threshold | - | Postoperative bleeding and need for transfusion showed that the aprotinin group had significantly lower mediastinal bleeding. |
| Miller 1980^1675^ | | - UK - English - 1980 - Single-Centre - 100 - Patients undergoing - transurethral prostatectomy (92) or endoscopic - bladder tumour resection | | Not stated | | - | - | Urology | | - PO TXA - No TXA - - | - | Four weeks after operation all patients were reviewed and the severity of haemorrhage and its timing were recorded on standard pro formas. Details of duration of haemorrhage and the association of clots were also noted. |
| Min 2015^1676^ | | - China - Chinese - 2015 - Single-Centre - 64 - Patients with primary osteoarthritis undergoing a unilateral total knee arthroplasty | | - | | 70.3(5.2)  Vs  69.2(7.4) | - | Orthopaedic Surgery | | - IV TXA - Placebo - - | - | Intraoperative blood loss, postoperative blood loss, postoperative haemoglobin levels, amount of blood transfusion, and number of patients requiring blood transfusion were compared. Fibrinogen, prothrombin time and other coagulation indicators were also examined before operation and 3 hours after operation. |
| Mirmohammadsadeghi 2018^1677^ | | - Iran - English - 2018 - Single-Centre - 125 - Inclusion criteria were patients undergoing CABG surgery alone, interrupting aspirin 3 days and Plavix at least 5 days before surgery, lack of consuming any other anticoagulant drugs such as heparin or warfarin, lack of coagulation and bleeding disorders, and lack of liver and kidney disease. | | Exclusion criteria were complex surgery, emergency surgery, and anticoagulation therapy before surgery, and having haemoglobin lower than 8 g per decilitre before surgery. | | 63.3(8)  Vs  62.5(8.5) | CV disease | Cardiac surgery | | - Top TXA - Placebo - - | - | 24 and 48 h chest tube drainage, haemoglobin decrease and packed RBC transfusion |
| Mohib 2015^1678^ | | - Pakistan - English - 2014 - Single-Centre - 100 - Patient who underwent for intertrochanteric fracture | | - | | 69(10)  Vs  70(9.4) | - | Orthopaedic Surgery | | - IV TXA - Placebo - Restrictive threshold | - | Numbers of blood transfusions required postoperatively were noted based on the postoperative haemoglobin readings. |
| Molloy 2007^1679^ | | - UK - English - 2005 - Single-Centre - 100 - Patients who underwent total knee replacement | | previous surgery to the knee, with the exception of meniscectomy, bleeding disorders, platelet or bone-marrow disorders, a level of creatinine > 250 μmol/l since this is a contraindication to the administration of tranexamic acid, or a history of thromboembolism. | | - | Anaemia | Orthopaedic Surgery | | - IV TXA - No TXA - - | - | Total blood loss. The number of units of blood transfused during the hospital stay was recorded, along with any complications attributed to the surgery or occurring within 90 days of the operation. |
| Motififard 2015^1680^ | | - Iran - English - 2013 - Single-Centre - 90 - Patients undergoing total knee arthroplasty | | Patients with previous history of cerebrovascular disease, thromboembolism, myocardial infarction, and those who were candidates for bilateral TKA | | 67(8)  Vs  65.6(4.9) | - | Orthopaedic Surgery | | - IV TXA - Placebo - - | Level of Hb 48 hours after surgery. | Hb levels, 6 and 24 hours after surgery, drain output during the first 48 hours after surgery, and blood product administration after surgery and duration of hospitalization. |
| Mu 2019^1681^ | | - China - English - 2017 - Single-Centre - 150 - Patients diagnosed with lumbar degenerative disease and who had no history of posterior lumbar decompression or interbody fusion with pedicle screw fixation | | 1) history of thromboembolism or evidence of existing thrombus on preoperative vascular B-mode ultrasound; 2) use of antiplatelet aggregation drugs within 6 months or symptom of coagulation dysfunction before surgery; 3) internal diseases such as cardiovascular disease, hepatorenal insufficiency, and hematologic system disease; 4) confirmed allergy history or high risk of allergy to TXA; 5) history of smoking (more than 10 cigarettes per day for more than 6 months) or drinking (at least 50 g of liquor with an alcohol volume ratio over 40% per day for more than 3 months) with unsuccessful cessation within 6 months before surgery; 6) a body mass index less than 18.5 or over 30.0; and 7) an inability to understand the study protocol after explanation or an unwillingness to participate. | | 54.2(7.3)  Vs  51.7(8.1)  Vs  52.5(6.7) | - | Orthopaedic Surgery | | - IV TXA - Top TXA - Placebo - - | - | blood biochemical indices, blood loss, and the number of blood transfusions |
| Murphy 2006^1682^ | | - UK - English - 2006 - Single-Centre - 100 - Patients who underwent off-pump CABG surgery | | Advanced chronic renal insufficiency (creatinine  >2 mg/dL), active chronic hepatitis or cirrhosis, neurologic dysfunction, hematologic disorders and the use of Clopidogrel pre-operatively. | | 64.9(7)  Vs  65.8(8.7) | CV disease  Renal disease | Cardiac surgery | | - IV TXA - No TXA - Cell salvage | - | Homologous packed red cells as blood replacement therapy |
| Myles 2017^1683^ | | - Australia - English - 2017 - Multi-Centre - 4631 - Patients undergoing CABG surgery | | 1. Poor (English) language comprehension  2. Clinician preference for antifibrinolytic therapy  3. Urgent surgery for unstable coronary syndromes where for clinical reasons antiplatelet  medication cannot be discontinued  4. Active peptic ulceration  5. Allergy or contraindication to aspirin or tranexamic acid  6. Aspirin therapy within 4 days of surgery  7. Warfarin or Clopidogrel therapy within 7 days of surgery, or GIIb/IIIa antagonists within 24 h of  surgery  8. Thrombocytopenia or any other known history of bleeding disorder  9. Severe renal impairment (serum creatinine >250 µmol/l, or estimated creatinine clearance <25 ml/min)  10. Recent haematuria  11. Thromboembolic disease relating to: history of postoperative or spontaneous pulmonary embolism, spontaneous arterial thrombosis or familial hypercoagulability (e.g. lupus anticoagulant, protein C deficiency)  12. Pregnancy | | 66.8(9.8)  Vs  67(9.6) | CV disease | Cardiac surgery | | - IV TXA - No TXA - - | composite of death and thrombotic complications (nonfatal myocardial infarction,  stroke, pulmonary embolism, renal failure, or bowel infarction) within  30 days after surgery. | Death, nonfatal myocardial infarction, stroke, pulmonary embolism, renal failure, bowel infarction, reoperation due to major haemorrhage or cardiac tamponade, and a requirement for transfusion. |
| Na 2016^1684^ | | - Korea - English - 2016 - Single-Centre - 55 - Patients undergoing total hip replacement arthroplasty | | Pre- and intra-operative blood transfusion; venous thrombo-embolism; coagulopathy; preoperative haemoglobin of < 10 g/dl; haematological or renal disease; and antiplatelet or anticoagulant medications, including regular and long-term use of nonsteroidal anti-inflammatory drugs within one month of surgery. | | 53(11.1)  Vs  50(11.8) | - | Orthopaedic Surgery | | - IV TXA - Placebo - POC testing - Restrictive threshold | Results of the ROTEM analyses. | Patients’ characteristics; surgery- and anaesthesia related information; laboratory results (haemoglobin, haematocrit, platelets, PT-INR, aPTT and fibrinogen); input (infused volume of crystalloid and colloid); output (intra- and postoperative blood loss and urine output); and transfusion of blood components. |
| Nagabhushan 2017^1685^ | | - India - English - 2017 - Single-Centre - 50 - The patients with American society of Anaesthesiologists (ASA) physical status I and II, aged 18-65 yr, scheduled for elective lumbar spine single level fusion surgery expected to last less than 3 hours, under general anaesthesia were included in the study. | | Patients known to have any coagulation disorder, altered liver and renal parameters, and on anticoagulants, antiplatelet medications were excluded from the study. | | 49.6(9.7)  Vs  48.8(12.8)  Vs  49.2(10)  Vs  51.7(9.7) | - | Orthopaedic Surgery | | - IV TXA - Batroxobin - IV TXA + Batroxobin - Placebo - - | - | Intraoperative and postoperative blood loss, haematocrit, allogenic blood transfusion, and deep vein thrombosis (DVT), postoperatively. |
| Napoli 2016^1686^ | | - Argentina - Spanish - 2016 - Single-Centre - 62 - Patients who underwent primary hip and knee arthroplasties | | - | | 66(9.9)  Vs  67.2(8.5) | - | Orthopaedic Surgery | | - IV TXA - Placebo - Restrictive threshold | - | Preoperative and postoperative haematocrit and haemoglobin, days of stay in hospital and number of red cell unit transfusion, complications and adverse effects. |
| NCT00824564 | | - India - English - 2011 - Single-Centre - 82 - Patient undergoing surgery for fracture shaft of femur - High risk of publication bias and no peer-review led to exclusion from the analysis | | - Patients with a platelet count less than 100, 000/mm3 or history of thrombocytopenia  - Patients with known coagulopathy  - Patients with anaemia (haemoglobin levels less than 8 mg/dl or haematocrit <24%)  - Patients with documented DVT or PE at screening or in past three months  - Patients having known hypersensitivity to tranexamic acid or any other constituent of the product  - Patients with any associated major illness (e.g., severe cardiac or respiratory disease)  Anticoagulants (other than LMWH or heparin in prophylactic doses to prevent deep vein thrombosis), direct thrombin inhibitors or thrombolytic therapy administered or completed within last week | | 33.7(12.6)  Vs  36.6(17) | - | Orthopaedic Surgery | | - IV TXA - Placebo - - | Total blood loss | Intra-operative Blood Loss; Post-operative Blood Loss; Total Blood Loss Assessed by Gross’ Formula; Number of Participants Receiving Transfusions; Change From Baseline in Haemoglobin Levels at End of Surgery, 1 hr Post-surgery, and Mornings of Day 1, Day 2, Day 4, Day 7 or Early Termination (ET) Post-surgery; Number of Participants With Deep Vein Thrombosis (DVT) Post Surgery; |
| NCT00827931 | | - India - English - 2012 - Single-Centre - 94 - Patient undergoing major abdominal surgery (Biliary strictures, Pancreatico-duodenectomy, Esophagectomy, Total proctocolectomy, Hemicolectomy, Gastrectomy, Other major abdominal surgeries with similar expected blood loss) - High risk of publication bias and no peer-review led to exclusion from the analysis | | - Patients with a platelet count less than 100, 000/mm3 or history of thrombocytopenia.  - Patients with known coagulopathy.  Patients with anaemia (haemoglobin levels less than 8 mg/dl)  - Patients with documented DVT or PE at screening or in past three months.  - Patients with any associated major illness (e.g., severe cardiac or respiratory disease).  - Anticoagulants (other than LMWH or heparin in prophylactic doses to prevent deep vein thrombosis), direct thrombin inhibitors or thrombolytic therapy administered or completed within last week | | - | - | General Surgery | | - IV TXA - Placebo - - | Reduction in postoperative blood loss | Intra-operative Blood Loss; Total Blood Loss; Total Blood Loss as Assessed by the Gross’ Formula; Percentage of Participants Receiving Transfusions; Haemoglobin Levels; Number of Participants With Deep Vein Thrombosis (DVT) Post Surgery; |
| Neilipovitz 2001^1687^ | | - Canada - English - 2001 - Single-Centre - 40 - Patients with scoliosis undergoing posterior spinal fusion surgery | | Patients with a history of a bleeding disorder, a low platelet count (,150), abnormal partial thromboplastin time or international ratio test, body mass index .30 kg/m2, previous thromboembolic event, or a family history of thromboembolism | | 14.1(2.1)  Vs  13.7(2.5) | - | Orthopaedic Surgery | | - IV TXA - Placebo - Cell salvage | - | Total amount of blood transfused in the perioperative period, thrombotic complications. |
| Niskanen 2005^1688^ | | - Finland - English - 2003 - Single-Centre - 39 - Patients with primary cemented hip arthroplasty for osteoarthritis | | Patients with rheumatoid arthritis and osteonecrosis, Patients with known coagulation disturbances including thromboembolic events, Patients using warfarin related preparations, or with allergy to tranexamic acid, or with signs of renal insufficiency | | 66(9.1)  Vs  65(8.2) | - | Orthopaedic Surgery | | - IV TXA - Placebo - - | Blood loss during the operation and the amount of drainage after the operation. | The amount of transfused units of red cells, wound leakage postoperatively, swelling and ecchymosis of the thigh, haematocrit, and possible complications. |
| Nouraei 2013^1689^ | | - Iran - English - 2013 - Single-Centre - 80 - Patients who underwent CABG surgery | | Age of more than 75 years; advanced liver, kidney, lung, or severe peripheral vascular disease; internal carotid artery narrowing of >50%; recent myocardial infarction, New York Heart Association class 3 and 4; CABG with valve operation; insulin-dependent diabetes mellitus; re-exploration; history of seizure disorder; haemoglobin (Hb) levels of <10 g/dL or haematocrit (Hct) levels of <30%; and anticoagulation usage 5 days before surgery. | | 60(9.6)  Vs  59.6(10) | CV disease | Cardiac surgery | | - Top TXA - Placebo - - | Volume of mediastinal bleeding | Units of transfused packed red cells, FFP, and platelet concentrate |
| Nuttall 2000^1690^ | | - USA - English - 2000 - Single-Centre - 160 - Cardiac surgery patients at high risk for bleeding | | Patients with histories of bleeding or a platelet disorder, prothrombin time (PT). 15.0 s, blood urea nitrogen level greater than 100 mg/dl, or a recent history of thrombolytic, warfarin, or heparin therapy. Patients were excluded if they were taking >325 mg of aspirin a day, had a bleeding time. 8.0 min, or had congenital heart disease; patients with weight less than 45 kg, or if they had a preoperative haemoglobin level <12.5 g/dl. | | 71(10)  Vs  67.5(12.2)  Vs  70.5(10.2)  Vs  63(13.5) | CV disease | Cardiac surgery | | - IV TXA - Combined - Aprotinin - Placebo - POC tesing | Number of allogeneic blood transfusions in the OR and in the first 24 h in the ICU. | Volume of intraoperative and ICU blood loss over the first 24 h, and duration of time between the end of CPB and OR discharge. |
| Oertli 1994^1691^ | | - Switzerland - English - 1994 - Single-Centre - 160 - Women with breast cancer undergoing lumpectomy | | Patients with a history of thromboembolic events, severe varicose veins. Coagulation disorders or were receiving anticoagulant drugs. | | 58.1(10.4)  Vs  59.4(14.6) | Cancer | Plastic surgery | | - PO TXA - Placebo - - | - | - |
| Onodera 2012^1692^ | | - Japan - English - 2012 - Single-Centre - 100 - Patients scheduled to undergo TKA | | Patients showing DVT preoperatively were excluded, as were those with known coagulation disorders, abnormal coagulation test values, or receiving anti-coagulation medication. | | 70.4(10.1)  Vs  70.5(8.3) | - | Orthopaedic surgery | | - IV TXA - Placebo - - | - | blood loss and the risk of asymptomatic DVT development |
| Oremus 2014^1693^ | | - Croatia - English - 2014 - Single-Centre - 98 - Adult patients undergoing primary THA or TKA | | 1) known hypersensitivity to TXA, 2) history of coagulation abnormalities and thromboembolic disease or current abnormal coagulation test values, 3) history of stroke or acute coronary syndromes within 3 months before surgery, 4) renal failure (serum creatinine > 250 mmol/L [2.83 mg/dL]) or liver cirrhosis, and 5) chronic (ongoing) anticoagulant therapy | | 68.8(8.6)  Vs  68.6(8.3) | - | Orthopaedic surgery | | - IV TXA - Placebo - Cell salvage | Proportion of patients receiving postoperatively collected autologous drained blood reinfusion and total volume of blood drained within 24 postoperative hours. | Reinfused autologous blood volume, intraoperative blood loss, total external blood loss, and development of Hb and Hct over time (until fourth postoperative day). |
| Orpen 2006^1694^ | | - UK - English - 2006 - Single-Centre - 29 - Patients due to undergo primary unilateral total knee arthroplasty | | Patients with a history of thromboembolic disease, cerebrovascular disease, recent myocardial infarction or unstable angina, a coagulation defect, those with an allergy to TA and those who, not fit to undergo surgery under general anaesthetic. | | 73(5.9)  Vs  69(8.1) | Anaemia | Orthopaedic surgery | | - IV TXA - Placebo - - | - | On table blood losses, haemoglobin levels. |
| Oztas 2015^1695^ | | - Turkey - English - 2013 - Single-Centre - 60 - Patients with unilateral TKR | | Patients with inflammatory arthritis, history of  thromboembolism, myocardial infarction and stroke and  TXA allergy | | 68.5(5.3)  Vs  67(6.1) | - | Orthopaedic surgery | | - IV TXA - No TXA - - | - | Total blood loss and transfusion rate |
| Painter 2018^1696^ | | - Australia - English - 2016 - Multi-Centre - 140 - Patients undergoing lower limb arthroplasty | | Contraindications to the  administration of TA including active thromboembolic disease  or a history of venous (spontaneous or provoked) or arterial thromboembolic disease | | 69(9.1)  Vs  68(8.6) | - | Orthopaedic surgery | | - IV TXA - Placebo - Restrictive threshold | proportion of patients receiving allogenic blood transfusion and the feasibility of extending our trial methodology | change in Hb concentration and PCV, the incidence of adverse clinical events, incidence of surgical complications, length of hospital stay, and the change in a range of quality of life (EQ-5D), quality of recovery (QoR-15), osteoarthritis severity and joint specific questionnaires (Oxford Hip or Knee score). |
| Pauzenberger 2017^1697^ | | - Austria - English - 2015 - Single-Centre - 54 - Patients undergoing unilateral primary stemless anatomical or stemmed reverse total shoulder arthroplasty | | Patient refusal to participate in the study, revision surgery, indication for hemiarthroplasty, known allergy to TXA, anticoagulative medication, severe comorbidities, history of arterial or venous thromboembolic events, coagulopathy, haematological disorders, retinopathy, refusal to receive blood transfusion, pregnancy, or breastfeeding. | | 70.3(9.3)  Vs  71.3(7.9) | - | Orthopaedic surgery | | - IV TXA - Placebo - - | Post-operative drain blood loss | Need for post-operative transfusions, and early clinical outcome. |
| Pawar 2016^1698^ | | - India - English - 2016 - Single-Centre - 80 - All males with moderate and severe bladder outlet obstruction with international prostate symptom score of 13 or more and quality of life score of three or more | | Patients having neurogenic bladder, prostate carcinoma, previous prostatic surgery, and bladder stones | | 56.8(6)  Vs  57.2(5.4) | Cancer  Anaemia | Urology | | - IV TXA - No Treatment - - | - | Adverse Reaction  Risk & number of RBC transfusion  Haemoglobin (Hb), packed cell volume (PCV), and vitals recorded preoperatively, after 30 min of operation and 24 h of operation. |
| Penta de Peppo 1995^1699^ | | - Italy - English - 1995 - Single-Centre - 30 - Patients undergoing elective open-heart surgery | | Patients with a history  of gastrointestinal bleeding | | 60(12)  Vs  62(7)  Vs  64(10)  Vs  63(7) | CV disease | Cardiac Surgery | | - IV TXA - E-aminocaproic acid - Aprotinin - No Treatment - Cell salvage | - | The amount of blood drained intraoperatively by the Cell Saver system and postoperatively through the chest drains was recorded before reinfusion to the patient, as was the total blood loss both 1 hour and 24 hours after surgery. |
| Perez-Jimeno 2018^1700^ | | - Spain - English - 2018 - Single-Centre - 293 - Only cemented or non-cemented primary elective THA were included. | | Patients were excluded if presenting with hyper- or hypo-coagulability disorders, known allergy to TXA, intravenous iron, folic acid or recombinant human erythropoietin, epilepsy or hip fracture. | | 67(12)  Vs  67(12) | - | Orthopaedic surgery | | - IV TXA - No TXA - Iron therapy - Restrictive threshold | RBCT rate (percentage of transfused patients) and index (RBCT units per patient) | pre-RBCT haemoglobin, post-operative thromboembolic complications |
| Pertlicek 2015^1701^ | | - Czech Republic - Czech - 2015 - Single-Centre - 119 - Patients having primary unilateral total knee arthroplasty | | - | | 69.1(7.8)  Vs  68.7(7.5) | - | Orthopaedic surgery | | - IV TXA - No Treatment - - | - | The intra-operative blood loss, post-operative blood loss based on drainage, pre- and post-operative levels of haemoglobin and haematocrit, and the number of administered blood transfusions |
| Peters 2015^1702^ | | - USA - English - 2012 - Single-Centre - 32 - Patients undergoing posterior spinal fusion of at least 5 levels for correction of adult spinal deformity | | Patients were excluded if they had renal dysfunction identified by elevated blood urea nitrogen and creatinine (Cr) or blood urea nitrogen to Cr ratio greater than 20:1, had religious and/or other beliefs limiting blood transfusion, were using anticoagulant medications, had medical history leading to an abnormal coagulation profile preoperatively, or had significant medical history preventing the use of TXA or EACA described in the protocol or any history of coronary artery disease with stent placement. | | 60  Vs  43 | - | Spinal Surgery | | - IV TXA - Placebo - Cell salvage | Intraoperative blood loss and total blood transfusion rate. | Postoperative drain output, total blood loss (estimated blood loss [EBL] + wound drainage), and the change in haematocrit (Hct). |
| Pinosky 1997^1703^ | | - USA - English - 1997 - Single-Centre - 39 - first-time CABG patients | | patient age > 85 years, pregnancy, history of bleeding diathesis, gastrointestinal or upper urinary tract bleeding, or history of allergies to any previous antifibrinolytic therapy. | | 62.6(2.1)  Vs  62.1(2.7)  Vs  60.6(2.5) | CV disease | Cardiac surgery | | - IV TXA - EACA - No TXA - Cell salvage | - | The absolute amount of blood loss |
| Pleym 2003 | | - Norway - English - 2003 - Single-Centre - 79 - Patient undergoing CABG | | Patients receiving treatment with heparin or low-molecular-weight heparin, oral anticoagulants, nonsteroidal anti-inflammatory drugs, or other platelet inhibitors. | | 63.6(9.9)  Vs  62(9.2) | CV disease | Cardiac surgery | | - IV TXA - Placebo - Cell salvage | - | Transfusions. Preoperative haemoglobin and plasma creatinine levels. Haematocrit, platelet count, international normalized ratio, activated partial thromboplastin time, fibrinogen, and D-dimer values recorded before surgery and in the morning on the first postoperative day. |
| Pourfakhr 2016^1704^ | | - Iran - English - 2016 - Single-Centre - 186 - Patients who underwent prostatectomy surgery | | Patients using anticoagulant drugs such as aspirin and dipyridamole, with high PT (prothrombin time) and PTT (partial thromboplastin time) for any reason, with any history of thrombotic events, with a history of bleeding disorders, with chronic kidney disease (serum creatinine > 180 umol/L), with cardiovascular disease treated with drug eluting stent, with atrial fibrillation, with congenital or acquired thrombophilia, with known or suspected allergy to TRA, and undergoing general or epidural anaesthesia with the acknowledgment of the supervising physician. | | 67.7(9.9)  Vs  64.9(8.9) | Cancer | Urology | | - IV TXA - Placebo - - | - | The amount of bleeding and the rate of blood transfusion, the amount of blood inside the blood bags. |
| Prabhu 2015^1705^ | | - India - English - 2015 - Single-Centre - 36 - Patients underwent total knee arthroplasty | | 1. Patients aged less than 60 years  2. History of haemoglobinopathies /haemophilia/sickle cell disease or with minor or major coagulopathies were all excluded.  3. Those on medications on thyroid were excluded.  4. Those on immunomodulators and long term steroid intake. | | - | - | Orthopaedic Surgery | | - PO TXA - Placebo - - | - | The total amount of blood loss |
| Prakash 2017^1706^ | | - India - English - 2015 - Single-Centre - 100 - Patients undergoing primary total knee arthroplasty | | All patients with secondary osteoarthritis (rheumatoid and other inflammatory arthritis, post-traumatic arthritis), known allergies to tranexamic acid, major comorbidities, coagulopathies (International Normalised Ratio [INR] > 1.4), previous history of stroke or severe ischaemic cardiopathy and patients undergoing bilateral total knee arthroplasty. | | 70.2  Vs  68.3 | Anaemia | Orthopaedic Surgery | | - IV TXA - No TXA - - | - | Post-operative blood loss, Requirement of blood transfusion, Requirement of blood transfusion |
| Prasad 2018^1707^ | | - India - English - 2018 - Single-Centre - 60 - American Society of Anaesthesiologist's classification physical status 1 and 2 patients, both males and females, electively posted for open abdominal tumour surgery in the department of surgical oncology were included as study population. | | Patients with a history of bleeding diathesis, pulmonary embolism or deep vein thrombosis, those posted for hepatic resection or liver surgery, those posted for laparoscopic tumour removal, and those with a known allergy to tranexamic acid were excluded from the study. | | 45.9(8.8)  Vs  47.2(11.1)  Vs  49.3(8.6) | Cancer | General Surgery | | - IV TXA+Placebo - IV TXA + IV TXA - Placebo - - | Intraoperative blood loss | Total volume of intravenous fluids infused and whole blood units or blood products transfused were noted. Total duration of surgery in minutes (from skin incision to skin closure) was noted. |
| Pugh 1995^1708^ | | - London - English - 1995 - Single-Centre - 45 - Patients, age 18 years or over, who were scheduled for routine primary cardiac surgery. | | Not stated | | 58(10)  Vs  66(9.3) | CV disease | Cardiac surgery | | - IV TXA - Placebo - Cell salvage | - | The volume of blood loss and blood replacement were measured in the operative and postoperative periods. Haemoglobin concentration, platelet count, and white cell counts were determined preoperatively and at 24 hours postoperatively. |
| Raksakietisak 2015^1709^ | | - Thailand - English - 2013 - Single-Centre - 78 - Low-risk adult patients undergoing complex laminectomy | | Patients with history of thromboembolic diseases | | 52.6(2.8)  Vs  53.1(11.7) | - | Orthopaedic Surgery | | - IV TXA - Placebo - - | Perioperative blood loss occurring intraoperatively and 24 hours postoperatively. | Incidence of blood transfusions. |
| Rannikko 2004^1710^ | | - Finland - English - 2002 - Single-Centre - 136 - Men requiring TURP for obstructive urinary symptoms | | Patients taking finasteride or with a history of prostate cancer | | 71(6.6)  Vs  68(8.8) | - | Urology | | - PO TXA - Placebo - - | - | - |
| Raviraj 2012^1711^ | | - India - English - 2012 - Single-Centre - 175 - Patients undergoing simultaneous bilateral total knee arthroplasty | | Patients with bleeding or clotting disorders, those on preoperative anticoagulation  therapy, abnormal coagulation profile, rheumatoid arthritis, renal disorders or insufficiency, sickle cell disease, patients allergic to local anaesthetics/tranexamic acid. | | 65(9)  Vs  68(9) | - | Orthopaedic Surgery | | - IV TXA - Placebo - - | - | Haemoglobin levels were measured on postoperative day 1 and day 2, and the difference between the preoperative levels and lowest postoperative level was taken as the drop in haemoglobin level. The number of units of packed red blood cells received in each group was documented. |
| Reid 1997^1712^ | | - USA - English - 1997 - Single-Centre - 41 - Paediatric patients undergoing repeat cardiac surgery | | Children with pre-existing coagulopathy or preoperative anticoagulation | | 3.2(2.2)  Vs  3.1(1.8) | CV disease | Cardiac surgery | | - IV TXA - No TXA - - | - | Total blood loss and transfusion requirements |
| Roy 2012^1713^ | | - India - English - 2012 - Single-Centre - 50 - Patients undergoing primary unilateral total knee arthroplasty | | Patients with known allergy to tranexamic acid, severe anaemia (Hb %< 9 gm/dl), hepatic/cardio-respiratory/renal insufficiency, congenital or acquired coagulopathy and recent history of thromboembolic episode. Patients with severe deformity (> than 20 deg varus and flexion) and restricted range of motion (<90 deg) were also excluded | | 66(7.1)  Vs  66.5(8) | - | Orthopaedic Surgery | | - IV TXA - Placebo - - | - | Total blood loss and transfusion requirements |
| Sabry 2018^1714^ | | - Egypt - English - 2017 - Single-Centre - 70 - Patients who underwent decortication surgery for chronic thoracic empyema, encysted effusion, or clotted haemothorax on the elective way. | | Patients who required lung resection, reopening due to surgical bleeding, patients requiring anticoagulant postoperatively for fear of deep vein thrombosis, patients with renal failure, patients with liver cirrhosis, primary blood disease such as haemophilia or else, know allergy to tranexamic acid, and pregnant female patients. | | 41.9(14.6)  Vs  45.2(13.6) | - | Thoracic Surgery | | - Top TXA - Placebo - - | - | Total drainage and postoperative blood transfusion |
| Sadeghi 2007^1715^ | | - Iran - English - 2005 - Single-Centre - 67 - Patients with a diagnosis of fracture of the hip - necessitating hip surgery | | Patients with un-displaced subcapital fractures treated by pinning that have been shown to be fractures with low level loss of blood. Patients with preoperative haemoglobin less than 10 g/L., platelets count less than 100×10^9/l of blood, a known coagulopathies disorders, renal insufficiency (creatinine > 2 mg/dL), advanced hepatic dysfunction, and history of thromboembolic were also excluded. | | 51.8(25.7)  Vs  44.4(26.1) | Anaemia | Orthopaedic Surgery | | - PO TXA - Placebo - - | - | Blood loss during surgery, Transfusions |
| Sa-Ngasoongsong 2011^1716^ | | - Thailand - English - 2009 - Single-Centre - 48 - Patients with primary knee osteoarthritis i) no previous knee surgery; ii) no risk of abnormal bleeding tendency or bleeding disorder (normal coagulogram, serum creatinine <2.0 mg/dL, stop nonsteroidal anti-inflammatory drugs and antiplatelet drugs more than 7 days; and iii) no contra-indication for TXA use (no active intravascular clotting process, no acquired defective colour vision, no subarachnoid haemorrhage, no hypersensitivity to TXA, and no any of history of serious adverse effects, thrombotic disorder and haematuria) | | Patients with incomplete data collection, for example, malfunctioned drain or accidental drain removal. | | 69(8.2)  Vs  69.2(7.6) | - | Orthopaedic Surgery | | - IV TXA - Placebo - - | - | Basic postoperative data, such as drain volume, haematocrit (Hct), haemoglobin (Hb), amount of blood transfusion, and WOMAC score, were collected by well-trained research assistant. Complicated postoperative data  requiring clinical examination or physician  diagnosis, such as range of motion, and diagnosis  of complication, were collected by one of  the authors |
| Sa-Ngasoongsong 2013^1717^ | | - Thailand - UK - 2011 - Single-Centre - 135 - patients undergoing conventional TKR | | (1) no risk of abnormal  bleeding tendency or bleeding disorder (normal coagulogram, serum creatinine < 2.0 mg/dL, stop nonsteroidal anti-inflammatory drugs and antiplatelet drugs more than 7 days; and (2) no contra-indication for TXA use (no active intravascular clotting process, no acquired defective colour vision, no subarachnoid haemorrhage, no hypersensitivity to TXA, and no any of history of serious adverse effects, thrombotic disorder and haematuria). | | 68.1(6.2)  Vs  67.6(8.7)  Vs  66.2(7.3) | - | Orthopaedic Surgery | | - IV TXA (high dose) - IV TXA (low dose) - Placebo - - | - | Blood transfusion requirement was measured by recording the number of patients receiving transfusion and amount of blood transfusion in unit. Functional outcomes, such as KSK and WOMAC score, were evaluated at the clinic at 3-month, 6-month and 1-year period postoperatively. Postoperative complications such as wound hematoma, surgical site infection or systemic infection were evaluated at ward, at clinic as time of follow-up and/or by phone interview periodically. |
| Santos 2006^1718^ | | - Brazil - English - 2006 - Single-Centre - 60 - Patients undergoing CABG | | Patients undergoing cardiac surgery reoperation, renal insufficiency (plasma creatinine concentration higher than 2 mg/kg), and a history of haematological disorders, hepatic dysfunction or antiplatelet therapy within seven days of surgery. | | 62(9.2)  Vs  59(8.7) | CV disease | Cardiac surgery | | - IV TXA - Placebo - - | - | The mass of blood collected via mediastinal and pleural drains for a period beginning with chest closure and lasting 24 h represented blood loss. Other clinical outcomes were also analysed, such as reopening rates, myocardial infarction (new persistent Q-wave and creatine kinase myocardial-band levels more than 30 U/mL), acute renal insufficiency (plasma creatinine concentration higher than 2 mg/ kg), number of RBC transfusions, allergic reactions, convulsive seizures, mortality, and stroke (stroke as neurologic complication was defined by hemiparesis, hemiplegia, aphasia, or confusion and disorientation). |
| Sarzaeem 2014^1719^ | | - Iran - English - 2012 - Single-Centre - 200 - Patients with age over 18 years with planned TKA due to degenerative arthritis | | Patients with any cardiovascular problems (such as myocardial infarction, atrial fibrillation, angina), cerebrovascular conditions (such as previous stroke or previous vascular surgery) and thromboembolic disorders | | 66.9(7.2)  Vs  68.1(6.8)  Vs  67.5(7.6)  Vs  66.8(8.2) | - | Orthopaedic Surgery | | - IV TXA - IA TXA - Top TXA - No TXA - - | - | The amount of drainage was recorded in order to estimate the postoperative blood loss. Transfusion data. |
| Schiavone 2018^1720^ | | - Italy - English - 2015 - Single-Centre - 90 - Patients suffering from pertrochanteric fractures surgically treated with osteosynthesis with SupernailGT | | Polytrauma, patients operated more than 48 hours after the traumatic event; refusal of consent to participate in the study; dementia; patients whose relatives have not given their consent to participate; oral anticoagulant therapy; contraindications to treatment with tranexamic acid (a history of prior venous or arterial thrombosis, brain stroke, patients with creatinine clearance below 30 ml/min); patients who were administered tranexamic acid during or at the end of surgery; patients who require one or more transfusions before surgery; patients with INR> 1.2; patients with haematological diseases; patients who had the intra-operative complication of the migration of the intra-pelvic wire guide | | 84.3(8.3)  Vs  84.3(8.3) | CV disease  Renal disease  Anaemia | Orthopaedic Surgery | | - Top TXA - Placebo - - | proportion of  patients receiving at least 1 U of allogenic RBC  transfusion according to transfusion protocol. | - |
| Seddighi 2017^1721^ | | - Iran - English - 2011 - Single-Centre - 40 - Patients aged 20–70 years who were a candidate for major spinal surgeries, good medical condition, and accepted informed consent to attend the study. | | Patients aged < 20 and more than 70-year-old who had ischemic heart disease, diabetes, hepatic failure, traumatic vertebral fractures, severe renal failure, active intravascular clotting process, recent thromboembolic events, pregnancy, blurred color vision, coagulopathy, alcoholism and consumption of fluoxetine, contraceptives, insulin, and carbamazepine. | | 49.8(12.2)  Vs  43.7(10.2) | - | Orthopaedic Surgery | | - IV TXA - Placebo - - | - | The patient’s characteristics, type and duration of surgery, and the intra and postoperative blood loss were recorded |
| Seo 2013^1722^ | | - Korea - English - 2011 - Single-Centre - 150 - Patients aged between 55 and 80 years who planned to undergo TKA due to degenerative arthritis on a knee joint. | | Patients with any cardiovascular problems (such as myocardial infarction history, atrial fibrillation, angina), patients with cerebrovascular conditions (such as previous stroke or vascular surgery history), patients with thromboembolic disorders, or those exhibiting a deteriorating general condition. | | 66.8(6.3)  Vs  67.8(6.1) | - | Orthopaedic Surgery | | - IV TXA - Placebo - - |  | The amount of drainage was recorded in order to estimate the blood loss during TKA, and the difference in haemoglobin levels between the preoperative and the postoperative lowest one was also calculated. The frequency of transfusion, the number of blood units transfused, any perioperative complications or events such as infection, deep vein thrombosis (DVT), and pulmonary embolism were also recorded accordingly. |
| Seol 2016^1723^ | | - Korea - English - 2016 - Single-Centre - 100 - TKA patients | | Patients with secondary osteoarthritis (e.g., rheumatoid arthritis, posttraumatic osteoarthritis, gouty arthritis), a cardiovascular problem (e.g., myocardial infarction, atrial fibrillation, angina, heart failure), simultaneous bilateral TKA, a history of thromboembolic disease, bleeding disorder, known allergy to tranexamic acid, and lifelong warfarin therapy for thromboembolism prophylaxis | | 70.2(22.9)  Vs  68.3(8.1) | - | Orthopaedic Surgery | | - IV TXA - Placebo - - | - | The total volume of drained blood and the decrease in haemoglobin  at 6 hours, 24 hours, 48 hours and 5 days postoperatively were recorded. Blood transfusions were recorded as the number of units of packed erythrocytes. |
| Sethna 2005^1724^ | | - USA - English - 2005 - Single-Centre - 44 - Patients scheduled to undergo elective spinal fusion | | Patients with (1) pre-existing renal and hepatic disorders; (2) bleeding diathesis and abnormal prothrombin time, partial thromboplastin time (PTT), or platelet counts; and (3) intake of acetylsalicylate within 2 weeks or nonsteroidal anti-inflammatory drugs within 7 days before surgery. | | 13.6(1.8)  Vs  14(2) | - | Orthopaedic Surgery | | - IV TXA - Placebo - Cell salvage | - | Blood loss, transfusion requirements, coagulation parameters, and complications were assessed |
| Seviciu 2016^1725^ | | - USA - English - 2016 - Single-Centre - 121 - Patients over 18 years of age undergoing elective total primary knee arthroplasty, under spinal anaesthesia | | Patients with adverse reaction to TXA; congenital or acquired coagulation disorder; preoperative platelet count <100,000/mL or international normalized ratio >1.4; history of DVT, PE, or CVA; acquired defective colour vision; renal insufficiency (glomerular filtration rate <20 mL/min); severe liver disease; coronary stents; or pregnant patients | | 65.7(8.6)  Vs  61.1(10.5)  Vs  64.8(8)  Vs  62.9(8.4) | - | Orthopaedic Surgery | | - IV TXA - IV TXA+BSS - BSS only - Placebo - - | The change in Hb at day 3 | Change in haematocrit and estimated blood loss. |
| Shakeri 2018^1726^ | | - Iran - English - 2018 - Single-Centre - 50 - Patients who had either lumbar spinal stenosis or lumbar spondylolisthesis and were candidates for 2 or more than 2 levels of laminectomy and posterolateral fusion performed with instruments (pedicle screw and rods). | | Patients with a history of treatment with anticoagulant drugs, dipyridamole and oral contraceptives, those with abnormal international normalized ratio, prothrombin time and partial thromboplastin time, patients with cerebrovascular accident, myocardial infarction, coagulopathies, traumatic brain injury, cardiopulmonary resuscitation, renal failure, smoking, opioids, diabetes mellitus, hypertension, coronary artery disease, pregnant and breastfeeding women, and those who received packed cell transfusion during or after operation | | 50.5(6.5)  Vs  49.1(9.1) | - | Orthopaedic Surgery | | - IV TXA - Placebo - - | - | The two groups were compared with respect to age, sex, weight, body mass index (BMI), bleeding in the operation room, total volume of bleeding, bleeding volume in the first 12 hours after surgery, volume of bleeding between 12–24 hours after surgery, packed cells received, and hos­pitalization time. |
| Shen 2015^1727^ | | - China - English - 2013 - Single-Centre - 81 - 1) Primary knee osteoarthritis and (2) unilateral TKA. | | (1) inflammatory or autoimmune diseases; (2) blood coagulation disorders; (3) history of thromboembolic disease; (4) severe anaemia; (5) peripheral neuropathy; (6) malignant tumour; (7) TXA or low molecular heparin contraindication; (8) pre-operative anticoagulant drug use; and (9) those who did not cooperate in the experiment. | | 65.7(8.2)  Vs  64.9(7.9) | - | Orthopaedic Surgery | | - IV TXA - Placebo - - | - | The following data were obtained: (1) height, and weight, and body mass index; (2) intraoperative blood loss, i.e., the liquid of the drainage bottle minus the intraoperative flushing flu­id plus the net increase in gauze; (3) post-operative drainage amount at 12 h and total drainage amount; (4) Hgb, Hct, PLT, D-dimer, total blood loss, and hidden blood loss which was cal­culated according to Sehat-design mathematical methods [9], pre-operative and post-operative levels of Hgb, Hct, and PLT at 1, 3, and 5 days, and pre-operative and post-operative 24-h D-dimer values; and (5) DVT. |
| Shi 2013a^1728^ | | - China - English - 2013 - Multi-Centre - 552 - Patients eligible for randomization were 1173 men and women aged 18 to 85 years undergoing primary and isolated on-pump CABG | | Previous cardiac surgery, haematocrit level less than 33%, platelet count less than 100 000 x 10^3/uL, allergy to tranexamic acid, and being recruited in other studies. | | 60(9.4)  Vs  59.6(9) | CV disease | Cardiac surgery | | - IV TXA - Placebo - - | blood loss, major bleeding, and red blood cell  (RBC) transfusion volume and exposure. | Major morbidity and mortality. Major morbidity was defined as permanent disability caused by stroke, postoperative myocardial infarction, renal failure, and respiratory failure. |
| Shi 2013b^1729^ | | - China - English - 2013 - Single-Centre - 117 - Patients receiving on-pump coronary artery bypass grafting without Clopidogrel and Aspirin cessation | | Previous cardiac surgery, haematocrit less than 33%, platelet count less than 100,000/mL, or allergy to tranexamic acid, and those recruited in other studies. | | 60.3(8.4)  Vs  59.5(9.9) | CV disease | Cardiac surgery | | - IV TXA - Placebo - - | Volume of allogeneic erythrocyte transfused perioperatively. | - |
| Shi 2017^1730^ | | - China - English - 2016 - Single-Centre - 100 - (1) Patients with lumbar spinal stenosis or lumbar spondylolisthesis who were scheduled to undergo posterior lumbar decompression interbody fusion; the conservative therapy had failed. (2) Patients aged 18 to 80 years. (3) Patients who provided written informed consent. | | (1) Allergy to TA. (2) History of bleeding disorders or thromboembolic events. (3) Severe cardiac or respiratory disease and renal or hepatic dysfunction. (4) Platelet count <150,000/mm^3^. (5) Preoperative Hb <10 g/dL. (6) Uncontrolled hypertension; high blood pressure (BP >160/90 mm Hg). (7) ASA physical status >III. (8) Intake of nonsteroidal anti-inflammatory drugs within 7 days before surgery. (9) Pregnancy. | | 53.7(12)  Vs  55.8(13.1) | - | Spinal Surgery | | - IV TXA - Placebo - - | Intraoperative estimated blood loss and total blood loss. | Packed red blood cells received and postoperative haemoglobin and haematocrit levels. |
| Shimizu 2011^1731^ | | - Japan - English - 2007 - Single-Centre - 160 - Children younger than 18 years of age who were scheduled to undergo elective cardiac surgery with CPB | | Neonates of less than 1 month of age, children on mechanical ventilation preoperatively, and children on inotropic support before surgery were excluded from the study. Other exclusion criteria included a pre-existing coagulation disorder, re-operation within 48 h, obvious kidney or liver disease, and known allergy to TXA | | 2.6(0.8)  Vs  2.6(0.9) | CV disease | Cardiac surgery | | - IV TXA - Placebo - - | 24-h blood loss. | Re-exploration of the chest for bleeding, transfusions of blood products requirement, Mechanical ventilation in the ICU, length of stay, and complications. |
| Shinde 2015^1732^ | | - India - English - 2015 - Single-Centre - 56 - Patients of Indian origin undergoing TKA for primary osteoarthritis of the knee joint | | Allergy to TEA, rheumatoid arthritis, revision total knee arthroplasty, coagulopathy (preoperative platelet count ≤150000/mm^3^, BT, PT, CT abnormality), previous history of thromboembolic disease (cerebrovascular accident, deep vein thrombosis, myocardial infarction), severe ischemic heart disease, NYHA class 3 and 4, serum creatinine >1.5 mg/dL, severe pulmonary disease, e.g. FEV1 ≤50% normal, hepatic failure and preoperative anaemia (Hb <10 g/dL). | | 64.6(7.6)  Vs  61.2(8.2) | - | Orthopaedic Surgery | | - IV TXA - Placebo - - | - | Blood loss, blood transfusion requirements. |
| Shore-Lesserson 1996^1733^ | | - USA - English - 1996 - Single-Centre - 30 - Adult patients undergoing repeat open heart surgery | | Patients were excluded if they had preoperative coagulopathy that included thrombocytopenia  (Platelet count <100,000/mm^3), uremic thrombocytopathy (patients receiving preoperative dialysis), and inherited or acquired coagulopathy (von Willebrand disease, haemophilia A, residual Warfarin effect, etc.). Also excluded were patients receiving inotropic therapy or intra-aortic balloon counter-pulsation, and patients who refused blood transfusion for religious reasons. | | 68(13)  Vs  63(6) | CV disease  Anaemia | Cardiac surgery | | - IV TXA - Placebo - POC testing - Cell salvage | - | Routine coagulation tests, D-dimer levels, mediastinal tube drainage, and transfusion requirements were compared |
| Song 2017^1734^ | | - Korea - English - 2015 - Single-Centre - 200 - Patients undergoing primary navigated TKA | | patients with secondary osteoarthritis (rheumatoid and other inflammatory arthritis, posttraumatic arthritis), known allergies to TXA, major comorbidities (American Society of Anaesthesiology (ASA) grade 4 and above), coagulopathies (INR >1.4), history of previous deep vein thrombosis (DVT) or patients on antithrombotic treatment, previous history of stroke or severe ischemic cardiopathy, and patients undergoing bilateral total knee arthroplasty | | 69.2(6.4)  Vs  69.8(6.8)  Vs  70.8(6.8)  Vs  69.8(6.8) | - | Orthopaedic Surgery | | - IV TXA - Top TXA - Combined - Placebo - - | - | Evident loss through drain, total loss based on Gross method and haemoglobin balance method, hidden losses, haemoglobin and haematocrit drop, functional scores, and all possible complications related to TXA. |
| Speekenbrink 1995^1735^ | | - Netherlands - English - 1995 - Single-Centre - 60 - Patients undergoing CABG (with a preoperative platelet count of less than 246 x 10(9)/L) | | Patients with a body weight of more than 100 kg. Patients with already impaired renal function (creatinine level more than 200 µmol/L) were not included. Also patients with intravenous heparin treatment or a history of coagulopathy were excluded. | | 61(11)  Vs  60(9)  Vs  62(10)  Vs  57(12) | CV disease | Cardiac surgery | | - IV TXA - Dipyridamole - Aprotinin - Placebo - - | - | Intraoperative haemoglobin loss. The volume of mediastinally shed blood was measured 6 and 24 hours after the operation. Intraoperative and postoperative transfusions of homologous blood products were recorded. |
| Spitler 2019^1736^ | | - USA - English - 2019 - Single-Centre - 93 - Patients with fractures of the pelvic ring, acetabulum, and proximal femur. | | - | | 47.2  Vs  42.8 | - | Orthopaedic Surgery | | - IV TXA - No TXA - Cell Salvage | Transfusion rates and total blood loss (TBL) |  |
| Springer 2016^1737^ | | - USA - English - 2016 - Single-Centre - 186 - 1. Patients presenting for primary unilateral hip or knee arthroplasty 2. N18 y of age 3. Preoperative haemoglobin on day of surgery ≥ 10 mg/dL | | 1. Patients with a preoperative Hgb b 10 mg/dL 2. Patients who are unwilling to consent to blood transfusions 3. Patients with a history of bleeding disorder 4. Patients on anticoagulation therapy preoperatively (ASA 325 mg, Plavix or Coumadin) 5. Patients with a history of thromboembolic events (DVT, PE, CVA MI) 6.Patients with platelet counts b 100,000 7. Patients with kidney disease (serum Cr N 1.2) 8. Patients with end-stage renal disease or on haemodialysis 9. Patients with renal transplant 10. Patients presenting for bilateral total hip or knee arthroplasty 11. Patients presenting for conversion or revision total hip or knee procedures 12. Patients donating pre-autologous blood 13. Patients with primary hematologic disease or malignancy 14. Patients with allergy to TA 15. Patients with hepatic disease 16. Patients not discontinuing steroids use before surgery 17. Patients with religious beliefs/practices prohibiting blood transfusions 18. Patients with cognitive impairment 19. Patients who are terminally ill. | | 63.3(12.1)  (Overall mean) | - | Orthopaedic Surgery | | - IV TXA - Reinfusion drains - No TXA - Iron therapy | Allogeneic blood transfusion, measured as a dichotomous variable; the change in haemoglobin level (delta haemoglobin); autologous blood reinfusion; and hospital costs. | - |
| Stowers 2017^1738^ | | - New Zealand - English - 2017 - Multi-Centre - 134 - Patients older than 18 years undergoing primary unilateral TKA | | History or risk of thrombosis, active thromboembolic disease, refused blood products, known hypersensitivity to TXA or any of its ingredients, complex hematologic disorders requiring manipulation, pregnant and lactating women, taking anticoagulant therapy within 5 days of surgery (warfarin, dabigatran, heparin, rivaroxaban), or had severe renal failure (estimated glomerular filtration rate <29) | | 70(7.6)  Vs  70(8.5)  Vs  71(8.6) | - | Orthopaedic Surgery | | - IV TXA - IA TXA - Placebo - - | estimated blood loss (EBL)  as calculated from the difference from preoperative haemoglobin  (Hb) and final Hb before discharge or day 3 at the latest. | Functional measurements using patient self-reported questionnaires (Short-Form 12 survey and Oxford knee scores) were performed preoperatively and at 6 weeks after surgery. Transfusion rates, median length of stay, and 30-day readmissions and complications were also measured. Important complications captured included symptomatic deep vein thrombosis (DVT), pulmonary embolism (PE), and infection. ROM, both passive and active, was measured as a surrogate for postoperative swelling. |
| Sudprasert 2018 ^1739^ | | - Thailand - English - 2016 - Single-Centre - 57 - Men and women, 18 to 70 years of age with injuries involving the thoracic or lumbar spine (Thoracolumbar Injury Classification and Severity score ≥5) undergoing long-segment instrumented posterior spinal fusion with local autologous bone graft No neurological deficits American Society of Anesthesiologists physical status class I, II, or III | | Renal insufficiency History of thromboembolic events (e.g., pulmonary embolism, embolic stroke, and deep venous thrombosis) History of significant cardiovascular diseases (e.g., unstable angina, recent myocardial infarction, significant arrhythmia, and uncontrolled hypertension) History of acquired defective colour vision Coagulation disorder Gross haematuria or microhematuria Displaced laminar fracture on computed tomography axial section that might be associated with dural tears Allergy to tranexamic acid Take aspirin or nonsteroidal anti-inflammatory drugs within a week before randomization and during the hospitalization | | 52(16.2)  Vs  51.5(18.1) | - | Orthopaedic Surgery | | - Top TXA - Placebo | Requirement for PRC transfusion postoperatively prior to discharge home. | Total drainage volume, time to drain removal, and duration of postoperative hospitalization. |
| Sun 2017^1740^ | | - China - English - 2017 - Single-Centre - 180 - Patients who were scheduled to undergo primary unilateral TKA | | Allergy to TA, anaemia, severe cardiopulmonary disease, and refusal of blood products and those complicated with haematological or thromboembolism disease | | 67.2(8.1)  Vs  67.3(7.2)  Vs  68.1(7.9)  Vs  67.4(8.4) | - | Orthopaedic Surgery | | - IV TXA (High dose) - IV TXA (Medium dose) - IV TXA (Low dose) - No TXA - - | Postoperative blood transfusion | The blood loss including  intraoperative blood loss (fluid volume in intraoperative drainage  bottle _ rinse solution volume) and postoperative blood loss (the drainage volume for 48 hours postoperatively) |
| Taghaddomi 2009a^1741^ | | - Iran - English - 2009 - Single-Centre - 80 - Patients undergoing lumbar hernia disc resection | | History of bleeding disorder, chronic renal insufficiency (serum creatinine>2 mg/dL), perioperative anaemia (Hb<10 gr/dL), and warfarin medication | | 40.4(7.6)  Vs  40.5(12.4)  Vs  42(17.3)  Vs  42.6(10.4) | - | Orthopaedic Surgery | | - Total intravenous +TXA - Total intravenous - TXA - Inhalation Anaesthetic +TXA - Inhalation Anaesthetic - TXA - - | - | The patients characteristics and intraoperative variables including the amount of blood loss, duration of the surgery, hemodynamic changes, the time of awareness, duration of recovery period were collected |
| Taghaddomi 2009b^1742^ | | - Iran - English - 2009 - Single-Centre - 100 - Patients undergoing off-pump coronary artery bypass surgery | | Patients with a history of bleeding disorders, active chronic hepatitis or cirrhosis, chronic renal insufficiency (serum creatinine >2 mg/dL), preoperative anaemia (Hb < 11 g/dL), previous cardiac surgery, and myocardial infarction >7 days before surgery. Also, patients receiving potent antiplatelet agents like adenosine diphosphate  inhibitors (Ticlopidine and Clopidogrel) but not aspirin were excluded | | 54.7(10.9)  Vs  60.3(10.2) | CV disease | Cardiac surgery | | - IV TXA - No TXA - - | - | Hematologic parameters, volume of blood loss, blood transfusion, and other clinical data were recorded throughout the perioperative period. |
| Taksaudom 2017^1743^ | | - Thailand - English - 2015 - Single-Centre - 80 - Patients who underwent elective on-pump cardiac surgery | | Re-sternotomy procedure, emergency or urgent cases, bleeding diathesis (haemophilia or platelet count<10010^9/L, preoperative coagulopathy), renal failure (creatinine level>2.0 mg/dL), history of TA allergy, discontinuation of antiplatelet medication less than 7 days before surgery, heparin infusion within 24 h before surgery, aortic surgery, and complex adult congenital heart disease. | | 57(13)  Vs  56.3(14.3) | CV disease | Cardiac surgery | | - Top TXA - Placebo - - | 24-h blood loss | The volume of blood products transfused, re-exploration rate, length of hospital stay, mortality, morbidity, and TA-related complications. |
| Tanaka 2001^1744^ | | - Japan - English - 2001 - Single-Centre - 99 - Patients who were undergoing total knee arthroplasty | | Known allergy to TNA, preoperative hepatic or renal dysfunction, serious cardiac or respiratory disease, congenital or acquired coagulopathy, and a history of thromboembolic disease. | | 65(7.4)  Vs  65(8.1)  Vs  65(8.1)  Vs  65(8.8) | Anaemia | Orthopaedic Surgery | | - IV TXA - Pre-op TXA - Post-op TXA - No TXA - - | - | The need for blood transfusion and apparent blood loss. Thromboembolic and other complications were noted during the hospital stay. |
| Tang 2018^1745^ | | - China - English - 2015 - Single-Centre - 587 - Patients were diagnosed with elbow stiffness by Kay classification; patients diagnosed with heterotopic ossification of bone; (3) patients without skin sensibility aging from 45 to 81 years old; (4) patients without surgical contraindication | | Patients with muscle atrophy, nerve damage or poor postoperative recovery; patients with severe primary diseases, mental disease, severe skin diseases or other complications affects elbow joint; (3) patients with a joint instability; (4) clinical trial subjects who didn't respond well to treatment or had other reasons | | 65.1(3.5)  Vs  65.3(4.1) | - | Orthopaedic Surgery | | - IV TXA - No TXA - - | - | Postoperative haemorrhage and complications |
| Tavares Sanchez 2018^1746^ | | - Spain - Spanish - 2015 - Single-Centre - 119 - Patients undergoing cementless total hip arthroplasty | | Patients who were allergic to tranexamic acid (Amchafibrin) or any of its components, who had experienced adverse reactions previously after administration of the drug and when the reason for surgery was an acute fracture (admitted via the emergency department) were excluded from the study. | | - | - | Orthopaedic Surgery | | - Top TXA - Placebo - - | - | Bleeding, transfusion requirements and length of stay, and describe the complications |
| Tengberg 2016^1747^ | | - Denmark - English - 2016 - Single-Centre - 72 - Patients undergoing surgery for extra-capsular hip fractures | | Allergy to tranexamic acid, ongoing thromboembolic event (deep venous thrombosis (DVT), pulmonary embolism (PE), arterial thrombosis or cerebral thrombosis), reduced kidney function (defined as a serum creatinine > 120 umol/L), anticoagulation therapy including vitamin K-antagonists, direct thrombin inhibitors, direct factor X-a inhibitors and platelet aggregation inhibitors (not including acetylsalicylic acid), disseminated intravascular coagulation (DIC), bleeding in the upper urinary tract (risk of obstruction), patients with a history of cramps, subarachnoid bleeding, malignancy, pathological fracture, previous operation on the affected hip, more than one current fracture, or bodyweight in excess of 100 kg. | | 79.8(11.2)  Vs  75(12.6) | Anaemia | Orthopaedic Surgery | | - IV TXA - IV TXA - Placebo - - | Total blood loss (TBL) | Number of transfusions, risk reduction for receiving at least one transfusion and surgical blood loss during the operative procedure. |
| Thipparampall 2017^1748^ | | - India - English - 2017 - Single-Centre - 59 - Patients undergoing hip surgeries | | Patients with a history of severe ischaemic heart disease, pulmonary embolism, deep vein thrombosis (DVT), hepatic or renal failure or allergy to TA were excluded from the study. | | 48(14)  Vs  45(13)  Vs  55(11) | Anaemia | Orthopaedic Surgery | | - IV TXA (bolus) - IV TXA (bolus + infusion) - Placebo - - | Intraoperative blood loss | Need for transfusions. Hb and haematocrit values were recorded at 6 h after surgery, on the morning of post-operative day 1 and 2. Patients were monitored clinically for evidence of DVT twice daily. |
| Tian 2018^1749^ | | - China - English - 2017 - Single-Centre - 100 - patients of intertrochanteric fractures, underwent with proximal femoral nail anti-rotation | | (1) pathological fracture; (2) allergy to TXA; (3) Serious cardiac or respiratory disease; (4) congenital or acquired coagulopathy; (5) history of thromboembolic disease such as cerebral infarction, pulmonary embolism, myocardial infarction, or deep vein thrombosis; (6) recent thrombophilia; (7) preoperative hepatic or renal dysfunction (male creatinine level >115 mmol/L, female creatinine level >100 mmol/L); and (8) diabetic. | | 77.7(6.5)  Vs  79.2(6.5) | - | Orthopaedic Surgery | | - IV TXA - No TXA - - | - | Volume of intraoperative blood loss and postoperative drainage, and the need for postoperative blood transfusion and transfusion volume for all patients. |
| Triyudanto 2016^1750^ | | - Indonesia - English - 2016 - Single-Centre - 22 - Patients having TKR | | Patients who consumed anticoagulant and anti-thrombocyte aggregation, had preoperative Hb ≤10.5 g/dl for man and woman, had intraoperative blood loss ≥500 cc, with mental illness, had uncontrolled diabetes mellitus (DM), rheumatoid arthritis, malignancy, and immunosuppression, had infected knee, had abnormal prothrombin time (PT) and activated partial thromboplastin test (APTT) | | 65.1(6.8)  (Overall mean) | - | Orthopaedic Surgery | | - IV TXA - IA TXA - Placebo - - | Postoperative bleeding | Number of RBC transfusion  Perioperative blood loss |
| Tsutsumimoto 2011^1751^ | | - Japan - English - 2011 - Single-Centre - 40 - Patients undergoing total hip and knee arthroplasty. | | Patients with chronic renal failure, cirrhosis of the liver, serious cardiac disease, allergy to TXA, a history of thromboembolic disease, bleeding disorders, hyper-coagulation status, disseminated intravascular coagulation, and those who were receiving antiplatelet and/or anticoagulant drugs. | | 68(11)  Vs  65.8(11.8) | Anaemia | Orthopaedic Surgery | | - IV TXA - Placebo - - | - | Intra- and postoperative blood loss |
| Tzatzairis 2016^1752^ | | - Greece - English - 2015 - Single-Centre - 120 - Patients with a diagnosis of primary osteoarthritis undergoing unilateral TKR without tourniquet | | Allergy and/or hypersensitivity to TXA; subarachnoid haemorrhage; a known history of thromboembolic disease, cardiovascular disease (a history of myocardial angina or infarction); coronary or vascular stent placed within the past 12 months; preoperative renal or hepatic dysfunction; cerebral vascular disease (a history of stroke); preoperative coagulopathy (a platelet [PLT] count <150,000/mm3 or an international normalized ratio greater than 1.4; retinal vein or artery occlusion | | 69.5(6.6)  Vs  69.1(8.6)  Vs  68.5(7.5) | - | Orthopaedic Surgery | | - IV TXA - Top TXA - No TXA - - | calculated blood loss, the transfusion rate, and quantity of allogeneic blood units | Complications such as  DVT, pulmonary embolism, superficial and deep infections, and any  deterioration of hepatic or renal function. |
| Ugurlu 2017^1753^ | | - Turkey - English - 2015 - Single-Centre - 123 - Patients undergoing primary unilateral total knee arthroplasty | | Flexion deformity of > 30 degrees, varus/valgus > 30 degrees, preoperative  use of anticoagulants (acetylsalicylic acid, enoxaparin, warfarin, or any other oral or IV agent), abnormalities in coagulation screening tests, history of DVT or pulmonary embolism, transient ischemic attack, stroke, renal (serum  creatinine > 2 standard deviation [SD] for age) or hepatic insufficiency, and pregnancy | | 69.4(7.5)  Vs  70.6(8.6)  Vs  66.4(6.6) | - | Orthopaedic Surgery | | - IV TXA - Top TXA - No TXA - Restrictive threshold | - | The haemoglobin values were recorded preoperatively and postoperatively on the same day and on day 1 and day 2. Removal of the drain postoperatively and length of hospital stay, as well as any complications such as pulmonary embolism or deep venous thrombosis, were also noted. |
| Uozaki 2001^1754^ | | - Japan - English - 2001 - Single-Centre - 14 - Patients undergoing elective cardiopulmonary bypass for coronary artery bypass surgery. | | Not stated | | 72.3(4.1)  Vs  63.3(5.3) | CV disease | Cardiac surgery | | - IV TXA - Placebo - - | - | Intraoperative and postoperative blood loss |
| Vanek 2005^1755^ | | - Czech Republic - English - 2004 - Single-Centre - 91 - Patients undergoing OPCAB | | Not stated | | 68.4(5.6)  Vs  67.3(4.5)  Vs  68.9(4.5) | CV disease | Cardiac surgery | | - IV TXA - Aprotinin - Placebo - - | 30-day mortality | ICU LOS  Hospital LOS  Risk of RBC transfusion  Perioperative blood loss  Reoperation for bleeding |
| Vara 2017^1756^ | | - USA - English - 2017 - Single-Centre - 102 - Patients undergoing primary reverse total shoulder arthroplasty | | Minors, acute proximal humeral fracture, concomitant procedures (e.g., latissimus dorsi tendon transfer), known allergy to TXA, preoperative anaemia (Hb <11 g/dL in women, Hb <12 g/dL in men), refusal of blood products, coagulopathy (thrombophilia, platelet count <150,000 mm3, international normalized ratio >1.4, partial thromboplastin time >1.4 times normal), history of thromboembolic event, major comorbidities (severe pulmonary disease, coronary artery disease, previous myocardial infarction, renal failure), or refusal to give written consent. | | 67(9)  Vs  66(9) | - | Orthopaedic Surgery | | - IV TXA - Placebo - - | - | Calculated total blood loss, drain output, and haemoglobin (Hb) drop were measured. Postoperative transfusions were recorded.  Complications were assessed out to 6 weeks postoperatively. |
| Veien 2002^1757^ | | - Denmark - English - 2002 - Single-Centre - 30 - Patients scheduled for TKR in spinal anaesthesia with the use of a tourniquet, | | Patients with age less than 18 years, recent myocardial infarction (<6months), unstable angina, severe aortic or mitral valve stenosis, previous stroke, unmedicated hypertension, history of thromboembolic episodes, bleeding disorders or warfarin medication. | | 70.5(9.5)  Vs  69.5(9) | - | Orthopaedic Surgery | | - IV TXA - Placebo - Cell salvage | - | Blood loss |
| Verma 2014^1758^ | | - USA - English - 2014 - Single-Centre - 125 - Patients with adolescent idiopathic scoliosis | | - | | 15.3(2.3)  Vs  14.6(1.8)  Vs  15(2.3) | - | Spinal Surgery | | - IV TXA - EACA - Placebo - Cell salvage | Intraoperative blood loss and postoperative drainage. | Transfusion requirements and haematocrit changes both intraoperatively and postoperatively. |
| Vijay 2013^1759^ | | - India - English - 2013 - Single-Centre - 90 - Patients undergoing hip fracture surgery | | Patients with chronic disease like Rheumatoid arthritis, ischemic heart disease, malignancy, history of any previous thromboembolic episodes, haemoglobin <8 g/dl were excluded from the study. | | 48.8(16.2)  Vs  49.3(19.5) | Anaemia | Orthopaedic surgery | | - IV TXA - Placebo - Cell salvage | - | Postoperative bleeding (volume of blood in the drain), percentage fall of haemoglobin, transfusions and complications were recorded |
| Virani 2016^1760^ | | - India - English - 2015 - Single-Centre - 137 - Patients above 65 years of age, underwent peritrochanteric fracture surgery | | Patients with low preoperative platelet counts, bleeding disorders and coagulopathies, patients with severe hepato-renal dysfunction and cardiopulmonary disease, and those on aspirin or NSAIDS in the week preceding surgery | | 67  Vs  69.1 | Anaemia | Orthopaedic surgery | | - IV TXA - No TXA - - | - | The postoperative drain output was recorded, as well as the haemoglobin level and the patients needing blood transfusion. |
| Volquind 2016^1761^ | | - Brazil - English - 2013 - Single-Centre - 62 - Patients undergoing primary total knee replacement | | Patient’s refusal to participate in the study, allergies to drugs used, changes related to coagulation, use of nonsteroidal anti-inflammatory or antiplatelet drugs seven days before surgery, kidney or liver failure, pregnancy, and previous history of deep venous thrombosis or pulmonary embolism | | 67.8(5)  Vs  63.9(4) | - | Orthopaedic surgery | | - IV TXA - Placebo - - | - | Haemoglobin, haematocrit, and blood loss were recorded 24 h after surgery. Deep vein thrombosis was investigated during patient’s hospitalization and 15 and 30 days after surgery in review visits. |
| Wang 2012^1762^ | | - China - English - 2012 - Single-Centre - 231 - Patients scheduled for elective OPCAB | | Known allergy to the study drug, history of bleeding disorders, preoperative anaemia (haemoglobin  [Hb] <10 g/dL), chronic renal insufficiency (serum creatinine >2 mg/dL), active chronic hepatitis or cirrhosis, previous cardiac surgery, myocardial infarction < 30 days, and withdrawal of clopidogrel or aspirin <5 days before surgery. | | 60.5(8)  Vs  60(8.5) | CV disease | Cardiac surgery | | - IV TXA - No TXA - POC testing | - | Postoperative bleeding and transfusion requirements |
| Wang 2013^1763^ | | - China - English - 2013 - Single-Centre - 60 - Patients with degenerative lumbar instability with stenosis | | Patients with chronic renal failure, cirrhosis of the liver, serious cardiac disease, allergy to TXA, thromboembolic disease, bleeding disorders, hyper coagulation status, disseminated intravascular coagulation, and those who were receiving antiplatelet and/or anticoagulant drugs at the time of the study | | 63.1(4)  Vs  62(4.6) | - | Orthopaedic surgery | | - IV TXA - Placebo - Restrictive threshold | - | Intraoperative and postoperative  blood loss |
| Wang 2015a^1764^ | | - China - English - 2015 - Single-Centre - 60 - patients treated with unilateral primary cement TKA | | Patients with a body mass index (BMI) < 35 kg/m2, rheumatoid arthritis, simultaneous bilateral TKA, allergy to TXA, preoperative anaemia (haemoglobin [Hb] value of <11 g/dL in females and <12 g/dL in males), refusal of allogeneic blood products, or a history of coagulopathy or a thromboembolic event | | 64.9(6.3)  Vs  64.9(6.7) | - | Orthopaedic surgery | | - Top TXA - Placebo - - | Total blood loss, transfusion rate, and the number of blood units transfused. | Coagulation-fibrinolysis markers, including prothrombin time (PT), activated partial thromboplastin time (APTT), thrombin time (TT), platelet numbers (PLT), fibrinogen (FIB) and D-dimer levels recorded on PODs 1, 3, and 5. The wound healing condition (skin necrosis, hematoma, infection) was monitored the patients discharged. |
| Wang 2015b^1765^ | | - China - English - 2014 - Single-Centre - 100 - Patients underwent primary unilateral TKA | | Patients with preoperative anaemia or coagulopathy; patients with infectious active diseases like lower limb infection or systemic infection disease; patients with TXA contraindications; patients with a history of venous thromboembolic disease or thromboembolic disorders; patients with clotting problem like liver tumour or cirrhosis; patients intended to participate in autologous blood transfusion; incompatibility patients. | | 52.6(12.4)  Vs  53.2(10.2) | - | Orthopaedic surgery | | - Top TXA - Placebo - - | - | Postoperative haemoglobin, blood coagulation index, total blood loss volume, drainage volume, blood transfusion rate and lower extremity deep vein thrombosis (DVT) rate |
| Wang 2015c^1766^ | | - China - Chinese - 2015 - Single-Centre - 69 - Patients who received bilateral total knee arthroplasty | | - | | 62(4.6)  Vs  64(7) | - | Orthopaedic surgery | | - IV TXA - Placebo - - | - | Total blood loss, intraoperative blood loss, the hidden blood loss, amount of postoperative drainage, the ratio of blood transfusion, hemoglobin, D-dimer, prothrombin time and activated partial thromboplastin time |
| Wang 2016^1767^ | | - China - English - 2014 - Single-Centre - 124 - Patients scheduled for THA | | History of any of the following: haemophilia, deep vein thrombosis, pulmonary embolism, stents, ischemic heart disease, anticoagulant medication, serious liver or renal dysfunction, or allergy to tranexamic acid. | | 59.3(11.2)  Vs  63.8(12.5) | - | Orthopaedic surgery | | - IV TXA - Placebo - - | proportions of patients in each group  (a) requiring blood transfusion, (b) experiencing deep vein thrombosis  (DVT) or (c) experiencing pulmonary embolism (PE). | Total blood loss, drained blood loss, decrease in haemoglobin and haematocrit as well as other complications. |
| Wang 2017a^1768^ | | - Taiwan - English - 2015 - Single-Centre - 198 - Primary unilateral minimally invasive TKA | | Patients who had a coagulopathy, severe renal impairment (creatinine clearance, <30 mL/min), concomitant use of protease inhibitors of human immunodeficiency virus, or fibrinolytic agents that contraindicated the use of Rivaroxaban, prior surgery on the affected knee, a history of thromboembolic disease requiring life-long anticoagulant therapy or antiplatelet drugs that could not be stopped before operation, previous allergic history to TXA, or contrast medium for radiographic examination or a preoperative Hb level less than 10 g/dL | | 68.1(6.6)  Vs  69.6(7.7) | - | Orthopaedic surgery | | - IV TXA - Placebo - - | - | Total blood loss was calculated from the maximum haemoglobin drop after surgery plus amount of transfusion. The transfusion rate and wound complications were recorded in all patients. |
| Wang 2017b^1769^ | | - Taiwan - English - 2017 - Single-Centre - 150 - Patients aged 30 years and older, who were scheduled for a primary unilateral TKA for end-stage osteoarthritis | | 1. Patients with preoperative Hb <110 g/L. 2. Patients with thromboembolic history or preoperative situation like DVT or PE, or arterial stenosis with or without concomitant coronary artery bypass grafting. 3. Patients with preoperative D-dimer >3 times normal level. 4. Patients with cardiovascular history, such as myocardial infraction, angina, or atrial fibrillation. 5. Patients with cerebrovascular history of previous stroke. 6. Patients with clotting disorders including prolonged prothrombin time or activated partial thromboplastin time, or abnormal international normalized ratio. 7. Patients with allergic history of TXA. 8. Pregnant or lactating women, drug abusers or alcoholics. 9. Patient with severe complications, such as severe liver and kidney diseases, New York Heart Association class III or above, heart failure, or patients with severe infection. 10. Patients combined the use of other medicine that may have an impact on the outcome of the study. 11. Patients diagnosed as inflammatory arthritis including rheumatoid arthritis, pigmented villonodular synovitis, and so on. | | 67.4(8.2)  Vs  67.6(7.4) | - | Orthopaedic surgery | | - IV TXA - Placebo - - | - | The amount of total and hidden blood loss (HBL), drainage, transfusion, changes in haemoglobin levels, and complications were recorded. |
| Wang 2019^1770^ | | - China - English - 2018 - Single-Centre - 300 - all patients (age > 18 years) with hip osteoarthritis or osteonecrosis of the femoral head, scheduled for elective, unilateral, primary THA, were consecutively screened | | known allergy to TXA; a haemoglobin (Hb) level of < 11 g/dL; a history of arrhythmia, pulmonary embolism (PE), deep venous thrombosis (DVT) or severe ischaemic heart disease; an acquired or congenital coagulopathy; previous vascular or cardiac bypass surgery; a history of high-risk medical co-morbidities (severe renal insufficiency, hepatic failure or severe pulmonary disease); current full dose anticoagulant therapy (warfarin or heparin) within 1 week; refusal of blood products or participation; or participation in another clinical trial during the last year. | | 68.7(9.8)  Vs  67.3(8.3)  Vs  68.9(7.7)  Vs  67.2(9.6)  Vs  68.5(9.5) | - | Orthopaedic Surgery | | - Placebo - PO TXA (3g+3g Placebo) - PO TXA (4g + 2g Placebo) - PO TXA (5g+1g Placebo) - PO TXA (6g) - Restrictive threshold | Total blood loss on POD 3. | Hb drops on POD 1 and 3, total blood loss on POD 1, intra-operative blood loss, allogeneic red cell transfusion rates, the number of blood units transfused, the length of hospital stay, the post-operative changes in joint function (i.e. the range of motion [ROM] and the severity of hip pain at rest and with movement based on visual analogue scale [0, no pain, and 100, worst pain imaginable] on POD 1, 2 and 3) and Harris Hip Score (HHS) at discharge. |
| Watts 2017^1771^ | | - USA - English - 2017 - Single-Centre - 138 - Patients who presented with a low-energy, isolated, FNF (AO 31B) treated with either hemi- or total hip arthroplasty within 72 hours of injury | | Blood transfusion before surgery; creatinine clearance (CrCl) <30 mL/min; previous unprovoked and/or recurrent deep venous thrombosis (DVT) or pulmonary embolism (PE); recent myocardial infarction (MI), cerebrovascular event, or provoked DVT or PE within 30 days; coronary stent placement within 6 months; history of heritable hypercoagulable condition; disseminated intravascular coagulation; subarachnoid haemorrhage; pregnancy; and active breastfeeding. | | 81(10)  Vs  82.2(10) | - | Orthopaedic surgery | | - IV TXA - Placebo - Restrictive threshold | Proportion of patients who underwent blood transfusion during hospitalization. | Calculated blood loss, number of units transfused during hospitalization, and incidence of adverse events at 30 and 90 days including thromboembolic event, wound complications, reoperation, hospital readmission, and all-cause mortality. |
| Wei 2006^1772^ | | - China - English - 2006 - Single-Centre - 76 - Patients undergoing elective OPCAB | | Patients with valve diseases, myocardial infarction less than four weeks before surgery, left ventricular ejection fraction lower than 40%, neurologic or pulmonary disorders, renal and liver failure were not eligible. | | 62.8(7.9)  Vs  60.7(8) | Anaemia | Cardiac surgery | | - IV TXA - Placebo - - | - | Haematochemical parameters including platelet adhesion rate, D-dimer and fibrinopeptide-A (FPA) were analysis. Volume of blood loss, blood transfusion and other clinical data were recorded throughout the perioperative period. |
| Wei 2014^1773^ | | - China - English - 2014 - Single-Centre - 201 - 1. Age 45–80 years 2. Preoperative haemoglobin values N11 g/dl 3. Normal international normalized ratio (INR), prothrombin time (PT), partial thromboplastin time (PTT) values 4. Consented to undergo unilateral cementless THA 5. Had no history of previous hip surgery | | 1. Had a documented history of thrombo-embolism 2. Had an allergy to TXA 3. Had a high risk of venous thrombosis for intravenous use of TXA according to the American Academy of Orthopaedic Surgeons Guideline | | 63.6(7)  Vs  63.9(6.7) | - | Orthopaedic surgery | | - IV+Top TXA - Placebo - - | the nadir in-patient Hct, maximum  Hct drop from preoperative levels, length of hospital stay, transfusion  rates, wound complications and total blood loss (TBL) | - |
| Wong 2008^1774^ | | - Canada - English - 2008 - Single-Centre - 147 - Patients having spinal fusion surgery | | Patients with a history of allergy to TXA, acquired disturbances of  colour vision, spine tumour, intra-dural pathology, ankylosing spondylitis, preoperative anaemia, i.e., haemoglobin <11 g/dL in females; haemoglobin <12 g/dL in males, refusal of blood products i.e., Jehovah’s witnesses, coagulopathy, preoperative anticoagulant therapy, fibrinolytic disorders requiring intraoperative antifibrinolytic treatment, preoperative platelet count <150,000/mm3, International Normalized Ratio (INR) >1.4, prolonged partial thromboplastin time (PTT) (>1.4 x normal), a history of thromboembolic disease, pregnancy, significant co-morbidities i.e., severe ischemic heart disease New York Heart Association Class III–IV, previous myocardial infarct (MI), severe pulmonary disease, i.e., forced expiratory volume in 1 min <50% normal, chronic renal failure, hepatic failure. If intraoperative surgical complications such as uncontrollable surgical bleeding from broken vertebral laminae, or dural tears, etc. occurred, the patients were excluded from the study. | | 56.8(16.2)  Vs  50(16.2) | - | Orthopaedic surgery | | - IV TXA - Placebo - Cell salvage | The total perioperative estimated and calculated blood loss intraoperatively and 24 h postoperatively. | Incidence of allogeneic blood exposure, and duration of hospital stay. |
| Wu 2006^1775^ | | - Taiwan - English - 2004 - Single-Centre - 214 - Patients undergoing liver resections for various liver tumours | | Patients who underwent  emergency surgery for a ruptured liver tumour or patients whose liver tumours were resected under cardiopulmonary bypass | | 57(14)  Vs  62(16.5) | - | Hepatobiliary Surgery | | - IV TXA - Placebo - Restrictive threshold | - | The patients’ background, blood transfusion rates, and early postoperative results in the 2 groups were compared. |
| Xie 2015^1776^ | | - China - English - 2012 - Single-Centre - 90 - Age 18 to 65 years, the presence of a unilateral closed calcaneal fracture, type II or type III, according to Sanders classification (14), and the absence of chronic disease (e.g., hypertension, hypercholesterolemia, and diabetes mellitus) or the presence of well controlled chronic illness | | Patients with bilateral calcaneal fractures or other injuries, a known coagulopathy disorder, renal insufficiency, hepatic dysfunction, serious cardiac disease, an allergy to TXA, or receiving antiplatelet and/or anticoagulant drugs at the time of the study | | 43.4(8.8)  Vs  42.6(9.8) | - | Orthopaedic Surgery | | - IV TXA - Placebo - Restrictive threshold | Blood loss | Wound complications |
| Xu 2012^1777^ | | - China - English - 2012 - Single-Centre - 80 - Patients undergoing scheduled idiopathic scoliosis surgery | | Pre-existing cardiac, pulmonary, renal and hepatic disorders; intake of NSAIDs within 7 days before surgery; history of coagulation disorders, Deep vein thrombosis (DVT) or pulmonary embolisms; lower preoperative Hb (\100 g/l); abnormal clotting tests, such as prothrombin time (PT) and platelet counts. | | 20.4(3.1)  Vs  18.8(4.3)  Vs  19.1(3.2)  Vs  20.6(3.7) | - | Orthopaedic Surgery | | - Placebo - Batroxobin - IV TXA - IV TXA+Batroxibin - Placebo - - | - | The amounts of blood loss, transfusion  requirements, frozen fresh plasma (FFP) and overall drainage were assessed. The haemoglobin concentration  (Hb), haematocrit and platelet counts were recorded preoperative  y, postoperatively and on the first operative day.  The coagulation parameters were measured meanwhile.  Deep vein thrombosis (DVT) was diagnosed by ultrasound. |
| Xu 2015^1778^ | | - China - English - 2014 - Single-Centre - 224 - Patients were adults who received primary unilateral THA regardless of the type or size of prosthesis implanted; the intervention was topical (intra-articular) administration of TXA; the full text of each article was available; (iv) outcome measures included total blood loss, transfusion rate, and incidence of thromboembolic complications | | Patients who had allergy to tranexamic acid; thrombotic disorder; patients who were on anticoagulant treatment. | | 67.5(10.7)  Vs  67.4(8.8) | - | Orthopaedic Surgery | | - Top TXA - Placebo - Restrictive threshold | The rate of deep vein thrombosis (DVT) and pulmonary embolism (PE), transfusion rate, difference between the preoperative haemoglobin and the lowest postoperative haemoglobin during the hospital stay. | Total volume of drainage, intraoperative blood loss, total blood loss and other perioperative complications. |
| Xu 2017^1779^ | | - China - English - 2016 - Single-Centre - 80 - Patients with spinal degenerative diseases | | (1) patients with comorbid severe medical diseases such as Osteoporosis, anaemia, renal failure, and cardiovascular diseases; (2) patients with abnormal coagulation function; (3) patients who have taken anti-platelet aggregates such as aspirin or anticoagulants in the last month; and (4) patients who had a history of thromboembolisms. | | 53.1(12)  Vs  57.4(10.7) | - | Orthopaedic Surgery | | - Top TXA - No TXA - - | - | Intraoperative blood loss, drainage, transfusion requirements |
| Yanartas 2015^1780^ | | - Turkey - English - 2015 - Single-Centre - 132 - Patients undergoing CABG , 18 to 75 years of age, body mass index between 25 and 31, with normal ejection fraction (≥50%), initial haematocrit value within the boundaries of the normal for adult male and female patients (31 to 40% for women and 34 to 45% for men). | | Re-do cardiac surgery, emergent surgery, preoperative coagulation disorder, preoperative use of Clopidogrel, Coumarin anticoagulants, heparin, or acetylsalicylic acid within the previous 5 days before operation, preoperative congestive heart failure, ejection fraction <49%, preoperative renal dysfunction (serum creatinine > 1.3 mg/dL), chronic oliguria/anuria requiring dialysis, preoperative hepatic dysfunction (serum aspartate/alanine amino transferase > 40 U/L), preoperative electrolyte imbalance, history of pancreatitis or current  Corticosteroid treatment. | | 60.1(9.2)  Vs  62.5(9.6)  Vs  61.8(7.9)  Vs  63.2(11.4) | CV disease | Cardiac Surgery | | - IV TXA (RS) - RS only - IV TXA (HES) - HES only - - | Values of haemoglobin, haematocrit, platelet, prothrombin time, activated prothrombin time, international normalized ratio (INR), blood urea nitrogen (BUN), creatinine, sodium, potas­sium, chloride, lactate, pH, base excess | The effect of priming solution on clinical out­comes such as; 1-Aortic cross-clamp time, 2-Cardiopulmonary bypass time, 3-The use of inotropic support, 4-Intra-aortic balloon pump, 5-Prolonged mechanical ventilation, 6-Deve-lopment of pneumonia, 7-Perioperative myo­cardial infarction, 8- Cerebrovascular event (stroke, transient ischemic attack), seizure, 9-Atrial fibrillation and other rhythm disturbanc­es, 10-Need for renal replacement therapy (RRT), 11-Reoperation secondary to bleeding, 12-Intensive care unit stay, 13-Hospital stay and, 14-Thirty-day mortality |
| Yang 2015^1781^ | | - Greece - English - 2013 - Single-Centre - 80 - Patients underwent Primary TKA | | Patients with haemorrhagic blood diseases; haemoglobin  (Hb)<90 g/L; with peripheral nerve vascular disease, cancer, history of thromboembolic disease; affected lower limb with a history of infection; and ASA rating>3. | | 69(5)  Vs  67(6) | - | Orthopaedic Surgery | | - IA TXA - Placebo - - | - | Routine blood examination, blood loss and blood transfusion after TKA |
| Yassen 1993^1782^ | | - UK - English - 1993 - Single-Centre - 20 - Patients undergoing orthoptic liver transplantation | | No stated | | 44.8(12.2)  Vs  49.6(14.2) | - | Hepatobiliary Surgery | | - IV TXA - No TXA - Cell salvage | - | Transfusion and blood loss |
| Yen 2017^1783^ | | - Taiwan - English - 2016 - Single-Centre - 98 - Patients who underwent primary minimally invasive TKA | | Patients with a documented history of thromboembolic disease, cardiovascular disease (myocardial infarction or angina), stroke, coagulopathy, lifelong warfarin treatment for thromboembolic prophylaxis, impaired hepatic or renal function (impaired hepatic function was defined as liver enzyme level, AST or ALT, which is more than twice normal range, history of liver cirrhosis, elevated total bilirubin level, or coagulopathy (INR < 1.3); and impaired renal function was defined as GFR<55ml/min/1.73 m^2, which is relative contraindicated for chemical venous thromboembolism and venography), and patients with an allergy history to tranexamic acid or concomitant use of protease inhibitors of human immunodeficiency virus, or fibrinolytic agent that contraindicated the use of Rivaroxaban and preoperative anaemia (a haemoglobin level of ≤10 g/dl). | | 69.1(7.9)  Vs  69.6(5.5)  Vs  70.8(6) | - | Orthopaedic Surgery | | - IV TXA - Top TXA - Placebo - - | Estimated total blood loss. Haemoglobin (Hb) and haematocrit (Hct) levels were measured on PODs 1, 2, and 4. | The rate of perioperative blood transfusion, the rate of deep-vein thrombosis (DVT), wound complications, visual analogue scale (VAS) on POD 1, the length of hospital stay, and the range of motion of the knee. |
| Yi 2016^1784^ | | - China - English - 2014 - Single-Centre - 150 - Patients undergoing total hip arthroplasty | | Patients with an allergy to TXA; had been treated with warfarin, heparin, or oestrogen before surgery; had a history of hyper-coagulation, haemophilia, deep vein thrombosis, or pulmonary embolism; were morbidly obese; or had hepatic or renal dysfunction. | | 53.6(14.7)  Vs  54(12.5)  Vs  56.6(12.2) | - | Orthopaedic Surgery | | - IV TXA+Top TXA - IV TXA + Placebo - Placebo - - | Blood-loss variables (total, intraoperative, and drainage blood loss; changes in haemoglobin, haematocrit, and platelet concentration; and amount of IV transfusion fluid) and transfusion values (frequency of transfusion and number of transfused blood units). | The length of the hospital stay, range of hip motion, Harris hip score, and prevalence of deep vein thrombosis and pulmonary embolism. |
| Yuan 2017^1785^ | | - China - English - 2017 - Single-Centre - 560 - Patients who underwent TKA, osteoarthritis or rheumatoid arthritis, primary unilateral TKA, at least a 3-week follow-up, normal clotting mechanism, and effectively controlled medical diseases. | | Previous bilateral TKA, revision TKA, severe hepatic and/or renal diseases, coagulopathy, or a bleeding disorder. | | 63.7(8)  Vs  63.2(6.9)  Vs  63.1(6.8)  Vs  63.2(6.9) | - | Orthopaedic Surgery | | - IV TXA - Top TXA - PO TXA - Placebo - - | Postoperative 48-hour Hb loss and drainage volume, number of transfusions, transfusion and TXA costs, and thromboembolic complications. | Postoperative inpatient time and wound healing 3 weeks after TKA. |
| Yue 2014^1786^ | | - China - English - 2013 - Single-Centre - 101 - Patients undergoing primary unilateral total hip arthroplasty for OA or ONFH | | Patients who were receiving anticoagulant therapy, patients with a history of haemophilia, deep venous thrombosis, pulmonary embolism or ischemic heart disease and patients who were allergic to tranexamic acid | | 60.9(13.2)  Vs  63.7(10) | - | Orthopaedic Surgery | | - Top TXA - Placebo - - | The transfusion rate, the DVT and PE events. | Total blood loss, drain blood loss, haemoglobin and haematocrit drop, postoperative hospitalization days and other complications. |
| Zabeeda 2002^1787^ | | - Israel - English - 2002 - Single-Centre - 50 - Patients scheduled for elective or urgent CABG. | | Patients with an ejection fraction less than 40%, impaired kidney function (creatinine > 2 mg/dL), a history of abnormal bleeding, or an abnormal coagulation profile. Patients receiving bilateral mammary artery grafts were excluded from the study. | | 65.6(9)  Vs  65(13) | CV disease | Cardiac surgery | | - IV TXA - Placebo - Restrictive threshold | - | Blood loss, transfusion, reoperation, fibrinogen level, fibrinogen split products, platelet size, and platelet function. |
| Zekcer 2017^1788^ | | - Brazil - English - 2014 - Single-Centre - 90 - Patients with unilateral total knee arthroplasty (TKA) as a result of Ahlbäch grade III, IV and V arthrosis | | History or identified risk of deep venous thrombosis or pulmonary embolism or history of coagulation or cardiovascular disorders; vascular diseases | | - | - | Orthopaedic Surgery | | - IV TXA - Top TXA - No TXA - - | volume of blood loss | Need for transfusion (patient received two units of packed red blood cells every time haemoglobin levels were below 8.0 g/dL). |
| Zeng 2017^1789^ | | - China - English - 2014 - Single-Centre - 100 - All adult patients (aged between 18 and 90 years) undergoing primary unilateral THA | | Allergy to TXA, preoperative hepatic or renal dysfunction, preoperative use of anticoagulant medication 7 days prior to surgery, history of fibrinolytic disorder, cerebrovascular accident, myocardial infarction, New York heart association class III or IV heart failure, atrial fibrillation, history of deep vein thrombosis or pulmonary embolus, preoperative international normalized ratio (INR) >1.4, activated partial thromboplastin time (aPTT) >1.4× normal, platelets <140 000/mm3, and failure to give consent. | | 51.1(14.9)  Vs  56.1(11.2) | - | Orthopaedic Surgery | | - IV TXA - Placebo - - | total blood loss (calculated  using Gross’s equation), haemoglobin, haematocrit and platelet concentration changes on the third postoperative day, the amount of drainage, the amount of intraoperative blood loss, the frequency of transfusion, and the number of blood units transfused. | the length of postoperative stay, range of hip motion  (measured by goniometer), Harris hip scores (HHS), and any perioperative complications or events such as infection,  DVT or PE. |
| Zhang 2007^1790^ | | - Chinese - Chinese - 2007 - Single-Centre - 102 - Patients underwent total knee arthroplasty | | - | | 68.1(9)  Vs  67.6(8.3) | - | Orthopaedic Surgery | | - IV TXA - Placebo - - | - | The amounts of blood loss and blood transfusion during operation and after operation. |
| Zhang 2015^1791^ | | - China - Chinese - 2015 - Single-Centre - 65 - Patients undergoing primary total hip arthroplasty | | - | | 62.3(16)  Vs  65.8(10.6) | - | Orthopaedic Surgery | | - IV TXA - Placebo - - | - | Intraoperative blood loss, postoperative dominant blood loss and hidden blood loss, pain score, blood transfusion rate, deep vein thrombosis and day of hospitalization |
| Zhang 2016^1792^ | | - China - English - 2014 - Single-Centre - 50 - Patients with osteonecrosis of the femoral head who underwent unilateral THA | | Patients with diabetes,  bleeding disorders, preoperative anaemia (haemoglobin Hb<120g/l),malignancies,  history of venous thrombosis disease, arteriosclerosis, varicose veins and other cardiovascular diseases, allergy to TXA, liver and kidney dysfunction, participation in other clinical trials and intraoperative adverse events which were believed could lead to intraoperative and postoperative bleeding. | | 44.5(2.4)  vs  43.4(3.8) | - | Orthopaedic Surgery | | - IV TXA - No TXA - Restrictive threshold | - | Adverse events, intraoperative blood loss, postoperative drainage, total loss of red blood cells. |
| Zhao 2018^1793^ | | - China - English - 2017 - Single-Centre - 120 - Patients undergoing primary THA | | Patients with a body weight index (BMI) > 30 kg/m2; Crowe type 3 or 4 dysplasia; previous hardware; prior hip surgery; and an inability to tolerate general anaesthesia. Patients meeting the above inclusions are being operated via the direct anterior approach for THA. In addition, patients were excluded if they had bilateral arthroplasty, allergy to TXA, or history of renal failure, kidney transplant, a recent arterial thromboembolic event such as myocardial infarction or stroke, hyper-coagulation, haemophilia, deep vein thrombosis, or pulmonary embolism. Patients were also excluded if they declined to participate or to receive blood products. | | 59.5(11.4)  Vs  60.4(10.3)  Vs  59.8(10.6) | - | Orthopaedic Surgery | | - IV TXA - PO TXA - Placebo - - | Haemoglobin drop, haematocrit levels, total blood loss, intra-operative blood loss, need for transfusion, and volume transfused. | Thromboembolic events, wound complications, the length of post-operative hospital stay, and 30-day readmission. |
| Zhou 2018^1794^ | | - China - English - 2018 - Single-Centre - 170 - All adult patients scheduled to undergo primary unilateral THA in our hospital and consented | | e allergy to TXA; coagulopathy (preoperative platelet count < 150,000/ mm3; international normalized ratio (INR) > 1.4; or any indicator of prolonged partial thromboplastin, prothrombin, and thrombin time of >1.4 times the normal.); history of thromboembolic disease, including deep vein thrombosis (DVT), pulmonary embolism (PE), myocardial infarction (MI), and cerebral infarction (CI); taking anticoagulant drugs within a week before surgery; major comorbidities, including severe ischemic heart disease (New York Heart Association Class III or IV), renal dysfunction (glomerular filtration rate < 60), or hepatic dysfunction (glutamic–pyruvic transaminase > 80 or glutamic oxaloacetic transaminase > 80); retinopathy; pregnancy; participated in another clinical trial within a year; and those who completely stay in bed for more than 3 weeks. | | 63.2(10)  Vs  65.8(9.4)  Vs  65.3(11.2) | - | Orthopaedic Surgery | | - IV TXA - Top TXA - Placebo - - | total blood loss | Allogeneic blood transfusion requirement, drain blood loss, decreased haemoglobin level. |
| Zohar 2004^1795^ | | - Israel - English - 2004 - Single-Centre - 40 - Patients undergoing elective total knee replacement | | Patients with a history of severe ischemic heart disease (New York Heart Association Class III and IV), chronic renal failure, cirrhosis, bleeding disorders, or current  anticoagulant therapy | | 73(8)  Vs  73(7) | - | Orthopaedic Surgery | | - IV TXA - Placebo - - | - | - |
| Zonis 1996^1796^ | | - Canada - English - 1996 - Single-Centre - 82 - Children undergoing cardiac operations in which cardiopulmonary bypass | | Patients with a history of haematuria, renal failure, previous thrombotic episodes, or past bleeding complications. | | 5.2(4.8)  Vs  4.3(4.2) | CV disease | Cardiac surgery | | - IV TXA - No TXA - - | - | Post-operative blood loss and fluid replacement were recorded for the next 24 hours. In addition, haemoglobin, platelet counts, and coagulation measures were recorded every 6 hours. |
| Zufferey 2010^1797^ | | - France - English - 2010 - Single-Centre - 110 - Patients requiring surgery for an isolated hip fracture of less than 48 h | | Pregnancy or breast-feeding, contraindication for tranexamic acid (previous arterial or venous thrombosis, creatinine clearance < 30 ml/min, previous seizure or Oestroprogestative therapy), multiple fractures, contraindication for prophylaxis with Fondaparinux (Arixtra, GlaxoSmithKline, Brentford, UK), and requirement for anticoagulant therapy that could not be stopped. | | 81(12)  Vs  82(9.7) | CV disease  Anaemia | Orthopaedic Surgery | | - IV TXA - Placebo - - | Incidence of patients requiring the transfusion of at least 1 U of allogeneic RBC from surgery up to day 8. | postoperative bacterial infection, which was defined as the composite of pneumonia, other lower respiratory tract infection, blood stream infection, urinary tract infection, superficial wound infection, deep wound infection, and osteomyelitis or septic arthritis up to 6 weeks. |
| - ***POC*** | | | | | | | | | | | | | |
| Ak 2009^1798^ | | - Turkey - English - 2009 - Single-Centre - 224 - Adult patients undergoing elective first time CABG with cardiopulmonary bypass | | Preoperative haemodynamic instability, malignancies, history of bleeding diathesis, use of low molecular weight heparin until the day of operation, recent treatment (<5days) with a glycoprotein IIb/IIIa antagonist or Clopidogrel, impaired renal function (creatinine>2mg/dL) and liver disease resulting in elevated liver function tests | | 63.2(19.2)  Vs  65.9(22.1) | | CV disease | | Cardiac Surgery | - TEG - Standard of care - Tranexamic Acid | incidence of blood transfusion, blood loss | amount of blood and blood products consumed perioperatively, blood loss  mediastinal chest tube drainage, need for additional protamine, need of tranexamic acid  infusion, mortality, risk of surgical cause of reoperation for bleeding and clinical complications  outcome after CABG (superficial soft tissue infection, major respiratory complications,  postoperative renal dysfunction) and haematological variables (haematocrit  and platelets) |
| Avidan 2004^1799^ | | - United Kingdom - English - 2004 - Single-Centre - 102 - Routine elective first-time CABG surgery with cardiopulmonary bypass, managed according to standard clinical practice at local institution treated by the same surgical, intensivist and anaesthetic team | | Patients with preoperative abnormal clotting tests, including INR> 1.5, aPTT ratio > 1.5, platelet count < 150 X 109 litre−1, any medication affecting  coagulation within 72 hours of surgery, including warfarin, heparin, low molecular  weight heparin, aspirin and Clopidogrel | | 65.9(22.1)  Vs  62(9.6) | | CV disease | | Cardiac Surgery | - TEG+Hepcon+PFA - Standard of care - Tranexamic acid - Restrictive Threshold | Blood loss and transfusion, postoperative 24-hour blood loss- | INR, aPTT, TEG variables, haemoglobin and platelet values, coagulation  values |
| Cui 2010^1800^ | | - China - English - 2009 - Single-Centre - 31 - Cyanotic paediatric patients diagnosed with transposition of the great arteries or double-outlet right ventricle; the operation that the patients underwent was arterial switch operation or double roots transplantation. Haematocrit higher than 54% before operation | | History of blood disease; anticoagulation treatment before surgery; medication that affects haemostasis (such as prostaglandin E1); difficult sternal closure caused by anatomical or surgical reasons | | 2.5(2.1)  Vs  3.7(1.7) | | CV disease | | Cardiac Surgery | - TEG + fibrinogen - Standard of care - Cell Salvage | - | chest closure time (c-T); FFP volume used at closure time (c-FFP); PLT units used at closure time (c-PLT); FFP volume used in the first 24 h in ICU (ICU-FFP); PLTs used in ICU (ICU-PLT); red blood cells (RBCs) used in ICU during the first 24 h (ICU-RBC); total FFP (FFP volume used in operation and in ICU during the first 24 h); total RBC (RBC units used in operation and ICU during the first 24 h);total PLT (PLT units used in closure time and ICU during the first 24 h); chest drainage at 1, 6, and 24 h; mechanical ventilator time; ICU stay; and hospitalization time |
| Girdauskas 2010^1801^ | | - Germany - English - 2010 - Single-Centre - 56 - adult patients (> 18 years) undergoing high risk aortic surgery including urgent and emergency surgery (25 with acute type A dissection) with hypothermic circulatory arrest | | Pregnant, known (inherited) coagulation disorders (haemophilia A or B, activated protein C resistance, etc), inability to give informed consent | | 63.5(13.5)  Vs  59.9(17.5) | | CV disease | | Cardiac Surgery | - ROTEM - Control - Tranexamic acid - Restrictive Threshold - Cell Salvage | cumulative transfusion of allogeneic blood units (PRBCs, FFP, and  platelets) | use of prothrombin complex concentrate, fibrinogen concentrate,  and recombinant factor VIIa (NovoSeven), blood losses in the first 12 and 24 postoperative  hours, risk of surgical re-exploration for bleeding, time to extubation, neurologic  and renal complications, length of stay in ICU |
| Karkouti 2016^1802^ | | - Canada - English - 2015 - Multi-Centre - 7402 - patients undergoing cardiac surgery with cardiopulmonary bypass | | None stated | | 67(4.4)  Vs  67(3.7) | | CV disease | | Cardiac Surgery | - ROTEM + PLT MAPPING - Control - - | red cell transfusion from surgery to postoperative day seven- | Transfusion of other blood products, major bleeding, and major complications. |
| Kultufan Turan 2006^1803^ | | - Turkey - Turkish - 2010 - Single-Centre - 40 - Cardiac surgery either CABG or valve surgery | | None stated | | 51.4  Vs  55.1 | | CV disease | | Cardiac Surgery | - TEG - Control - - | incidence of blood transfusion (whole blood, RBCs, FFP, and platelets) | - |
| Nuttal 2001^1804^ | | - USA - English - 2001 - Single-Centre - 92 - Adult men and not pregnant adult women with abnormal microvascular bleeding after CPB, all types of elective open cardiac surgery requiring CPB | | Patients were not excluded if they received preoperative aspirin or  antiplatelet therapy | | 69(42.2)  Vs  68(48) | | CV disease | | Cardiac Surgery | - TEG+SLT - Control - Tranexamic acid | need for allogenic blood products during the entire stay in hospital | platelet count, TEG variables, PT, aPTT, mediastinal drainage in the ICU,  risk of reoperation due to bleeding |
| Royston 2001^1805^ | | - United Kingdom - English - 2010 - Single-Centre - 60 - Adult patients (> 21 years), high risk of requiring haemostatic products, cardiac surgery (heart transplantation, revascularization, bypass, Ross procedure, multiple valve or valve and revascularization surgery) | | If reoperation due to bleeding was performed or early death of the patient, the data were excluded and replaced by measurements from an additional patient allocated to the same group | | - | | CV disease | | Cardiac Surgery | - TEG - Control - - | reduced total exposure to haemostatic component therapies | mortality, TEG variables, PT, aPTT, platelet count, fibrinogen concentration,  mediastinal tube drainage at 6 and 12 hours |
| Shore-Lesserson 1999^1806^ | | - USA - English - 1999 - Single-Centre - 105 - Adult cardiac surgical patients at moderate to high risk of microvascular bleeding and thus had a moderate to high risk for requiring a transfusion. Included patients underwent single valve replacement, multiple valve replacement, combined coronary artery bypass plus valvular procedure, cardiac reoperation, or thoracic aortic replacement. Patients receiving preoperative heparin infusion and those who had taken aspirin within the past 7 days were included | | Significant pre-existing hepatic disease (transaminase levels > 2 times control) or renal disease requiring dialysis, or if they required preoperative inotropic support | | 64.5(15)  Vs  67.1(14.5) | | CV disease | | Cardiac Surgery | - TEG - Control - - | reduction in transfusion requirements | Coagulation tests, TEG variables, postoperative blood loss into mediastinal  drainage at 6-hour intervals for 2 days postoperatively, platelet count, PT, aPTT, fibrinogen level, TEG variables |
| Wang 2010^1807^ | | - Taiwan - English - 2010 - Single-Centre - 28 - Adult patients undergoing orthotopic liver transplantation | | None stated | | 58.6(4.8)  Vs  51.3(12.5) | | Anaemia | | HPB Surgery | - TEG - Control - Restrictive threshold | - | 3 years mortality, transfusion requirements, total amount of IV  fluids (fluid total, hydroxyethyl starch, albumin), blood loss, urine output |
| Weber 2012^1808^ | | - Germany - English - 2010 - Single-Centre - 100 - Patients were suitable for this trial after two inclusion steps Step 1: Patients (>= 18 years) scheduled for elective, complex cardiothoracic surgery (combined CABG and valve surgery, double or triple valve procedures, aortic surgery or redo surgery) with CPB were re-operatively screened for eligibility, and written consent was obtained Step 2: Patients were enrolled in the study after heparin reversal following CPB if at least one of the two inclusion criteria were fulfilled: (1) diffuse bleeding from capillary beds at wound surfaces requiring haemostatic therapy as assessed by the anaesthesiologist and surgeon by inspecting the operative field and/or (2) intraoperative or postoperative (during the first 24 postoperative hours) blood loss exceeding 250 mL/hour or 50 mL/10 min | | Pregnancy | | 72(8)  Vs  70(8) | | CV disease | | Cardiac Surgery | - ROTEM + PLT MAPPING - Control - Tranexamic acid - Restrictive Threshold - Cell Salvage | the number of transfused units of packed erythrocytes during the period between  inclusion into the study and 24 hours after ICU admission | •The number of transfused units of FFP, platelet concentrates and any other administered haemostatic therapy during the period between inclusion into the study and 24 hours after ICU admission  • Volume of intraoperatively and up to 24 hours postoperatively re-transfused salvaged  washed erythrocytes  • Postoperative chest tube blood loss 6, 12, and 24 hours after ICU admission  • Lowest haemoglobin concentration between inclusion into the study and 24 hours after ICU admission  • Number of re-thoracotomies during the first 24 postoperative hours  • PaO2/FiO2 indices at 2, 4, 12, and 24 hours after ICU admission  • Postoperative time of mechanical ventilation  • Length of ICU stay and hospital stay  • Incidence of acute renal failure, sepsis, thromboembolism, and allergic complications  • Mortality during a 6-month follow-up  • Costs of haemostatic therapy as prescribed by local pharmacy and blood bank |
| Westbrook 2009^1809^ | | - Australia - English - 2009 - Single-Centre - 69 - All patients presenting for cardiac surgery with the exception of lung transplantation | | None stated | | 66.3  Vs  61 | | CV disease  Anaemia | | Cardiac Surgery | - TEG + PLT MAPPING - Control - Tranexamic acid | - | Blood loss, intubation time (hours), minimum Hb (g/L), ICU stay, hospital stay (days) |

# Summary of Risk of bias table (eFigure 2)

The overall risk of bias is indicated by [green] for low risk of bias, [yellow] for unclear risk of bias, and [red] for high risk of bias. The results are expressed as percentages, with 396 studies included. For the details of the criteria used for rating please see: Higgins JPT, et al, 2011, Assessing risk of bias in included studies. Chapter 8. Cochrane Handbook for Systematic Reviews of Interventions Version 5.10: The Cochrane Collaboration.

**
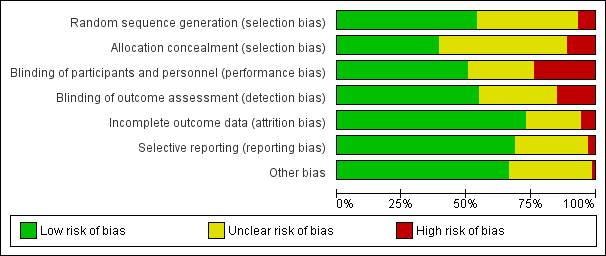
**

## Iron treatment


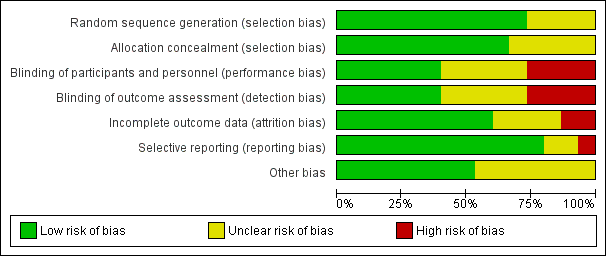


## Cell Salvage


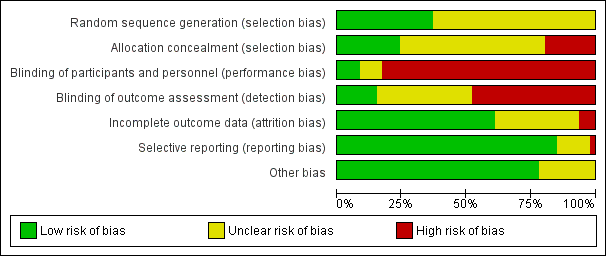


## Restrictive vs. Liberal


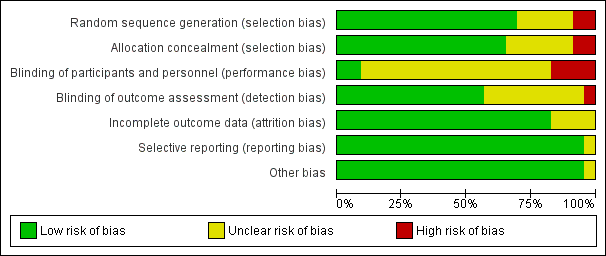


## Tranexamic Acid


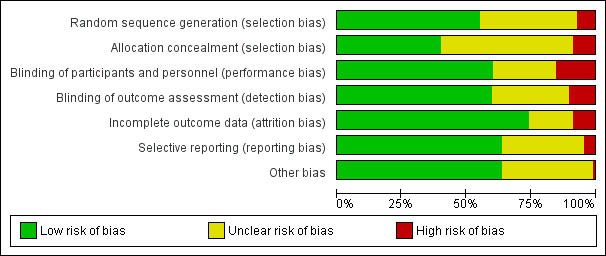


## Point of Care testing


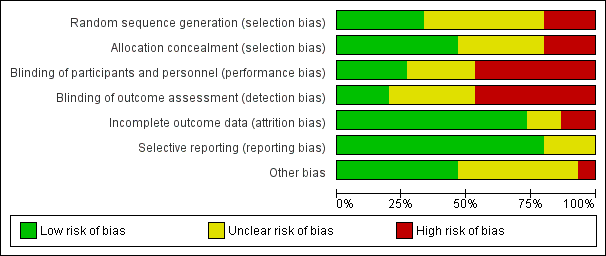


# Risk of bias table of included studies (eFigure 3)

The colours indicate [green] for low risk of bias, [yellow] for unclear risk of bias, and [red] for high risk of bias for each individual study. A total of 396 studies are included and described. For the details of the criteria used for rating please see: Higgins JPT, et al, 2011, Assessing risk of bias in included studies. Chapter 8. Cochrane Handbook for Systematic Reviews of Interventions Version 5.10: The Cochrane Collaboration.

## Iron treatment


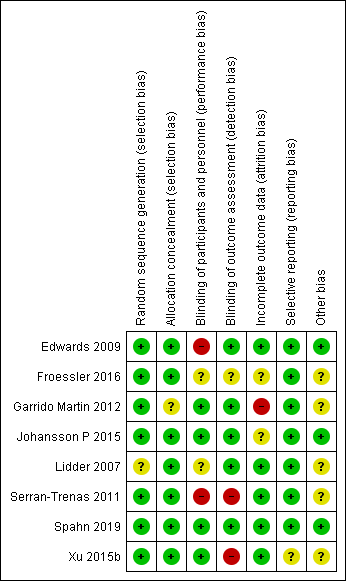


## Cell Salvage


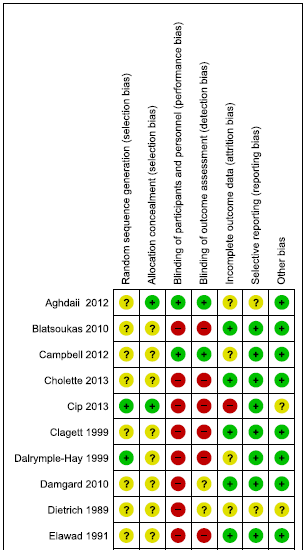


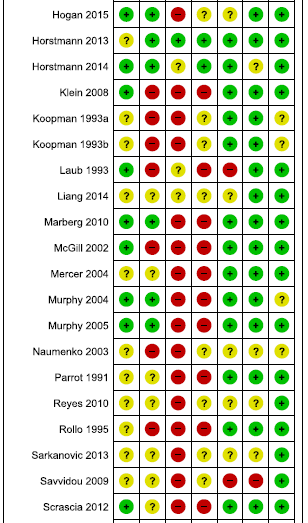


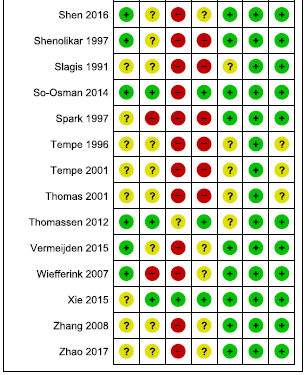


## Restrictive vs. Liberal


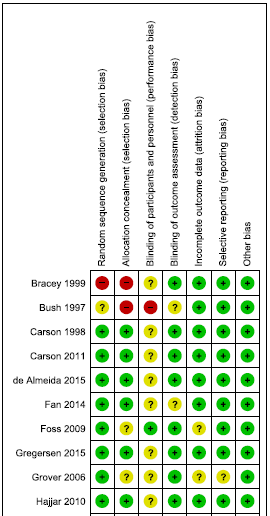


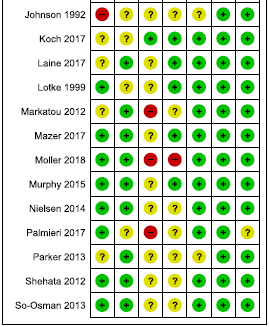


## Tranexamic Acid


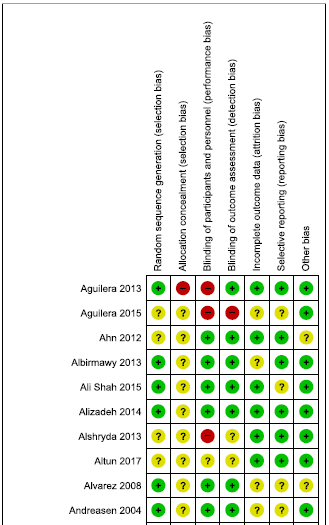


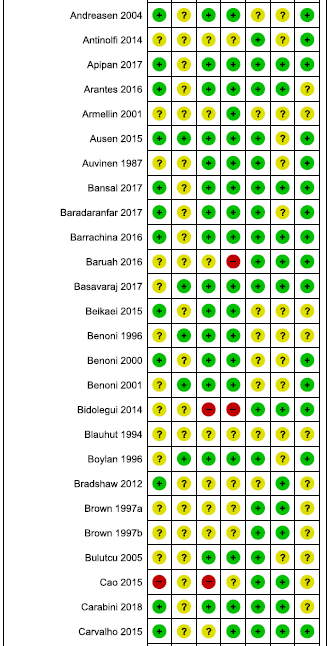


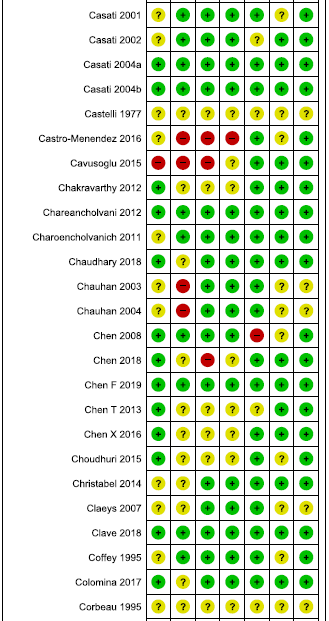


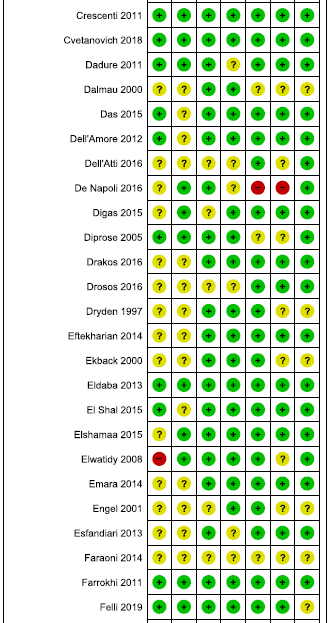


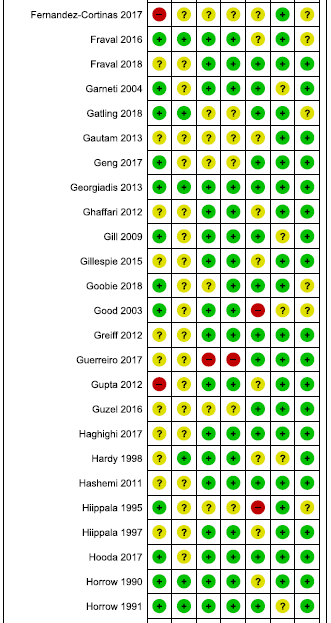


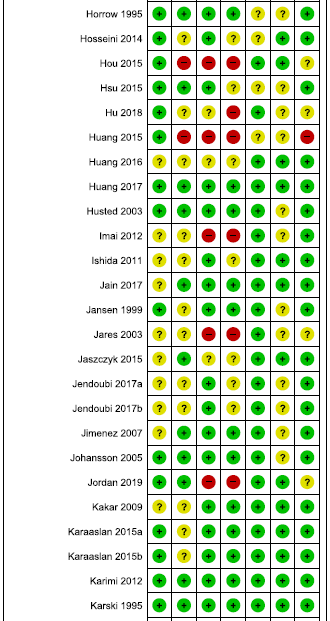


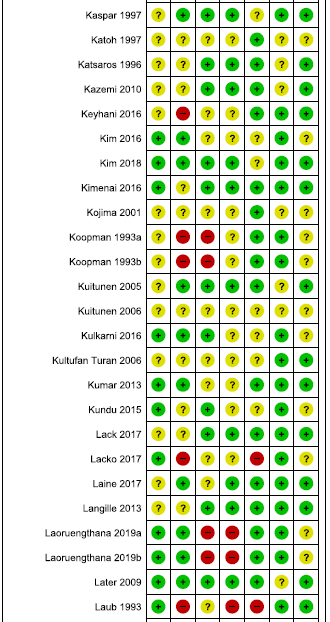


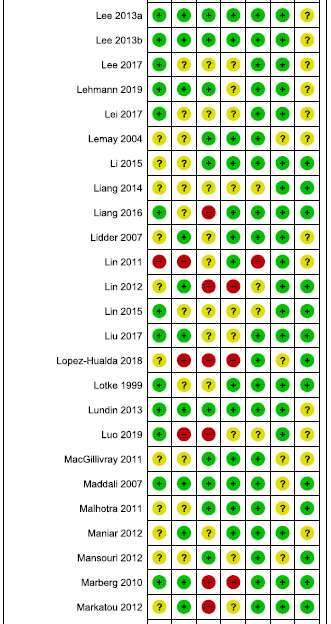


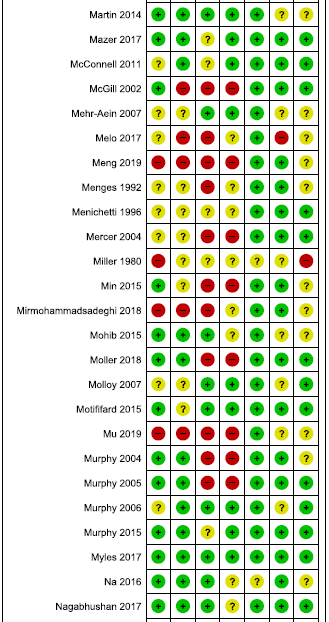


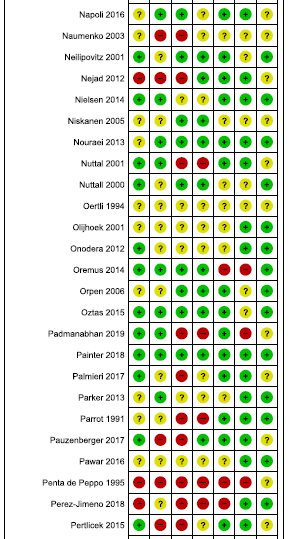


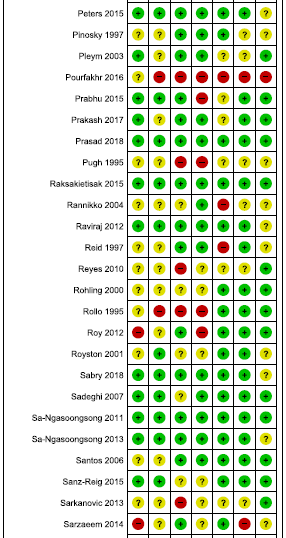


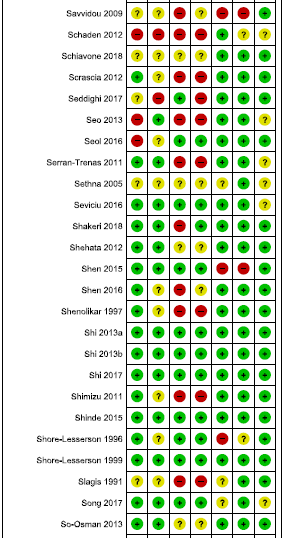


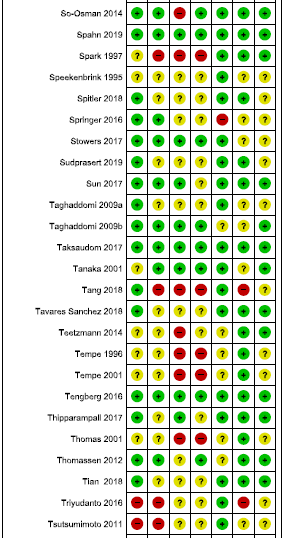


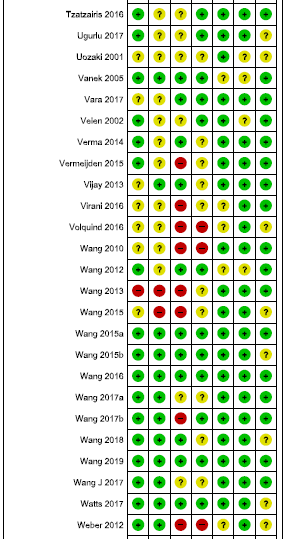


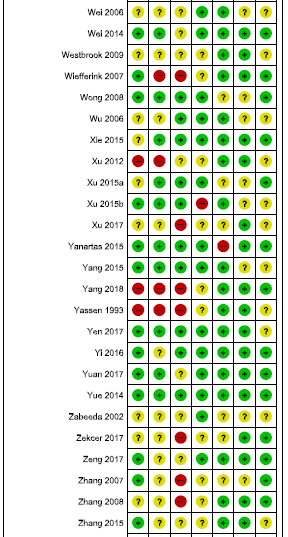


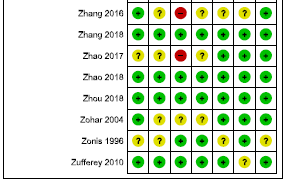


## Point of Care


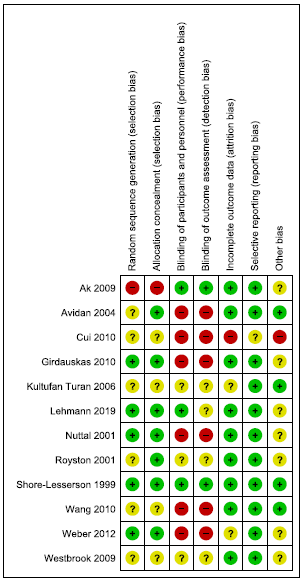


# Forest plots for transfusion and bleeding outcomes

## Risk of Transfusion (eFigure4a)

Summary Forest plots of pooled effect estimates for all PBM interventions combined as well as for the individual interventions: Iron treatment, Restrictive vs. Liberal threshold, Tranexamic Acid, Cell Salvage, and Point of Care testing) versus controls for the outcome frequency of red blood cells transfusions with heterogeneity expressed as I^2^. The results are expressed as: Risk Ratio (RR) along with 95% confidence intervals (CI) and Random effects. The heterogeneity is expressed as I^2^, with P<0.05 considered statistically significant.


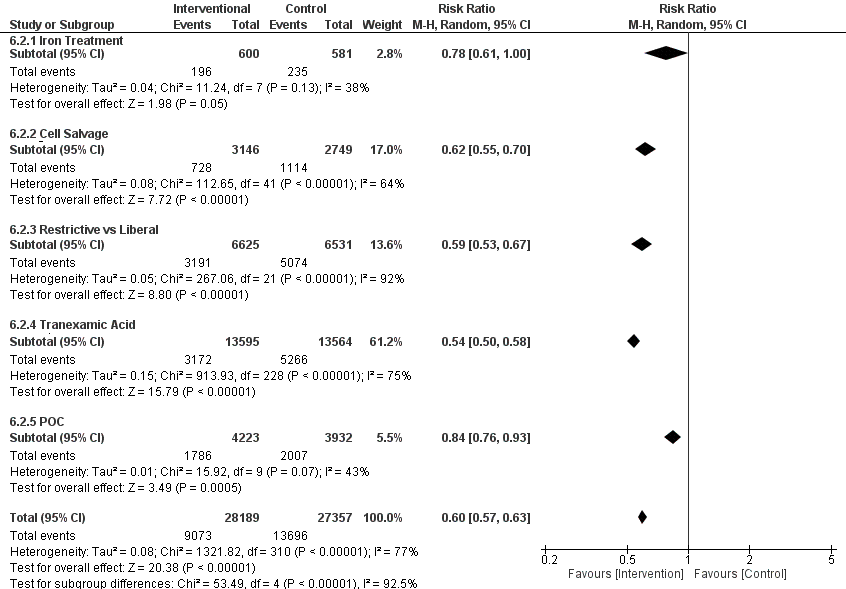


## Risk of receiving red cell transfusion GRADE assessment (eTable 15a)

| **Certainty assessment** | | | | | | | | | | | | | | | | | | | | | | **№ of patients** | | | | | | | | | **Effect** | | | | | | | **Certainty** | **Importance** |
| --- | --- | --- | --- | --- | --- | --- | --- | --- | --- | --- | --- | --- | --- | --- | --- | --- | --- | --- | --- | --- | --- | --- | --- | --- | --- | --- | --- | --- | --- | --- | --- | --- | --- | --- | --- | --- | --- | --- | --- |
| **№ of studies** | | | **Study design** | **Risk of bias** | | | | **Inconsistency** | | | | **Indirectness** | | | | | **Imprecision** | | **Other considerations** | | | **Combined blood management interventions** | | | | | **standard care** | | | | **Relative (95% CI)** | | | | | **Absolute (95% CI)** | |
| **Iron Therapy** | | | | | | | | | | | | | | | | | | | | | | | | | | | | | | | | | | | | | | | |
| 9 | randomised trials | | | | | serious ^a^ | | | | | not serious | | | | not serious ^b^ | | | not serious | | | none | | | 196/600 (32.7%) | | | | | 235/581 (40.4%) | | | | | **RR 0.78** (0.61 to 1.00) | | | **89 fewer per 1,000** (from 158 fewer to 0 fewer) | ⨁⨁⨁◯ MODERATE | IMPORTANT |
| **Cell Salvage** | | | | | | | | | | | | | | | | | | | | | | | | | | | | | | | | | | | | | | | |
| 43 | randomised trials | | | | | | serious ^a^ | | | | not serious | | | not serious | | | not serious | | | none | | 728/3146 (23.1%) | | | | | 1114/2749 (40.5%) | | | | | | | **RR 0.62** (0.55 to 0.70) | | | **154 fewer per 1,000** (from 182 fewer to 122 fewer) | ⨁⨁⨁◯ MODERATE | IMPORTANT |
| **Restrictive vs. Liberal transfusion strategy** | | | | | | | | | | | | | | | | | | | | | | | | | | | | | | | | | | | | | | | |
| 21 | randomised trials | | | | | not serious ^a^ | | | | | not serious ^b^ | | | not serious | | not serious | | | | strong association | | 3191/6625 (48.2%) | | | | 5074/6531 (77.7%) | | | | | | | **RR 0.59** (0.53 to 0.67) | | | | **319 fewer per 1,000** (from 365 fewer to 256 fewer) | ⨁⨁⨁⨁ HIGH | IMPORTANT |
| **Tranexamic Acid** | | | | | | | | | | | | | | | | | | | | | | | | | | | | | | | | | | | | | | | |
| 224 | randomised trials | | | | not serious ^a^ | | | | not serious | | | not serious | | | | | serious ^b^ | | | none | | | | 3212/13680 (23.5%) | | | | 5327/13619 (39.1%) | | | | | | **RR 0.54** (0.50 to 0.59) | | | **180 fewer per 1,000** (from 196 fewer to 160 fewer) | ⨁⨁⨁◯ MODERATE | IMPORTANT |
| **Point of care (POC)** | | | | | | | | | | | | | | | | | | | | | | | | | | | | | | | | | | | | | | | |
| 10 | | randomised trials | | | serious ^a^ | | | | | not serious | | | not serious | | | | not serious | | | none | | | | 1786/4223 (42.3%) | 2007/3932 (51.0%) | | | | | **RR 0.84** (0.76 to 0.93) | | | | | **82 fewer per 1,000** (from 123 fewer to 36 fewer) | | | ⨁⨁⨁◯ MODERATE | IMPORTANT |
| **Combined Interventions** | | | | | | | | | | | | | | | | | | | | | | | | | | | | | | | | | | | | | | | |
| 306 | | randomised trials | | | | | serious ^a^ | | | | not serious | | | not serious | | | serious ^b^ | | | publication bias strongly suspected | | | 8988/27743 (32.4%) | | | | | 13573/26942 (50.4%) | | | | **RR 0.60** (0.57 to 0.63) | | | | **202 fewer per 1,000** (from 217 fewer to 186 fewer) | | ⨁◯◯◯ VERY LOW | IMPORTANT |

**Legend:**

**CI:** Confidence interval; **RR:** Risk ratio; **SMD:** Standardised mean difference; **MD:** Mean difference

**Iron therapy**

#### Explanations

a. Moderate risk of performance and detection bias.

b. Keeler 2017 and Khalafallah 2012 compared different administration methods between groups.

c. High risk of performance bias, moderate risk of detection bias.

d. Different clinical settings.

e. Wide confidence interval - included potential for harm and benefit.

f. Blinding unlikely. Early Termination. Unequal arms due to simple randomisation.

g. Different clinical settings (orthopaedic and general surgery).

h. Confidence intervals included potential for important harm and benefit. Small population size.

i. A single trial (Bernabeu Wittel 2016) conducted in an orthopaedic clinical setting.

j. Risk of performance and detection bias

**Cell Salvage**

#### Explanations

a. High risk of performance and detection bias.

b. Unclear risk of selection bias, high risk of performance and detection bias.

c. Several trials with small population size and low number of events. Wide confidence intervals.

d. Different kind of settings and interventions considered. Control group seem to be favoured in cardiac surgery settings (possibly related to the effect of salvaged blood reinfusion after CPB), while orthopaedic settings (e.g. Elawad 1991, Horstmann 2013) favour cell salvage.

e. Klein 2008 reported patients with creatinine higher than 200mmol/l 2

f. Different clinical settings considered.

g. Reported as "use of inotropes" in most of the studies.

h. Cholette 2013 conducted on a paediatric population.

**Restrictive vs Liberal transfusion strategy**

#### Explanations

a. Unclear risk of performance bias, due to the nature of the intervention itself

b. Tests for heterogeneity show a low p-value and I^2^>80%., however, this is not considered important as the direction of effect is the same for all studies

c. Different surgical disciplines considered in the analysis.

d. I^2^> 80%, p<0·05

e. Large variability in results, several trials had small population size and low number of events.

f. De Almeyda 2015 conducted on a population of oncology patients.

g. Variation in outcome definition

h. Different definition of considered outcomes.

**Tranexamic acid**

#### Explanations

a. Several low quality trials, albeit with a small weight in terms of the results.

b. Several studies with small sample size and low number of events.

c. May be due to several kinds of surgical disciplines considered in the analysis.

d. Different definitions of the outcome considered.

**Point of care testing (POC)**

#### Explanations

a. Moderate risk of selection bias, high risk of performance and detection bias.

b. Avidan 2004 compares POC testing with a laboratory-guided algorithm

c. Different clinical settings considered and different kind of POC testing.

d. Wide confidence intervals.

e. Small population size, low number of events.

f. Risk of performance and detection bias.

g. High risk of performance, detection and attrition bias.

h. A single RCT conducted in a German cardiac surgery centre

i. High risk of selection bias.

j. A single RCT conducted in a Turkish cardiac centre on patients scheduled for CABG surgery.

k. Cui 2010 had a population of paediatric patients undergoing complex cardiac procedures.

**Combined Interventions**

#### Explanations

a. Moderate risk of Performance Bias coming from POC and Cell Salvage trials.

b. Several studies with small sample size and low number of events.

c. A restrictive transfusion threshold had an opposite effect from the other interventions, with an increase in length of stay.

## Number of red cells transfused (eFigure4b)

Summary Forest plots of pooled effect estimates for all PBM interventions combined as well as for the individual interventions: Iron treatment, Restrictive vs. Liberal threshold, Tranexamic Acid, Cell Salvage, and Point of Care testing) versus controls for the outcome volume of red blood cells transfused expressed in units, with heterogeneity expressed as I^2^. The results are expressed as: Standardised Mean Difference (SMD) along with 95% confidence intervals (CI) and Random effects. The heterogeneity is expressed as I^2^, with P<0.05 considered statistically significant.


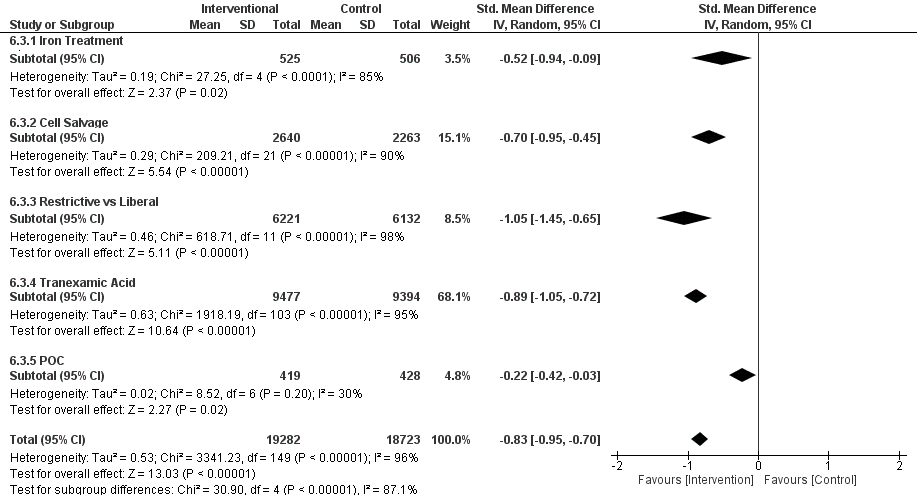


## Number of red cells transfused GRADE assessment (eTable 15b)

| **Certainty assessment** | | | | | | | | | | | | | | | | | | | | | | | | | | | | **№ of patients** | | | | | | | | | | **Effect** | | | | | | **Certainty** | | | **Importance** |
| --- | --- | --- | --- | --- | --- | --- | --- | --- | --- | --- | --- | --- | --- | --- | --- | --- | --- | --- | --- | --- | --- | --- | --- | --- | --- | --- | --- | --- | --- | --- | --- | --- | --- | --- | --- | --- | --- | --- | --- | --- | --- | --- | --- | --- | --- | --- | --- |
| **№ of studies** | | | **Study design** | **Risk of bias** | | | | **Inconsistency** | | | **Indirectness** | | | | | | **Imprecision** | | | | | | **Other considerations** | | | | | **Combined blood management interventions** | | | | | **standard care** | | | | | **Relative (95% CI)** | | | | | **Absolute (95% CI)** |
| **Iron Therapy** | | | | | | | | | | | | | | | | | | | | | | | | | | | | | | | | | | | | | | | | | | | | | | | |
| 9 | randomised trials | | | | | | serious ^a^ | | | not serious | | | | not serious ^b^ | | | | | not serious | | | | none | | | | | 525 | | | | | 506 | | | | - | | | | | SMD **0.52 SD fewer** (0.94 fewer to 0.09 fewer) | | ⨁⨁⨁◯ MODERATE | | IMPORTANT | |
| **Cell Salvage** | | | | | | | | | | | | | | | | | | | | | | | | | | | | | | | | | | | | | | | | | | | | | | | |
| 31 | randomised trials | | | | | | serious ^a^ | | | not serious | | | not serious | | | | | not serious | | | | | none | | | | 2640 | | | | | 2263 | | | | - | | | | | SMD **0.7 SD fewer** (0.95 fewer to 0.45 fewer) | | | ⨁⨁⨁◯ MODERATE | | IMPORTANT | |
| **Restrictive vs. Liberal transfusion strategy** | | | | | | | | | | | | | | | | | | | | | | | | | | | | | | | | | | | | | | | | | | | | | | | |
| 17 | randomised trials | | | | | serious ^a^ | | | | not serious ^b^ | | | not serious | | | | | not serious | | | | none | | | | 6221 | | | | | 6132 | | | | - | | | | | SMD **1.05 SD lower** (1.45 lower to 0.65 lower) | | | | | ⨁⨁⨁◯ MODERATE | | IMPORTANT |
| **Tranexamic Acid** | | | | | | | | | | | | | | | | | | | | | | | | | | | | | | | | | | | | | | | | | | | | | | | |
| 153 | randomised trials | | | | not serious ^a^ | | | | not serious | | not serious | | | | serious ^b^ | | | | | none | | | | 9537 | | | | | 9454 | | | | | | | | - | | | | SMD **0.89 SD lower** (1.05 lower to 0.73 lower) | | | ⨁⨁⨁◯ MODERATE | | | IMPORTANT |
| **Point of care (POC)** | | | | | | | | | | | | | | | | | | | | | | | | | | | | | | | | | | | | | | | | | | | | | | | |
| 11 | | randomised trials | | | serious ^a^ | | | | | not serious | | not serious | | | | not serious | | | | | none | | | | 419 | | | | | 428 | | | | - | | | | | SMD **0.22 SD fewer** (0.42 fewer to 0.03 fewer) | | | | | ⨁⨁⨁◯ MODERATE | | | IMPORTANT |
| **Combined Interventions** | | | | | | | | | | | | | | | | | | | | | | | | | | | | | | | | | | | | | | | | | | | | | | | |
| 221 | | randomised trials | | | serious ^a^ | | | | | not serious | | not serious | | | | serious ^b^ | | | | | publication bias strongly suspected | | | | 19065 | | | | | 18533 | | | | - | | | | | SMD **0.83 lower** (0.96 lower to 0.7 lower) | | | | | ⨁◯◯◯ VERY LOW | | | IMPORTANT |

**Legend:**

**CI:** Confidence interval; **RR:** Risk ratio; **SMD:** Standardised mean difference; **MD:** Mean difference

**Iron therapy**

#### Explanations

a. Moderate risk of performance and detection bias.

b. Keeler 2017 and Khalafallah 2012 compared different administration methods between groups.

c. High risk of performance bias, moderate risk of detection bias.

d. Different clinical settings.

e. Wide confidence interval - included potential for harm and benefit.

f. Blinding unlikely. Early Termination. Unequal arms due to simple randomisation.

g. Different clinical settings (orthopaedic and general surgery).

h. Confidence intervals included potential for important harm and benefit. Small population size.

i. A single trial (Bernabeu Wittel 2016) conducted in an orthopaedic clinical setting.

j. Risk of performance and detection bias

**Cell Salvage**

#### Explanations

a. High risk of performance and detection bias.

b. Unclear risk of selection bias, high risk of performance and detection bias.

c. Several trials with small population size and low number of events. Wide confidence intervals.

d. Different kind of settings and interventions considered. Control group seem to be favoured in cardiac surgery settings (possibly related to the effect of salvaged blood reinfusion after CPB), while orthopaedic settings (e.g. Elawad 1991, Horstmann 2013) favour cell salvage.

e. Klein 2008 reported patients with creatinine higher than 200mmol/l 2

f. Different clinical settings considered.

g. Reported as "use of inotropes" in most of the studies.

h. Cholette 2013 conducted on a paediatric population.

**Restrictive vs Liberal transfusion strategy**

#### Explanations

a. Unclear risk of performance bias, due to the nature of the intervention itself

b. Tests for heterogeneity show a low p-value and I^2^>80%., however, this is not considered important as the direction of effect is the same for all studies

c. Different surgical disciplines considered in the analysis.

d. I^2^> 80%, p<0·05

e. Large variability in results, several trials had small population size and low number of events.

f. De Almeyda 2015 conducted on a population of oncology patients.

g. Variation in outcome definition

h. Different definition of considered outcomes.

**Tranexamic acid**

#### Explanations

a. Several low quality trials, albeit with a small weight in terms of the results.

b. Several studies with small sample size and low number of events.

c. May be due to several kinds of surgical disciplines considered in the analysis.

d. Different definitions of the outcome considered.

**Point of care testing (POC)**

#### Explanations

a. Moderate risk of selection bias, high risk of performance and detection bias.

b. Avidan 2004 compares POC testing with a laboratory-guided algorithm

c. Different clinical settings considered and different kind of POC testing.

d. Wide confidence intervals.

e. Small population size, low number of events.

f. Risk of performance and detection bias.

g. High risk of performance, detection and attrition bias.

h. A single RCT conducted in a German cardiac surgery centre

i. High risk of selection bias.

j. A single RCT conducted in a Turkish cardiac centre on patients scheduled for CABG surgery.

k. Cui 2010 had a population of paediatric patients undergoing complex cardiac procedures.

**Combined Interventions**

#### Explanations

a. Moderate risk of Performance Bias coming from POC and Cell Salvage trials.

b. Several studies with small sample size and low number of events.

c. A restrictive transfusion threshold had an opposite effect from the other interventions, with an increase in length of stay.

## Risk of FFP transfusion (eFigure4c)

Summary Forest plots of pooled effect estimates for all PBM interventions combined as well as for the individual interventions: Iron treatment, Restrictive vs. Liberal threshold, Tranexamic Acid, Cell Salvage, and Point of Care testing) versus controls for the outcome of FFP transfusions with heterogeneity of effect estimates expressed as I^2^. The results are expressed as: Risk Ratio (RR) along with 95% confidence intervals (CI) and Random effects. The heterogeneity is expressed as I^2^, with P<0.05 considered statistically significant.

**
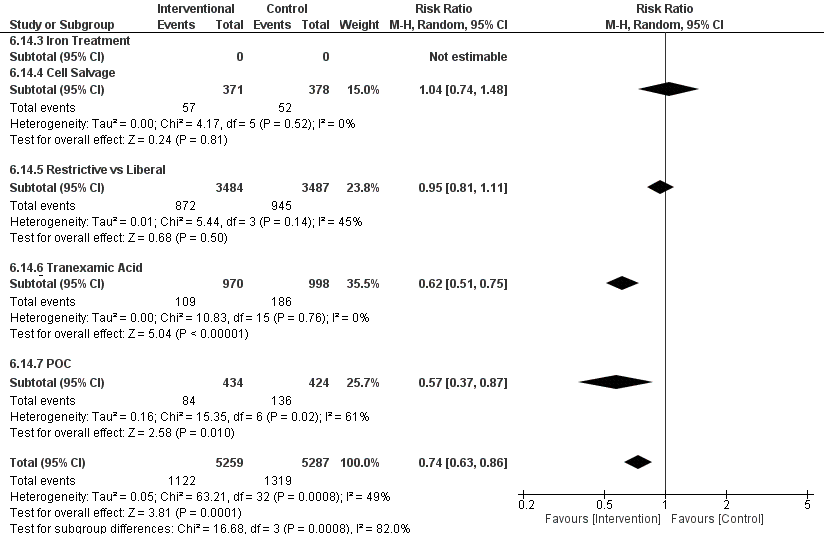
**

## Risk of FFP transfusion GRADE assessment (eTable 15c)

| **Certainty assessment** | | | | | | | | | | | | | | **№ of patients** | | | | | **Effect** | | | **Certainty** | | **Importance** |
| --- | --- | --- | --- | --- | --- | --- | --- | --- | --- | --- | --- | --- | --- | --- | --- | --- | --- | --- | --- | --- | --- | --- | --- | --- |
| **№ of studies** | | | **Study design** | **Risk of bias** | **Inconsistency** | | | **Indirectness** | | **Imprecision** | | **Other considerations** | | **Combined blood management interventions** | | **standard care** | | | **Relative (95% CI)** | | **Absolute (95% CI)** |
| **Iron Therapy** | | | | | | | | | | | | | | | | | | | | | | | | |
| 0 | | NA | | NA | NA | | | | NA | | NA | NA | | NA | NA | | NA | | | | NA | NA | | NA |
| **Cell Salvage** | | | | | | | | | | | | | | | | | | | | | | | | |
| 6 | | randomised trials | | serious ^b^ | | not serious | | | not serious | serious ^c^ | | none | | 57/371 (15.4%) | 52/378 (13.8%) | | **RR 1.04** (0.74 to 1.48) | | | **6 more per 1,000** (from 36 fewer to 66 more) | | ⨁⨁◯◯ LOW | IMPORTANT | |
| **Restrictive vs. Liberal transfusion strategy** | | | | | | | | | | | | | | | | | | | | | | | | |
| 3 | | randomised trials | | not serious ^a^ | | not serious | | | not serious | not serious | | none | | 872/3484 (25.0%) | 945/3487 (27.1%) | | **RR 0.95** (0.81 to 1.11) | | | **14 fewer per 1,000** (from 51 fewer to 30 more) | | ⨁⨁⨁⨁ HIGH | IMPORTANT | |
| **Tranexamic Acid** | | | | | | | | | | | | | | | | | | | | | | | | |
| 15 | | randomised trials | | not serious ^a^ | | | not serious | | not serious | | serious ^b^ | none | | 110/995 (11.1%) | | 190/1023 (18.6%) | **RR 0.61** (0.51 to 0.74) | | | **72 fewer per 1,000** (from 91 fewer to 48 fewer) | | ⨁⨁⨁◯ MODERATE | IMPORTANT | |
| **Point of care (POC)** | | | | | | | | | | | | | | | | | | | | | | | | |
| 7 | randomised trials | | | serious ^a^ | | not serious ^b^ | | not serious | | | not serious | none | 84/434 (19.4%) | | | 136/424 (32.1%) | | **RR 0.57** (0.37 to 0.87) | | | **138 fewer per 1,000** (from 202 fewer to 42 fewer) | ⨁⨁⨁◯ MODERATE | | IMPORTANT |
| **Combined Interventions** | | | | | | | | | | | | | | | | | | | | | | | | |
| 31 | randomised trials | | | serious ^a^ | not serious | | | | not serious | | serious ^b^ | publication bias strongly suspected | | 1083/5132 (21.1%) | | 1262/5158 (24.5%) | | **RR 0.75** (0.63 to 0.88) | | | **61 fewer per 1,000** (from 91 fewer to 29 fewer) | ⨁◯◯◯ VERY LOW | | IMPORTANT |

**Legend:**

**CI:** Confidence interval; **RR:** Risk ratio; **SMD:** Standardised mean difference; **MD:** Mean difference

**Iron therapy**

#### Explanations

a. Moderate risk of performance and detection bias.

b. Keeler 2017 and Khalafallah 2012 compared different administration methods between groups.

c. High risk of performance bias, moderate risk of detection bias.

d. Different clinical settings.

e. Wide confidence interval - included potential for harm and benefit.

f. Blinding unlikely. Early Termination. Unequal arms due to simple randomisation.

g. Different clinical settings (orthopaedic and general surgery).

h. Confidence intervals included potential for important harm and benefit. Small population size.

i. A single trial (Bernabeu Wittel 2016) conducted in an orthopaedic clinical setting.

j. Risk of performance and detection bias

**Cell Salvage**

#### Explanations

a. High risk of performance and detection bias.

b. Unclear risk of selection bias, high risk of performance and detection bias.

c. Several trials with small population size and low number of events. Wide confidence intervals.

d. Different kind of settings and interventions considered. Control group seem to be favoured in cardiac surgery settings (possibly related to the effect of salvaged blood reinfusion after CPB), while orthopaedic settings (e.g. Elawad 1991, Horstmann 2013) favour cell salvage.

e. Klein 2008 reported patients with creatinine higher than 200mmol/l 2

f. Different clinical settings considered.

g. Reported as "use of inotropes" in most of the studies.

h. Cholette 2013 conducted on a paediatric population.

**Restrictive vs Liberal transfusion strategy**

#### Explanations

a. Unclear risk of performance bias, due to the nature of the intervention itself

b. Tests for heterogeneity show a low p-value and I^2^>80%., however, this is not considered important as the direction of effect is the same for all studies

c. Different surgical disciplines considered in the analysis.

d. I^2^> 80%, p<0·05

e. Large variability in results, several trials had small population size and low number of events.

f. De Almeyda 2015 conducted on a population of oncology patients.

g. Variation in outcome definition

h. Different definition of considered outcomes.

**Tranexamic acid**

#### Explanations

a. Several low quality trials, albeit with a small weight in terms of the results.

b. Several studies with small sample size and low number of events.

c. May be due to several kinds of surgical disciplines considered in the analysis.

d. Different definitions of the outcome considered.

**Point of care testing (POC)**

#### Explanations

a. Moderate risk of selection bias, high risk of performance and detection bias.

b. Avidan 2004 compares POC testing with a laboratory-guided algorithm

c. Different clinical settings considered and different kind of POC testing.

d. Wide confidence intervals.

e. Small population size, low number of events.

f. Risk of performance and detection bias.

g. High risk of performance, detection and attrition bias.

h. A single RCT conducted in a German cardiac surgery centre

i. High risk of selection bias.

j. A single RCT conducted in a Turkish cardiac centre on patients scheduled for CABG surgery.

k. Cui 2010 had a population of paediatric patients undergoing complex cardiac procedures.

**Combined Interventions**

#### Explanations

a. Moderate risk of Performance Bias coming from POC and Cell Salvage trials.

b. Several studies with small sample size and low number of events.

c. A restrictive transfusion threshold had an opposite effect from the other interventions, with an increase in length of stay.

## Risk of Platelets transfusion (eFigure4d)

Summary Forest plots of pooled effect estimates for all PBM interventions combined as well as for the individual interventions: Iron treatment, Restrictive vs. Liberal threshold, Tranexamic Acid, Cell Salvage, and Point of Care testing) versus controls for the outcome frequency of platelet transfusions with heterogeneity expressed as I^2^. The results are expressed as: Risk Ratio (RR) along with 95% confidence intervals (CI) and Random effects. The heterogeneity is expressed as I^2^, with P<0.05 considered statistically significant.


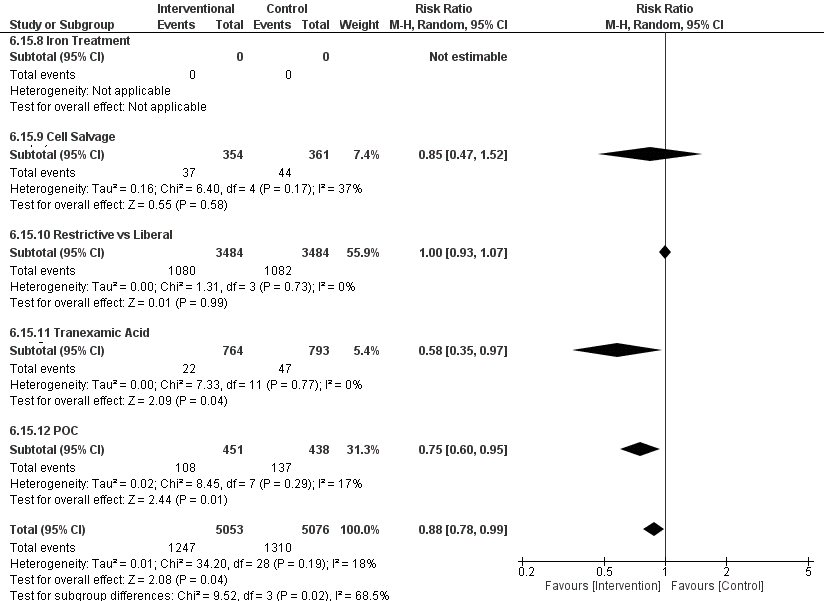


## Risk of Platelets transfusion GRADE assessment (eTable 15d)

| **Certainty assessment** | | | | | | | | | | | | | | | | **№ of patients** | | | **Effect** | | | | | **Certainty** | | **Importance** |
| --- | --- | --- | --- | --- | --- | --- | --- | --- | --- | --- | --- | --- | --- | --- | --- | --- | --- | --- | --- | --- | --- | --- | --- | --- | --- | --- |
| **№ of studies** | | | **Study design** | | **Risk of bias** | **Inconsistency** | | | **Indirectness** | | | **Imprecision** | | | **Other considerations** | **Combined blood management interventions** | **standard care** | | **Relative (95% CI)** | | **Absolute (95% CI)** | | |
| **Iron Therapy** | | | | | | | | | | | | | | | | | | | | | | | | | | |
| 0 | | NA | | | NA | NA | | | | | NA | | NA | | NA | NA | | NA | NA | | NA | | | NA | | NA |
| **Cell Salvage** | | | | | | | | | | | | | | | | | | | | | | | | | | |
| 5 | | randomised trials | | serious ^b^ | | | not serious | | | not serious | | serious ^c^ | | none | | 37/354 (10.5%) | 44/361 (12.2%) | | **RR 0.85** (0.47 to 1.52) | | | **18 fewer per 1,000** (from 65 fewer to 63 more) | | ⨁⨁◯◯ LOW | | IMPORTANT |
| **Restrictive vs. Liberal transfusion strategy** | | | | | | | | | | | | | | | | | | | | | | | | | | |
| 3 | | randomised trials | | not serious ^a^ | | | not serious | | | not serious | | not serious | | none | | 1080/3484 (31.0%) | 1082/3484 (31.1%) | | **RR 1.00** (0.93 to 1.07) | | | **0 fewer per 1,000** (from 22 fewer to 22 more) | | ⨁⨁⨁⨁ HIGH | | IMPORTANT |
| **Tranexamic Acid** | | | | | | | | | | | | | | | | | | | | | | | | | | |
| 11 | randomised trials | | | not serious ^a^ | | not serious | | | | not serious | | serious ^b^ | | none | | 24/789 (3.0%) | 52/818 (6.4%) | | **RR 0.56** (0.35 to 0.91) | | | **28 fewer per 1,000** (from 41 fewer to 6 fewer) | | ⨁⨁⨁◯ MODERATE | | IMPORTANT |
| **Point of care (POC)** | | | | | | | | | | | | | | | | | | | | | | | | | | |
| 8 | | randomised trials | | serious ^a^ | | | not serious ^b^ | | | not serious | | not serious | | none | | 108/451 (23.9%) | 137/438 (31.3%) | | **RR 0.75** (0.60 to 0.95) | | | **78 fewer per 1,000** (from 125 fewer to 16 fewer) | | ⨁⨁⨁◯ MODERATE | | IMPORTANT |
| **Combined Interventions** | | | | | | | | | | | | | | | | | | | | | | | | | | |
| 27 | | randomised trials | | serious ^a^ | | | | not serious | | not serious | | serious ^b^ | | publication bias strongly suspected | | 1240/4926 (25.2%) | 1311/4947 (26.5%) | | **RR 0.87** (0.77 to 0.98) | **34 fewer per 1,000** (from 61 fewer to 5 fewer) | | | ⨁◯◯◯ VERY LOW | | IMPORTANT | |

**Legend:**

**CI:** Confidence interval; **RR:** Risk ratio; **SMD:** Standardised mean difference; **MD:** Mean difference

**Iron therapy**

#### Explanations

a. Moderate risk of performance and detection bias.

b. Keeler 2017 and Khalafallah 2012 compared different administration methods between groups.

c. High risk of performance bias, moderate risk of detection bias.

d. Different clinical settings.

e. Wide confidence interval - included potential for harm and benefit.

f. Blinding unlikely. Early Termination. Unequal arms due to simple randomisation.

g. Different clinical settings (orthopaedic and general surgery).

h. Confidence intervals included potential for important harm and benefit. Small population size.

i. A single trial (Bernabeu Wittel 2016) conducted in an orthopaedic clinical setting.

j. Risk of performance and detection bias

**Cell Salvage**

#### Explanations

a. High risk of performance and detection bias.

b. Unclear risk of selection bias, high risk of performance and detection bias.

c. Several trials with small population size and low number of events. Wide confidence intervals.

d. Different kind of settings and interventions considered. Control group seem to be favoured in cardiac surgery settings (possibly related to the effect of salvaged blood reinfusion after CPB), while orthopaedic settings (e.g. Elawad 1991, Horstmann 2013) favour cell salvage.

e. Klein 2008 reported patients with creatinine higher than 200mmol/l 2

f. Different clinical settings considered.

g. Reported as "use of inotropes" in most of the studies.

h. Cholette 2013 conducted on a paediatric population.

**Restrictive vs Liberal transfusion strategy**

#### Explanations

a. Unclear risk of performance bias, due to the nature of the intervention itself

b. Tests for heterogeneity show a low p-value and I^2^>80%., however, this is not considered important as the direction of effect is the same for all studies

c. Different surgical disciplines considered in the analysis.

d. I^2^> 80%, p<0·05

e. Large variability in results, several trials had small population size and low number of events.

f. De Almeyda 2015 conducted on a population of oncology patients.

g. Variation in outcome definition

h. Different definition of considered outcomes.

**Tranexamic acid**

#### Explanations

a. Several low quality trials, albeit with a small weight in terms of the results.

b. Several studies with small sample size and low number of events.

c. May be due to several kinds of surgical disciplines considered in the analysis.

d. Different definitions of the outcome considered.

**Point of care testing (POC)**

#### Explanations

a. Moderate risk of selection bias, high risk of performance and detection bias.

b. Avidan 2004 compares POC testing with a laboratory-guided algorithm

c. Different clinical settings considered and different kind of POC testing.

d. Wide confidence intervals.

e. Small population size, low number of events.

f. Risk of performance and detection bias.

g. High risk of performance, detection and attrition bias.

h. A single RCT conducted in a German cardiac surgery centre

i. High risk of selection bias.

j. A single RCT conducted in a Turkish cardiac centre on patients scheduled for CABG surgery.

k. Cui 2010 had a population of paediatric patients undergoing complex cardiac procedures.

**Combined Interventions**

#### Explanations

a. Moderate risk of Performance Bias coming from POC and Cell Salvage trials.

b. Several studies with small sample size and low number of events.

c. A restrictive transfusion threshold had an opposite effect from the other interventions, with an increase in length of stay.

## Perioperative blood loss (eFigure4e)

Summary Forest plots of pooled effect estimates for all PBM interventions combined as well as for the individual interventions: Iron treatment, Restrictive vs. Liberal threshold, Tranexamic Acid, Cell Salvage, and Point of Care testing) versus controls for the outcome volume of blood loss. The results are expressed as: Standardised Mean Difference (SMD) along with 95% confidence intervals (CI) and Random effects. The heterogeneity is expressed as I^2^, with P<0.05 considered statistically significant.


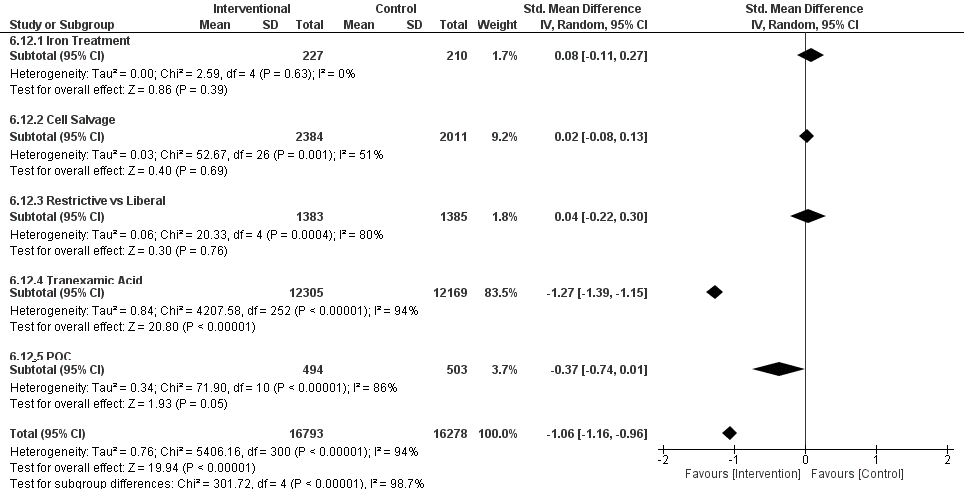


## Perioperative blood loss GRADE assessment (eTable 15e)

| **Certainty assessment** | | | | | | | | | | | | | | | | | | **№ of patients** | | | **Effect** | | **Certainty** | | **Importance** | | | |
| --- | --- | --- | --- | --- | --- | --- | --- | --- | --- | --- | --- | --- | --- | --- | --- | --- | --- | --- | --- | --- | --- | --- | --- | --- | --- | --- | --- | --- |
| **№ of studies** | **Study design** | | **Risk of bias** | | | **Inconsistency** | | **Indirectness** | | **Imprecision** | | | | | **Other considerations** | | | **Combined blood management interventions** | | **standard care** | **Relative (95% CI)** | **Absolute (95% CI)** |
| **Iron Therapy** | | | | | | | | | | | | | | | | | | | | | | | | | | | | |
| 6 | randomised trials | | | | serious ^c^ | | not serious ^d^ | not serious | | | | serious ^e^ | | | none | | | 227 | | 210 | - | SMD **0.08 SD more** (0.11 fewer to 0.27 more) | | ⨁⨁◯◯ LOW | | IMPORTANT | |
| **Cell Salvage** | | | | | | | | | | | | | | | | | | | | | | | | | | | | |
| 28 | randomised trials | | | | serious ^a^ | | serious ^d^ | | not serious | | | not serious | | | none | | | 2384 | | 2011 | - | SMD **0.02 SD more** (0.08 fewer to 0.13 more) | | ⨁⨁◯◯ LOW | | IMPORTANT | |
| **Restrictive vs. Liberal transfusion strategy** | | | | | | | | | | | | | | | | | | | | | | | | | | | | |
| 5 | randomised trials | | | | not serious ^a^ | | serious ^c,d^ | | not serious | | | serious ^e^ | | | none | | | 1383 | | 1385 | - | SMD **0.04 SD more** (0.22 fewer to 0.3 more) | | ⨁⨁◯◯ LOW | | IMPORTANT | |
| **Tranexamic Acid** | | | | | | | | | | | | | | | | | | | | | | | | | | | | |
| 261 | randomised trials | | | | not serious ^a^ | | not serious | | not serious | | serious ^b^ | | none | | | 12390 | | | 12254 | | - | SMD **1.26 SD fewer** (1.38 fewer to 1.14 fewer) | | ⨁⨁⨁◯ MODERATE | | IMPORTANT | |
| **Point of care (POC)** | | | | | | | | | | | | | | | | | | | | | | | | | | | | |
| 12 | randomised trials | | | serious ^a^ | | | serious ^c^ | | not serious | | serious ^d^ | | | none | | | 494 | | | 503 | - | SMD **0.37 SD fewer** (0.74 fewer to 0.01 more) | | ⨁◯◯◯ VERY LOW | | IMPORTANT |
| **Combined Interventions** | | | | | | | | | | | | | | | | | | | | | | | | | | | | |
| 311 | | randomised trials | | serious ^a^ | | | serious ^d^ | | not serious | | serious ^b^ | | | | publication bias strongly suspected | | 16347 | | | 15839 | - | SMD **1.06 lower** (1.17 lower to 0.95 lower) | | ⨁◯◯◯ VERY LOW | IMPORTANT | | | |

**Legend:**

**CI:** Confidence interval; **RR:** Risk ratio; **SMD:** Standardised mean difference; **MD:** Mean difference

**Iron therapy**

#### Explanations

a. Moderate risk of performance and detection bias.

b. Keeler 2017 and Khalafallah 2012 compared different administration methods between groups.

c. High risk of performance bias, moderate risk of detection bias.

d. Different clinical settings.

e. Wide confidence interval - included potential for harm and benefit.

f. Blinding unlikely. Early Termination. Unequal arms due to simple randomisation.

g. Different clinical settings (orthopaedic and general surgery).

h. Confidence intervals included potential for important harm and benefit. Small population size.

i. A single trial (Bernabeu Wittel 2016) conducted in an orthopaedic clinical setting.

j. Risk of performance and detection bias

**Cell Salvage**

#### Explanations

a. High risk of performance and detection bias.

b. Unclear risk of selection bias, high risk of performance and detection bias.

c. Several trials with small population size and low number of events. Wide confidence intervals.

d. Different kind of settings and interventions considered. Control group seem to be favoured in cardiac surgery settings (possibly related to the effect of salvaged blood reinfusion after CPB), while orthopaedic settings (e.g. Elawad 1991, Horstmann 2013) favour cell salvage.

e. Klein 2008 reported patients with creatinine higher than 200mmol/l 2

f. Different clinical settings considered.

g. Reported as "use of inotropes" in most of the studies.

h. Cholette 2013 conducted on a paediatric population.

**Restrictive vs Liberal transfusion strategy**

#### Explanations

a. Unclear risk of performance bias, due to the nature of the intervention itself

b. Tests for heterogeneity show a low p-value and I^2^>80%., however, this is not considered important as the direction of effect is the same for all studies

c. Different surgical disciplines considered in the analysis.

d. I^2^> 80%, p<0·05

e. Large variability in results, several trials had small population size and low number of events.

f. De Almeyda 2015 conducted on a population of oncology patients.

g. Variation in outcome definition

h. Different definition of considered outcomes.

**Tranexamic acid**

#### Explanations

a. Several low quality trials, albeit with a small weight in terms of the results.

b. Several studies with small sample size and low number of events.

c. May be due to several kinds of surgical disciplines considered in the analysis.

d. Different definitions of the outcome considered.

**Point of care testing (POC)**

#### Explanations

a. Moderate risk of selection bias, high risk of performance and detection bias.

b. Avidan 2004 compares POC testing with a laboratory-guided algorithm

c. Different clinical settings considered and different kind of POC testing.

d. Wide confidence intervals.

e. Small population size, low number of events.

f. Risk of performance and detection bias.

g. High risk of performance, detection and attrition bias.

h. A single RCT conducted in a German cardiac surgery centre

i. High risk of selection bias.

j. A single RCT conducted in a Turkish cardiac centre on patients scheduled for CABG surgery.

k. Cui 2010 had a population of paediatric patients undergoing complex cardiac procedures.

**Combined Interventions**

#### Explanations

a. Moderate risk of Performance Bias coming from POC and Cell Salvage trials.

b. Several studies with small sample size and low number of events.

c. A restrictive transfusion threshold had an opposite effect from the other interventions, with an increase in length of stay.

## Re-operation for bleeding (eFigure4f)

Summary Forest plots of pooled effect estimates for all PBM interventions combined as well as for the individual interventions: Iron treatment, Restrictive vs. Liberal threshold, Tranexamic Acid, Cell Salvage, and Point of Care testing) versus controls for the outcome reoperation for bleeding. The results are expressed as: Risk Ratio (RR) along with 95% confidence intervals (CI) and Random effects. The heterogeneity is expressed as I^2^, with P<0.05 considered statistically significant.


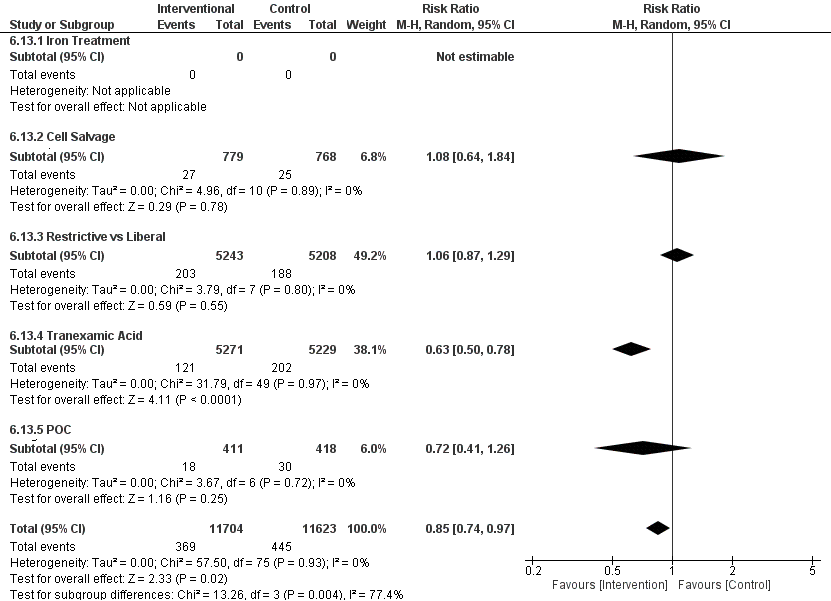


## Re-operation for bleeding GRADE assessment (eTable 15f)

| **Certainty assessment** | | | | | | | | | | | | | | **№ of patients** | | | | | **Effect** | | | | | **Certainty** | | **Importance** |
| --- | --- | --- | --- | --- | --- | --- | --- | --- | --- | --- | --- | --- | --- | --- | --- | --- | --- | --- | --- | --- | --- | --- | --- | --- | --- | --- |
| **№ of studies** | **Study design** | | **Risk of bias** | | | **Inconsistency** | | **Indirectness** | **Imprecision** | | **Other considerations** | | | **Combined blood management interventions** | **standard care** | | | | **Relative (95% CI)** | | **Absolute (95% CI)** | | |
| **Iron Therapy** | | | | | | | | | | | | | | | | | | | | | | | | | | |
| 0 | NA | | NA | | NA | | | NA | | NA | | NA | | NA | NA | | | NA | | | NA | | | NA | | NA |
| **Cell Salvage** | | | | | | | | | | | | | | | | | | | | | | | | | | |
| 14 | randomised trials | | serious ^a^ | | not serious | | | not serious | | serious ^c^ | | none | | 27/779 (3.5%) | 25/768 (3.3%) | | **RR 1.08** (0.64 to 1.84) | | | | **3 more per 1,000** (from 12 fewer to 27 more) | | | ⨁⨁◯◯ LOW | | IMPORTANT |
| **Restrictive vs. Liberal transfusion strategy** | | | | | | | | | | | | | | | | | | | | | | | | | | |
| 8 | randomised trials | | not serious ^a^ | | not serious | | | not serious | | serious ^e^ | | none | | 203/5243 (3.9%) | 188/5208 (3.6%) | | **RR 1.06** (0.87 to 1.29) | | | | **2 more per 1,000** (from 5 fewer to 10 more) | | | ⨁⨁⨁◯ MODERATE | | IMPORTANT |
| **Tranexamic Acid** | | | | | | | | | | | | | | | | | | | | | | | | | | |
| 50 | randomised trials | | not serious ^a^ | | not serious | | | not serious | | serious ^b^ | | none | | 121/5271 (2.3%) | 202/5199 (3.9%) | | **RR 0.62** (0.50 to 0.78) | | | | **15 fewer per 1,000** (from 19 fewer to 9 fewer) | | ⨁⨁⨁◯ MODERATE | | | IMPORTANT |
| **Point of care (POC)** | | | | | | | | | | | | | | | | | | | | | | | | | | |
| 8 | randomised trials | | serious ^a^ | | not serious | | | not serious | | serious ^d^ | | none | | 18/411 (4.4%) | 30/418 (7.2%) | | **RR 0.72** (0.41 to 1.26) | | | | **20 fewer per 1,000** (from 42 fewer to 19 more) | | ⨁⨁◯◯ LOW | | IMPORTANT | |
| **Combined Interventions** | | | | | | | | | | | | | | | | | | | | | | | | | | |
| 79 | | randomised trials | | serious ^a^ | serious | | not serious | | | serious ^b^ | | none | 369/11606 (3.2%) | | 459/11555 (4.0%) | **RR 0.83** (0.73 to 0.95) | | | | **7 fewer per 1,000** (from 11 fewer to 2 fewer) | | ⨁◯◯◯ VERY LOW | | | IMPORTANT | |

**Legend:**

**CI:** Confidence interval; **RR:** Risk ratio; **SMD:** Standardised mean difference; **MD:** Mean difference

**Iron therapy**

#### Explanations

a. Moderate risk of performance and detection bias.

b. Keeler 2017 and Khalafallah 2012 compared different administration methods between groups.

c. High risk of performance bias, moderate risk of detection bias.

d. Different clinical settings.

e. Wide confidence interval - included potential for harm and benefit.

f. Blinding unlikely. Early Termination. Unequal arms due to simple randomisation.

g. Different clinical settings (orthopaedic and general surgery).

h. Confidence intervals included potential for important harm and benefit. Small population size.

i. A single trial (Bernabeu Wittel 2016) conducted in an orthopaedic clinical setting.

j. Risk of performance and detection bias

**Cell Salvage**

#### Explanations

a. High risk of performance and detection bias.

b. Unclear risk of selection bias, high risk of performance and detection bias.

c. Several trials with small population size and low number of events. Wide confidence intervals.

d. Different kind of settings and interventions considered. Control group seem to be favoured in cardiac surgery settings (possibly related to the effect of salvaged blood reinfusion after CPB), while orthopaedic settings (e.g. Elawad 1991, Horstmann 2013) favour cell salvage.

e. Klein 2008 reported patients with creatinine higher than 200mmol/l 2

f. Different clinical settings considered.

g. Reported as "use of inotropes" in most of the studies.

h. Cholette 2013 conducted on a paediatric population.

**Restrictive vs Liberal transfusion strategy**

#### Explanations

a. Unclear risk of performance bias, due to the nature of the intervention itself

b. Tests for heterogeneity show a low p-value and I^2^>80%., however, this is not considered important as the direction of effect is the same for all studies

c. Different surgical disciplines considered in the analysis.

d. I^2^> 80%, p<0·05

e. Large variability in results, several trials had small population size and low number of events.

f. De Almeyda 2015 conducted on a population of oncology patients.

g. Variation in outcome definition

h. Different definition of considered outcomes.

**Tranexamic acid**

#### Explanations

a. Several low quality trials, albeit with a small weight in terms of the results.

b. Several studies with small sample size and low number of events.

c. May be due to several kinds of surgical disciplines considered in the analysis.

d. Different definitions of the outcome considered.

**Point of care testing (POC)**

#### Explanations

a. Moderate risk of selection bias, high risk of performance and detection bias.

b. Avidan 2004 compares POC testing with a laboratory-guided algorithm

c. Different clinical settings considered and different kind of POC testing.

d. Wide confidence intervals.

e. Small population size, low number of events.

f. Risk of performance and detection bias.

g. High risk of performance, detection and attrition bias.

h. A single RCT conducted in a German cardiac surgery centre

i. High risk of selection bias.

j. A single RCT conducted in a Turkish cardiac centre on patients scheduled for CABG surgery.

k. Cui 2010 had a population of paediatric patients undergoing complex cardiac procedures.

**Combined Interventions**

#### Explanations

a. Moderate risk of Performance Bias coming from POC and Cell Salvage trials.

b. Several studies with small sample size and low number of events.

c. A restrictive transfusion threshold had an opposite effect from the other interventions, with an increase in length of stay.

# Funnel plots for transfusion and bleeding outcomes (eFigure 5)

Funnel plots for all the trials reporting the effect on transfusion and bleeding outcomes described in the study protocol. Results are shown for all studies as well as for individual interventions. These funnel plots show significant publication bias for all the transfusion and bleeding outcomes, with the asymmetry primarily driven by the Tranexamic acid studies. The symbols for each intervention type are marked in the subgroups legends of each figure.


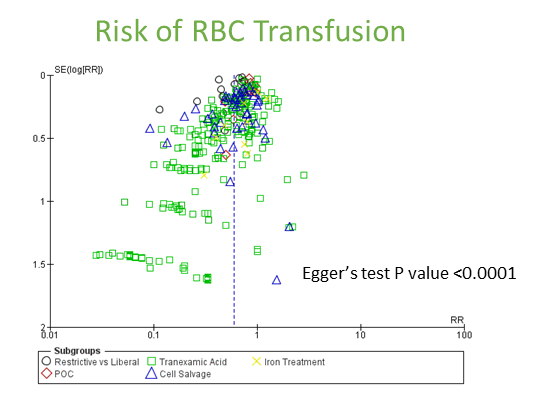


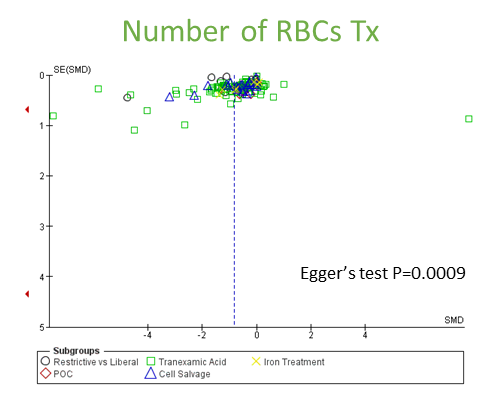


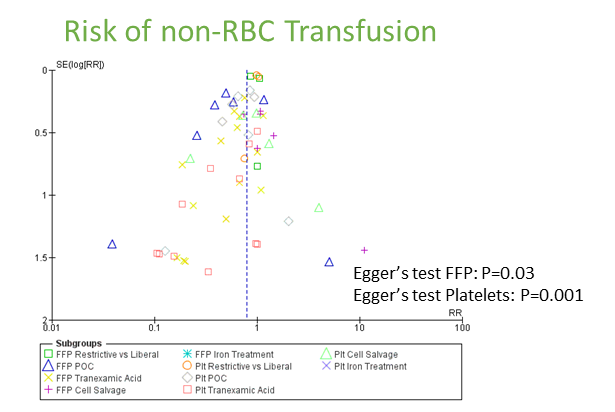


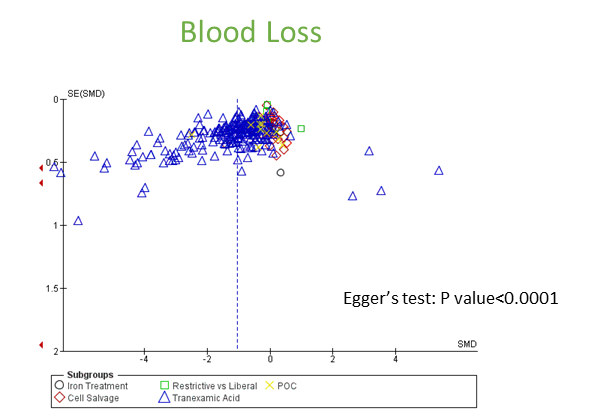


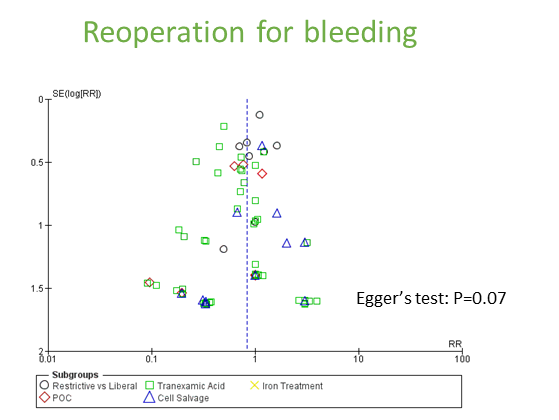


# Subgroup analyses for transfusion and bleeding outcomes (eTable 3)

Pre-specified sub groups included: Bleeding Risk, Anaemia at baseline, Intervention target, cardiovascular disease, Renal disease, Oncological disease and Clinical setting. The effect estimates for different subgroups were broadly similar to that of the primary analysis. The results are reported as: Risk Ratio (RR), 95% Confidence Intervals and p-values for dichotomous outcomes and Standardised Mean Difference (SMD), 95% Confidence Intervals and P values for continuous outcomes. The heterogeneity was reported as I^2^, with P values. The effects considered were random. P values of <0.05 were considered statistically significant. The colour [green] indicates a statistically significant treatment effect in favour of the intervention.

| **Outcome** | **Subgroup/Moderator** | **Type** | **# of studies** | **Patients (n)** | **Output measurement type** | **I^2^** | **P value** | **Result** | **P value** |
| --- | --- | --- | --- | --- | --- | --- | --- | --- | --- |
| **Risk of receiving red cell transfusion** | Bleeding Risk | High | 124 | 32479 | Risk Ratio (M-H, Random, 95% CI) | 65% | <0.001 | 0.73 [0.70, 0.77] | <0.001 |
|  |  | Medium | 180 | 21917 | Risk Ratio (M-H, Random, 95% CI) | 83% | <0.001 | 0.44 [0.40, 0.49] | <0.001 |
|  |  | Low | 9 | 9 | Risk Ratio (M-H, Random, 95% CI) | 54% | 0.03 | 0.43 [0.26, 0.71] | <0.001 |
|  | Anaemia at Baseline | Yes | 36 | 5214 | Risk Ratio (M-H, Random, 95% CI) | 95% | <0.001 | 0.55 [0.44, 0.69] | <0.001 |
|  |  | No | 296 | 51857 | Risk Ratio (M-H, Random, 95% CI) | 70% | <0.001 | 0.62 [0.59, 0.65] | <0.001 |
|  | Intervention Target | Anaemia | 67 | 19702 | Risk Ratio (M-H, Random, 95% CI) | 83% | <0.001 | 0.62 [0.58, 0.67] | <0.001 |
|  |  | Bleeding | 206 | 31608 | Risk Ratio (M-H, Random, 95% CI) | 71% | <0.001 | 0.61 [0.57, 0.65] | <0.001 |
|  | Cardiovascular Disease | Yes | 111 | 34190 | Risk Ratio (M-H, Random, 95% CI) | 80% | <0.001 | 0.72 [0.68, 0.76] | <0.001 |
|  |  | No | 214 | 22227 | Risk Ratio (M-H, Random, 95% CI) | 83% | <0.001 | 0.44 [0.39, 0.49] | <0.001 |
|  | Renal Disease | Yes | 11 | 9314 | Risk Ratio (M-H, Random, 95% CI) | 94% | <0.001 | 0.66 [0.56, 0.79] | <0.001 |
|  |  | No | 299 | 46008 | Risk Ratio (M-H, Random, 95% CI) | 74% | <0.001 | 0.59 [0.55, 0.62] | <0.001 |
|  | Oncological Disease | Yes | 12 | 1136 | Risk Ratio (M-H, Random, 95% CI) | 0% | 0.75 | 0.63 [0.53, 0.76] | 0.002 |
|  |  | No | 312 | 55095 | Risk Ratio (M-H, Random, 95% CI) | 78% | <0.001 | 0.60 [0.57, 0.63] | <0.001 |
|  | Clinical Setting | Cardiac Surgery | 101 | 30647 | Risk Ratio (M-H, Random, 95% CI) | 65% | <0.001 | 0.75 [0.71, 0.78] | <0.001 |
|  |  | Orthopaedic Surgery | 180 | 21009 | Risk Ratio (M-H, Random, 95% CI) | 87% | <0.001 | 0.43 [0.38, 0.48] | <0.001 |
|  |  | Hepatobiliary Surgery | 2 | 296 | Risk Ratio (M-H, Random, 95% CI) | 93% | <0.001 | 0.16 [0.00, 34.51] | 0.51 |
|  |  | Urogenital Surgery | 8 | 1333 | Risk Ratio (M-H, Random, 95% CI) | 28% | 0.21 | 0.56 [0.35, 0.90] | 0.02 |
|  |  | Other | 32 | 2988 | Risk Ratio (M-H, Random, 95% CI) | 66% | <0.001 | 0.59 [0.48, 0.73] | <0.001 |
| **Numbers of red cells transfused** | Bleeding Risk | High | 115 | 24176 | Std· Mean Difference (IV, Random, 95% CI) | 96% | <0.001 | -0.73 [-0.89, -0.57] | <0.001 |
|  |  | Medium | 114 | 14470 | Std· Mean Difference (IV, Random, 95% CI) | 95% | <0.001 | -1.14 [-1.38, -0.90] | <0.001 |
|  |  | Low | 2 | 256 | Std· Mean Difference (IV, Random, 95% CI) | 56% | 0.13 | -1.03 [-1.45, -0.61] | <0.001 |
|  | Anaemia at Baseline | Yes | 25 | 4058 | Std· Mean Difference (IV, Random, 95% CI) | 92% | <0.001 | -0.85 [-1.25, -0.45] | <0.001 |
|  |  | No | 206 | 34924 | Std· Mean Difference (IV, Random, 95% CI) | 96% | <0.001 | -0.89 [-1.03, -0.76] | <0.001 |
|  | Intervention Target | Anaemia | 58 | 18353 | Std· Mean Difference (IV, Random, 95% CI) | 97% | <0.001 | -1.04 [-1.28, -0.81] | <0.001 |
|  |  | Bleeding | 130 | 16631 | Std· Mean Difference (IV, Random, 95% CI) | 94% | <0.001 | -0.80 [-0.98, -0.62] | <0.001 |
|  | Cardiovascular Disease | Yes | 101 | 25720 | Std· Mean Difference (IV, Random, 95% CI) | 96% | <0.001 | -0.78 [-0.94, -0.62] | <0.001 |
|  |  | No | 129 | 13112 | Std· Mean Difference (IV, Random, 95% CI) | 95% | <0.001 | -1.01 [-1.24, -0.79] | <0.001 |
|  | Renal Disease | Yes | 12 | 9386 | Std· Mean Difference (IV, Random, 95% CI) | 98% | <0.001 | -0.63 [-1.11, -0.15] | 0.01 |
|  |  | No | 220 | 35604 | Std· Mean Difference (IV, Random, 95% CI) | 95% | <0.001 | -0.87 [-1.00, -0.73] | <0.001 |
|  | Oncological Disease | Yes | 6 | 499 | Std· Mean Difference (IV, Random, 95% CI) | 64% | 0.03 | -0.94 [-1.30, -0.58] | 0.002 |
|  |  | No | 221 | 37993 | Std· Mean Difference (IV, Random, 95% CI) | 96% | <0.001 | -0.90 [-1.03, -0.77] | <0.001 |
|  | Clinical Setting | Cardiac Surgery | 92 | 22664 | Std· Mean Difference (IV, Random, 95% CI) | 96% | <0.001 | -0.83 [-1.01, -0.65] | <0.001 |
|  |  | Orthopaedic Surgery | 110 | 14028 | Std· Mean Difference (IV, Random, 95% CI) | 96% | <0.001 | -1.13 [-1.37, -0.89] | <0.001 |
|  |  | Hepatobiliary Surgery | 5 | 202 | Std· Mean Difference (IV, Random, 95% CI) | 30% | 0.22 | -0.38 [-0.73, -0.03] | 0.04 |
|  |  | Urogenital Surgery | 1 | 200 | Std· Mean Difference (IV, Random, 95% CI) | N/A | N/A | -1.06 [-1.36, -0.77] | <0.001 |
|  |  | Other | 23 | 1808 | Std· Mean Difference (IV, Random, 95% CI) | 91% | <0.001 | -0.48 [-0.85, -0.11] | 0.009 |
| **Risk of receiving FFP** | Bleeding Risk | High | 11 | 1348 | Risk Ratio (M-H, Random, 95% CI) | 0% | 0.47 | 0.63 [0.44, 0.89] | 0.01 |
|  |  | Medium | 2 | 158 | Risk Ratio (M-H, Random, 95% CI) | 21% | 0.26 | 0.64 [0.15, 2.79] | 0.55 |
|  |  | Low | 1 | 153 | Risk Ratio (M-H, Random, 95% CI) | N/A | N/A | 4.81 [0.23, 98.56] | 0.31 |
|  | Anaemia at Baseline | Yes | 0 | 0 | Risk Ratio (M-H, Random, 95% CI) | N/A | N/A | Not estimable | Not estimable |
|  |  | No | 14 | 1532 | Risk Ratio (M-H, Random, 95% CI) | 0% | 0.68 | 0.58 [0.47, 0.72] | <0.001 |
|  | Intervention Target | Anaemia | 1 | 58 | Risk Ratio (M-H, Random, 95% CI) | N/A | N/A | 3 [0.13, 70.74] | 0.5 |
|  |  | Bleeding | 13 | 1474 | Risk Ratio (M-H, Random, 95% CI) | 0% | 0.68 | 0.62 [0.44, 0.87] | <0.001 |
|  | Cardiovascular Disease | Yes | 9 | 900 | Risk Ratio (M-H, Random, 95% CI) | 0% | 0.73 | 0.55 [0.43, 0.69] | <0.001 |
|  |  | No | 5 | 632 | Risk Ratio (M-H, Random, 95% CI) | 0% | 0.48 | 0.81 [0.47, 1.40] | 0.46 |
|  | Renal Disease | Yes | 0 | 0 | Risk Ratio (M-H, Random, 95% CI) | N/A | N/A | Not estimable | Not estimable |
|  |  | No | 14 | 1532 | Risk Ratio (M-H, Random, 95% CI) | 0% | 0.68 | 0.58 [0.47, 0.72] | <0.001 |
|  | Oncological Disease | Yes | 1 | 100 | Risk Ratio (M-H, Random, 95% CI) | N/A | N/A | 0.44 [0.15, 1.35] | 0.15 |
|  |  | No | 13 | 1432 | Risk Ratio (M-H, Random, 95% CI) | 0% | 0.62 | 0.59 [0.47, 0.73] | <0.001 |
|  | Clinical Setting | Cardiac Surgery | 10 | 1231 | Risk Ratio (M-H, Random, 95% CI) | 0% | 0.55 | 0.58 [0.47, 0.73] | <0.001 |
|  |  | Orthopaedic Surgery | 0 | 0 | Risk Ratio (M-H, Random, 95% CI) | N/A | N/A | Not estimable | Not estimable |
|  |  | Hepatobiliary Surgery | 0 | 0 | Risk Ratio (M-H, Random, 95% CI) | N/A | N/A | Not estimable | Not estimable |
|  |  | Urogenital Surgery | 0 | 0 | Risk Ratio (M-H, Random, 95% CI) | N/A | N/A | Not estimable | Not estimable |
|  |  | Other | 4 | 301 | Risk Ratio (M-H, Random, 95% CI) | 0% | 0.49 | 0.57 [0.24, 1.37] | 0.21 |
| **Risk of receiving Platelets** | Bleeding Risk | High | 11 | 1323 | Risk Ratio (M-H, Random, 95% CI) | 0% | 0.51 | 0.64 [0·36, 1·12] | 0.12 |
|  |  | Medium | 1 | 58 | Risk Ratio (M-H, Random, 95% CI) | N/A | N/A | 3 [0.13, 70.74] | 0.5 |
|  |  | Low | 0 | 0 | Risk Ratio (M-H, Random, 95% CI) | N/A | N/A | Not estimable | Not estimable |
|  | Anaemia at Baseline | Yes | 0 | 0 | Risk Ratio (M-H, Random, 95% CI) | N/A | N/A | Not estimable | Not estimable |
|  |  | No | 12 | 1381 | Risk Ratio (M-H, Random, 95% CI) | 0% | 0.52 | 0.67 [0.38, 1.17] | 0.16 |
|  | Intervention Target | Anaemia | 1 | 58 | Risk Ratio (M-H, Random, 95% CI) | N/A | N/A | 3 [0.13, 70.74] | 0.5 |
|  |  | Bleeding | 11 | 1323 | Risk Ratio (M-H, Random, 95% CI) | 0% | 0.51 | 0.64 [0.36, 1.12] | 0.12 |
|  | Cardiovascular Disease | Yes | 8 | 849 | Risk Ratio (M-H, Random, 95% CI) | 0% | 0.43 | 0.66 [0.3, 1.46] | 0.3 |
|  |  | No | 4 | 532 | Risk Ratio (M-H, Random, 95% CI) | 13% | 0.33 | 0.62 [0.25, 1.53] | 0.3 |
|  | Renal Disease | Yes | 0 | 0 | Risk Ratio (M-H, Random, 95% CI) | N/A | N/A | Not estimable | Not estimable |
|  |  | No | 12 | 1381 | Risk Ratio (M-H, Random, 95% CI) | 0% | 0.52 | 0.67 [0.38, 1.17] | 0.16 |
|  | Oncological Disease | Yes | 0 | 0 | Risk Ratio (M-H, Random, 95% CI) | N/A | N/A | Not estimable | Not estimable |
|  |  | No | 12 | 1381 | Risk Ratio (M-H, Random, 95% CI) | 0% | 0.52 | 0.67 [0.38, 1.17] | 0.16 |
|  | Clinical Setting | Cardiac Surgery | 9 | 1180 | Risk Ratio (M-H, Random, 95% CI) | 0% | 0.58 | 0.73 [0.41, 1.31] | 0.29 |
|  |  | Orthopaedic Surgery | 0 | 0 | Risk Ratio (M-H, Random, 95% CI) | N/A | N/A | Not estimable | Not estimable |
|  |  | Hepatobiliary Surgery | 0 | 0 | Risk Ratio (M-H, Random, 95% CI) | N/A | N/A | Not estimable | Not estimable |
|  |  | Urogenital Surgery | 0 | 0 | Risk Ratio (M-H, Random, 95% CI) | N/A | N/A | Not estimable | Not estimable |
|  |  | Other | 3 | 201 | Risk Ratio (M-H, Random, 95% CI) | 28% | 0.25 | 0.33 [0.04, 2.51] | 0.29 |
| **Perioperative blood loss** | Bleeding Risk | High | 133 | 13053 | Std· Mean Difference (IV, Random, 95% CI) | 95% | <0.0001 | -0.89 [-1.05, -0.72] | <0.001 |
|  |  | Medium | 176 | 18739 | Std· Mean Difference (IV, Random, 95% CI) | 93% | <0.001 | -1.11 [-1.25, -0.98] | <0.001 |
|  |  | Low | 19 | 1845 | Std· Mean Difference (IV, Random, 95% CI) | 95% | <0.001 | -1.95 [-2.49, -1.41] | <0.001 |
|  | Anaemia at Baseline | Yes | 35 | 4550 | Std· Mean Difference (IV, Random, 95% CI) | 95% | <0.001 | -0.75 [-1.07, -0.42] | <0.001 |
|  |  | No | 289 | 28891 | Std· Mean Difference (IV, Random, 95% CI) | 94% | <0.001 | -1.09 [-1.20, -0.98] | <0.001 |
|  | Intervention Target | Anaemia | 42 | 7712 | Std· Mean Difference (IV, Random, 95% CI) | 71% | <0.001 | -0.03 [-0.13, 0.07] | 0.56 |
|  |  | Bleeding | 285 | 27555 | Std· Mean Difference (IV, Random, 95% CI) | 94% | <0.001 | -1.22 [-1.34, -1.11] | <0.001 |
|  | Cardiovascular Disease | Yes | 108 | 12986 | Std· Mean Difference (IV, Random, 95% CI) | 95% | <0.001 | -0.86 [-1.03, -0.69] | <0.001 |
|  |  | No | 220 | 20651 | Std· Mean Difference (IV, Random, 95% CI) | 93% | <0.001 | -1.17 [-1.29, -1.04] | <0.001 |
|  | Renal Disease | Yes | 7 | 2717 | Std· Mean Difference (IV, Random, 95% CI) | 85% | <0.001 | -0.27 [-0.56, 0.02] | -<0.001 |
|  |  | No | 326 | 33523 | Std· Mean Difference (IV, Random, 95% CI) | 94% | <0.001 | -1.07 [-1.17, -0.96] | <0.001 |
|  | Oncological Disease | Yes | 13 | 1362 | Std· Mean Difference (IV, Random, 95% CI) | 92% | <0.001 | -0.57 [-1.09, -0.06] | 0.03 |
|  |  | No | 312 | 32205 | Std· Mean Difference (IV, Random, 95% CI) | 94% | <0.001 | -1.08 [-1.18, -0.97] | <0.001 |
|  | Clinical Setting | Cardiac Surgery | 103 | 10838 | Std· Mean Difference (IV, Random, 95% CI) | 95% | <0.001 | -0.93 [-1.12, -0.74] | <0.001 |
|  |  | Orthopaedic Surgery | 172 | 17956 | Std· Mean Difference (IV, Random, 95% CI) | 94% | <0.001 | -1.18 [-1.32, -1.05] | <0.001 |
|  |  | Hepatobiliary Surgery | 4 | 307 | Std· Mean Difference (IV, Random, 95% CI) | 0% | 0.94 | -0.44 [-0.66, -0.21] | <0.001 |
|  |  | Urogenital Surgery | 6 | 1062 | Std· Mean Difference (IV, Random, 95% CI) | 92% | <0.001 | -1.15 [-1.70, -0.59] | <0.001 |
|  |  | Other | 42 | 3360 | Std· Mean Difference (IV, Random, 95% CI) | 95% | <0.001 | -1.01 [-1.36, -0.65] | <0.001 |
| **Re-operation for bleeding** | Bleeding Risk | High | 67 | 15786 | Risk Ratio (M-H, Random, 95% CI) | 0% | 0.99 | 0.88 [0.75, 1.02] | 0.25 |
|  |  | Medium | 6 | 2399 | Risk Ratio (M-H, Random, 95% CI) | 0% | 0.6 | 1.22 [0.75, 1.97] | 0.42 |
|  |  | Low | 2 | 181 | Risk Ratio (M-H, Random, 95% CI) | 54% | 0.14 | 0.96 [0.04, 21.78] | 0.98 |
|  | Anaemia at Baseline | Yes | 3 | 2100 | Risk Ratio (M-H, Random, 95% CI) | 8% | 0.34 | 0.64 [0.30, 1.39] | 0.26 |
|  |  | No | 72 | 16266 | Risk Ratio (M-H, Random, 95% CI) | 0% | 0.95 | 0.92 [0.79, 1.07] | 0.54 |
|  | Intervention Target | Anaemia | 20 | 11839 | Risk Ratio (M-H, Random, 95% CI) | 0% | 0.97 | 1·06 [0.88, 1.28] | 0.52 |
|  |  | Bleeding | 55 | 6527 | Risk Ratio (M-H, Random, 95% CI) | 0% | 0.99 | 0.68 [0.54, 0.87] | 0.03 |
|  | Cardiovascular Disease | Yes | 67 | 17380 | Risk Ratio (M-H, Random, 95% CI) | 0% | 0.99 | 0.88 [0.75, 1.03] | 0.27 |
|  |  | No | 8 | 986 | Risk Ratio (M-H, Random, 95% CI) | 0% | 0.6 | 1.20 [0.73, 1.99] | 0.47 |
|  | Renal Disease | Yes | 7 | 8443 | Risk Ratio (M-H, Random, 95% CI) | 0% | 0.82 | 1.10 [0.90, 1.35] | 0.36 |
|  |  | No | 68 | 9923 | Risk Ratio (M-H, Random, 95% CI) | 0% | 1 | 0.74 [0.60, 0.91] | 0.04 |
|  | Oncological Disease | Yes | 2 | 258 | Risk Ratio (M-H, Random, 95% CI) | 45% | 0.18 | 1.05 [0.26, 4.26] | 0.95 |
|  |  | No | 73 | 18108 | Risk Ratio (M-H, Random, 95% CI) | 0% | 0.99 | 0.89 [0.76, 1.03] | 0.29 |
|  | Clinical Setting | Cardiac Surgery | 64 | 15448 | Risk Ratio (M-H, Random, 95% CI) | 0% | 0.99 | 0.90 [0.77, 1.05] | 0.43 |
|  |  | Orthopaedic Surgery | 4 | 2295 | Risk Ratio (M-H, Random, 95% CI) | 0% | 0.52 | 1.00 [0.53, 1.88] | 0.99 |
|  |  | Hepatobiliary Surgery | 1 | 82 | Risk Ratio (M-H, Random, 95% CI) | N/A | N/A | 0.71 [0.17, 2.99] | 0.65 |
|  |  | Urogenital Surgery | 0 | 0 | Risk Ratio (M-H, Random, 95% CI) | N/A | N/A | Not estimable | Not estimable |
|  |  | Other | 6 | 541 | Risk Ratio (M-H, Random, 95% CI) | 36% | 0.18 | 0.68 [0.28, 1.66] | 0.4 |

# Sensitivity analysis for transfusion and bleeding outcomes based on exclusion of studies at high risk of bias (eTable 4)

Analysis of primary and secondary outcomes excluding all trials considered at high risk of bias. In the assessment of overall risk of bias, studies at high risk of bias for any category were excluded. Effect estimates were consistent with those of the primary analysis.

The results are reported as: Risk Ratio (RR), 95% Confidence Intervals and P values for dichotomous outcomes and Standardised Mean Difference (SMD), 95% Confidence Intervals and p-values for continuous outcomes. Heterogeneity was expressed as I^2^ and P values. The effects considered were random. P values of <0.05 were considered statistically significant. The colour [green] indicates a statistically significant treatment effect in favour of the intervention groups.

| **Outcome and subcategories** | **# of studies** | **Patients (n)** | **Output measurement type** | **I^2^** | **P value** | **Result** | **P value** |
| --- | --- | --- | --- | --- | --- | --- | --- |
| **Risk of receiving red cell transfusion** | 216 | 36902 | Risk Ratio (M-H, Random, 95% CI) | 73% | <0.001 | 0.60 [0.56, 0.63] | <0.001 |
| Iron Treatment | 4 | 665 | Risk Ratio (M-H, Random, 95% CI) | 24% | 0.27 | 0.71 [0.50, 1.00] | 0.08 |
| Cell Salvage | 5 | 623 | Risk Ratio (M-H, Random, 95% CI) | 0% | 0.46 | 0.53 [0.41, 0.70] | <0.001 |
| Restrictive vs Liberal | 16 | 12130 | Risk Ratio (M-H, Random, 95% CI) | 94% | <0.001 | 0.56 [0.49, 0.64] | <0.001 |
| Tranexamic Acid | 188 | 23244 | Risk Ratio (M-H, Random, 95% CI) | 64% | <0.001 | 0.59 [0.54, 0.63] | <0.001 |
| POC | 4 | 240 | Risk Ratio (M-H, Random, 95% CI) | 60% | 0.06 | 0.70 [0.46, 1.07] | 0.1 |
| **Numbers of red cells transfused** | 147 | 29092 | Std· Mean Difference (IV, Random, 95% CI) | 96% | <0.001 | -0.82 [-0.97, -0.66] | <0.001 |
| Iron Treatment | 4 | 665 | Std· Mean Difference (IV, Random, 95% CI) | 77% | 0.01 | -0.52 [-1.03, -0.01] | 0.02 |
| Cell Salvage | 6 | 738 | Std· Mean Difference (IV, Random, 95% CI) | 86% | <0.001 | -0.79 [-1.38, -0.20] | 0.009 |
| Restrictive vs Liberal | 12 | 11327 | Std· Mean Difference (IV, Random, 95% CI) | 99% | <0.001 | -1.22 [-1.82, -0.62] | <0.001 |
| Tranexamic Acid | 122 | 16148 | Std· Mean Difference (IV, Random, 95% CI) | 94% | <0.001 | -0.81 [-0.97, -0.64] | <0.001 |
| POC | 3 | 214 | Std· Mean Difference (IV, Random, 95% CI) | 0% | 0.43 | -0.30 [-0.63, 0.03] | 0.07 |
| **Risk of receiving FFP** | 21 | 9012 | Risk Ratio (M-H, Random, 95% CI) | 45% | 0.01 | 0.73 [0.61, 0.88] | <0.001 |
| Iron Treatment | 0 | 0 | Risk Ratio (M-H, Random, 95% CI) | N/A | N/A | Not estimable | N/A |
| Cell Salvage | 0 | 0 | Risk Ratio (M-H, Random, 95% CI) | 0% | 0.52 | Not estimable | N/A |
| Restrictive vs Liberal | 3 | 6913 | Risk Ratio (M-H, Random, 95% CI) | 59% | 0.09 | 0.95 [0.8, 1.12] | 0.51 |
| Tranexamic Acid | 16 | 1968 | Risk Ratio (M-H, Random, 95% CI) | 0% | 0.76 | 0.62 [0.51, 0.75] | <0.001 |
| POC | 2 | 131 | Risk Ratio (M-H, Random, 95% CI) | 0% | 0.82 | 0.27 [0.1, 0.73] | 0.009 |
| **Risk of receiving Platelets** | 17 | 8598 | Risk Ratio (M-H, Random, 95% CI) | 13% | 0.3 | 0.97 [0.87, 1.08] | 0.51 |
| Iron Treatment | 0 | 0 | Risk Ratio (M-H, Random, 95% CI) | N/A | N/A | Not estimable | N/A |
| Cell Salvage | 0 | 0 | Risk Ratio (M-H, Random, 95% CI) | N/A | N/A | Not estimable | N/A |
| Restrictive vs Liberal | 3 | 6910 | Risk Ratio (M-H, Random, 95% CI) | 0% | 0.66 | 1.00 [0.93, 1.07] | 0.98 |
| Tranexamic Acid | 12 | 1557 | Risk Ratio (M-H, Random, 95% CI) | 0% | 0.77 | 0.58 [0.35, 0.97] | 0.04 |
| POC | 2 | 131 | Risk Ratio (M-H, Random, 95% CI) | 79% | 0.03 | 1.65 [0.07, 38.72] | p=0.76 |
| **Perioperative blood loss** | 203 | 20995 | Std· Mean Difference (IV, Random, 95% CI) | 95% | <0.001 | -1.22 [-1.35, -1.08] | <0.001 |
[truncated: 583,748 more chars]
